# Supplementary material for: Urban-rural differences in hypertension prevalence in low-income and middle-income countries, 1990–2020: A systematic review and meta-analysis
Source: PLoS Med. 2022 Aug 25;19(8):e1004079. doi: 10.1371/journal.pmed.1004079 (PMC9410549; doi:10.1371/journal.pmed.1004079)
Supplement: S1 Protocol — (PDF) [file pmed.1004079.s002.pdf]

S1 Protocol

Urban-rural differences in hypertension prevalence in low-income and middle-income countries, 1990-2020: a systematic review and meta-analysis

Table of Contents

e-Methods: Overall **2**

e-Methods: Search strategy **4**

e-Methods: Data extraction **5**

e-Methods: Additional sources of data **10**

e-Methods: Data Preparation and Statistical analysis **11**

REFERENCES **13**

e-Methods: Overall

We followed PRISMA and Cochrane Handbook guidelines and this review is registered on PROSPERO: 2018 CRD42018091671.

We aimed to evaluate the following research questions:

What is the effect of urban environments on hypertension prevalence in low and middle-income countries? Is the effect of urban environments on hypertension prevalence dependent on country-level socioeconomic development?

We aimed to estimate the prevalence of hypertension/high blood pressure in urban and rural areas of low and middle-income countries and pooled them by conducting a meta-analysis. We also aimed to evaluate whether part of this potential difference can be explained by some country-level characteristics and its temporal evolution (e.g., HDI, GDP, income status, year of data collection) by conducting a meta-regression.

Inclusion/Exclusion criteria

We included studies reporting prevalence of hypertension/high-blood pressure that included participants 15 years and older, from a general population representative of the target population including 400 or more participants. These studies must come from low-income and/or middle-income countries as classified by the World Bank, 2018 fiscal year. Studies must have collected data from 1990 onwards (period of data collection). Prevalence in urban and rural areas must have been evaluated using similar protocols for sampling and blood pressure measurements, ideally simultaneously evaluated and not apart more than 4 years.

We excluded abstracts, not original data, data from high-income countries, any data collected before 01/01/1990, language not English, data for participants below 15 years of age, studies only conducted in rural or urban areas, studies that included only males or females, or from specific populations (e.g., pregnant women, tertiary hospitals, specific clinic, etc), studies that reported only self-reported or medical diagnosed hypertension, studies not reporting any urban-rural contrast in hypertension/high-blood pressure or SBP/DBP values, studies that reported between countries differences or international migrant contrasts. Finally, when information needed to consider eligibility was missing the study was excluded. We carefully revised all studies from the same cohort/survey-year to avoid any duplicate and the selected one was the best match with our definitions, the larger in terms of total sample size or the earliest published.

Urban and rural definition might be challenging and varies according to each country. We aimed to include studies with a clear contrast between urban and rural areas. To guide the inclusion/exclusion and data extraction, we develop the following guide during the systematic review protocol:

Area	Scenario	Decision
Intermediate levels of urbanicity	Study compared participants living in **Semi-urban** area x **Urban** areas	**Exclude**
	Study compared participants living in **Semi-urban** area x **Rural** areas	**Exclude**
	Study compared participants living in **Rural** x **Semi-urban** x **Urban** areas	**Include** and extract data on **rural** and **urban.**
	Study compared participants living in **Rural** x **Urban** x **Slum** areas	Include because slums are largely urban. **Merge prevalence for (urban + slum) x rural.**
Score to define levels of urbanicity	Study stratified participants based on a score on low, medium, high (or other ordinal levels) of urbanicity / urbanization	Include and compare **low** vs **high**, or the **extremes.**
	Study stratified participants based on an urbanization index, defined as percentage of life spent in a city. Not category 0%, but 0-25%.	Exclude (if not a spatial contrast in exposure). There is no way to define urban and rural.
Migrants	Study compared participants living in **Rural** x **Rural-to-Urban** migrants x **Urban** areas	Include as rural to urban migrants are, **currently**, urban dwellers, in this cross-sectional snapshot. So, actually they are urban upon survey. **Merge prevalence of (urban + rural to urban migrants**) x rural.
	Study compared participants living in **Always Rural** x **Always Urban** x **Mixed**	Include always rural and always urban, which are clear. The mixed group is not clear and was excluded. **Compare always rural and always urban.**
Other rural labelling	Study compared participants living in **Rural** x **Urban** x **Tribal** areas	Include as tribal is rural in those scenarios. **Merge tribal + rural.**
	Study compared participants living in **Urban** x **Indigenous**	Include if the **indigenous are non-urban indigenous and represents the main rural population in area.**
	Study compared participants living in **rural** (farmer) x **nomads** x **Urban** (citizens).	Include and **compare rural x urban. Exclude nomads.**
	Study compared tribal participants as semi-nomadic deep forest hunter gatherers (rural) x resettled communities x urbanized city-fringe dwellers	Include and **compare rural x urbanized city-fringe.**

Main outcome(s)

The primary outcome was prevalence of hypertension. We included studies that applied standard methods of measuring blood pressure and defined hypertension as systolic pressure more than or equal to 140 mmHg or diastolic blood pressure more than or equal to 90 mmHg and/or use of antihypertensive medication. When the study did not report hypertension as ≥140/90 mmHg (e.g., old WHO criteria as 160/95 mmHg or only SBP/DBP means), and we obtained enough information to convert it to ≥140/90 mmHg, we applied validated equations to derive the prevalence.[1] The secondary outcomes were mean SBP/DBP.

e-Methods: Search strategy

We carried out a systematic literature search in the electronic databases PubMed, Web of Science, Scopus and Embase. The literature search was conducted in May 2018. The time limit was set between 01/01/1990 to 01/05/2018. During the peer review, we updated the same search up to 10/03/2022. The search was conducted by both OR and AK. All the duplication, abstract & full text screening was done in CONVIDENCE (Covidence systematic review software, Veritas Health Innovation, Melbourne, Australia. Available at www.covidence.org). The process for screening all titles & abstracts that met the inclusion criteria was blinded and done by OR and AK. The full manuscripts were also screened by the same reviewers to make the final decision for all included studies. Any disagreements were resolved by consensus between OR & AK and the senior author (CT). In addition, during the full text review, we also did a manual search for potentially relevant references in the reference sections of previous original studies or systematic reviews of the literature on the effect of urban environments on hypertension prevalence in low and middle-income countries.

The search included the following terms (example for Scopus):

TOPIC: ("Blood pressure" OR "Systolic blood pressure" OR "Diastolic blood pressure" OR Hypertension OR Hypertensive OR "Elevated blood pressure" OR "Altered blood pressure" OR "Essential hypertension" OR "Blood pressure determination" OR Hypertens* OR "SBP" OR "DBP" OR "BP" OR "HTN") AND TOPIC: (Urban OR Rural OR "urban-rural" OR "urban rural" OR "Built environment" OR "Built environmental" OR "Built environments" OR "Physical environment" OR "Physical environmental" OR "Physical environments" OR Urbanization OR Urbanisation OR Urban* OR "Urban form" OR "Population density" OR "Residential location") AND TOPIC: ("Lower middle income country" OR "Lower middle income countries" OR "LMIC" OR "upper middle income country" OR "upper middle income countries" OR "middle income countries" OR "low income country" OR "low income countries" OR LIC OR "developing country" OR "developing countries" OR Afghanistan OR Albania OR Algeria OR American Samoa OR Samoa OR Amelika Sāmoa OR Angola OR Argentina OR Armenia OR Azerbaijan OR Bangladesh OR Belarus OR Belize OR Benin OR Bhutan OR Bolivia OR Bosnia and Herzegovina OR Bosnia OR Herzegovina OR Botswana OR Brazil OR Bulgaria OR Burkina Faso OR Burundi OR Cabo Verde OR Cambodia OR Cameroon OR Central African Republic OR Chad OR China OR Colombia OR Comoros OR Congo OR Democratic Republic of Congo OR Republic of Congo OR Congo Braziville OR Costa Rica OR Côte d'Ivoire OR Croatia OR Cuba OR Djibouti OR Dominica OR Dominican Republic OR Ecuador OR Egypt OR Arab Republic of Egypt OR El Salvador OR Equatorial Guinea OR Eritrea OR Ethiopia OR Fiji OR Gabon OR Gambia OR The Republic of Gambia OR Georgia OR Ghana OR Grenada OR Guatemala OR Guinea OR Guinea-Bissau OR Guyana OR Haiti OR Honduras OR India OR Indonesia OR Iran OR Islamic Rep. of Iran OR Islamic Republic OR Iraq OR Jamaica OR Jordan OR Kazakhstan OR Kenya OR Kiribati OR Korea OR Dem. People's Rep. of Korea OR Democratic People's Republic of Korea OR Kosovo OR Kyrgyz Republic OR Kyrgyzstan OR Kirghizia OR Lao PDR OR Lao People's Democratic Republic OR Laos OR Lebanon OR Lesotho OR Liberia OR Libya OR Macedonia OR Republic of

Macedonia OR Madagascar OR Malawi OR Malaysia OR Maldives OR Mali OR Marshall Islands OR Mauritania OR Mauritius OR Mexico OR Micronesia OR Federated States of Micronesia OR Moldova OR Mongolia OR Montenegro OR Morocco OR Mozambique OR Myanmar OR Namibia OR Nauru OR Nepal OR Nicaragua OR Niger OR Nigeria OR Pakistan OR Panama OR Papua New Guinea OR Paraguay OR Peru OR Philippines OR Romania OR Russian Federation OR Russia OR Rwanda OR Samoa OR São Tomé and Principe OR Senegal OR Serbia OR Sierra Leone OR Solomon Islands OR Somalia OR South Africa OR South Sudan OR Sri Lanka OR St. Lucia OR Saint Lucia OR St. Vincent and the Grenadines OR Saint Vincent and the Grenadines OR Saint Vincent OR Sudan OR Suriname OR Swaziland OR Syrian Arab Republic OR Syria OR Tajikistan OR Tanzania OR Thailand OR Timor-Leste OR Timor Leste OR Togo OR Tonga OR Tunisia OR Turkey OR Turkmenistan OR Tuvalu OR Uganda OR Ukraine OR Uzbekistan OR Vanuatu OR Venezuela OR Republic of Venezuela OR Vietnam OR West Bank and Gaza OR West Bank OR Gaza OR Yemen OR Republic of Yemen OR Zambia OR Zimbabwe)
Refined by: LANGUAGES: (ENGLISH OR UNSPECIFIED) AND PUBLICATION YEARS: (2018 OR 2010 OR 2002 OR 1995 OR 2017 OR 2009 OR 2001 OR 1994 OR 2016 OR 2008 OR 2000 OR 1993 OR 2015 OR 2007 OR 1999 OR 1992 OR 2014 OR 2006 OR 1998 OR 1991 OR 2013 OR 2005 OR 1997 OR 1990 OR 2012 OR 2004 OR 1996 OR 2011 OR 2003)
Timespan: 1989-2018.
Search language=Auto

* PUBLICATION YEARS were updated to 2022 in the new search.

e-Methods: Data extraction

A standardised electronic data collection form was developed in REDcap (Research Electronic Data Capture) which was first piloted by OR, AK and CT. The data extraction followed a prespecified protocol and was conducted independently by each extractor blinded to extraction data of other extractors. All data extractors (OTR, AK, CDG, FAO, JIH, AC, CT) were trained by the first author (OR) and paired in four teams of two data extractors each. The first 10 papers were piloted by each data extractor for clarifications and refinement. If a study reported more than one survey (e.g. different years or different countries) the data was extracted for all surveys. If there was more than one paper from the same cohort/survey, we used the paper, which provided the most comprehensive and clear information on HTN prevalence for urban-rural contrast. When the information was not available in the main paper, we used data from additional papers from the same cohort and supplementary data cited in the main paper (e.g., raw data publicly available, WHO STEPS Country Reports) for extracting the relevant information. We extracted crude and adjusted prevalence estimates when both estimates were available. We also extracted sex-specific data when available. Data were then exported for standardisation and resolving conflicts. Disagreements between pairs was checked by OR and AK and conflicts discussed with CT.

e-Methods: Risk of Bias assessment

We used the OHAT Bias Tool to evaluate the risk of bias (RoB). We evaluated 3 domains: “Selection Bias / Sampling”, “Detection bias/Measurement error – Exposure”, and “Detection bias/Measurement error – Outcome”. We choose these domains because of research question and characteristics of our systematic review: to assess prevalence (sampling) of hypertension/high-blood pressure (outcome) in two contrasting areas (exposure).

1) Brief definition of RoB from OHAT manual:

"This risk-of-bias tool evaluates internal validity – the assessment of whether the design and conduct of the study compromised the credibility of the link between exposure and outcome (Higgins and Green 2011, IOM 2011, Viswanathan et al. 2012). There are other aspects of a study that will impact its utility for addressing the research question such as external validity – indirectness or applicability, which are addressed elsewhere in the OHAT Approach."

2) Bias evaluated in 4 groups:

**Definitely Low risk of bias:**<br>For Cohort and Cross-Sectional	There is direct evidence that subjects (both exposed and non-exposed) were similar (e.g., recruited from the same eligible population, recruited with the same method of ascertainment using the same inclusion and exclusion criteria, and were of similar age and health status), recruited within the same time frame, and had the similar participation/response rates.<br>**Note:** A study will be considered low risk of bias if baseline characteristics of groups differed but these differences were considered as potential confounding or stratification variables.
**Probably Low risk of bias:**<br>For Cohort and Cross-Sectional	There is indirect evidence that subjects (both exposed and non-exposed) were similar (e.g., recruited from the same eligible population, recruited with the same method of ascertainment using the same inclusion and exclusion criteria, and were of similar age and health status), recruited within the same time frame, and had the similar participation/response rates, **OR** differences between groups would not appreciably bias results.
**Probably High risk of bias:**<br>For Cohort and Cross-Sectional	There is indirect evidence that subjects (both exposed and non-exposed) were not similar, recruited within very different time frames, or had the very different participation/response rates, **OR** there is insufficient information provided about the comparison group including a different rate of non-response without an explanation (record "NR" as basis for answer).
**Definitely High risk of bias:**<br>For Cohort and Cross-Sectional	There is direct evidence that subjects (both exposed and non-exposed) were not similar, recruited within very different time frames, or had the very different participation/response rates.

Sampling Risk of Bias guide (Did selection of study participants result in appropriate comparison groups? i.e., the selection of Urban and Rural population differs in the sense of resulting in different characteristics distribution affecting their HTN prevalence)

**Risk**	**Sampling description**	**Comments**
Definitely Low risk of bias	Direct evidence that the target population in Urban and Rural populations was actually sampled.	Data showing the analyzed population is representative of the target, and that there is no difference between Urban and Rural sampling
Probably Low risk of bias	The use of traditional/validated sampling strategies or a sampling that cover the entire area/village population and/or is a representative sample of target population.	Following the usual sampling strategies would be in this category. Minor differences of response rate between Urban and Rural areas are allowed to accommodate the sampling.
Probably High risk of bias	Noticeable differential response rate, not usual sampling strategies, included walk-in patients in health clinics.	Despite sampling, important differences on the response rate between Urban and Rural (e.g., 85% in Urban and 50% in Rural), or if data shows that factors driving these differences are also likely related to hypertension prevalence.
		Some studies did not use a census list or household list, but were based on clinics. These were included when the clinics catchment area was representative of the sampled area (e.g., the unique health clinics in the city). Papers with hospital cases were already excluded.
		In some papers, the recruitment was done on radio and TV, in rural, based on church announcements. This could be an indirect evidence of differential sampling between Urban and Rural. Or, if Not reported.
Definitely High risk of bias	Proof that the target population in Urban and Rural populations was differently sampled between urban and rural.	Data showing the analyzed population is not representative of Urban and Rural populations.<br>Note: this category of risk of bias is not expected, because studies with “definitely high risk of bias” were excluded for not fulfilling the inclusion criteria during full text selection.

Note: we did not evaluate the sex difference between responders at this stage, i.e., if study overrepresented females compared to males, we did not automatically assemble high risk of bias

Exposure Risk of Bias guide (Detection bias refers to systematic differences between experimental/control groups with regards to how outcomes and exposures are assesse and also considers validity and reliability of methods used to assess outcomes/exposures).

Risk	Exposure measurement	Comments
**Definitely Low risk of bias**	Development of a score to define urbanicity	Use of a validated score to define urban and rural.
**Probably Low risk of bias**	Urban and rural definitions that contrast urban and rural realities for that country.	Includes National Census definition, any objective metric, any well-defined contrast between Urban and Rural.<br><br>1. Clearly stated use of national sampling lists or derived from them (e.g., national census, electoral lists, local government areas (LGA's), enumeration areas or other national representations).<br>2. Objective measure such as distance, population density etc.<br>3. Clearly stated the domains and variables for defining urban and rural (not necessarily needed the model or weights for how the variables were used). E.g. patterns of major chronic disease rates and risk exposures, levels of economic development, relative stability of the population and local infrastructure etc.<br>4. Use of global scale definitions, such as from world bank definitions.<br>5. Mix of any of the above listed.
**Probably High risk of bias**	Not well-defined contrast between Urban and Rural, potential for certain degree of misclassification for that country.	Urban and rural definitions based on ethnic, work-related groups if these groups are not clearly urban and rural populations.<br>Or not reported.<br><br>1. If no information is provided or not much information is provided like mentioning only urban and rural, leading to concerns if the used variables/domains for urban and rural areas selection created urban-rural contrast for that country.<br>2. Urban and rural definition based exclusive on ethnic or specific work-related groups.
**Definitely High risk of bias**	There was no differentiation between Rural and Urban areas.	These studies were excluded during full-text selection, such as those evaluating semi-urban or peri-urban areas.

Outcome Risk of Bias guide (Detection bias refers to systematic differences between experimental/control groups with regards to how outcomes and exposures are assessed and also considers validity and reliability of methods used to assess outcomes/exposures).

Risk	Outcome measurement	Comments
Definitely Low risk of bias	Direct measurement of reliability, etc.	Report of agreement between measures, coefficient of variation, intracluster correlation coefficient (ICC), etc.
Probably Low risk of bias	Used the usual protocols for blood pressure measurement, trained staff etc.	Follow the minimum recommendation from the society guidelines: some resting before measurement, adequate cuff, avoid of caffeine alcohol, calibrated device, mention to trained staff, etc.
Probably High risk of bias	Not reported or vague mentions.	Some cohorts/surveys did not mention the blood pressure measurement protocol. Please, also check the original protocol/references.
		Not reported
		Measured SBP/DBP only on those without previous hypertension diagnosis/self-reported.
		There are differences in the blood pressure protocol measurement between Urban and Rural.
		Hypertension defined from electronic health records or chart review.
Definitely High risk of bias	Inclusion of only self-reported hypertension; direct evidence of biased blood pressure measurement.	These studies were excluded during full-text selection, such as those reporting only self-reported hypertension diagnosis.

e-Methods: Additional sources of data

Data	Source	Comment	Date exported/downloaded
LMIC countries list	World bank	https://datahelpdesk.worldbank.org/knowledgebase/articles/906519-world-bank-country-and-lending-groups	May 2018
Historical countries income classification	World Bank	http://databank.worldbank.org/data/download/site-content/OGHIST.xls	14/10/2020
World map	World bank	Shapefile: World Country Polygons - Very High Definition https://development-data-hub-s3-public.s3.amazonaws.com/ddhfiles/779551/wb_countries_admin0_10m.zip	03/11/2020
LMIC general data	World bank	General country data, infant mortality rate, GNI per capita, Atlas method (current US$), urban population.<br>Download by the R package *wdi*	10/06/2022
Human development index (HDI)	United Nations, United Nations Development Programme (UNDP)	**Definition**: A composite index measuring average achievement in three basic dimensions of human development—a long and healthy life, knowledge and a decent standard of living. See Technical note 1 at http://hdr.undp.org/sites/default/files/hdr2019_technical_notes.pdf for details on how the HDI is calculated.<br>**Source**: HDRO calculations based on data from UNDESA (2019b), UNESCO Institute for Statistics (2019), United Nations Statistics Division (2019b), World Bank (2019a), Barro and Lee (2018) and IMF (2019).<br>http://hdr.undp.org/en/indicators/137506	10/06/2022

e-Methods: Data Preparation and Statistical analysis

The unit of analyses was based on individual surveys. We extracted standard errors for the prevalence of hypertension following the hierarchy 1) standard error per se when provided, 2) lower and upper limit values from confidence intervals,[2] and 3) square root of ([hypertension prevalence×(1–hypertension prevalence)]/sample size).[3] When the study provided only percentages for a category (e.g., proportion of participants from rural area, proportion of participants with hypertension in urban areas), we transformed it to numbers, using available data.

Our primary summary measure was prevalence difference. We conducted the meta-analysis and meta-regression analyses using a random-effects model, with a restricted-maximum likelihood estimator and applying the Knapp and Hartung adjustment.[4] We prioritized age, sex and/or sampling weights adjusted prevalence estimates instead of crude ones when both were available. In the models, we used the prevalence (0 to 1) when provided, or estimated it based on the raw numbers, to improve accuracy and reduce the impact of rounding.

We estimated pooled urban-rural differences in hypertension prevalence across subsets (e.g., region, income classification) using a meta-regression model.[5] We estimated Q_e, I^2 and R^2 to evaluated residual heterogeneity in the meta-regression.[2,6] Publication bias was evaluated with Egger test. We evaluated non-linearity for continuous moderators applying restricted cubic splines and choosing the parsimonious model based on AIC, BIC and likelihood-ratio test. We adjusted all meta-regression models with the five study-level moderators that explained part of the heterogeneity when evaluating country-level moderators. We entered country-level moderators separately on each model because of high collinearity:

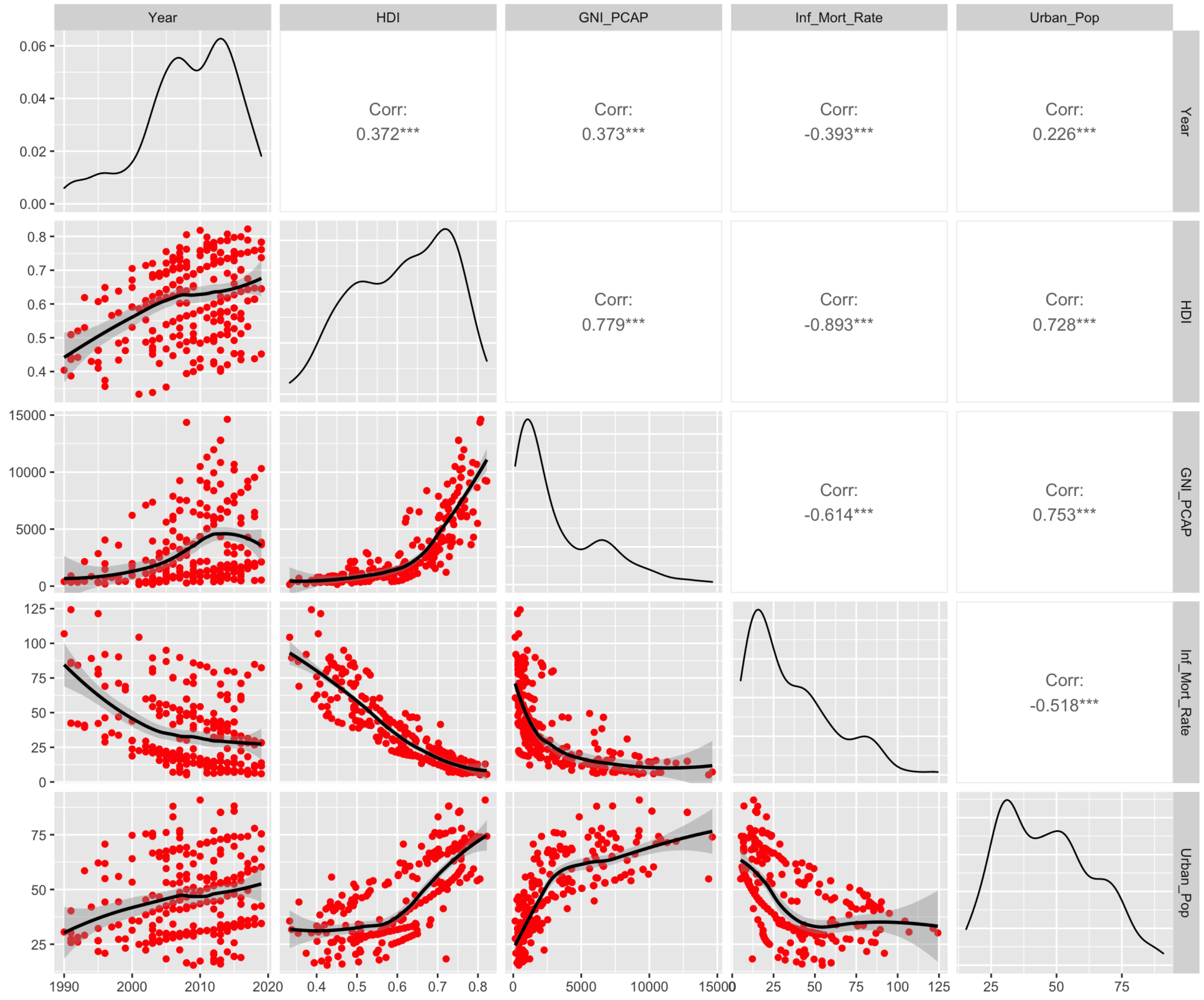

We derived the predicted urban-rural difference from each meta-regression model, setting each moderator to vary within the observed range (e.g., year of starting data collection from 1990 to 2019) and setting the five study-level characteristics to its expected least-biased category (e.g., probably low risk of bias in sampling). In a post-hoc decision to understand the main driver of the difference variation over country-level indicators, we derived the predicted hypertension prevalence from each meta-regression model for urban and rural areas following the same steps for the urban-rural difference.

All analyses were done in R, version 4.0.2, using the packages *tidyverse*,[7] *tidylog*,[8] readxl,[9] rms,[10] and *metafor*[11]. Any deviance from the pre-specified analysis was labelled as post-hoc. All statistical tests were two-sided and a P≤0.05 was considered statistically significant.

REFERENCES

1 Zhou B, Bentham J, Di Cesare M, *et al.* Worldwide trends in blood pressure from 1975 to 2015: a pooled analysis of 1479 population-based measurement studies with 19·1 million participants. *The Lancet* 2017; **389**: 37–55.

2 Cochrane Handbook for Systematic Reviews of Interventions. https://handbook-5-1.cochrane.org/ (accessed Jan 25, 2021).

3 Mills KT, Bundy JD, Kelly TN, *et al.* Global Disparities of Hypertension Prevalence and Control: A Systematic Analysis of Population-Based Studies From 90 Countries. *Circulation* 2016; **134**: 441–50.

4 Viechtbauer W, López-López JA, Sánchez-Meca J, Marín-Martínez F. A comparison of procedures to test for moderators in mixed-effects meta-regression models. *Psychol Methods* 2015; **20**: 360–74.

5 Rubio-Aparicio M, López-López JA, Viechtbauer W, Marín-Martínez F, Botella J, Sánchez-Meca J. Testing Categorical Moderators in Mixed-Effects Meta-analysis in the Presence of Heteroscedasticity. *The Journal of Experimental Education* 2020; **88**: 288–310.

6 López-López JA, Marín-Martínez F, Sánchez-Meca J, Van den Noortgate W, Viechtbauer W. Estimation of the predictive power of the model in mixed-effects meta-regression: A simulation study. *Br J Math Stat Psychol* 2014; **67**: 30–48.

7 Wickham H, Averick M, Bryan J, *et al.* Welcome to the tidyverse. *Journal of Open Source Software* 2019; **4**: 1686.

8 Elbers B. tidylog: Logging for ‘dplyr’ and ‘tidyr’ Functions. 2020 https://CRAN.R-project.org/package=tidylog.

9 Wickham H, Bryan J. readxl: Read Excel Files. 2019 https://CRAN.R-project.org/package=readxl.

10 Jr FEH. rms: Regression Modeling Strategies. 2020 https://CRAN.R-project.org/package=rms.

11 Viechtbauer W. Conducting meta-analyses in R with the metafor package. *Journal of Statistical Software* 2010; **36**: 1–48.
