# Supplementary material for: Urban-rural differences in hypertension prevalence in low-income and middle-income countries, 1990–2020: A systematic review and meta-analysis
Source: PLoS Med. 2022 Aug 25;19(8):e1004079. doi: 10.1371/journal.pmed.1004079 (PMC9410549; doi:10.1371/journal.pmed.1004079)

S1 Data

Urban-rural differences in hypertension prevalence in low-income and middle-income countries, 1990-2020: a systematic review and meta-analysis

Table of Contents

Geographical coverage of the 66 included low-income and middle-income countries 2

Number of studies and time coverage over time by country 3

Geographical coverage of the 66 included low-income and middle-income countries

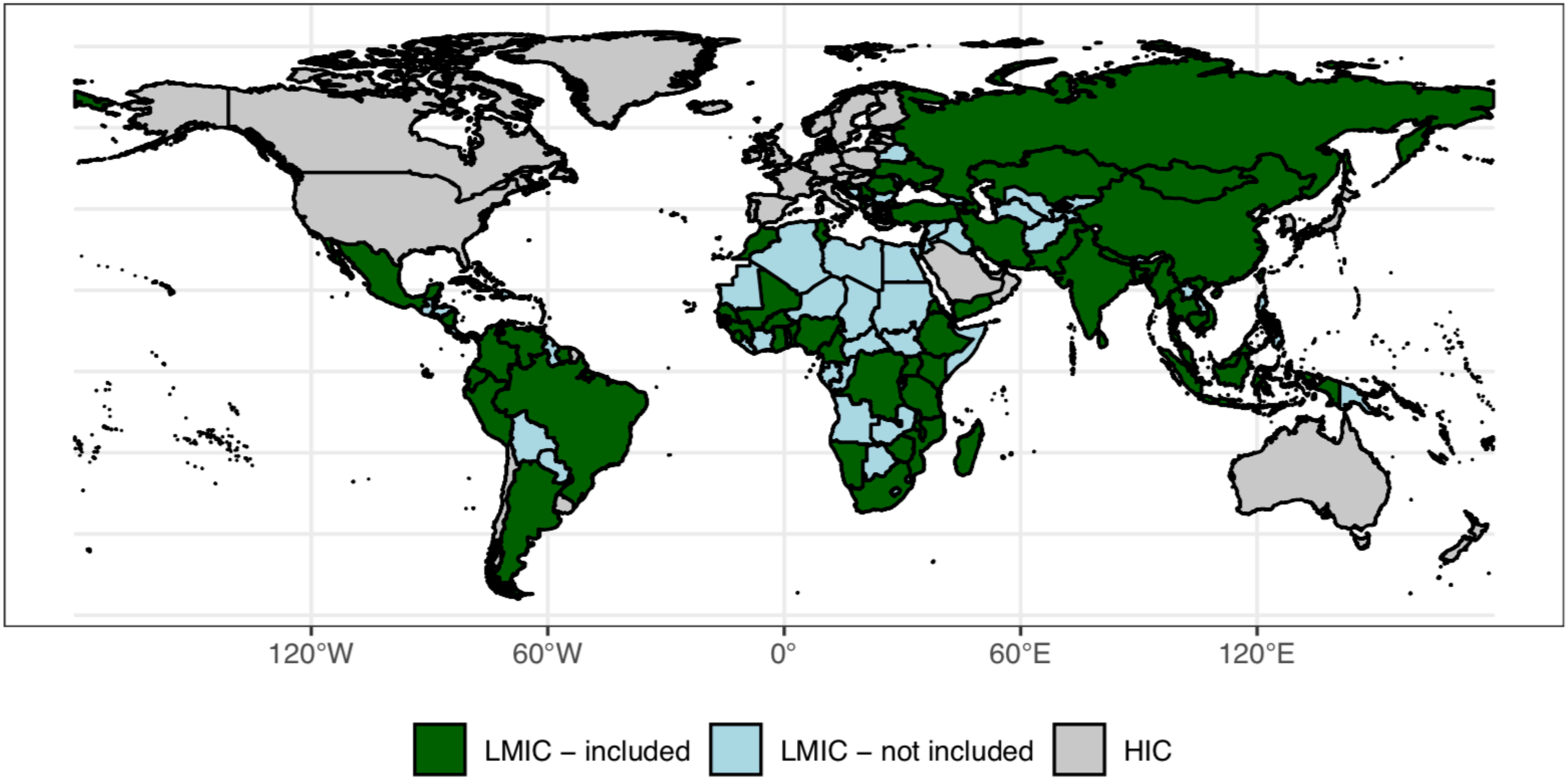

We used the World Bank definition for income status using the reference as 2018 fiscal year as well as the shapefile for the world map (https://datacatalog.worldbank.org/search/dataset/0038272, Shapefile: **World Country Polygons - Very High Definition**, File: **"wb_countries_admin0_10m.zip",** License: "**Data Access and Licensing: Classification: Public. This dataset is classified as Public under the Access to Information Classification Policy. Users inside and outside the Bank can access this dataset. License: Creative Commons Attribution 4.0"**). LMIC: low-income and middle-income country, HIC: high-income country.

Number of studies and time coverage over time by country

A – Number of studies per country

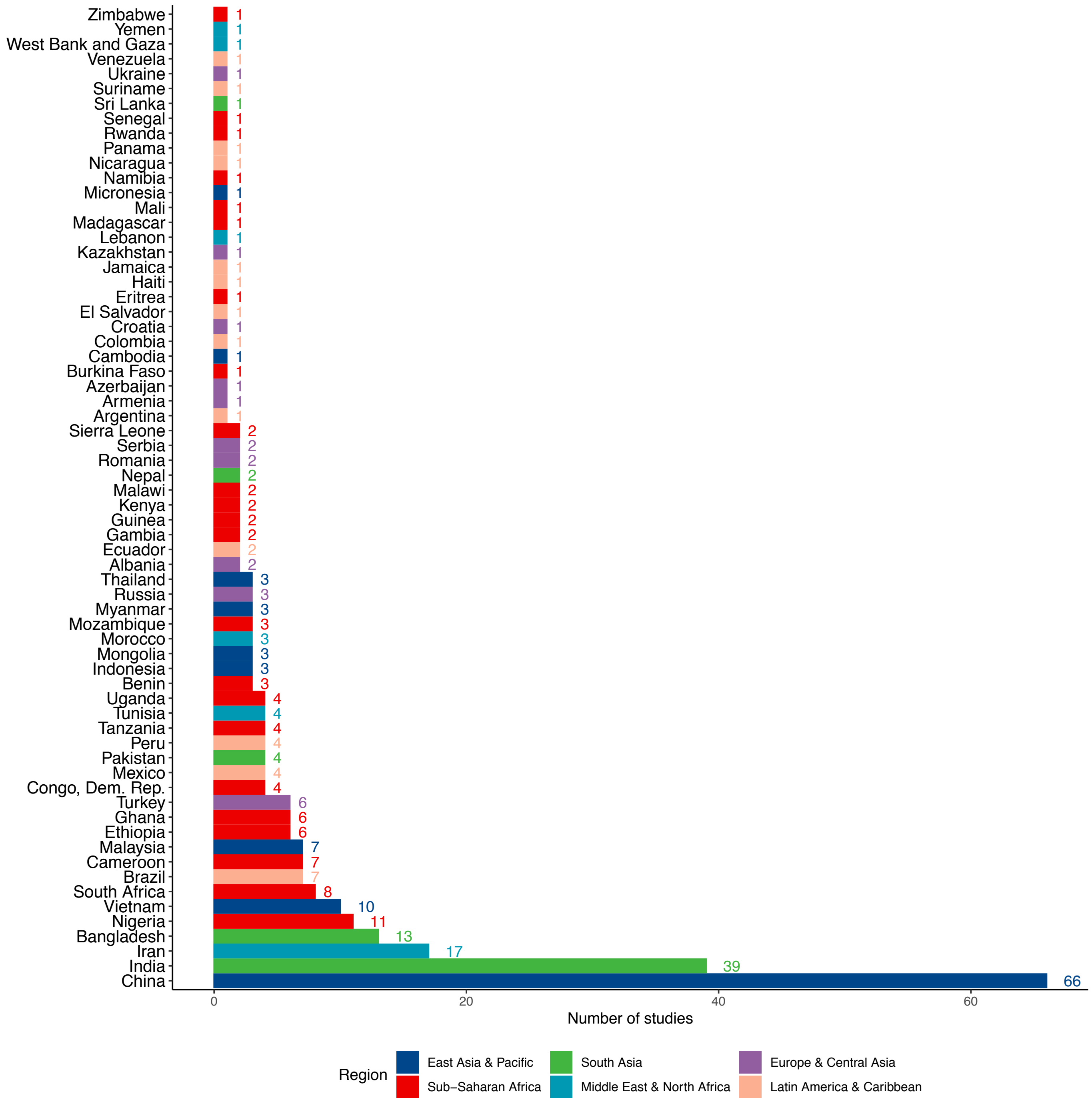

B – Period covered by studies per country (period includes year starting and ending data collection)

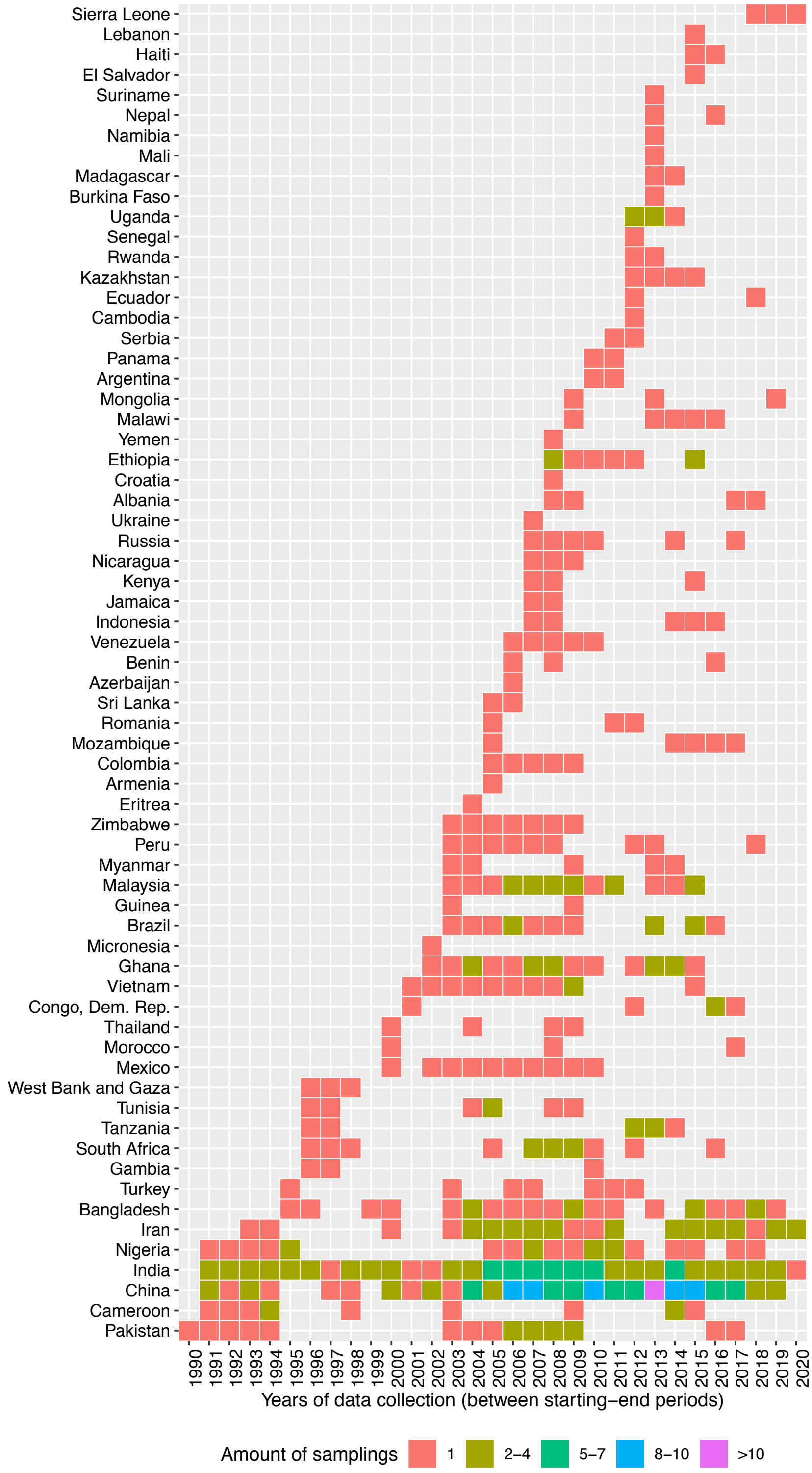

Supplement: S1 Data — (PDF) [file pmed.1004079.s003.pdf]
