# Supplementary material for: Urban-rural differences in hypertension prevalence in low-income and middle-income countries, 1990–2020: A systematic review and meta-analysis
Source: PLoS Med. 2022 Aug 25;19(8):e1004079. doi: 10.1371/journal.pmed.1004079 (PMC9410549; doi:10.1371/journal.pmed.1004079)
Supplement: S2 Data — (PDF) [file pmed.1004079.s004.pdf]

S2 Data

Urban-rural differences in hypertension prevalence in low-income and middle-income countries, 1990-2020: a systematic review and meta-analysis

Table of Contents

Characteristics of the 255 included studies arranged by country/year of publication: cohort, data from more than one wave or country, article reference 2

Characteristics of the 255 included studies arranged by country/year of publication: year of data collection, population, coverage, risk of sampling bias 31

Characteristics of the 255 included studies arranged by country/year of publication: urban/rural definition and exposure risk of bias 47

Characteristics of the 255 included studies arranged by country/year of publication: blood pressure definitions, features and outcome risk of bias 58

Sample size and demographic characteristics of the 299 surveys arranged by region and year starting collection 86

Blood pressure characteristics of the 299 surveys arranged by region and year starting collection 99

REFERENCES 115

Characteristics of the 255 included studies arranged by country/year of publication: cohort, data from more than one wave or country, article reference

First author Year publication	Country	Survey acronym	Survey Name	Data from ≥1 wave/country	Article title	Journal Name
Abba 2022[1]	Albania	DHA-2017/18	Albania Demographic Health and Survey	No	Household Air Pollution and High Blood Pressure: A Secondary Analysis of the 2016 Albania Demographic Health and Survey Dataset	Int. J. Environ. Res. Public Health
Lamelas 2019[2]	Argentina	PURE - CESCAS	The Prospective Urban Rural Epidemiology study - Cardiovascular disease and risk factors in the Southern Cone of Latin America	No	Prevalence, awareness, treatment and control of hypertension in rural and urban communities in Latin American countries	Journal of Hypertension
Sayeed 2002[3]	Bangladesh			No	Prevalence of hypertension in Bangladesh: Effect of socioeconomic risk factor on difference between rural and urban community	Bangladesh Medical Research Council Bulletin
Hussain 2005[4]	Bangladesh			No	Type 2 diabetes in rural and urban population: Diverse prevalence and associated risk factors in Bangladesh	Diabetic Medicine
Zaman 2015[5]	Bangladesh	WHO STEPS	World Health Organisation (WHO) stepwise approach to surveillance of chronic disease risk factors	No	Clustering of non-communicable diseases risk factors in Bangladeshi adults: An analysis of STEPS survey 2013	BMC public health
Biswas 2016[6]	Bangladesh	BDHS	Bangladesh Demographic and Health Survey	No	Socio-Economic Inequality of Chronic Non-Communicable Diseases in Bangladesh	PLoS ONE
Rahman 2017[7]	Bangladesh	WHO STEPS	World Health Organisation (WHO) stepwise approach to surveillance of chronic disease risk factors	No	Prevalence, treatment patterns, and risk factors of hypertension and pre-hypertension among Bangladeshi adults	Journal of Human Hypertension
Islam 2018[8]	Bangladesh		Burden of musculoskeletal disorders in Bangladesh	No	Epidemiology of hypertension among Bangladeshi adults using the 2017 ACC/AHA Hypertension Clinical Practice Guidelines and Joint National Committee 7 Guidelines	J. Hum. Hypertens.

First author Year publication	Country	Survey acronym	Survey Name	Data from ≥1 wave/country	Article title	Journal Name
Riaz 2020[9]	Bangladesh	STEPS-2018	STEPS survey for NCD risk factors in Bangladesh	No	Risk factors for non-communicable diseases in Bangladesh: Findings of the population-based cross-sectional national survey 2018	BMJ Open
Hanif 2021[10]	Bangladesh	FSNSP	Bangladesh Food Security and Nutrition Surveillance	No	Gender-specific prevalence and associated factors of hypertension among elderly Bangladeshi people: Findings from a nationally representative cross-sectional survey	BMJ Open
Hasan 2021[11]	Bangladesh	SHASTO	Strengthening Health Systems through Organizing Communities	No	Prevalence and associated factors of hypertension in selected urban and rural areas of Dhaka, Bangladesh: Findings from SHASTO baseline survey	BMJ Open
Khanam 2021[12]	Bangladesh	BDHS - 2017	Bangladesh Demographic and Health Survey	No	Prevalence and Factors of Hypertension Among Bangladeshi Adults	High Blood Press. Cardiovasc. Prev.
Paul 2021[13]	Bangladesh			No	Hypertension and its physio-psychosocial risks factors in elderly people: A cross-sectional study in north-eastern region of Bangladesh	J. Geriatr. Cardiol.
Delisle 2012[14]	Benin			No	Urbanisation, nutrition transition and cardiometabolic risk: the Benin study	The British journal of nutrition
Houehanou 2015[15]	Benin	WHO STEPS, Benin	World Health Organisation (WHO) stepwise approach to surveillance of chronic disease risk factors	No	Magnitude of cardiovascular risk factors in rural and urban areas in Benin: Findings from a nationwide steps survey	PLoS ONE
Colette 2020[16]	Benin			No	The prevalence of high blood pressure and its relationship with sociodemographic, anthropometric and lifestyles indicators: A population-based study in Aplahoue health District, Benin	Univers. J. Public. Health.

First author Year publication	Country	Survey acronym	Survey Name	Data from ≥1 wave/country	Article title	Journal Name
Almeida 2015[17]	Brazil			No	Prevalence and treatment of hypertension in urban and riverside areas in Porto Velho, the Brazilian Amazon	Postgraduate Medicine
De Paula 2015[18]	Brazil	HiperDia database	Programa Hiperdia	No	Metabolic syndrome prevalence in elderly of urban and rural communities' participants in the HIPERDIA in the city of Coimbra/MG, Brazil	Investigacion y educacion en enfermeria
Malta 2018[19]	Brazil	NHS (PNAD)	National Health Survey	No	Prevalence of arterial hypertension according to different diagnostic criteria, National Health Survey	Rev. Bras. Epidemiol.
Santiago 2019[20]	Brazil			No	Prevalence of systemic arterial hypertension and associated factors among adults from the semi-arid region of Pernambuco, Brazil	Arq. Bras. Cardiol.
deSouza 2020[21]	Brazil		State Surveys of Noncommunicable Diseases	Yes	Temporal variation in prevalence, awareness and control of hypertension in urban and rural areas in Northeast Brazil between 2006 and 2016	Cad. Saude Publica
Soubeiga 2017[22]	Burkina Faso	WHO STEPS	World Health Organisation (WHO) stepwise approach to surveillance of chronic disease risk factors	No	Prevalence and factors associated with hypertension in Burkina Faso: a countrywide cross-sectional study	BMC public health
Wagner 2018[23]	Cambodia			No	Diabetes and cardiometabolic risk factors in Cambodia: Results from two screening studies	Journal of Diabetes
Cooper 1997[24]	Cameroon	ICSHIB	International Collaborative Study of Hypertension in Blacks	No	The prevalence of hypertension in seven populations of West African origin	American Journal of Public Health
Sobngwi 2002[25]	Cameroon	ENHIP	Cameroon Essential Non communicable disease health intervention project	No	Physical activity and its relationship with obesity, hypertension and diabetes in urban and rural Cameroon	International Journal of Obesity
Fezeu 2010[26]	Cameroon			Yes	Ten-year change in blood pressure levels and prevalence of hypertension in urban and rural Cameroon	Journal of Epidemiology and Community Health

First author Year publication	Country	Survey acronym	Survey Name	Data from ≥1 wave/country	Article title	Journal Name
Lissock 2011[27]	Cameroon			No	Rural and urban differences in metabolic profiles in a Cameroonian population	Pan African Medical Journal
Kaze 2015[28]	Cameroon			No	Prevalence and determinants of chronic kidney disease in rural and urban Cameroonians: A cross-sectional study	BMC Nephrology
Lemogoum 2018[29]	Cameroon			No	Prevalence, awareness, treatment, and control of hypertension among rural and urban dwellers of the Far North Region of Cameroon	Journal of Hypertension
Tao 1995[30]	China		Third Nation-wide Survey of BP	No	Hypertension prevalence and status of awareness, treatment and control in China	Chinese Medical Journal
Reynolds 2003[31]	China	InterASIA - China	International Collaborative Study of Cardiovascular Disease in Asia	No	Geographic variations in the prevalence, awareness, treatment and control of hypertension in China	Journal of Hypertension
WangZ 2004[32]	China	China MUCA	China Multi-Center Study of Cardiovascular Epidemiology	Yes	Trends in prevalence, awareness, treatment and control of hypertension in the middle-aged population of China, 1992-1998	Hypertension Research
Wu 2008[33]	China	CNHS	China National Nutrition and Health Survey	No	Prevalence, awareness, treatment, and control of hypertension in China data from the China National Nutrition and Health Survey 2002	Circulation
Xu 2008[34]	China		The Beijing eye study	No	Prevalence of arterial hypertension in the adult population in rural and urban China: The Beijing eye study	American Journal of Hypertension
Zhang 2008[35]	China			No	Prevalence and Factors Associated With CKD: A Population Study from Beijing	American Journal of Kidney Diseases
Zuo 2009[36]	China			No	Prevalence of metabolic syndrome and factors associated with its components in Chinese adults	Metabolism: Clinical and Experimental

First author Year publication	Country	Survey acronym	Survey Name	Data from ≥1 wave/country	Article title	Journal Name
Fu 2010[37]	China			No	Prevalence of hypertension and risk factors in Heilongjiang province in 2007	Chinese Medical Journal
Zhao 2011[38]	China			No	Prevalence of metabolic syndrome in rural and urban Chinese population in Qingdao	Journal of Endocrinological Investigation
Cai 2012[39]	China			No	Prevalence, awareness, treatment, and control of hypertension among adults in Beijing, China	Clinical and Experimental Hypertension
Zheng 2012[40]	China			No	Prevalence, self-awareness, treatment, and control of hypertension in Lhasa, Tibet	Clinical and Experimental Hypertension
Gao 2013[41]	China		China National Diabetes and Metabolic Disorders Study	No	Prevalence of Hypertension in China: A Cross-Sectional Study	PLoS ONE
Lao 2013[42]	China		The Guangdong Health Survey	Yes	Hypertension prevalence, awareness, treatment, control and associated factors in a developing southern Chinese population: analysis of serial cross-sectional health survey data 2002-2010	American Journal of Hypertension
WangH 2013[43]	China			No	Factors Associated with Prevalence, Awareness, Treatment and Control of Hypertension among Adults in Southern China: A Community-Based, Cross-Sectional Survey	PLoS ONE
Xu 2013[44]	China	GPCDRFS	Guangdong Provincial Chronic Disease Risk Factor Surveillance	Yes	Prevalence, awareness, treatment, and control of hypertension among residents in Guangdong Province, China, 2004 to 2007	Circulation: Cardiovascular Quality and Outcomes

First author Year publication	Country	Survey acronym	Survey Name	Data from ≥1 wave/country	Article title	Journal Name
Bi 2014[45]	China	SMASH	Shandong-Ministry of Health Action on Salt Reduction and Hypertension	No	Hypertension prevalence, awareness, treatment, and control and sodium intake in Shandong province, China: Baseline results from Shandong-ministry of health action on salt reduction and hypertension (SMASH), 2011	Preventing Chronic Disease
Fan 2014[46]	China			No	Prevalence, awareness, treatment and control of hypertension in Henan province, China	Australian Journal of Rural Health
WangJ 2014[47]	China		China National Survey of Chronic Kidney Disease Working Group	No	Prevalence, awareness, treatment, and control of hypertension in China: Results from a National Survey	American Journal of Hypertension
Attard 2015[48]	China	CHNS	China Health and Nutrition Survey	Yes	Associations between age, cohort, and urbanization with SBP and DBP in China: A population-based study across 18 years	Journal of Hypertension
Bi 2015[49]	China		China Noncommunicable Disease Surveillance	No	Status of cardiovascular health in Chinese adults	Journal of the American College of Cardiology
Ma 2015[50]	China		Beijing Longitudinal Study of Aging	Yes	Epidemiological characteristics of hypertension in the elderly in Beijing, China	PLoS ONE
Wei 2015[51]	China			No	Prevalence of hypertension and associated risk factors in Dehui City of Jilin Province in China	Journal of Human Hypertension
Hu 2016[52]	China			No	Prevalence, awareness, treatment, and control of hypertension and associated risk factors among adults in Xi'an, China a cross-sectional study	Medicine (United States)
Huang 2016[53]	China			No	Prevalence, awareness, treatment, and control of hypertension among Chinas Sichuan Tibetan population: A cross-sectional study	Clinical and Experimental Hypertension

First author Year publication	Country	Survey acronym	Survey Name	Data from ≥1 wave/country	Article title	Journal Name
Lewington 2016[54]	China	KSCDC	The Kadoorie Study of Chronic Disease in China	No	The burden of hypertension and associated risk for cardiovascular mortality in China	JAMA Internal Medicine
Li W 2016[55]	China	PURE - China	The Prospective Urban Rural Epidemiology Study	No	Hypertension prevalence, awareness, treatment, and control in 115 rural and urban communities involving 47 000 people from China	Journal of Hypertension
Wu 2016[56]	China	CPCHC	Chinese Physiological Constant and Health Condition Survey	No	Prevalence and clustering of major cardiovascular risk factors in China: A recent cross-sectional survey	Medicine (United States)
Yang 2016[57]	China			No	Prevalence, Awareness, Treatment, Control and Risk Factors Associated with Hypertension among Adults in Southern China, 2013	PLoS ONE
Zhang 2016[58]	China			No	Prevalence of hypertension in Chinese population aged over 40 and subgroup of survival stroke patients	Biomedical Research (India)
Hu 2017[59]	China			No	Prevalence and risk factors of prehypertension and hypertension in Southern China	PLoS ONE
Huang 2017[60]	China			No	Prevalence and risk factors associated with hypertension in the Chinese Qiang population	Clinical and experimental hypertension (New York, N.Y.: 1993)
Li J 2017[61]	China	CHNS	China Health and Nutrition Survey	Yes	Urban-rural disparities in hypertension prevalence, detection, and medication use among Chinese Adults from 1993 to 2011	International Journal for Equity in Health
Li Q 2017[62]	China	CSPP	The China Stroke Prevention Project	No	Prevalence of Stroke and Vascular Risk Factors in China: A Nationwide Community-based Study	Scientific Reports
Li Y 2017[63]	China	CCDRFS	China Chronic Disease and Risk Factors Surveillance	No	Burden of hypertension in China: A nationally representative survey of 174,621 adults	International Journal of Cardiology

First author Year publication	Country	Survey acronym	Survey Name	Data from ≥1 wave/country	Article title	Journal Name
Liu X 2017a[64]	China			No	The risk factors of 9-year follow-up on hypertension in middle-aged people in Tujia-Nationality settlement of China	Journal of Human Hypertension
Liu X 2017b[65]	China			No	Hypertension prevalence, awareness, treatment, control, and associated factors in Southwest China: An update	Journal of Hypertension
Lu 2017[66]	China	"PEACE - China, China PEACE Million Persons Project"	China Patient-centred Evaluative Assessment of Cardiac Events	No	Prevalence, awareness, treatment, and control of hypertension in China: data from 1.7 million adults in a population-based screening study (China PEACE Million Persons Project)	The Lancet
ZhangFL 2017[67]	China		The China National Stroke Screening Survey	No	Hypertension prevalence, awareness, treatment, and control in northeast China: a population-based cross-sectional survey	Journal of Human Hypertension
WangJ 2018[68]	China		The Chinese Major Cardiovascular Disease Prevalence Survey and Key Technology Research Implementation Plan	No	Differences in prevalence of hypertension and associated risk factors in urban and rural residents of the North-eastern region of the people's republic of China: A cross-sectional study	PLoS ONE
WangZ 2018[69]	China	CHS	The China Hypertension Survey	No	Status of Hypertension in China: Results from the China Hypertension Survey, 2012-2015	Circulation
Du 2019[70]	China			No	Prevalence, awareness, treatment and control of hypertension and sodium intake in Zhejiang Province, China: A cross-sectional survey in 2017	PLoS ONE
Wang 2019[71]	China			No	Prevalence, Awareness, Treatment, and Control and Related Factors of Hypertension in Multiethnic Agriculture, Stock-Raising, and Urban Xinjiang, Northwest China: A Cross-Sectional Screening for 47000 Adults	Int. J. Hypertens.

First author Year publication	Country	Survey acronym	Survey Name	Data from ≥1 wave/country	Article title	Journal Name
Wei 2019[72]	China		Lanxi Cohort	No	Cohort profile: The Lanxi Cohort study on obesity and obesity-related non-communicable diseases in China	BMJ Open
Xing 2019[73]	China			No	Urban-rural disparities in status of hypertension in Northeast China: A population-based study, 2017-2019	Clin. Epidemiol.
Ding 2020[74]	China	CHARLS 2011	China Health and Retirement Longitudinal Study	No	Smoking, heavy drinking, physical inactivity, and obesity among middle-aged and older adults in China: Cross-sectional findings from the baseline survey of CHARLS 2011-2012	BMC Public Health
Han 2020[75]	China	SPECT-China	Survey of Prevalence in East China of Metabolic Diseases and Risk Factors China	No	Prevalence of hyperuricaemia in an Eastern Chinese population: A cross-sectional study	BMJ Open
Li 2020[76]	China			No	Awareness of hypertension and related factors in northeastern China: a cross-sectional study	J. Hum. Hypertens.
Ma 2020[77]	China	BCDRFS2017	Beijing Chronic Disease and Risk Factors Surveillance	No	Prevalence and Related Factors of Metabolic Syndrome in Beijing, China (Year 2017)	Obes. Facts
Su 2020[78]	China		Health checking program in Xinjiang - 2019	No	Prevalence and correlation of metabolic syndrome: A cross-sectional study of nearly 10 million multi-ethnic Chinese adults	Diabetes Metab. Syndr. Obes. Targets Ther.
Li 2021[79]	China	FJES	Fujian Eye Study	No	Intraocular pressure of adults in a coastal province in southern China: the Fujian cross-sectional eye study	Ann. Palliat. Med.
Ma 2021[80]	China	CHNS 1991 - 2015	China Health and Nutrition Survey	No	Trends in hypertension prevalence, awareness, treatment and control rates among Chinese adults, 1991-2015	J Hypertens

First author Year publication	Country	Survey acronym	Survey Name	Data from ≥1 wave/country	Article title	Journal Name
Yu 2021[81]	China			No	Hypertension among Mongolian adults in China: A cross-sectional study of prevalence, awareness, treatment, control, and related factors: Hypertension among Mongolian adults in China	J. Clin. Hypertens.
Camacho 2016[82]	Colombia	PURE- Colombia	The Prospective Urban Rural Epidemiology	No	Social disparities explain differences in hypertension prevalence, detection and control in Colombia	Journal of Hypertension
Longo-Mbenza 2008[83]	Democratic Republic of the Congo			No	Screen detection and the WHO STEPwise approach to the prevalence and risk factors of arterial hypertension in Kinshasa	European Journal of Cardiovascular Prevention and Rehabilitation
Katchunga 2019[84]	Democratic Republic of the Congo		The Bukavu observational study	Yes	The trend in blood pressure and hypertension prevalence in the general population of South Kivu between 2012 and 2016: Results from two representative cross-sectional surveys - The Bukavu observational study	PLoS ONE
Masimango 2020[85]	Democratic Republic of the Congo			No	Prevalence and Risk Factors of CKD in South Kivu, Democratic Republic of Congo: A Large-Scale Population Study	Kidney Intl. Rep.
Markovic 2011 / Bergman[86]	Croatia	CRISIC-fm	Cardiovascular Risk and Intervention Study In Croatia-family medicine	No	Continental-Mediterranean and rural-urban differences in cardiovascular risk factors in Croatian population	Croatian Medical Journal
Felix 2020[87]	Ecuador	PURE-Ecuador	The Prospective Urban Rural Epidemiology study - Ecuador	No	Low levels of awareness, treatment, and control of hypertension in Andean communities of Ecuador	J. Clin. Hypertens.
Pérez-Galarza 2021[88]	Ecuador	ENSANUT-ECU	Encuesta Nacional de Salud y Nutrición 2012	No	Prevalence of overweight and metabolic syndrome, and associated sociodemographic factors among adult Ecuadorian populations: the ENSANUT-ECU study	J. Endocrinol. Invest.
Orantes-Navarro 2019[89]	El Salvador	ENECA-ELS 2015	National Survey of Chronic Noncommunicable Diseases in Adults in El Salvador	No	The chronic kidney disease epidemic in El Salvador: A cross-sectional study	MEDICC Rev.

First author Year publication	Country	Survey acronym	Survey Name	Data from ≥1 wave/country	Article title	Journal Name
Mufunda 2006[90]	Eritrea	WHO STEPS	World Health Organisation (WHO) stepwise approach to surveillance of chronic disease risk factors	No	The prevalence of hypertension and its relationship with obesity: Results from a national blood pressure survey in Eritrea	Journal of Human Hypertension
Giday 2011[91]	Ethiopia			No	Prevalence and determinants of hypertension in rural and urban areas of southern Ethiopia	Ethiopian medical journal
Muluneh AT 2012[92]	Ethiopia			No	Population based survey of chronic non-communicable diseases at Gilgel gibe field research center, southwest Ethiopia.	Ethiop J Health Sci.
Mengistu 2014[93]	Ethiopia			No	Pattern of blood pressure distribution and prevalence of hypertension and prehypertension among adults in Northern Ethiopia: Disclosing the hidden burden	BMC Cardiovascular Disorders
Abebe 2015[94]	Ethiopia			No	Prevalence and associated factors of hypertension: A cross-sectional community-based study in Northwest Ethiopia	PLoS ONE
Gebreyes 2018[95]	Ethiopia	WHO-STEP 2015	National NCDs STEPS Survey	No	Prevalence of high bloodpressure, hyperglycemia, dyslipidemia, metabolic syndrome and their determinants in Ethiopia: Evidences from the National NCDs STEPS Survey, 2015	PLoS ONE
Tesfaye 2019[96]	Ethiopia			No	Prevalence and associated factors of hypertension in Amhara regional state city and its	Afr. Health Sci.
VanDerSande 2000[97]	Gambia			No	Blood pressure patterns and cardiovascular risk factors in rural and urban Gambian communities	Journal of Human Hypertension

First author Year publication	Country	Survey acronym	Survey Name	Data from ≥1 wave/country	Article title	Journal Name
Cham 2018[98]	Gambia	WHO STEPS, Gambia	World Health Organisation (WHO) stepwise approach to surveillance of chronic disease risk factors, Gambia	No	Burden of hypertension in The Gambia: evidence from a national World Health Organization (WHO) STEP survey	International journal of epidemiology
Agyemang 2006[99]	Ghana			No	Rural and urban differences in blood pressure and hypertension in Ghana, West Africa	Public Health
Obirikorang 2015[100]	Ghana			No	Obesity and cardio-metabolic risk factors in an urban and rural population in the Ashanti region-Ghana: A comparative cross-sectional study	PLoS ONE
Kodaman 2016[101]	Ghana			No	Cardiovascular disease risk factors in Ghana during the rural-to-urban transition: A cross-sectional study	PLoS ONE
Agyemang 2018[102]	Ghana	RODAM	Research on Obesity and Diabetes among African Migrants	No	Variations in hypertension awareness, treatment, and control among Ghanaian migrants living in Amsterdam, Berlin, London, and non-migrant Ghanaians living in rural and urban Ghana-the RODAM study	Journal of Hypertension
Sanuade 2018[103]	Ghana	GDHS-2014	Ghana Demographics and Health Survey	No	Hypertension prevalence, awareness, treatment and control in Ghanaian population: Evidence from the Ghana demographic and health survey	PLOS ONE
Baldé 2007[104]	Guinea			No	Diabetes and impaired fasting glucose in rural and urban populations in Futa Jallon (Guinea): prevalence and associated risk factors	Diabetes and Metabolism
Camara 2016[105]	Guinea	WHO STEPS, Guinea	World Health Organisation (WHO) stepwise approach to surveillance of chronic disease risk factors - Guinea	No	High prevalence, low awareness, treatment and control rates of hypertension in Guinea: Results from a population-based STEPS survey	Journal of Human Hypertension

First author Year publication	Country	Survey acronym	Survey Name	Data from ≥1 wave/country	Article title	Journal Name
DeGennaroJr 2018[106]	Haiti			No	Community-based diagnosis of non-communicable diseases and their risk factors in rural and urban Haiti: a cross-sectional prevalence study	BMJ open
Singh 1997a[107]	India			No	Prevalence of coronary artery disease and coronary risk factors in rural and urban populations of north India	European Heart Journal
Kusuma 2004[108]	India			No	Prevalence of hypertension in some cross-cultural populations of Visakhapatnam district, South India	Ethnicity and Disease
Kumar 2006[109]	India			No	Urbanization and coronary heart disease: A study of urban-rural differences in northern India	Indian Heart Journal
Kusuma 2008[110]	India			No	Hypertension in Orissa, India: a cross-sectional study among some tribal, rural and urban populations	Public Health
Gupta 2009[111]	India	JHW	Jaipur Heart Watch Study	No	Hypertension epidemiology in India: Lessons from Jaipur heart watch	Current Science
Midha 2009[112]	India			No	Prevalence and determinants of hypertension in the urban and rural population of a north Indian district.	East African journal of public health
Allender 2010[113]	India		NCD risk factor surveillance survey	No	Level of urbanization and noncommunicable disease risk factors in Tamil Nadu, India	Bulletin of the World Health Organization
Thankappan 2010[114]	India			No	Risk factor profile for chronic non-communicable diseases: Results of a community-based study in Kerala, India	Indian Journal of Medical Research
Das 2011[115]	India			No	Prevalence of cardiovascular disease risk factors by habitat: A study on adult Asian Indians in West Bengal, India	Anthropologischer Anzeiger
Bharati 2012[116]	India			No	Prevalence and covariates of undiagnosed hypertension in the adult population of Puducherry, South India	Nepal Journal of Epidemiology

First author Year publication	Country	Survey acronym	Survey Name	Data from ≥1 wave/country	Article title	Journal Name
Samuel 2012[117]	India		Vellore Birth Cohort study	No	Socio-economic status and cardiovascular risk factors in rural and urban areas of Vellore, TamilNadu, South India	International Journal of Epidemiology
Bhagyalaxmi 2013[118]	India	WHO STEPS	World Health Organisation (WHO) stepwise approach to surveillance of chronic disease risk factors	No	Prevalence of risk factors of non-communicable diseases in a district of Gujarat, India	Journal of Health, Population and Nutrition
Millett 2013[119]	India	IMS	Indian Migration Study	No	Associations between Active Travel to Work and Overweight, Hypertension, and Diabetes in India: A Cross-Sectional Study	PLoS Medicine
Bhadoria 2014[120]	India			No	Prevalence of hypertension and associated cardiovascular risk factors in Central India	Journal of Family & Community Medicine
Farag 2014[121]	India	SEEK	Screening and Early Evaluation of Kidney disease	No	Burden and predictors of hypertension in India: results of SEEK (Screening and Early Evaluation of Kidney Disease) study	BMC Nephrology
Bhansali 2015[122]	India	ICMR - INDIAB	Indian Council of Medical Research - India Diabetes Study	No	Prevalence of and risk factors for hypertension in urban and rural India: The ICMR-INDIAB study	Journal of Human Hypertension
Norboo 2015[123]	India			No	Prevalence of hypertension at high altitude: Cross-sectional survey in Ladakh, Northern India 2007-2011	BMJ Open
Krishnan 2016[124]	India	CSI Kerala CRP Study		No	Prevalence of coronary artery disease and its risk factors in Kerala, South India: A community-based cross-sectional study	BMC Cardiovascular Disorders
Oommen 2016a[125]	India	WHO STEPS - Tamil version	World Health Organisation (WHO) stepwise approach to surveillance of chronic disease risk factors	No	Prevalence of risk factors for non-communicable diseases in rural & urban Tamil Nadu	Indian Journal of Medical Research
Oommen 2016b[126]	India			No	Rising trend of cardiovascular risk factors between 1991-1994 and 2010-2012: A repeat cross sectional survey in urban and rural Vellore	Indian Heart Journal

First author Year publication	Country	Survey acronym	Survey Name	Data from ≥1 wave/country	Article title	Journal Name
Bandela 2017[127]	India			No	Study of metabolic syndrome and its components among Kurnool district population of Andhra Pradesh with different ethnic backgrounds	Journal of Cardiovascular Disease Research
Kanungo 2017[128]	India			No	Patterns and predictors of undiagnosed and uncontrolled hypertension: Observations from a poor-resource setting	Journal of Human Hypertension
Prabhakaran 2017[129]	India			Yes	20-Year Trend of CVD Risk Factors: Urban and Rural National Capital Region of India	Global Heart
Tripathy 2017[130]	India	WHO STEPS	World Health Organisation (WHO) stepwise approach to surveillance of chronic disease risk factors	No	Alarmingly high prevalence of hypertension and pre-hypertension in North India-results from a large cross-sectional STEPS survey	Plos One
Geldsetzer 2018[131]	India	DLHS - 4 and AHS	District level household and facility survey & Annual Health Survey	No	Diabetes and hypertension in India a nationally representative study of 1.3 million adults	JAMA Internal Medicine
Sarma 2019[132]	India			No	Prevalence of risk factors of non-communicable diseases in Kerala, India: Results of a cross-sectional study	BMJ Open
Kokane 2020[133]	India		State level STEPS survey 2017	No	Determinants of behavioural and biological risk factors for cardiovascular diseases from state level STEPS survey (2017-19) in Madhya Pradesh	PeerJ
Mohanty 2020[134]	India		Niyantrita Madhumeha Bharata 2017	No	Trends of Hypertension and Neurological Diseases in India: A Nationwide Survey Reporting the Distribution Across Geographical Areas	Ann. Neurosci.
Kumar 2021[135]	India	NFHS-4	National Family Health Survey	No	Sex differences in prevalence and risk factors of hypertension in India: Evidence from the National Family Health Survey-4	PLoS ONE

First author Year publication	Country	Survey acronym	Survey Name	Data from ≥1 wave/country	Article title	Journal Name
Mohanty 2021[136]	India	LASI	Longitudinal Ageing Study in India	No	Awareness, treatment, and control of hypertension in adults aged 45 years and over and their spouses in India: A nationally representative cross-sectional study	PLoS Med.
Nanditha 2021[137]	India	STRiDE-I	Secular Trends in Diabetes in India	Yes	Secular trends in cardiovascular risk factors among urban and rural populations in Tamil Nadu, India	Diabetes Res. Clin. Pract.
Patel 2021[138]	India	NFHS 5 Gujarat	National Family Health Survey	No	Trend of hypertension in **G**ujarat-understanding the nfhs-4 and nfhs-5 data	Natl. J. Community. Med.
Sivanantham 2021[139]	India		Puducherry district-wide STEPS Survey	No	Profile of risk factors for Non-Communicable Diseases (NCDs) in a highly urbanized district of India: Findings from Puducherry district-wide STEPS Survey, 2019-20	PLoS ONE
Hussain 2016[140]	Indonesia	IFLS - 4	Indonesian Family Life Survey	No	Prevalence, awareness, treatment and control of hypertension in Indonesian adults aged > 40 years: Findings from the Indonesia Family Life Survey (IFLS)	PLoS ONE
Maharani 2019[141]	Indonesia	SMART health Extended study		No	Cardiovascular disease risk factor prevalence and estimated 10-year cardiovascular risk scores in Indonesia: The SMARThealth Extend study	PLoS ONE
Sujarwoto 2020[142]	Indonesia	IFLS-5 - 2014	Indonesia Family Life Survey	No	Participation in community-based health care interventions (CBHIs) and its association with hypertension awareness, control and treatment in Indonesia	PLoS ONE
SarrafZadegan 1997[143]	Iran		The Isfahan Hypertension Study	No	Blood pressure pattern in urban and rural areas in Isfahan, Iran	Journal of Human Hypertension
Janghorbani 2008[144]	Iran	INHANES	Iranian National Health and Nutrition Examination Survey	No	Nationwide survey of prevalence and risk factors of prehypertension and hypertension in Iranian adults	Journal of Hypertension

First author Year publication	Country	Survey acronym	Survey Name	Data from ≥1 wave/country	Article title	Journal Name
Azimi-Nezhad 2009a[145]	Iran			No	Anthropometric indices of obesity and the prediction of cardiovascular risk factors in an Iranian population	The Scientific World Journal
Ebrahimi 2010[146]	Iran	SURFNCD II (2006) (SECOND NATIONAL SURVEY)	Survey of Noncommunicable Disease (NCD) Risk Factors of Iran	No	Social disparities in prevalence, treatment and control of hypertension in Iran: Second National Surveillance of Risk Factors of Noncommunicable Diseases, 2006	Journal of Hypertension
Shirani 2011[147]	Iran	IHHP	Isfahan Healthy Heart Program	No	Gender differences in the prevalence of hypertension in a representative sample of Iranian population: The Isfahan healthy heart program	Acta Biomedica
Malekzadeh 2013[148]	Iran	GCS	Golestan Cohort Study	No	Prevalence, awareness and risk factors of hypertension in a large cohort of Iranian adult population	Journal of Hypertension
Esteghamati 2016[149]	Iran	SURFNCD – 2005/2007	Surveillance of Risk Factors of Non-Communicable Diseases (NCD) for Iran	Yes	Awareness, treatment and control of pre-hypertension, and hypertension among adults in Iran	Archives of Iranian Medicine
Khorrami 2017[150]	Iran	SuRFNCD - 2011	Surveillance of Risk Factors of Non-Communicable Diseases	No	Urbanization and noncommunicable disease (NCD) risk factors: WHO STEPwise Iranian NCD risk factors surveillance in 2011	Eastern Mediterranean Health Journal
Rajati 2019[151]	Iran	RaNCD - PERSIAN (Ravansar / Kermanshah)	Ravansar Non-Communicable Disease (RaNCD) cohort (PERSIAN COHORT)	No	Prevalence, awareness, treatment, and control of hypertension and their determinants: Results from the first cohort of non-communicable diseases in a Kurdish settlement	Sci. Rep.
Katibeh 2020[152]	Iran		The Yazd Eye Study	No	Hypertension and associated factors in the Islamic Republic of Iran: A population-based study	East. Mediterr. Health J.
Ahmadi 2021[153]	Iran	SCS-Persian (Chaharmahal and Bakhtiari)	Shahrekord Cohort Study (Shahrekord PERSIAN Cohort Study)	No	Non-communicable diseases in the southwest of Iran: profile and baseline data from the Shahrekord PERSIAN Cohort Study	BMC Public Health

First author Year publication	Country	Survey acronym	Survey Name	Data from ≥1 wave/country	Article title	Journal Name
Jalali 2021[154]	Iran	NMPCH-2019	National Mobilization for the Prevention and Control of HTN	No	Blood Pressure Screening Campaign in the Adult Population	HEALTH SCOPE
Naghipour 2021[155]	Iran	PGCS	Persian Guilan Cohort Study	No	Epidemiologic profile of hypertension in Northern Iranian population: The PERSIAN Guilan cohort study (PGCS)	Ann. of Global Health
Rezaianzadeh 2021[156]	Iran	Kherameh cohort - PERSIAN (Fars)	Kherameh cohort study	No	The prevalence and predictors of pre-hypertension and hypertension in Kherameh cohort study: a population based study on 10,663 persons in south of Iran	J. Hum. Hypertens.
Allameh 2022[157]	Iran			No	Prevalence and Associated Risk Factors of Hypertension for the Middle-Aged Population (30-59 Years) in Iran: A National Cross-Sectional Study.	High Blood Press. Cardiovasc. Prev.
Ferguson 2011[158]	Jamaica	JHLS - 2	The Jamaica Health and Lifestyle Survey	No	An update on the burden of cardiovascular disease risk factors in Jamaica findings from the Jamaica health and lifestyle survey 2007-2008	West Indian Medical Journal
Supiyev 2016[159]	Kazakhstan			No	Diabetes prevalence, awareness and treatment and their correlates in older persons in urban and rural population in the Astana region, Kazakhstan	Diabetes Research and Clinical Practice
Mathenge 2010[160]	Kenya			No	Urbanization, ethnicity and cardiovascular risk in a population in transition in Nakuru, Kenya: a population-based survey	BMC public health
Walekhwa 2021[161]	Kenya	WHO-Steps 2015	Kenya STEPs survey	No	Tobacco Use and Risk Factors for Hypertensive Individuals in Kenya	HEALTHCARE
Fahs 2017[162]	Lebanon			No	The Prevalence and Awareness of Cardiovascular Diseases Risk Factors among the Lebanese Population: A Prospective Study Comparing Urban to Rural Populations	Cardiology Research and Practice

First author Year publication	Country	Survey acronym	Survey Name	Data from ≥1 wave/country	Article title	Journal Name
Ratovoson 2015[163]	Madagascar	HDSS Moramanga	"Health and	No	Hypertension, a neglected disease in rural and urban areas in Moramanga, Madagascar	PLoS ONE
Msyamboza 2011[164]	Malawi	WHO STEPS	World Health Organisation (WHO) stepwise approach to surveillance of chronic disease risk factors	No	The burden of selected chronic non-communicable diseases and their risk factors in Malawi: Nationwide steps survey	PLoS ONE
Price 2018[165]	Malawi	HDSS - Lilongwe (Area-25)	Karonga Health and Demographic Surveillance Site	No	Prevalence of obesity, hypertension, and diabetes, and cascade of care in sub-Saharan Africa: a cross-sectional, population-based study in rural and urban Malawi	The Lancet Diabetes and Endocrinology
Mohamud 2012[166]	Malaysia			No	Prevalence of metabolic syndrome and its risk factors in adult Malaysians: Results of a nationwide survey	Diabetes Research and Clinical Practice
Abdul-Razak 2016[167]	Malaysia	REDISCOVER	Responding to Increasing Cardiovascular disease prevalence	No	Prevalence, awareness, treatment, control and socio demographic determinants of hypertension in Malaysian adults	BMC public health
Naing 2016[168]	Malaysia	NHMSs	National Health and Morbidity Surveys	Yes	Hypertension in Malaysia: An analysis of trends from the national surveys 1996 to 2011	Medicine (United States)
Naidu 2019[169]	Malaysia	NHMS 2015	National Health and Morbidity Survey	No	Factors associated with the severity of hypertension among Malaysian adults	PLoS ONE
Isa 2021[170]	Malaysia			No	Dietary sodium intake and its association with hypertension: A cross-sectional study in selangor, malaysia	J. Pak. Med. Assoc.
Bâ 2018[171]	Mali	WHO STEPS	World Health Organisation (WHO) stepwise approach to surveillance of chronic disease risk factors	No	Hypertension and Associated Factors in Rural and Urban Areas Mali: Data from the STEP 2013 Survey	International Journal of Hypertension
Guerrero-Romero 2000[172]	Mexico			No	Prevalence of hypertension in indigenous inhabitants of traditional communities from the north of Mexico	Journal of Human Hypertension

First author Year publication	Country	Survey acronym	Survey Name	Data from ≥1 wave/country	Article title	Journal Name
Beltrán-Sanchéz 2011[173]	Mexico	MxFLS	Mexican Family Life Survey	No	Links between childhood and adult social circumstances and obesity and hypertension in the Mexican population.	Journal of aging and health
Hosey 2014[174]	Micronesia	WHO STEPS (FSM)	World Health Organisation (WHO) stepwise approach to surveillance of chronic disease risk factors, Federated States of Micronesia	No	Association of Socioeconomic Position and Demographic Characteristics with Cardiovascular Disease Risk Factors and Healthcare Access among Adults Living in Pohnpei, Federated States of Micronesia	International journal of chronic diseases
Pengpid 2022[175]	Mongolia	Mongolia STEPS surveys 2009, 2013 and 2019	Mongolia STEPS surveys	Yes	National trends in metabolic syndrome among adults in Mongolia from three cross-sectional surveys in 2009, 2013 and 2019	Diabetes Metab. Syndr. Clin. Res. Rev.
Tazi 2003[176]	Morocco			No	Prevalence of the main cardiovascular risk factors in Morocco: Results of a National Survey, 2000	Journal of Hypertension
Ziyyat 2014[177]	Morocco			No	Epidemiology of hypertension and its relationship with type 2 diabetes and obesity in eastern Morocco	Springer Plus
Pengpid 2020[178]	Morocco	Morocco STEPS survey 2017	Morocco STEPS survey	No	Prevalence and correlates of the metabolic syndrome in a cross-sectional community-based sample of 18-100 year-olds in Morocco: Results of the first national STEPS survey in 2017	Diabetes Metab. Syndr. Clin. Res. Rev.
Damasceno 2009[179]	Mozambique	WHO STEPS, portugese version	World Health Organisation (WHO) stepwise approach to surveillance of chronic disease risk factors	No	Hypertension prevalence, awareness, treatment, and control in Mozambique: Urban/rural gap during epidemiological transition	Hypertension
Jessen 2018[180]	Mozambique	WHO STEPS, portugese version	World Health Organisation (WHO) stepwise approach to surveillance of chronic disease risk factors	No	Hypertension in Mozambique: trends between 2005 and 2015	Journal of Hypertension

First author Year publication	Country	Survey acronym	Survey Name	Data from ≥1 wave/country	Article title	Journal Name
Mika 2020[181]	Mozambique	InCoMas	Inqu	No	The prevalence of hypertension and its distribution by sociodemographic factors in Central Mozambique: a cross sectional study	BMC Public Health
Quasem 2001[182]	Multicountry			Yes	Prevalence, awareness, treatment and control of hypertension among the elderly in Bangladesh and India: A multicentre study	Bulletin of the World Health Organization
Prince 2012[183]	Multicountry	10/66 DRG	10/66 Dementia Research Group	Yes	Hypertension prevalence, awareness, treatment and control among older people in Latin America, India and China: A 10/66 cross-sectional population-based survey	Journal of Hypertension
Basu 2013[184]	Multicountry	WHO SAGE	World Health Organization Study on Global Aging and Adult Health	Yes	Social epidemiology of hypertension in middle-income countries: Determinants of prevalence, diagnosis, treatment, and control in the WHO SAGE study	Hypertension
Chow 2013[185]	Multicountry	PURE	The Prospective Urban Rural Epidemiology	Yes	Prevalence, awareness, treatment, and control of hypertension in rural and urban communities in high-, middle-, and low-income countries	JAMA - Journal of the American Medical Association
Harhay 2013[186]	Multicountry	DHS	Demographic Health Surveys	Yes	Education, household wealth and blood pressure in Albania, Armenia, Azerbaijan and Ukraine: Findings from the Demographic Health Surveys, 2005-2009	European Journal of Internal Medicine
Kavishe 2015[187]	Multicountry			Yes	High prevalence of hypertension and of risk factors for non-communicable diseases (NCDs): A population based cross-sectional survey of NCDS and HIV infection in North-western Tanzania and Southern Uganda	BMC Medicine

First author Year publication	Country	Survey acronym	Survey Name	Data from ≥1 wave/country	Article title	Journal Name
Gupta 2017[188]	Multicountry	PURE - South Asia	The Prospective Urban Rural Epidemiology study	Yes	Association of household wealth index, educational status, and social capital with hypertension awareness, treatment, and control in South Asia	American Journal of Hypertension
Bjertness 2016[189]	Myanmar	WHO STEPS	World Health Organisation (WHO) stepwise approach to surveillance of chronic disease risk factors, Myanmar	No	Prevalence and determinants of hypertension in Myanmar - a nationwide cross-sectional study	BMC public health
Htet 2017[190]	Myanmar	WHO STEPS	World Health Organisation (WHO) stepwise approach to surveillance of chronic disease risk factors	Yes	Changes in prevalence, awareness, treatment and control of hypertension from 2004 to 2014 among 25-74-year-old citizens in the Yangon Region, Myanmar	BMC public health
Craig 2018[191]	Namibia	DHS 2013	Demographic and Health Surveys	No	Prevalence and predictors of hypertension in Namibia: A national-level cross-sectional study	PLoS ONE
Aryal 2015[192]	Nepal	WHO STEPS, Nepal	World Health Organisation (WHO) stepwise approach to surveillance of chronic disease risk factors	No	The burden and determinants of non-communicable diseases risk factors in Nepal: Findings from a nationwide STEPS survey	PLoS ONE
Mehata 2018[193]	Nepal	NDHS	Nepal Demographic Health Survey	No	Prevalence, awareness, treatment and control of hypertension in Nepal: data from nationally representative population-based cross-sectional study	Journal of hypertension
Laux 2012[194]	Nicaragua			No	Prevalence of hypertension and associated risk factors in six Nicaraguan communities	Ethnicity and Disease
Ezenwaka 1997[195]	Nigeria			No	The prevalence of insulin resistance and other cardiovascular disease risk factors in healthy elderly southwestern Nigerians	Atherosclerosis
Okosun 1999[196]	Nigeria	ICSHIB	International Collaborative Study on Hypertension in Blacks	No	Abdominal Adiposity in Six Populations of West African Descent: Prevalence and Population Attributable Fraction of Hypertension	Obesity Research

First author Year publication	Country	Survey acronym	Survey Name	Data from ≥1 wave/country	Article title	Journal Name
Isezuo SA 2011[197]	Nigeria			No	Prevalence, associated factors and relationship between prehypertension and hypertension: a study of two ethnic African populations in Northern Nigeria	J Human Hypertension
Abegunde 2013[198]	Nigeria			No	Health problems and associated risk factors in selected urban and rural elderly population groups of South-West Nigeria	Annals of African Medicine
Adediran 2013[199]	Nigeria			No	Anthropometric differences among natives of Abuja living in urban and rural communities: correlations with other cardiovascular risk factors	BMC research notes
Ejim 2013[200]	Nigeria			No	Cardiovascular risk factors in middle-aged and elderly residents in South-East Nigeria: the influence of urbanization	Nigerian journal of medicine: journal of the National Association of Resident Doctors of Nigeria
Murthy 2013[201]	Nigeria		Nigerian national blindness and visual impairment survey	No	Prevalence and risk factors for hypertension and association with ethnicity in Nigeria: Results from a national survey	Cardiovascular Journal of Africa
Okpechi 2013[202]	Nigeria	WHO STEPS	World Health Organisation (WHO) stepwise approach to surveillance of chronic disease risk factors	No	Blood Pressure Gradients and Cardiovascular Risk Factors in Urban and Rural Populations in Abia State South Eastern Nigeria Using the WHO STEPwise Approach	PLoS ONE
Oguoma 2015[203]	Nigeria	PACCS - PHASE 1	Prediabetes and Cardiovascular Complication Study	No	Prevalence of cardiovascular disease risk factors among a Nigerian adult population: relationship with income level and accessibility to CVD risks screening	BMC public health
Odili 2020[204]	Nigeria	REMAH	Removing the Mask on Hypertension	No	Prevalence, awareness, treatment and control of hypertension in Nigeria: Data from a nationwide survey 2017	Glo. Heart

First author Year publication	Country	Survey acronym	Survey Name	Data from ≥1 wave/country	Article title	Journal Name
Umuerri 2020[205]	Nigeria			No	Prevalence and correlates of prehypertension and hypertension among adults in Delta State, Nigeria: A cross-sectional community-based study	Ghana Med. J.
Jafar 2003[206]	Pakistan	NHSP	National Health Survey of Pakistan	No	Ethnic subgroup differences in hypertension in Pakistan	Journal of Hypertension
Tareen 2011[207]	Pakistan	URCDS	Urban Rural Chronic Diseases Study	No	Location of residence or social class, which is the stronger determinant associated with cardiovascular risk factors among Pakistani population? A cross sectional study	Rural and remote health
Basit 2020[208]	Pakistan	NDSP	National Diabetes Survey of Pakistan	No	Prevalence and contributing risk factors for hypertension in urban and rural areas of Pakistan; a study from second National Diabetes Survey of Pakistan (NDSP) 2016	Clin. Exp. Hypertens.
McDonaldPosso 2014[209]	Panama	PREFEC	Survey on Risk Factors Associated With Cardiovascular Disease	No	High blood pressure in Panama: Prevalence, sociodemographic and biologic profile, treatment, and control (STROBE)	Medicine (United States)
Miranda 2011[210]	Peru	PERU MIGRANT - baseline	PEru's Rural to Urban MIGRANTs	No	Differences in cardiovascular risk factors in rural, urban and rural-to-urban migrants in Peru	Heart
Bernabe-Ortiz 2017b[211]	Peru	CRONICAS		No	Impact of urbanisation and altitude on the incidence of, and risk factors for, hypertension	Heart
Chambergo-Michilot 2021[212]	Peru	DHS-2018	Peruvian Demographic and Health Survey	No	Socioeconomic determinants of hypertension and prehypertension in Peru: Evidence from the peruvian demographic and health survey	PLoS ONE
Dorobantu 2010[213]	Romania	SEPHAR	Study for the Evaluation of Prevalence of Hypertension and Cardiovascular Risk in Adult Population in Romania	No	Prevalence, awareness, treatment, and control of hypertension in Romania: Results of the SEPHAR study	International Journal of Hypertension

First author Year publication	Country	Survey acronym	Survey Name	Data from ≥1 wave/country	Article title	Journal Name
Dorobantu 2012[214]	Romania	SEPHAR-II	Study for the Evaluation of Prevalence of Hypertension and Cardiovascular Risk in Adult Population in Romania	No	Profile of the Romanian hypertensive patient data from SEPHAR II study	Romanian journal of internal medicine = Revue Roumaine de m
Artyukhov 2017[215]	Russia	ESSE-RF	Epidemiological Survey of cardiovascular diseases in different regions of the Russian Federation	No	Prevalence of arterial hypertension in the Krasnoyarsk Krai (Siberia, Russia)	BMC Cardiovascular Disorders
Balanova 2019[216]	Russia	ESSE- RF-2 study		No	Prevalence, awareness, treatment and control of hypertension in Russian Federation (data of observational ESSE- RF-2 study)	Ration. Pharmacother. Cardiol.
Nahimana 2017[217]	Rwanda	WHO STEPS	World Health Organisation (WHO) stepwise approach to surveillance of chronic disease risk factors	No	A population-based national estimate of the prevalence and risk factors associated with hypertension in Rwanda: implications for prevention and control	BMC Public Health
Seck 2014b[218]	Senegal			No	Chronic kidney disease epidemiology in Northern Senegal: A cross-sectional study	Iranian Journal of Kidney Diseases
Lovic 2013[219]	Serbia	PAHIS	Prevalence of Hypertension in Serbia Study	No	Prevalence of arterial hypertension in Serbia: PAHIS study	Journal of Hypertension
Marinkovic 2014[220]	Serbia			No	Prevalence of hypertension in adults in the A Umadija District, Serbia - A cross-sectional study	Vojnosanitetski Pregled
Odland 2020[221]	Sierra Leone			No	Prevalence and access to care for cardiovascular risk factors in older people in Sierra Leone: a cross-sectional survey	BMJ Open
Geraedts 2021[222]	Sierra Leone	PRESSCO 2020	Prevalence Study on Surgical Conditions	No	Evaluating the cascade of care for hypertension in Sierra Leone	Trop. Med. Int. Health
VanRooyen 2000[223]	South Africa	THUSA	Transition and Health during Urbanisation of South Africans	No	An epidemiological study of hypertension and its determinants in a population in transition: The THUSA study	Journal of Human Hypertension

First author Year publication	Country	Survey acronym	Survey Name	Data from ≥1 wave/country	Article title	Journal Name
Van Zyl 2012[224]	South Africa	AHA-FS	Assuring Health for All in the Free State	No	Risk-factor profiles for chronic diseases of lifestyle and metabolic syndrome in an urban and rural setting in South Africa	African Journal of Primary Health Care and Family Medicine
Kandala 2013[225]	South Africa	The South African DHS	South African Demographic and Health Survey	No	Geographic variation of hypertension in sub-Saharan Africa: A case study of South Africa	American Journal of Hypertension
Dolman 2014[226]	South Africa	PURE - South Africa	The Prospective Urban and Rural Epidemiology (PURE) Study	No	The use of predefined diet quality scores in the context of CVD risk during urbanization in the South African Prospective Urban and Rural Epidemiological (PURE) study	Public Health Nutrition
Egbujie 2016[227]	South Africa	PURE- South Africa	The Prospective Urban and Rural Epidemiology (PURE) Study	No	A cross-sectional study of socioeconomic status and cardiovascular disease risk among participants in the prospective Urban Rural Epidemiological (PURE) study	South African Medical Journal
Kandala 2021[228]	South Africa	SANHANES-2012/DHS-2016	South African Demographic and Health Survey	Yes	Mapping the burden of hypertension in south africa: A comparative analysis of the national 2012 sanhanes and the 2016 demographic and health survey	Int. J. Environ. Res. Public Health
Katulanda 2014[229]	Sri Lanka	SLDCS	Sri Lanka Diabetes and Cardiovascular Study	No	The prevalence, predictors and associations of hypertension in Sri Lanka: A cross-sectional population based national survey	Clinical and Experimental Hypertension
Krishnadath 2016[230]	Suriname		Suriname Health Study	No	Ethnic differences in prevalence and risk factors for hypertension in the Suriname Health Study: A cross sectional population study	Population Health Metrics
Edwards 2000[231]	Tanzania			No	Hypertension prevalence and care in an urban and rural area of Tanzania	Journal of Hypertension
Stanifer 2016[232]	Tanzania	CKD-AFRIKA	Comprehensive Kidney Disease Assessment for Risk factors, epidemiology, Knowledge, and Attitudes Study	No	Neighbourhood clustering of non-communicable diseases: results from a community-based study in Northern Tanzania	BMC public health

First author Year publication	Country	Survey acronym	Survey Name	Data from ≥1 wave/country	Article title	Journal Name
Mosha 2017[233]	Tanzania	HDSS	The Magu Health and Demographic Sentinel Surveillance	No	Prevalence, awareness and factors associated with hypertension in North West Tanzania	Global Health Action
Suriyawongpaisal 2003[234]	Thailand	InterASIA-Thailand	The International Collaborative Study of Cardiovascular Disease in Asia	No	Cardiovascular risk factor levels in urban and rural Thailand - The International Collaborative Study of Cardiovascular Disease in Asia (InterASIA)	European Journal of Cardiovascular Prevention and Rehabilitation
Aekplakorn 2012[235]	Thailand	Thai NHES III - IV	Thai National Health Examination Survey	Yes	Changes in prevalence, awareness, treatment and control of hypertension in Thai population, 2004-2009: Thai National Health Examination Survey III-IV	Journal of Hypertension
Bouguerra 2006[236]	Tunisia	TNNS	Tunisian National Nutrition Survey	No	Prevalence of metabolic abnormalities in the Tunisian adults: A population-based study	Diabetes and Metabolism
Hammami 2011[237]	Tunisia			No	Awareness, treatment and control of hypertension among the elderly living in their home in Tunisia	BMC Cardiovascular Disorders
Aounallah-Skhiri 2012[238]	Tunisia			No	Blood pressure and associated factors in a North African adolescent population. a national cross-sectional study in Tunisia	BMC Public Health
Ben Romdhane 2012[239]	Tunisia	TAHINA	Epidemiological Transition and Health Impact in North Africa	No	Hypertension among Tunisian adults: Results of the TAHINA project	Hypertension Research
Sonmez 1999[240]	Turkey			No	The epidemiology of elevated blood pressure as an estimate for hypertension in Aydin, Turkey	Journal of Human Hypertension
Altun 2005[241]	Turkey	PatenT	Prevalence, awareness, treatment and control of hypertension in Turkey	No	Prevalence, awareness, treatment and control of hypertension in Turkey (the PatenT study) in 2003	Journal of Hypertension
Metintas 2009[242]	Turkey			No	Awareness of hypertension and other cardiovascular risk factors in rural and urban areas in Turkey	Transactions of the Royal Society of Tropical Medicine and Hygiene

First author Year publication	Country	Survey acronym	Survey Name	Data from ≥1 wave/country	Article title	Journal Name
Sengul 2016[243]	Turkey	PatenT 2	Prevalence, awareness, treatment, and control of hypertension in Turkey	No	Changes in hypertension prevalence, awareness, treatment, and control rates in Turkey from 2003 to 2012	Journal of Hypertension
Dastan 2017[244]	Turkey		Chronic Disease and Risk Factor Survey	No	Urban and rural differences in hypertension risk factors in Turkey	Anatolian Journal of Cardiology
Oğuz 2018[245]	Turkey	PURE - Turkey	The Prospective Urban Rural Epidemiology study	No	The Prospective Urban Rural Epidemiology (PURE) study: PURE Turkey	Turk Kardiyol Dern Ars
Musinguzi 2013[246]	Uganda			No	Prevalence, Awareness and Control of Hypertension in Uganda	Plos One
Guwatudde 2015[247]	Uganda			No	The epidemiology of hypertension in Uganda: Findings from the national non-communicable diseases risk factor survey	PLoS ONE
Nakibuuka 2015[248]	Uganda			No	Stroke-risk factors differ between rural and urban communities: Population survey in central Uganda	Neuroepidemiology
Nieto-Martínez 2018[249]	Venezuela	VEMSOLS	Venezuelan Metabolic Syndrome, Obesity and Lifestyle Study	No	Prevalence of cardiometabolic risk factors in three populations from Venezuela: the VEMSOLS STUDY 2006-2010	Medicas UIS
Nguyen 2012[250]	Vietnam	1. NESH, 2. HF-S, 3. DM-S, 4. NCDs, 5. HMPS	1. The national epidemiological survey on hypertension, 2. The survey on heart failure, 3. The survey on diabetes and its risk factors, 4. The survey on non-communicable disease risk factors, 5. The screening surveys at all communes from the project on hypertension management in rural communes	Yes	Time trends in blood pressure, body mass index and smoking in the Vietnamese population: A meta-analysis from multiple cross-sectional surveys	PLoS ONE
Do 2015[251]	Vietnam		National Adult Overweight Survey	No	National prevalence and associated risk factors of hypertension and prehypertension among Vietnamese adults	American Journal of Hypertension
Jensen 2018[252]	Vietnam	WHO STEPS, Vietnam	World Health Organisation (WHO) stepwise approach to surveillance of chronic disease risk factors	No	The association of estimated salt intake with blood pressure in a Viet Nam national survey	PLoS ONE

First author Year publication	Country	Survey acronym	Survey Name	Data from ≥1 wave/country	Article title	Journal Name
Hoang 2019[253]	Vietnam	WHO-STEP 2015	WHO Step	No	Patterns of Raised Blood Pressure in Vietnam: Findings from the WHO STEPS Survey 2015	Int. J. Hypertens.
Abdul-Rahim 2001[254]	West Bank and Gaza			No	The metabolic syndrome in the West Bank population: An urban-rural comparison	Diabetes Care
Modesti 2013a[255]	Yemen	HYDY	Hypertension and Diabetes in Yemen	No	Relationship between hypertension, diabetes and proteinuria in rural and urban households in Yemen	Journal of Human Hypertension

Characteristics of the 255 included studies arranged by country/year of publication: year of data collection, population, coverage, risk of sampling bias

First author Year publication	Country	Year of data collection		Brief description of included population	Country coverage	Risk of Bias - Sampli
		Start	End			
Abba 2022	Albania	2017	2018	Adults >15 years	National	Probably Low risk of bi
Lamelas 2019	Argentina	2010	2011	Adults Aged 35 - 70 years	Other	Probably Low risk of bi
Sayeed 2002	Bangladesh	1995	1996	**Inclusion:** Adults aged 20 years and above and non-pregnant women. **Exclusion:** Individuals who had fever, diarrhoea, recent trauma, oedema, cyanosis, jaundice and those taking glucocorticoids, oral contraceptives and diuretics.	Subnational	Probably Low risk of bi
Hussain 2005	Bangladesh	2004	2004	Adults Aged 20 years and above	Other	Probably Low risk of bi
Zaman 2015	Bangladesh	2013	2013	Adults aged 25 years and above	National	Probably Low risk of bi
Biswas 2016	Bangladesh	2011	2011	Adults aged 35 years and above	National	Probably Low risk of bi
Rahman 2017	Bangladesh	2009	2010	**Inclusion:** Adults aged 25 years and above **Exclusion:** Individuals who were institutionalized, including people residing in hospitals, prisons, nursing homes, etc.	National	Probably Low risk of bi
Islam 2018	Bangladesh	2015	2015	**Inclusion:** Men and women aged 18 years and above residing in rural and urban areas of Bangladesh. **Exclusion:** tourists and the institutionalized.	National	Probably Low risk of bi
Riaz 2020	Bangladesh	2017	2018	Adults aged 18 to 69 years, who were usual residents of the households for at least 6 months and stayed the night before the survey.	National	Probably Low risk of bi
Hanif 2021	Bangladesh	2018	2019	Elderly people (60 years and above)	National	Probably Low risk of bi
Hasan 2021	Bangladesh	2018	2018	Adult men and women aged >30 years residing in selected urban and rural areas of Dhaka division, Bangladesh.	Other	Probably Low risk of bi
Khanam 2021	Bangladesh	2015	2016	All the men and women aged ≥35 years residing in the selected households.	National	Probably Low risk of bi
Paul 2021	Bangladesh	2018	2018	Adults aged 50 years and over from the north-east (e.g., Sylhet District) region of Bangladesh.	Other	Probably Low risk of bi

First author<br>Year publication	Country	Year of data collection		Brief description of included population	Country coverage	Risk of Bias - Sampli
		Start	End			
Delisle 2012	Benin	2006	2006	**Inclusion:** Adults aged between 25 - 60 years. The subjects were all Beninese-born adults who had lived in the study area for at least 6 months.<br>**Exclusion:** Individuals with a prior diagnosis of hypertension, diabetes or CHD were excluded, as they might have changed their diet (and lifestyle) since the diagnosis.	Other	Probably Low risk of bi
Houehanou 2015	Benin	2008	2008	**Inclusion:** Adults aged above 24 years and below 65 years living in Benin for at least 6 months prior to the date of the survey.<br>**Exclusion:** Pregnant women and people who could not complete the survey.	National	Probably Low risk of bi
Colette 2020	Benin	2016	2016	Adults aged 25 to 64 living in the health district of Aplahoué.	Other	Probably Low risk of bi
Almeida 2015	Brazil	2013	2013	**Inclusion:** Adults aged between 35 - 80 years<br>**Exclusion:** Pregnant women and individuals with infectious diseases.	Other	Probably Low risk of bi
DePaula 2015	Brazil	2013	2013	Elderly aged 60 years and/or above, permanent residence for more than 10 years and have attended at least one clinic visit in the last six months in a unit of municipal FHS.	Other	Probably High risk of b
Malta 2018	Brazil	2013	2013	Adults 18 years and above	National	Probably Low risk of bi
Santiago 2019	Brazil	2015	2015	Male and female adults aged 20 to 59 years residing in the semi-arid region of Pernambuco.	Other	Probably Low risk of bi
deSouza 2020	Brazil	2006	2016	Adults aged 20 years or older	Other	Probably Low risk of bi
Soubeiga 2017	Burkina Faso	2013	2013	**Inclusion:** Adults aged 25 - 64 years who had been residing in the country for at least six (06) months on the day of the survey.<br>**Exclusion:** Individuals with disabilities hampering their ability to answer the questions (serious mental disorder, hearing or intellectual disability).	National	Probably Low risk of bi
Wagner 2018	Cambodia	2012	2012	Adults aged 25 and above	Subnational	Probably High risk of b
Cooper 1997	Cameroon	1991	1994	Adults aged 25 years and above	Other	Probably Low risk of bi
Sobngwi 2002	Cameroon	1998	1998	Adults aged 15 years and above	Other	Probably Low risk of bi
Fezeu 2010	Cameroon	1994	2003	**Inclusion:** Adults aged 24 - 74 years<br>**Exclusion:** Pregnant women	Other	Probably High risk of b

First author<br>Year publication	Country	Year of data collection		Brief description of included population	Country coverage	Risk of Bias - Sampli
		Start	End			
Lissock 2011	Cameroon	2009	2009	**Inclusion:** Adults aged 18 years and above.<br>**Exclusion:** Pregnant women and subjects with severe illnesses or physical handicap that could not permit anthropometric measurements.	Other	Probably High risk of b
Kaze 2015	Cameroon	2014	2014	**Inclusion:** Adults aged 20 years and above who had been living in the study setting for more than three months.<br>**Exclusion:** Individuals with serious mental or physical (limb amputation or paralysis) disability, pregnant or breastfeeding women and participants with simultaneous leucocyturia and urine nitrites.	Other	Probably Low risk of bi
Lemogoum 2018	Cameroon	2014	2015	Adults aged 18 years and above and non-pregnant women	Other	Probably Low risk of bi
Tao 1995	China	1991	1991	Adults aged 15 years and above	National	Probably Low risk of bi
Reynolds 2003	China	2000	2001	Adults aged 35 - 74 years	Subnational	Probably Low risk of bi
WangZ 2004	China	1992	1998	Adults aged 35 - 59 years	National	Probably Low risk of bi
Wu 2008	China	2002	2002	Adults aged 18 years and above who participated in the 2002 China National Nutrition and Health Survey	National	Probably Low risk of bi
Xu 2008	China	2006	2006	Adults aged 40 years and above	Other	Probably High risk of b
Zhang 2008	China	2006	2006	Adults aged 18 years and above	Other	Probably Low risk of bi
Zuo 2009	China	2002	2002	**Inclusion:** Adults aged 35 - 74 years.<br>**Exclusion:** Pregnant women, and physically or mentally disabled person unable to follow simple questions and examinations.	Other	Probably Low risk of bi
Fu 2010	China	2007	2007	Adults aged 15 years and above and being permanent residents from both rural (Lanxi) & urban area (Nangang District of Harbin) of Heilongjiang province from 01 January to 28 February in 2007.	Other	Probably High risk of b
Zhao 2011	China	2006	2006	Adults aged 35 - 74 years and living in Qingdao for at least 5 years.	Other	Probably High risk of b
Cai 2012	China	2008	2008	Adult aged 18 - 79 years	Other	Probably Low risk of bi
Zheng 2012	China	2010	2010	Adults aged 18 years or above	Other	Probably Low risk of bi
Gao 2013	China	2007	2008	Adults aged 20 years and above who participated in 2007 - 2008 China National Diabetes and Metabolic Disorders Study.	National	Probably Low risk of bi
Lao 2013	China	2004	2010	Adults aged 18 - 69 years	Other	Probably Low risk of bi

First author Year publication	Country	Year of data collection		Brief description of included population	Country coverage	Risk of Bias - Sampli
		Start	End			
WangH 2013	China	2010	2010	**Inclusion:** Adults aged 18 years and above<br>**Exclusion:** Individuals with stroke, dementia, schizophrenia, ill in bed, deaf and dumb, less than six months of living in the local.	Other	Probably Low risk of bi
Xu 2013	China	2004	2007	**Inclusion:** Adults aged 18 - 69 years. Only people who had lived in their current residence for 6 months or longer in the past 12 months.<br>**Exclusion:** Pregnant women or for whom no blood pressure data were available.	Other	Probably Low risk of bi
Bi 2014	China	2011	2011	**Inclusion:** Adults aged 18 - 69 years living in Shandong province.<br>**Exclusion:** Individuals who were with disability and mental disorders.	Other	Probably Low risk of bi
Fan 2014	China	2012	2012	Adults aged 15 - 74 years	Other	Probably Low risk of bi
WangJ 2014	China	2009	2010	Adults aged 18 years or above	Subnational	Probably Low risk of bi
Attard 2015	China	1991	2009	**Inclusion:** Adults aged 18 - 70 years for whom blood pressure was collected during at least one of the 1991, 1993, 1997, 2000, 2004, 2006, or 2009 exams.<br>**Exclusion:** Pregnant women at time of exam or missing covariates.	National	Probably Low risk of bi
Bi 2015	China	2010	2010	Adults aged 20 years or above living in their current residence for ≥ 6 months were eligible to participate.	National	Probably Low risk of bi
Ma 2015	China	2000	2007	Elderly aged 60 years or above	Other	Probably Low risk of bi
Wei 2015	China	2013	2013	Adults aged 18 years & above and permanent residents of the city.	Other	Probably Low risk of bi
Hu 2016	China	2013	2013	**Inclusion:** Adults aged 18 years or above with more than 6 months in their current residence and who were willing to participate were included in the survey.<br>**Exclusion:** Individuals with dementia, schizophrenia, serious illness, are deaf, or are dumb.	Other	Probably Low risk of bi
Huang 2016	China	2013	2014	Adults aged 15 years and above	Other	Probably Low risk of bi
Lewington 2016	China	2004	2009	Adults aged 35 - 74 years	Subnational	Probably Low risk of bi
Li W 2016	China	2005	2009	Adults aged 35 - 70 years and intended to live at their current address for a further 4 years.	Subnational	Probably Low risk of bi
Wu 2016	China	2007	2011	Adults aged 18 years and above	Subnational	Probably Low risk of bi

First author Year publication	Country	Year of data collection		Brief description of included population	Country coverage	Risk of Bias - Sampli
		Start	End			
Yang 2016	China	2013	2013	Adults aged 15 years or above	Other	Probably Low risk of bi
Zhang 2016	China	2012	2012	Adults aged 40 or above	Subnational	Probably Low risk of bi
Hu 2017	China	2013	2014	Adults aged 15 years or above and participants living in Jiangxi Province for 6 months.	Other	Probably Low risk of bi
Huang 2017	China	2012	2013	Adults aged 20 years and above	Other	Probably Low risk of bi
Li J 2017	China	1993	2011	Adults aged 18 years and above	Subnational	Probably Low risk of bi
Li Q 2017	China	2013	2013	Adults aged 40 years or above	National	Probably Low risk of bi
Li Y 2017	China	2013	2014	Adults aged 18 years and above that had stayed in the survey site at least for 6 months in the last 12 months.	National	Probably Low risk of bi
Liu X 2017a	China	2005	2014	Adults aged 45 - 59 years	Other	Probably High risk of b
Liu X 2017b	China	2012	2013	Adults aged 18 years and above	Other	Probably Low risk of bi
Lu 2017	China	2014	2017	Adults aged 35 - 75 years	National	Probably Low risk of bi
ZhangFL 2017	China	2016	2016	**Inclusion:** Adults aged 40 years and above who had lived in Dehui City of Jilin province for more than 6 months. **Exclusion:** Individuals who were unwilling to participate in the survey or judged to be very frail.	Other	Probably Low risk of bi
WangJ 2018	China	2014	2015	Adults aged 15 years or above	Other	Probably Low risk of bi
WangZ 2018	China	2012	2015	Adults aged 18 years or above	National	Probably Low risk of bi
Du 2019	China	2016	2017	Residents aged 18 to 69 years without disability and mental disorders living in the selected areas for 6 to 12 months before the investigation.	Other	Probably Low risk of bi
Wang 2019	China	2014	2016	Residents aged ≥15 years and living for ≥ 6 months in the selected households.	Other	Probably Low risk of bi
Wei 2019	China	2015	2017	Local permanent residents aged 18 to 80 years at baseline who are physically and mentally capable of completing the questionnaire interviews and physical examination and women who are not pregnant or breastfeeding.	Other	Probably Low risk of bi
Xing 2019	China	2017	2019	**Inclusion:** All permanent residents aged 40 years and above in each village. **Exclusion:** Pregnant women or who had a mental disorder.	Other	Probably Low risk of bi

First author<br>Year publication	Country	Year of data collection		Brief description of included population	Country coverage	Risk of Bias - Sampli
		Start	End			
Ding 2020	China	2011	2012	Chinese adults aged ≥45 years	National	Probably Low risk of bi
Han 2020	China	2014	2015	**Inclusion:** Local residents more than 18 years and lived in their current area for more than 6 months were included in this study. **Exclusion:** Subjects with severe communication problems, acute illness or an unwillingness to participate.	Subnational	Probably Low risk of bi
Li 2020	China	2014	2015	General population aged ≥15 years was selected in the Jilin province.	Other	Probably Low risk of bi
Ma 2020	China	2017	2017	Beijing residents aged 18-79 years. Only persons who had lived in their current residence for ≥6 months in the past 12 months were eligible to participate.	Other	Probably Low risk of bi
Su 2020	China	2019	2019	Adults aged 18 years and above	Other	Probably Low risk of bi
Li 2021	China	2018	2019	Fujian residents aged 50 years or above	Other	Probably Low risk of bi
Ma 2021	China	2015	2015	Adults aged 20-79 years from the CHNS conducted between 1991 and 2015 were included in the study.	National	Probably Low risk of bi
Yu 2021	China	2018	2019	Mongolian adults aged ≥18 years living in China	Subnational	Probably Low risk of bi
Camacho 2016	Colombia	2005	2009	Adults aged 35 - 70 years	Subnational	Probably Low risk of bi
Longo-Mbenza 2008	Democratic Republic of the Congo	2001	2001	Adults aged 15 years and above	Other	Probably Low risk of bi
Katchunga 2019	Democratic Republic of the Congo	2012	2016	The subjects were 18 years and above	Other	Probably Low risk of bi
Masimango 2020	Democratic Republic of the Congo	2016	2017	Individuals aged 18 years and above	Other	Probably Low risk of bi
Markovic 2011 / Bergman	Croatia	2008	2008	**Inclusion:** Adults aged 40 years or above.<br>**Exclusion:** Individuals with communication disability (dysphasia, aphasia), severe dementia, and non-cardiac disease with estimated life expectancy of less than six months.	National	Probably Low risk of bi
Felix 2020	Ecuador	2018	2018	Individuals aged 35-70 years included in the Ecuadorian cohort of the PURE study.	Other	Probably Low risk of bi
Pérez-Galarza 2021	Ecuador	2012	2012	Participants aged 18-59 years with complete information on sociodemographic and biochemistry data.	National	Probably Low risk of bi

First author<br>Year publication	Country	Year of data collection		Brief description of included population	Country coverage	Risk of Bias - Sampli
		Start	End			
Orantes-Navarro 2019	El Salvador	2015	2015	Adults aged ≥20 years, residing in each selected household and providing written informed consent to participate in the study.	National	Probably Low risk of bi
Mufunda 2006	Eritrea	2004	2004	Adults aged 15 - 64 years	National	Probably Low risk of bi
Giday 2011	Ethiopia	2008	2008	**Inclusion:** Adults aged 18 and above<br>**Exclusion:** Pregnant women	Other	Probably High risk of b
Muluneh AT 2012	Ethiopia	2008	2009	Adults aged 15 - 64 years	Other	Probably Low risk of bi
Mengistu 2014	Ethiopia	2010	2011	Adults aged 18 years or above	Other	Probably High risk of b
Abebe 2015	Ethiopia	2012	2012	Adults aged 35 years and above	Other	Probably Low risk of bi
Gebreyes 2018	Ethiopia	2015	2015	**Inclusion:** Adults aged age 15-69 years who had lived at their current place of residence for at least six months and considered Ethiopia to be their primary place of residence regardless of their citizenship status. **Exclusion:** institutionalized-adults residing in hospitals, prisons, nursing homes, and other similar institutions or residents whose primary residences are military camps or dormitories, critically ill, mentally disabled and those with some type of physical disability that is not suitable for physical measurement.	National	Probably Low risk of bi
Tesfaye 2019	Ethiopia	2015	2015	Adults aged 18 years and above	Other	Probably Low risk of bi
VanDerSande 2000	Gambia	1996	1997	Adults aged 15 years and above	Other	Probably High risk of b
Cham 2018	Gambia	2010	2010	**Inclusion:** Adults aged 25 - 64 years.<br>**Exclusion:** Pregnant women	National	Probably Low risk of bi
Agyemang 2006	Ghana	2004	2004	Adults aged 18 years and above	Other	Probably High risk of b
Obirikorang 2015	Ghana	2013	2013	Adults aged 20 years or above	Other	Probably Low risk of bi
Kodaman 2016	Ghana	2002	2008	**Inclusion:** Adults aged 18 years or above.<br>**Exclusion:** Individuals with acute illness, and first or second-degree relation to someone already enrolled in the study.	Other	Probably High risk of b
Agyemang 2018	Ghana	2012	2015	Adults aged 25 - 70 years	Other	Probably Low risk of bi
Sanuade 2018	Ghana	2014	2014	Adults 15 - 49 years	National	Probably Low risk of bi
Balde 2007	Guinea	2003	2003	Adults aged 35 years and above	Other	Probably High risk of b
Camara 2016	Guinea	2009	2009	Adults aged 15 - 64 years	Subnational	Probably Low risk of bi

First author Year publication	Country	Year of data collection		Brief description of included population	Country coverage	Risk of Bias - Sampli
		Start	End			
DeGennaroJr 2018	Haiti	2015	2016	**Inclusion:** Adults aged 25 - 65 years<br>**Exclusion:** Individuals who were cognitively impaired and/or pregnant women.	Other	Probably Low risk of bi
Singh 1997a	India	1995	1995	Adults aged 25 - 64 years	Other	Probably Low risk of bi
Kusuma 2004	India	1995	1996	**Inclusion:** Adults aged 20 years or above, and belong to a specific endogamous group (already selected tribe/caste) inhabiting a particular environment (tribal hamlet/rural village/socially disadvantaged urban area).<br>**Exclusion:** Pregnant and lactating women, people suffering from obvious chronic diseases and individuals being treated for tuberculosis or leprosy, since these conditions and medications may alter the BP levels.	Other	Probably High risk of b
Kumar 2006	India	1995	2000	Adults aged 35 years and above	Other	Probably Low risk of bi
Kusuma 2008	India	2006	2006	No details provided, but age data suggest population of adults.	Other	Probably High risk of b
Gupta 2009	India	1992	1995	Adults aged 20 - 59 years from all the JHW studies	Other	Probably High risk of b
Midha 2009	India	2003	2004	Adults aged 20 years and above	Other	Probably Low risk of bi
Allender 2010	India	2003	2004	Adults aged 15 - 64 years and had resided in the household for at least 6 months at the time of survey.	Other	Probably Low risk of bi
Thankappan 2010	India	2005	2006	Adults aged between 15 - 64 years	Other	Probably Low risk of bi
Das 2011	India	2008	2010	**Inclusion:** Adults aged 30 years or above<br>**Exclusion:** Pregnant women, women on hormone therapy as well as individuals with known illness like ischemic heart disease (IHD), diabetes, and hypertension.	Other	Probably High risk of b
Bharati 2012	India	2009	2009	Adults aged 30 years and above	Other	Probably Low risk of bi
Samuel 2012	India	1998	2002	Adults aged 26 - 32 years	Other	Probably High risk of b
Bhagyalaxmi 2013	India	2008	2008	Adults aged 15 - 64 years	Other	Probably Low risk of bi
Millett 2013	India	2005	2007	**Inclusion:** Adults aged 18 years and above who reported being in the workforce (factory workers).<br>**Exclusion:** Individuals who reported being unemployed/did housework, had no information on their mode of transport to work, or had no information on their migration status.	Subnational	Probably High risk of b

First author Year publication	Country	Year of data collection		Brief description of included population	Country coverage	Risk of Bias - Sampli
		Start	End			
Bhadoria 2014	India	2014	2014	Adults aged 20 years and above	Other	Probably Low risk of bi
Farag 2014	India	2005	2007	Adults aged 18 years and above	Subnational	Probably High risk of b
Bhansali 2015	India	2008	2010	Adults aged 20 years and above	Subnational	Probably Low risk of bi
Norboo 2015	India	2007	2011	**Inclusion:** Adults aged 20 - 94 years<br>**Exclusion:** Individuals who were absentees, critical and terminal illness patients who cannot report to the study centre to complete the study.	Other	Probably Low risk of bi
Krishnan 2016	India	2011	2011	Adult aged 20 -79 years	Other	Probably Low risk of bi
Oommen 2016a	India	2010	2012	Adults aged 30 - 64 years	Other	Probably Low risk of bi
Oommen 2016b	India	1991	1994	Adults aged 30 - 60 years	Other	Probably Low risk of bi
Bandela 2017	India	2014	2014	**Inclusion:** Adults aged 20 - 60 years<br>**Exclusion:** Pregnant women, lactating mothers and those who refused to participate.	Other	Probably Low risk of bi
Kanungo 2017	India	2013	2014	Adult aged 18 years or above	Other	Probably Low risk of bi
Prabhakaran 2017	India	1991	2012	Adults aged 35 - 64 years	Subnational	Probably Low risk of bi
Tripathy 2017	India	2014	2015	Adults aged 18 - 69 years	Other	Probably Low risk of bi
Geldsetzer 2018	India	2012	2014	**Inclusion:** Adults aged 18 years or above.<br>**Exclusion:** Pregnant women	National	Probably Low risk of bi
Sarma 2019	India	2016	2017	Adults aged 18 - 69 years	Other	Probably Low risk of bi
Kokane 2020	India	2018	2019	Adults aged 18-69 years	Other	Probably Low risk of bi
Mohanty 2020	India	2017	2017	Adults aged 20 years and above	National	Probably Low risk of bi
Kumar 2021	India	2015	2016	Adult women aged 15-49 years and men aged 15-54 years.	National	Probably Low risk of bi
Mohanty 2021	India	2017	2018	Individuals aged 45 years and over and their spouses in all Indian states (except one)	National	Probably Low risk of bi
Nanditha 2021	India	2016	2017	Adults aged 20 years and above	Other	Probably Low risk of bi
Patel 2021	India	2019	2019	All women aged 15-49 years and men aged age 15-54 years.	Other	Probably Low risk of bi
Sivanantham 2021	India	2019	2020	Adults aged 18-69 years	Other	Probably Low risk of bi
Hussain 2016	Indonesia	2007	2008	Adults aged 40 years and above	Subnational	Probably Low risk of bi

First author Year publication	Country	Year of data collection		Brief description of included population	Country coverage	Risk of Bias - Sampli
		Start	End			
Maharani 2019	Indonesia	2016	2016	Adults aged 40 years and above in urban, semi urban and rural villages of the district of Malang.	Other	Probably Low risk of bi
Sujarwoto 2020	Indonesia	2014	2015	Adults aged 18 years and above	Subnational	Probably Low risk of bi
SarrafZadegan 1997	Iran	1993	1994	Adults aged 19 - 70 years	Other	Probably Low risk of bi
Janghorbani 2008	Iran	2004	2005	Adults aged 25 - 65 years	National	Probably Low risk of bi
Azimi-Nezhad 2009a	Iran	2004	2004	Adults aged 15 - 65 years	Other	Probably Low risk of bi
Ebrahimi 2010	Iran	2006	2006	Adults aged 15 - 64 years	National	Probably Low risk of bi
Shirani 2011	Iran	2000	2000	Adults aged 19 years or above	Other	Probably Low risk of bi
Malekzadeh 2013	Iran	2004	2008	Adults aged 40 - 75 years	Other	Probably Low risk of bi
Esteghamati 2016	Iran	2005	2007	Adults aged 25 - 64 years from 2005, 2007 and 2011 surveys, respectively.	National	Probably Low risk of bi
Khorrami 2017	Iran	2011	2011	Adults aged 20 - 70 years	National	Probably Low risk of bi
Rajati 2019	Iran	2014	2017	Permanent residents of Ravansar aged 35-65 years.	Other	Probably Low risk of bi
Katibeh 2020	Iran	2010	2011	Adult residents of Yazd aged 40-80 years	Other	Probably Low risk of bi
Ahmadi 2021	Iran	2016	2019	Adults aged between 35 and 70 years at the time of recruitment, having lived in the specified area for at least one year, having completed and signed the informed consent, and having Iranian nationality (i.e., having an Iranian birth certificate and a national identification number).	Other	Probably Low risk of bi
Jalali 2021	Iran	2019	2020	Adults people aged 30 and over	Other	Probably Low risk of bi
Naghipour 2021	Iran	2014	2017	Adults between 35-70 years	Other	Probably Low risk of bi
Rezaianzadeh 2021	Iran	2014	2017	Adults aged 40-70	Other	Probably Low risk of bi
Allameh 2022	Iran	2019	2020	Middle-aged Iranian population (30-59 years).	Subnational	Probably Low risk of bi
Ferguson 2011	Jamaica	2007	2008	Adults aged 15 - 74 years	National	Probably Low risk of bi
Supiyev 2016	Kazakhstan	2012	2015	Adults aged 50 - 75 years	Other	Probably High risk of b
Mathenge 2010	Kenya	2007	2008	Adults aged 50 years or above and resident in the cluster (i.e. living there at least 6 months per year) who had slept in the house either the night before or were planning on sleeping in the house that night.	Other	Probably Low risk of bi

First author<br>Year publication	Country	Year of data collection		Brief description of included population	Country coverage	Risk of Bias - Sampli
		Start	End			
Walekhwa 2021	Kenya	2015	2015	Adults aged 18-69 years	National	Probably Low risk of bi
Fahs 2017	Lebanon	2015	2015	**Inclusion:** Adults aged 45 years or above<br>**Exclusion:** Pregnant and lactating women, cancer patients, individuals with mental illnesses, and patients with established CVDs.	Subnational	Probably High risk of b
Ratovoson 2015	Madagascar	2013	2014	Adults aged 15 years and above	Other	Probably High risk of b
Msyamboza 2011	Malawi	2009	2009	Adults aged 25 - 64 years	National	Probably Low risk of bi
Price 2018	Malawi	2013	2016	Adults aged 18 years or above	Subnational	Probably Low risk of bi
Mohamud 2012	Malaysia	2008	2008	Adults aged 18 years or older	National	Probably Low risk of bi
Abdul-Razak 2016	Malaysia	2007	2011	Adults aged 30 years or above	Subnational	Probably High risk of b
Naidu 2019	Malaysia	2015	2015	Adults aged 18 years and above with complete socio-demographic variables and complete systolic and diastolic blood pressure measurements.	National	Probably Low risk of bi
Isa 2021	Malaysia	2013	2015	Malaysians of Malay origin between 35-75 years who had at least one blood pressure measurement taken during the interview.	Other	Probably High risk of b
Naing 2016	Malaysia	1996	2011	Adults aged 18 years and above in the 2006 and 2011 NHMS<br>Adults aged 30 years & above in the 1996 NHMS survey, and<br>Adults aged 15 years & above in the 2004 non-NHMS in Malaysia	National	Probably Low risk of bi
BA 2018	Mali	2013	2013	Adults aged 15 - 65 years	National	Probably Low risk of bi
Guerrero-Romero 2000	Mexico	2000	2000	**Inclusion:** Adults aged 35 - 64 years<br>**Exclusion:** Pregnant women	Other	Probably High risk of b
Beltran-Sanchez 2011	Mexico	2002	2002	Adults aged 20 years or above	National	Probably Low risk of bi
Hosey 2014	Micronesia	2002	2002	Adults aged 25 - 64 years	Other	Probably Low risk of bi
Pengpid 2022	Mongolia	2009	2019	2009: Adults aged 15- 64 years 2013 and 2019: Adults aged 15 - 69 years.	National	Probably Low risk of bi
Tazi 2003	Morocco	2000	2000	**Inclusion:** Adults aged 20 years and above.<br>**Exclusion:** Pregnant women and bed-ridden terminally ill people, as well as the mentally ill.	National	Probably Low risk of bi
Ziyyat 2014	Morocco	2008	2008	Adults aged 40 years or above	Other	Probably High risk of b

First author Year publication	Country	Year of data collection		Brief description of included population	Country coverage	Risk of Bias - Sampli
		Start	End			
Pengpid 2020	Morocco	2017	2017	Adults living in urban or rural areas, aged 18 and over and on the day of the survey usually living in ordinary households.	National	Probably Low risk of bi
Damasceno 2009	Mozambique	2005	2005	Adults aged 25 - 64 years	National	Probably Low risk of bi
Jessen 2018	Mozambique	2014	2015	Adults aged 15 - 64 years	National	Probably Low risk of bi
Mika 2020	Mozambique	2016	2017	Adults aged 20 years and above	Subnational	Probably Low risk of bi
Quasem 2001	Multicountry	1999	2000	Elderly aged 60 years and above	Other	Probably Low risk of bi
Prince 2012	Multicountry	2003	2006	Elderly aged 65 years and above	Other	Probably High risk of b
Basu 2013	Multicountry	2007	2010	**Inclusion:** Adults aged 18 years or above<br>**Exclusion:** Pregnant women and those that did not have complete blood pressure data or hypertension diagnostic and treatment history on interview.	National	Probably Low risk of bi
Chow 2013	Multicountry	2003	2009	Adults aged 35 - 70 years	Subnational	Probably Low risk of bi
Harhay 2013	Multicountry	2005	2009	Adults aged 15 - 49 years	National	Probably Low risk of bi
Kavishe 2015	Multicountry	2012	2013	Adults aged 18 years or above	Other	Probably Low risk of bi
Gupta 2017	Multicountry	2003	2009	Adults aged 35 - 70 years	Subnational	Probably Low risk of bi
Bjertness Exclusion criteria: 2016	Myanmar	2009	2009	**Inclusion:** Adults aged 15 - 64 years<br>**Exclusion:** Pregnant women, mentally ill & mentally retarded patients, very ill subjects, Institutionalized individuals (armed forces, hospitalized patients, prisoners) and temporary residents (<6 months).	National	Probably Low risk of bi
Htet 2017	Myanmar	2004	2014	**Inclusion:** Adults aged 25 - 74 year<br>**Exclusion:** Individuals who were too physically or mentally ill to participate, institutionalized people, military personnel, Buddhist monks and nuns.	Other	Probably Low risk of bi
Craig 2018	Namibia	2,013	2,013	Adults aged 35 - 64 years	National	Probably Low risk of bi
Aryal 2015	Nepal	2013	2013	Adults aged 15 - 69 years	National	Probably Low risk of bi
Mehata 2018	Nepal	2016	2016	Adults aged 15 years and above	National	Probably Low risk of bi
Laux 2012	Nicaragua	2007	2009	Adults aged 20 - 60 years	Subnational	Probably High risk of b
Ezenwaka 1997	Nigeria	1995	1995	Adults aged 55 years or above	Other	Probably High risk of b
Okosun	Nigeria	1991	1995	Adults aged 25 - 74 years	Other	Probably Low risk of bi

First author Year publication	Country	Year of data collection		Brief description of included population	Country coverage	Risk of Bias - Sampli
		Start	End			
Isezuo SA 2011	Nigeria	2009	2010	Adults aged 15 - 65 years	Other	Probably Low risk of bi
Abegunde 2013	Nigeria	2010	2011	Elderly aged 60 years and above	Other	Probably Low risk of bi
Adediran 2013	Nigeria	2010	2010	**Inclusion:** Adults aged 18 years and above<br>**Exclusion:** Pregnant women	Other	Probably Low risk of bi
Ejim 2013	Nigeria	2007	2008	Adults aged 40 - 70 years	Other	Probably High risk of b
Murthy 2013	Nigeria	2005	2007	Adults aged 40 years or above and being continually resident for at least the last three months.	National	Probably Low risk of bi
Okpechi 2013	Nigeria	2011	2012	Adults aged 18 years and above	Other	Probably Low risk of bi
Oguoma 2015	Nigeria	2014	2014	Adults aged 18 years or above with a verifiable contact address and residents/indigene.	Other	Probably High risk of b
Odili 2020	Nigeria	2017	2018	Nigerians aged 18 years and above	National	Probably Low risk of bi
Umuerri 2020	Nigeria	2015	2015	Adults aged 18 years and above who have lived in the study sites for at least one year.	Other	Probably Low risk of bi
Jafar 2003	Pakistan	1990	1994	Subjects aged 6 months to 110 years	National	Probably Low risk of bi
Tareen 2011	Pakistan	2006	2009	Adults aged 30 - 75 years	Other	Probably High risk of b
Basit 2020	Pakistan	2016	2017	Adults of aged 20 years and above	National	Probably Low risk of bi
McDonaldPosso 2014	Panama	2010	2011	**Inclusion:** Adults aged 18 years or above<br>**Exclusion:** Individuals with body mass index (BMI) <18.5 Kg/m2, and individuals for whom measurements of BMI, waist circumference, and BP could not be obtained.	Subnational	Probably Low risk of bi
Miranda 2011	Peru	2007	2008	**Inclusion:** Adults aged 30 years & above and permanently living in their residence<br>**Exclusion:** Pregnant women because of their transient physiological state, and those with mental disorders judged likely to impair survey completion.	Other	Probably Low risk of bi
Bernabe-Ortiz 2017b	Peru	2012	2013	Adults aged 35 years or above	Subnational	Probably Low risk of bi
Chambergo-Michilot 2021	Peru	2018	2018	**Included:** Adults aged 15 years or above **Excluded:** Individuals with missing and biologically non-plausible data in the independent and dependent variables.	National	Probably Low risk of bi
Dorobantu 2010	Romania	2005	2005	Adults aged 18 years or above	National	Probably Low risk of bi

First author Year publication	Country	Year of data collection		Brief description of included population	Country coverage	Risk of Bias - Sampli
		Start	End			
Dorobantu 2012	Romania	2011	2012	Adults aged 18 years or above	National	Probably Low risk of bi
Artyukhov 2017	Russia	2014	2014	Adults aged 25 - 64	Other	Probably High risk of b
Balanova 2019	Russia	2017	2017	Adults aged 25-64 years living in the Krasnodar region, Omsk and Ryazan regions, the Republic of Karelia.	Subnational	Probably Low risk of bi
Nahimana 2017	Rwanda	2012	2013	Adults aged 15 - 64 years	National	Probably Low risk of bi
Seck 2014b	Senegal	2012	2012	Adults aged 18 years and above, living in Saint-Louis more than 3 months.	Other	Probably Low risk of bi
Lovic 2013	Serbia	2012	2012	Adults aged 18 years and above	National	Probably High risk of b
Marinkovic 2014	Serbia	2011	2011	Adults aged 25 - 74 years	Other	Probably Low risk of bi
Odland 2020	Sierra Leone	2018	2018	Adults aged 40 years and above	Other	Probably Low risk of bi
Geraedts 2021	Sierra Leone	2019	2020	Adults aged ≥18 years	National	Probably Low risk of bi
VanRooyen 2000	South Africa	1996	1997	**Inclusion:** Adults aged 15 - 70 years<br>**Exclusion:** pregnant & lactating women, casual visitors, drunkenness and treatment for chronic diseases, for example hypertension and diabetes mellitus, mental diseases or other serious diseases.	Other	Probably High risk of b
vanZyl 2012	South Africa	2007	2009	Adults aged 25 - 64 years	Other	Probably High risk of b
Kandala 2013	South Africa	1998	1998	Adults aged 15 years and above	National	Probably Low risk of bi
Dolman 2014	South Africa	2005	2005	Adults aged 35 years and above that were non-users of any chronic medication and with no self-reported diseases.	Other	Probably Low risk of bi
Egbujie 2016	South Africa	2009	2009	Adults aged 35 - 70 years	Other	Probably Low risk of bi
Kandala 2021	South Africa	2012	2016	Adults aged 15 years and above from the SANHANES 2012 and the DHS 2016.	National	Probably Low risk of bi
Katulanda 2014	Sri Lanka	2005	2006	Adults aged 18 years or above	Subnational	Probably Low risk of bi
Krishnadath 2016	Suriname	2013	2013	Adults aged 15 - 65 years	National	Probably Low risk of bi
Edwards 2000	Tanzania	1996	1997	Adults aged 15 years or above	Other	Probably High risk of b
Stanifer 2016	Tanzania	2014	2014	**Inclusion:** Adults aged 18 years or above<br>**Exclusion:** Pregnant women	Other	Probably Low risk of bi
Mosha 2017	Tanzania	2012	2013	Adult aged 15 years or above	Other	Probably Low risk of bi

First author Year publication	Country	Year of data collection		Brief description of included population	Country coverage	Risk of Bias - Sampli
		Start	End			
Suriyawongpaisal 2003	Thailand	2000	2000	Adults aged 35 years or above	National	Probably High risk of b
Aekplakorn 2012	Thailand	2004	2009	Adults aged 15 years and above	National	Probably Low risk of bi
Bouguerra 2006	Tunisia	1996	1997	Adults aged 20 years or above who had measurements of height, body weight, waist circumference, blood pressure, fasting plasma glucose, total cholesterol and triglycerides.	National	Probably Low risk of bi
Hammami 2011	Tunisia	2008	2009	Elderly aged 65 years and above	Other	Probably Low risk of bi
Aounallah-Skhiri 2012	Tunisia	2005	2005	Adolescents aged 15 to 19 years	National	Probably Low risk of bi
BenRomdhane 2012	Tunisia	2004	2005	Adults aged 35 - 74 years	National	Probably High risk of b
Sonmez 1999	Turkey	1995	1995	**Inclusion:** Adults aged 18 years and above<br>**Exclusion:** non-residents, who had a mental disorder, severe obesity or a chronic metabolic illness such as chronic renal failure, hypothyroidism, and hyperthyroidism.	Other	Probably High risk of b
Altun 2005	Turkey	2003	2003	Adults aged 18 years and above	Subnational	Probably Low risk of bi
Metintas 2009	Turkey	2006	2007	Adults aged 40 years and above	Other	Probably Low risk of bi
Sengul 2016	Turkey	2012	2012	Adults aged 18 years and above	National	Probably Low risk of bi
Dastan 2017	Turkey	2011	2011	Adults aged 15 years and above	National	Probably Low risk of bi
Oğuz 2018	Turkey	2010	2010	**Inclusion:** Adults aged 35 - 70 years, residence in the visited household, and expected continued residence there for at least the next 4 years.<br>**Exclusion:** Individuals with presence of a severe mental disorder, severe frailty or immobility, and inadequate communication skills.	Subnational	Probably Low risk of bi
Musinguzi 2013	Uganda	2012	2012	**Inclusion:** Adults aged 15 years and above<br>**Exclusion:** Pregnant women	Other	Probably Low risk of bi
Guwatudde 2015	Uganda	2014	2014	Adults aged 18 - 69 years, who had resided in the sampled households for at least six months preceding the date of interview.	National	Probably Low risk of bi
Nakibuuka 2015	Uganda	2012	2013	Adults aged 18 years or above	Other	Probably Low risk of bi
Nieto-Martínez 2018	Venezuela	2006	2010	Adults aged 20 years and above	Subnational	Probably Low risk of bi
Nguyen 2012	Vietnam	2001	2009	Adults aged 25 - 74 years	National	Probably Low risk of bi
Do 2015	Vietnam	2005	2005	Adults aged 25 - 64 years	National	Probably Low risk of bi
Jensen 2018	Vietnam	2009	2009	Adults aged 25 - 64 years	Subnational	Probably Low risk of bi

First author<br>Year publication	Country	Year of data collection		Brief description of included population	Country coverage	Risk of Bias - Sampli
		Start	End			
Hoang 2019	Vietnam	2015	2015	Adults aged 18-69 years	National	Probably Low risk of bi
Abdul-Rahim 2001	West Bank and Gaza	1996	1998	Adults aged 30 - 65 years, residence within the borders of the survey area at least 6 months before the individual testing survey, and mental and physical ability to participate.	Other	Probably High risk of b
Modesti 2013a	Yemen	2008	2008	Adults aged 15 - 69 years	National	Probably Low risk of bi

Characteristics of the 255 included studies arranged by country/year of publication: urban/rural definition and exposure risk of bias

First author Year publication	Country	Urban and Rural definition used				Risk of bias – exposure
		Scales	Metrics	National/Official criteria (e.g., census)	Groups (e.g., ethnic, farmers)	
Abba 2022	Albania	No	No	Yes	No	Probably Low risk of bias
Lamelas 2019	Argentina	No	Yes	Yes	No	Probably Low risk of bias
Sayeed 2002	Bangladesh	No	Yes	No	Yes	Probably Low risk of bias
Hussain 2005	Bangladesh	No	Yes	No	No	Probably Low risk of bias
Zaman 2015	Bangladesh	No	No	Yes	No	Probably Low risk of bias
Biswas 2016	Bangladesh	No	No	Yes	No	Probably Low risk of bias
Rahman 2017	Bangladesh	No	No	Yes	No	Probably Low risk of bias
Islam 2018	Bangladesh	No	No	Yes	No	Probably Low risk of bias
Riaz 2020	Bangladesh	No	No	Yes	No	Probably Low risk of bias
Hanif 2021	Bangladesh	No	No	Yes	No	Probably Low risk of bias
Hasan 2021	Bangladesh	No	No	Yes	No	Probably Low risk of bias
Khanam 2021	Bangladesh	No	No	Yes	No	Probably Low risk of bias
Paul 2021	Bangladesh	No	No	No	No	Probably High risk of bias
Delisle 2012	Benin	No	Yes	No	No	Probably Low risk of bias
Houehanou 2015	Benin	No	Yes	Yes	No	Probably Low risk of bias
Colette 2020	Benin	No	No	No	No	Probably High risk of bias
Almeida 2015	Brazil	No	No	Yes	Yes	Probably Low risk of bias
DePaula 2015	Brazil	No	No	No	No	Probably High risk of bias
Malta 2018	Brazil	No	No	Yes	No	Probably Low risk of bias
Santiago 2019	Brazil	No	No	Yes	No	Probably Low risk of bias
deSouza 2020	Brazil	No	No	Yes	No	Probably Low risk of bias
Soubeiga 2017	Burkina Faso	No	No	Yes	No	Probably Low risk of bias
Wagner 2018	Cambodia	No	Yes	No	No	Probably Low risk of bias

First author Year publication	Country	Urban and Rural definition used				Risk of bias – exposure
		Scales	Metrics	National/Official criteria (e.g., census)	Groups (e.g., ethnic, farmers)	
Cooper 1997	Cameroon	No	No	No	Yes	Probably High risk of bias
Sobngwi 2002	Cameroon	No	No	No	Yes	Probably High risk of bias
Fezeu 2010	Cameroon	No	Yes	No	No	Probably Low risk of bias
Lissock 2011	Cameroon	No	No	No	No	Probably High risk of bias
Kaze 2015	Cameroon	No	No	Yes	No	Probably Low risk of bias
Lemogoum 2018	Cameroon	No	Yes	Yes	No	Probably Low risk of bias
Tao 1995	China	No	No	Yes	No	Probably Low risk of bias
Reynolds 2003	China	No	No	Yes	No	Probably Low risk of bias
WangZ 2004	China	No	No	Yes	Yes	Probably Low risk of bias
Wu 2008	China	No	Yes	Yes	No	Probably Low risk of bias
Xu 2008	China	No	Yes	No	No	Probably Low risk of bias
Zhang 2008	China	No	No	Yes	No	Probably Low risk of bias
Zuo 2009	China	No	No	No	No	Probably High risk of bias
Fu 2010	China	No	No	No	No	Probably High risk of bias
Zhao 2011	China	No	No	Yes	No	Probably Low risk of bias
Cai 2012	China	No	No	Yes	No	Probably Low risk of bias
Zheng 2012	China	No	Yes	No	Yes	Probably Low risk of bias
Gao 2013	China	No	No	Yes	No	Probably Low risk of bias
Lao 2013	China	No	No	Yes	No	Probably Low risk of bias
WangH 2013	China	No	Yes	No	No	Probably Low risk of bias
Xu 2013	China	No	No	Yes	No	Probably Low risk of bias
Bi 2014	China	No	No	Yes	No	Probably Low risk of bias
Fan 2014	China	No	No	No	No	Probably High risk of bias
WangJ 2014	China	No	No	Yes	No	Probably Low risk of bias
Attard 2015	China	Yes	No	No	No	Definitely Low risk of bias
Bi 2015	China	No	No	Yes	No	Probably Low risk of bias

First author Year publication	Country	Urban and Rural definition used				Risk of bias – exposure
		Scales	Metrics	National/Official criteria (e.g., census)	Groups (e.g., ethnic, farmers)	
Ma 2015	China	No	No	Yes	No	Probably Low risk of bias
Wei 2015	China	No	No	Yes	No	Probably Low risk of bias
Hu 2016	China	No	No	Yes	No	Probably Low risk of bias
Huang 2016	China	No	No	Yes	No	Probably Low risk of bias
Lewington 2016	China	No	Yes	No	No	Probably Low risk of bias
Li W 2016	China	No	Yes	Yes	No	Probably Low risk of bias
Wu 2016	China	No	Yes	No	No	Probably Low risk of bias
Yang 2016	China	No	No	Yes	No	Probably Low risk of bias
Zhang 2016	China	No	No	No	No	Probably High risk of bias
Hu 2017	China	No	No	Yes	No	Probably Low risk of bias
Huang 2017	China	No	No	Yes	No	Probably Low risk of bias
Li J 2017	China	No	No	Yes	Yes	Probably Low risk of bias
Li Q 2017	China	No	No	Yes	No	Probably Low risk of bias
Li Y 2017	China	No	No	Yes	No	Probably Low risk of bias
Liu X 2017a	China	No	No	No	No	Probably High risk of bias
Liu X 2017b	China	No	No	Yes	No	Probably Low risk of bias
Lu 2017	China	No	No	Yes	No	Probably Low risk of bias
ZhangFL 2017	China	No	No	Yes	No	Probably Low risk of bias
WangJ 2018	China	No	No	Yes	No	Probably Low risk of bias
WangZ 2018	China	No	No	Yes	No	Probably Low risk of bias
Du 2019	China	No	No	No	No	Probably High risk of bias
Wang 2019	China	No	No	Yes	Yes	Probably Low risk of bias
Wei 2019	China	No	No	No	No	Probably High risk of bias
Xing 2019	China	No	No	No	No	Probably High risk of bias
Ding 2020	China	No	No	Yes	No	Probably Low risk of bias
Han 2020	China	No	No	Yes	No	Probably Low risk of bias

First author Year publication	Country	Urban and Rural definition used				Risk of bias – exposure
		Scales	Metrics	National/Official criteria (e.g., census)	Groups (e.g., ethnic, farmers)	
Li 2020	China	No	No	Yes	No	Probably Low risk of bias
Ma 2020	China	No	No	Yes	No	Probably Low risk of bias
Su 2020	China	No	No	Yes	No	Probably Low risk of bias
Li 2021	China	No	No	Yes	No	Probably Low risk of bias
Ma 2021	China	No	Yes	No	No	Probably Low risk of bias
Yu 2021	China	No	No	No	Yes	Probably High risk of bias
Camacho 2016	Colombia	No	Yes	No	No	Probably Low risk of bias
Longo-Mbenza 2008	Democratic Republic of the Congo	No	Yes	Yes	No	Probably Low risk of bias
Katchunga 2019	Democratic Republic of the Congo	No	No	Yes	No	Probably Low risk of bias
Masimango 2020	Democratic Republic of the Congo	No	No	Yes	Yes	Probably Low risk of bias
Markovic 2011 / Bergman	Croatia	No	Yes	No	No	Probably Low risk of bias
Felix 2020	Ecuador	No	No	Yes	No	Probably Low risk of bias
Pérez-Galarza 2021	Ecuador	No	No	Yes	No	Probably Low risk of bias
Orantes-Navarro 2019	El Salvador	No	No	Yes	No	Probably Low risk of bias
Mufunda 2006	Eritrea	No	No	No	No	Probably High risk of bias
Giday 2011	Ethiopia	No	No	No	No	Probably High risk of bias
Muluneh AT 2012	Ethiopia	No	No	Yes	No	Probably Low risk of bias
Mengistu 2014	Ethiopia	No	Yes	No	No	Probably Low risk of bias
Abebe 2015	Ethiopia	No	No	No	No	Probably High risk of bias
Gebreyes 2018	Ethiopia	No	No	Yes	No	Probably Low risk of bias
Tesfaye 2019	Ethiopia	No	No	Yes	No	Probably Low risk of bias

First author Year publication	Country	Urban and Rural definition used				Risk of bias – exposure
		Scales	Metrics	National/Official criteria (e.g., census)	Groups (e.g., ethnic, farmers)	
VanDerSande 2000	Gambia	No	Yes	No	Yes	Probably Low risk of bias
Cham 2018	Gambia	No	No	Yes	No	Probably Low risk of bias
Agyemang 2006	Ghana	No	No	No	No	Probably High risk of bias
Obirikorang 2015	Ghana	No	Yes	No	No	Probably Low risk of bias
Kodaman 2016	Ghana	No	Yes	No	No	Probably Low risk of bias
Agyemang 2018	Ghana	No	No	Yes	No	Probably Low risk of bias
Sanuade 2018	Ghana	No	No	Yes	No	Probably Low risk of bias
Balde 2007	Guinea	No	Yes	No	No	Probably Low risk of bias
Camara 2016	Guinea	No	No	Yes	No	Probably Low risk of bias
DeGennaroJr 2018	Haiti	No	No	No	No	Probably High risk of bias
Singh 1997a	India	No	No	Yes	No	Probably Low risk of bias
Kusuma 2004	India	No	No	No	Yes	Probably High risk of bias
Kumar 2006	India	No	Yes	No	No	Probably Low risk of bias
Kusuma 2008	India	No	No	No	Yes	Probably High risk of bias
Gupta 2009	India	No	Yes	No	No	Probably Low risk of bias
Midha 2009	India	No	No	Yes	No	Probably Low risk of bias
Allender 2010	India	Yes	Yes	No	No	Definitely Low of risk of bias
Thankappan 2010	India	No	No	Yes	No	Probably Low risk of bias
Das 2011	India	No	Yes	No	No	Probably Low risk of bias
Bharati 2012	India	No	No	Yes	No	Probably Low risk of bias
Samuel 2012	India	No	Yes	No	Yes	Probably Low risk of bias
Bhagyalaxmi 2013	India	No	No	Yes	No	Probably Low risk of bias
Millett 2013	India	No	No	Yes	No	Probably Low risk of bias
Bhadoria 2014	India	No	No	No	No	Probably High risk of bias
Farag 2014	India	No	No	No	No	Probably High risk of bias
Bhansali 2015	India	No	No	Yes	No	Probably Low risk of bias

First author Year publication	Country	Urban and Rural definition used				Risk of bias – exposure
		Scales	Metrics	National/Official criteria (e.g., census)	Groups (e.g., ethnic, farmers)	
Norboo 2015	India	No	No	Yes	No	Probably Low risk of bias
Krishnan 2016	India	No	No	Yes	No	Probably Low risk of bias
Oommen 2016a	India	No	No	Yes	No	Probably Low risk of bias
Oommen 2016b	India	No	No	Yes	No	Probably Low risk of bias
Bandela 2017	India	No	No	No	No	Probably High risk of bias
Kanungo 2017	India	No	No	Yes	No	Probably Low risk of bias
Prabhakaran 2017	India	No	No	Yes	No	Probably Low risk of bias
Tripathy 2017	India	No	No	Yes	No	Probably Low risk of bias
Geldsetzer 2018	India	No	No	Yes	No	Probably Low risk of bias
Sarma 2019	India	No	No	Yes	No	Probably Low risk of bias
Kokane 2020	India	No	No	Yes	No	Probably Low risk of bias
Mohanty 2020	India	No	No	Yes	No	Probably Low risk of bias
Kumar 2021	India	No	No	Yes	No	Probably Low risk of bias
Mohanty 2021	India	No	No	Yes	No	Probably Low risk of bias
Nanditha 2021	India	No	Yes	Yes	No	Probably Low risk of bias
Patel 2021	India	No	No	Yes	No	Probably Low risk of bias
Sivanantham 2021	India	No	No	Yes	No	Probably Low risk of bias
Hussain 2016	Indonesia	No	No	No	No	Probably High risk of bias
Maharani 2019	Indonesia	No	Yes	No	No	Probably Low risk of bias
Sujarwoto 2020	Indonesia	No	No	Yes	No	Probably Low risk of bias
SarrafZadegan 1997	Iran	No	Yes	No	No	Probably Low risk of bias
Janghorbani 2008	Iran	No	No	No	No	Probably High risk of bias
Azimi-Nezhad 2009a	Iran	No	No	Yes	No	Probably Low risk of bias
Ebrahimi 2010	Iran	No	No	No	No	Probably High risk of bias
Shirani 2011	Iran	No	No	Yes	No	Probably Low risk of bias
Malekzadeh 2013	Iran	No	No	No	No	Probably High risk of bias

First author<br>Year publication	Country	Urban and Rural definition used				Risk of bias – exposure
		Scales	Metrics	National/Official criteria (e.g., census)	Groups (e.g., ethnic, farmers)	
Esteghamati 2016	Iran	No	No	No	No	Probably High risk of bias
Khorrami 2017	Iran	Yes	No	No	No	Definitely Low of risk of bias
Rajati 2019	Iran	No	No	Yes	No	Probably Low risk of bias
Katibeh 2020	Iran	No	No	No	No	Probably High risk of bias
Ahmadi 2021	Iran	No	No	Yes	No	Probably Low risk of bias
Jalali 2021	Iran	No	No	Yes	No	Probably Low risk of bias
Naghipour 2021	Iran	No	No	Yes	No	Probably Low risk of bias
Rezaianzadeh 2021	Iran	No	No	Yes	No	Probably Low risk of bias
Allameh 2022	Iran	No	No	No	No	Probably High risk of bias
Ferguson 2011	Jamaica	No	No	Yes	No	Probably Low risk of bias
Supiyev 2016	Kazakhstan	No	Yes	No	No	Probably Low risk of bias
Mathenge 2010	Kenya	No	No	Yes	No	Probably Low risk of bias
Walekhwa 2021	Kenya	No	No	Yes	No	Probably Low risk of bias
Fahs 2017	Lebanon	No	No	No	No	Probably High risk of bias
Ratovoson 2015	Madagascar	No	No	Yes	No	Probably Low risk of bias
Msyamboza 2011	Malawi	No	No	Yes	No	Probably Low risk of bias
Price 2018	Malawi	No	Yes	No	No	Probably Low risk of bias
Mohamud 2012	Malaysia	No	No	No	No	Probably High risk of bias
Abdul-Razak 2016	Malaysia	No	Yes	Yes	No	Probably Low risk of bias
Naing 2016	Malaysia	No	No	Yes	No	Probably Low risk of bias
Naidu 2019	Malaysia	No	Yes	Yes	No	Probably Low risk of bias
Isa 2021	Malaysia	No	No	No	No	Probably High risk of bias
BA 2018	Mali	No	No	No	No	Probably High risk of bias
Guerrero-Romero 2000	Mexico	No	No	No	Yes	Probably High risk of bias
Beltran-Sanchez 2011	Mexico	No	Yes	Yes	No	Probably Low risk of bias
Hosey 2014	Micronesia	No	No	Yes	No	Probably Low risk of bias

First author<br>Year publication	Country	Urban and Rural definition used				Risk of bias – exposure
		Scales	Metrics	National/Official criteria (e.g., census)	Groups (e.g., ethnic, farmers)	
Pengpid 2022	Mongolia	No	No	Yes	No	Probably Low risk of bias
Tazi 2003	Morocco	No	No	Yes	No	Probably Low risk of bias
Ziyyat 2014	Morocco	No	No	No	No	Probably High risk of bias
Pengpid 2020	Morocco	No	No	Yes	No	Probably Low risk of bias
Damasceno 2009	Mozambique	No	No	Yes	No	Probably Low risk of bias
Jessen 2018	Mozambique	No	No	Yes	No	Probably Low risk of bias
Mika 2020	Mozambique	No	No	Yes	No	Probably Low risk of bias
Quasem 2001	Multicountry	No	No	Yes	No	Probably Low risk of bias
Prince 2012	Multicountry	No	Yes	No	No	Probably Low risk of bias
Basu 2013	Multicountry	No	Yes	Yes	No	Probably Low risk of bias
Chow 2013	Multicountry	No	Yes	Yes	No	Probably Low risk of bias
Harhay 2013	Multicountry	No	Yes	No	No	Probably Low risk of bias
Kavishe 2015	Multicountry	No	No	Yes	No	Probably Low risk of bias
Gupta 2017	Multicountry	No	Yes	Yes	No	Probably Low risk of bias
Bjertness 2016	Myanmar	No	No	No	No	Probably High risk of bias
Htet 2017	Myanmar	No	No	Yes	No	Probably Low risk of bias
Craig 2018	Namibia	No	No	Yes	No	Probably High risk of bias
Aryal 2015	Nepal	No	No	Yes	No	Probably Low risk of bias
Mehata 2018	Nepal	No	No	Yes	No	Probably Low risk of bias
Laux 2012	Nicaragua	No	No	No	No	Probably High risk of bias
Ezenwaka 1997	Nigeria	No	Yes	No	Yes	Probably Low risk of bias
Okosun	Nigeria	No	No	No	No	Probably High risk of bias
Isezuo SA 2011	Nigeria	No	No	No	No	Probably High risk of bias
Abegunde 2013	Nigeria	No	Yes	No	No	Probably Low risk of bias
Adediran 2013	Nigeria	No	Yes	No	No	Probably Low risk of bias
Ejim 2013	Nigeria	No	No	Yes	No	Probably Low risk of bias

First author Year publication	Country	Urban and Rural definition used				Risk of bias – exposure
		Scales	Metrics	National/Official criteria (e.g., census)	Groups (e.g., ethnic, farmers)	
Murthy 2013	Nigeria	No	No	Yes	No	Probably Low risk of bias
Okpechi 2013	Nigeria	No	No	Yes	No	Probably Low risk of bias
Oguoma 2015	Nigeria	No	No	Yes	No	Probably Low risk of bias
Odili 2020	Nigeria	No	No	Yes	No	Probably Low risk of bias
Umuerri 2020	Nigeria	No	Yes	Yes	No	Probably Low risk of bias
Jafar 2003	Pakistan	No	No	Yes	No	Probably Low risk of bias
Tareen 2011	Pakistan	No	Yes	Yes	No	Probably Low risk of bias
Basit 2020	Pakistan	No	No	Yes	No	Probably Low risk of bias
McDonaldPosso 2014	Panama	No	No	Yes	Yes	Probably Low risk of bias
Miranda 2011	Peru	No	Yes	No	No	Probably Low risk of bias
Bernabe-Ortiz 2017b	Peru	No	Yes	No	No	Probably Low risk of bias
Chambergo-Michilot 2021	Peru	No	No	Yes	No	Probably Low risk of bias
Dorobantu 2010	Romania	No	Yes	Yes	No	Probably Low risk of bias
Dorobantu 2012	Romania	No	Yes	Yes	No	Probably Low risk of bias
Artyukhov 2017	Russia	No	No	No	No	Probably High risk of bias
Balanova 2019	Russia	No	No	No	No	Probably High risk of bias
Nahimana 2017	Rwanda	No	No	Yes	No	Probably Low risk of bias
Seck 2014b	Senegal	No	No	No	No	Probably High risk of bias
Lovic 2013	Serbia	No	No	No	No	Probably High risk of bias
Marinkovic 2014	Serbia	No	No	No	No	Probably High risk of bias
Odland 2020	Sierra Leone	No	No	Yes	No	Probably Low risk of bias
Geraedts 2021	Sierra Leone	No	No	Yes	No	Probably Low risk of bias
VanRooyen 2000	South Africa	No	Yes	No	Yes	Probably Low risk of bias
vanZyl 2012	South Africa	No	No	No	No	Probably High risk of bias
Kandala 2013	South Africa	No	No	Yes	No	Probably Low risk of bias
Dolman 2014	South Africa	No	Yes	Yes	No	Probably Low risk of bias

First author Year publication	Country	Urban and Rural definition used				Risk of bias – exposure
		Scales	Metrics	National/Official criteria (e.g., census)	Groups (e.g., ethnic, farmers)	
Egbujie 2016	South Africa	No	Yes	Yes	No	Probably Low risk of bias
Kandala 2021	South Africa	No	No	Yes	No	Probably Low risk of bias
Katulanda 2014	Sri Lanka	No	No	Yes	No	Probably Low risk of bias
Krishnadath 2016	Suriname	No	No	Yes	No	Probably Low risk of bias
Edwards 2000	Tanzania	No	No	No	No	Probably High risk of bias
Stanifer 2016	Tanzania	No	No	Yes	No	Probably Low risk of bias
Mosha 2017	Tanzania	No	No	No	No	Probably High risk of bias
Suriyawongpaisal 2003	Thailand	No	No	Yes	No	Probably Low risk of bias
Aekplakorn 2012	Thailand	No	No	Yes	No	Probably Low risk of bias
Bouguerra 2006	Tunisia	No	No	Yes	No	Probably Low risk of bias
Hammami 2011	Tunisia	No	No	No	No	Probably High risk of bias
Aounallah-Skhiri 2012	Tunisia	No	No	No	No	Probably High risk of bias
BenRomdhane 2012	Tunisia	No	No	Yes	No	Probably Low risk of bias
Sonmez 1999	Turkey	No	No	No	No	Probably High risk of bias
Altun 2005	Turkey	No	Yes	Yes	No	Probably Low risk of bias
Metintas 2009	Turkey	No	No	Yes	No	Probably Low risk of bias
Sengul 2016	Turkey	No	Yes	Yes	No	Probably Low risk of bias
Dastan 2017	Turkey	No	Yes	Yes	No	Probably Low risk of bias
Oğuz 2018	Turkey	No	Yes	Yes	No	Probably Low risk of bias
Musinguzi 2013	Uganda	No	Yes	Yes	No	Probably Low risk of bias
Guwatudde 2015	Uganda	No	Yes	Yes	No	Probably Low risk of bias
Nakibuuka 2015	Uganda	No	No	Yes	No	Probably Low risk of bias
Nieto-Martínez 2018	Venezuela	No	Yes	Yes	No	Probably Low risk of bias
Nguyen 2012	Vietnam	No	No	Yes	No	Probably Low risk of bias
Do 2015	Vietnam	No	No	Yes	No	Probably Low risk of bias
Jensen 2018	Vietnam	No	No	Yes	No	Probably Low risk of bias

First author Year publication	Country	Urban and Rural definition used				Risk of bias – exposure
		Scales	Metrics	National/Official criteria (e.g., census)	Groups (e.g., ethnic, farmers)	
Hoang 2019	Vietnam	No	No	Yes	No	Probably Low risk of bias
Abdul-Rahim 2001	West Bank and Gaza	No	Yes	Yes	No	Probably Low risk of bias
Modesti 2013a	Yemen	No	No	No	No	Probably High risk of bias

Characteristics of the 255 included studies arranged by country/year of publication: blood pressure definitions, features and outcome risk of bias

First author Year publication	Country	Hypertension definition included self-reported hypertension	Hypertension definition anti-hypertension drugs	Original Definition reported	Final definition (original or converted)	Definition used Society/ Organization criteria	Device name	Manual/ Automatic measurement	No. of measurements	No. of visits	Risk of b[illegible] outcon[illegible]
Abba 2022	Albania	No	Yes	140/90	140/90	Not specified	NR	NR	≥3	1	Probably Lo[illegible] of bias
Lamelas 2019	Argentina	No	Yes	140/90	140/90	Not specified	Omron digital BP device (Omron HEM-757; Omron Healthcare Inc., Lake Forest, Illinois, USA)	Not manual	2	1	Probably Lo[illegible] of bias
Sayeed 2002	Bangladesh	No	No	140/90	140/90	JNC	Mercury sphygmomanometer	Manual	2	1	Probably Lo[illegible] of bias
Hussain 2005	Bangladesh	No	No	140/90	140/90	Not specified	Standard mercury sphygmomanometer	Manual	NR	1	Probably Lo[illegible] of bias
Zaman 2015	Bangladesh	No	Yes	140/90	140/90	Not specified	Ordinary aneroid sphygmomanometers	Manual	2	1	Probably Lo[illegible] of bias
Biswas 2016	Bangladesh	No	No	140/90	140/90	AHA	Life Source UA-767 Plus blood pressure monitor (A&D Medical, San Jose, USA)	Not manual	≥3	1	Probably Lo[illegible] of bias
Rahman 2017	Bangladesh	No	Yes	140/90	140/90	AHA	Aneroid sphygmomanometer	Manual	2	1	Probably Lo[illegible] of bias
Islam 2018	Bangladesh	No	Yes	140/90	140/90	JNC	Calibrated aneroid sphygmomanometer	Manual	2	1	Probably Lo[illegible] of bias

First author Year publication	Country	Hypertension definition included self-reported hypertension	Hypertension definition anti-hypertension drugs	Original Definition reported	Final definition (original or converted)	Definition used Society/ Organization criteria	Device name	Manual/ Automatic measurement	No. of measurements	No. of visits	Risk of b outcon
Riaz 2020	Bangladesh	No	No	140/90	140/90	Not specified	Digital blood pressure measuring machine	Not manual	2	1	Probably Lo of bias
Hanif 2021	Bangladesh	Yes	No	140/90	140/90	JNC	Omron HEM 7120	Not manual	≥3	1	Probably Lo of bias
Hasan 2021	Bangladesh	Yes	No	140/90	140/90	Not specified	Omron HEM 7120	Not manual	≥3	1	Probably Lo of bias
Khanam 2021	Bangladesh	No	Yes	140/90	140/90	JNC	LIFE SOURCE® UA-767 Plus BP monitor	Not manual	2	1	Probably Lo of bias
Paul 2021	Bangladesh	No	Yes	140/90	140/90	AHA	Electronic BP Monitor (OMB) Model: BP-1307	Not manual	≥3	1	Probably Lo of bias
Delisle 2012	Benin	No	No	SBP/DBP	140/90	Not applicable	Standard mercury sphygmomanometer	Manual	2	1	Probably Lo of bias
Houehanou 2015	Benin	No	Yes	140/90	140/90	WHO	OMRON electronic BP monitor with a cuff	Not manual	≥3	1	Probably Lo of bias
Colette 2020	Benin	No	Yes	140/90	140/90	Not specified	NR	NR	2	1	Probably Lo of bias
Almeida 2015	Brazil	No	Yes	140/90	140/90	Not specified	OMRON HEM 7200 (Omron Healthcare, Inc., 1200 Lakeside Drive Bannockburn, IL 60015, USA)	Not manual	≥3	≥2	Probably Lo of bias
DePaula 2015	Brazil	No	No	140/90	140/90	ATP	Standard mercury sphygmomanometer	Manual	NR	1	Probably Lo of bias
Malta 2018	Brazil	Yes	Yes	140/90	140/90	Other	Calibrated digital device	Not manual	≥3	1	Probably Lo of bias
Santiago 2019	Brazil	No	Yes	140/90	140/90	Other	Glicomed™ sphygmomanometer, model CE-0483	Manual	2	1	Probably Lo of bias

First author Year publication	Country	Hypertension definition included self-reported hypertension	Hypertension definition anti-hypertension drugs	Original Definition reported	Final definition (original or converted)	Definition used Society/ Organization criteria	Device name	Manual/ Automatic measurement	No. of measurements	No. of visits	Risk of b[illegible] outcon[illegible]
deSouza 2020	Brazil	No	Yes	140/90	140/90	Other	Mercury column sphygmomanometer (model Glicomed-CE-0483, Accumed, Duque de Caxias, Brazil)	Manual	≥3	1	Probably Lo[illegible] of bias
Soubeiga 2017	Burkina Faso	No	Yes	140/90	140/90	Other	Blood pressure monitor (OMRON HEM-705 brand PC, Tokyo, Japan)	Not manual	≥3	1	Probably Lo[illegible] of bias
Wagner 2018	Cambodia	No	No	140/90	140/90	Not specified	Calibrated, digital sphygmomanometers by A&D Medical	Not manual	NR	1	Probably Hi[illegible] of bias
Cooper 1997	Cameroon	No	Yes	140/90	140/90	Not specified	Double-headed ('Y' terminal) stethoscopes.	Manual	≥3	1	Definitely L[illegible] of bias
Sobngwi 2002	Cameroon	No	Yes	140/90	140/90	Other	Standard mercury sphygmomanometer	Manual	2	1	Probably Lo[illegible] of bias
Fezeu 2010	Cameroon	No	Yes	140/90	140/90	Not specified	Standard mercury sphygmomanometer	Manual	≥3	1	Probably Lo[illegible] of bias
Lissock 2011	Cameroon	No	Yes	140/90	140/90	WHO	"Predicor" electronic sphygmomanometer	Not manual	2	1	Probably Lo[illegible] of bias
Kaze 2015	Cameroon	No	Yes	140/90	140/90	Not specified	Sphygmomanometer (OMRON HEM705CP, Omron Matsusaka Co, Matsusaka City, Mie-Ken, Japan)	Not manual	NR	1	Probably Lo[illegible] of bias

First author Year publication	Country	Hypertension definition included self-reported hypertension	Hypertension definition anti-hypertension drugs	Original Definition reported	Final definition (original or converted)	Definition used Society/ Organization criteria	Device name	Manual/ Automatic measurement	No. of measurements	No. of visits	Risk of b outcon
Lemogoum 2018	Cameroon	No	Yes	140/90	140/90	Other	Validated automated sphygmomanometer (HEM-705 CP, Omron Corporation, Tokyo, Japan)	Not manual	≥3	1	Probably Lo of bias
Tao 1995	China	No	Yes	140/90	140/90	Not specified	Standard mercury sphygmomanometer	Manual	≥3	1	Probably Lo of bias
Reynolds 2003	China	No	Yes	140/90	140/90	Other	Standard mercury sphygmomanometer	Manual	≥3	1	Probably Lo of bias
WangZ 2004	China	No	Yes	140/90	140/90	Not specified	Standard mercury sphygmomanometer	Manual	≥3	1	Probably Lo of bias
Wu 2008	China	No	Yes	140/90	140/90	Other	Standard mercury sphygmomanometer	Manual	2	1	Probably Lo of bias
Xu 2008	China	No	Yes	140/90	140/90	Not specified	Standard mercury sphygmomanometer	Manual	NR	1	Probably Lo of bias
Zhang 2008	China	No	Yes	140/90	140/90	Not specified	NR	NR	≥3	1	Probably Lo of bias
Zuo 2009	China	No	No	130/85	140/90	ATP	Standard mercury sphygmomanometer	Manual	≥3	1	Probably Lo of bias
Fu 2010	China	No	No	140/90	140/90	Other	Standard mercury sphygmomanometer	Manual	≥3	1	Probably Lo of bias
Zhao 2011	China	No	Yes	130/85	140/90	Other	NR	NR	≥3	1	Probably Lo of bias
Cai 2012	China	No	Yes	140/90	140/90	Not specified	Calibrated mercury sphygmomanometer	Manual	2	1	Probably Lo of bias

First author Year publication	Country	Hypertension definition included self-reported hypertension	Hypertension definition anti-hypertension drugs	Original Definition reported	Final definition (original or converted)	Definition used Society/ Organization criteria	Device name	Manual/ Automatic measurement	No. of measurements	No. of visits	Risk of b[illegible] outcon[illegible]
Zheng 2012	China	No	Yes	140/90	140/90	Other	Standard mercury sphygmomanometer	Manual	2	1	Probably Lo[illegible] of bias
Gao 2013	China	No	Yes	140/90	140/90	JNC	Standard mercury sphygmomanometer	Manual	2	1	Probably Lo[illegible] of bias
Lao 2013	China	No	Yes	140/90	140/90	Other	- Standard mercury sphygmomanometer, and<br>- Omron sphygmomanometers	Manual & digital	2	1	Probably Lo[illegible] of bias
WangH 2013	China	No	Yes	140/90	140/90	Not specified	Standard mercury sphygmomanometer	Manual	2	1	Probably Lo[illegible] of bias
Xu 2013	China	No	Yes	140/90	140/90	Other	Standard mercury sphygmomanometer	Manual	2	1	Probably Lo[illegible] of bias
Bi 2014	China	No	Yes	140/90	140/90	Other	Electronic Sphygmomanometer (HEM - 7071 Omron Corporation, Kyoto, Japan	Not manual	≥3	1	Probably Lo[illegible] of bias
Fan 2014	China	No	Yes	140/90	140/90	Not specified	- OMRON HEM-7071 (OMRON, Kyoto, Japan), and<br>- Mercury sphygmomanometer	Not manual	2	1	Probably Lo[illegible] of bias
WangJ 2014	China	No	Yes	140/90	140/90	Not specified	Standard mercury sphygmomanometer	Manual	≥3	1	Probably Lo[illegible] of bias

First author Year publication	Country	Hypertension definition included self-reported hypertension	Hypertension definition anti-hypertension drugs	Original Definition reported	Final definition (original or converted)	Definition used Society/ Organization criteria	Device name	Manual/ Automatic measurement	No. of measurements	No. of visits	Risk of b outcon
Attard 2015	China	Yes	No	140/90	140/90	JNC	Standard mercury sphygmomanometer	Not manual	≥3	1	Probably Lo of bias
Bi 2015	China	No	No	140/90	140/90	AHA	OMRON model HEM-7071, Omron Co., Kyoto, Japan	Not manual	≥3	1	Probably Lo of bias
Ma 2015	China	Yes	Yes	140/90	140/90	Not specified	Standard mercury sphygmomanometer	Manual	2	1	Probably Lo of bias
Wei 2015	China	No	Yes	140/90	140/90	WHO	Mercury sphygmomanometer (Jiangsu Yuyue medical equipment and supply Co., Ltd., Danyang City, China)	Manual	≥3	1	Probably Lo of bias
Hu 2016	China	Yes	Yes	140/90	140/90	Other	Standard mercury sphygmomanometer	Manual	≥3	1	Probably Lo of bias
Huang 2016	China	Yes	Yes	140/90	140/90	Other	Standard mercury sphygmomanometer	Manual	≥3	1	Probably Lo of bias
Lewington 2016	China	No	Yes	140/90	140/90	Not specified	Digital sphygmomanometer (Omron UA-779; Live Source)	Not manual	2	1	Probably Lo of bias
Li W 2016	China	Yes	Yes	140/90	140/90	Not specified	Omron automatic digital BP measuring device (Omron HEM-757; Omron, Kyoto, Japan)	Not manual	2	1	Probably Lo of bias

First author Year publication	Country	Hypertension definition included self-reported hypertension	Hypertension definition anti-hypertension drugs	Original Definition reported	Final definition (original or converted)	Definition used Society/ Organization criteria	Device name	Manual/ Automatic measurement	No. of measurements	No. of visits	Risk of b outcon
Wu 2016	China	No	Yes	140/90	140/90	Not specified	Omron HEM-7000 electronic sphygmomanometer (Omron Healthcare; Muko, Kyoto, Japan)	Not manual	NR	1	Probably Hi of bias
Yang 2016	China	No	Yes	140/90	140/90	Other	OMRON HBP-1300 Professional Portable Blood Pressure Monitor (OMRON, Kyoto, Japan)	Not manual	≥3	1	Probably Lo of bias
Zhang 2016	China	No	Yes	140/90	140/90	Other	NR	NR	NR	1	Probably Hi of bias
Hu 2017	China	No	Yes	140/90	140/90	JNC	Omron HBP-1300 Professional Portable Blood Pressure Monitor (Kyoto, Japan)	Not manual	≥3	1	Probably Lo of bias
Huang 2017	China	No	Yes	140/90	140/90	JNC	Standard mercury sphygmomanometer	Manual	NR	1	Probably Lo of bias
Li J 2017	China	Yes	No	140/90	140/90	JNC	NR	NR	≥3	1	Probably Lo of bias
Li Q 2017	China	No	No	SBP	140/90	Not applicable	Automated Sphygmomanometer	Not manual	≥3	1	Probably Lo of bias
Li Y 2017	China	No	Yes	140/90	140/90	Not specified	Omron digital BP device (HBP-1300) with a wide range cuff (9-17 inches)	Not manual	≥3	1	Probably Lo of bias
Liu X 2017a	China	No	Yes	140/90	140/90	Not specified	Standard mercury sphygmomanometer	Manual	≥3	1	Probably Lo of bias

First author Year publication	Country	Hypertension definition included self-reported hypertension	Hypertension definition anti-hypertension drugs	Original Definition reported	Final definition (original or converted)	Definition used Society/ Organization criteria	Device name	Manual/ Automatic measurement	No. of measurements	No. of visits	Risk of b[illegible] outcon[illegible]
Liu X 2017b	China	No	Yes	140/90	140/90	Not specified	Electronic sphygmomanometer	Not manual	≥3	1	Probably Lo[illegible] of bias
Lu 2017	China	No	Yes	140/90	140/90	JNC	Electronic blood pressure monitor (Omron HEM-7430; Omron Corporation, Kyoto, Japan)	Not manual	2	1	Probably Lo[illegible] of bias
ZhangFL 2017	China	Yes	Yes	140/90	140/90	Not specified	OMRON sphygmomanometer (OMRON HEM- 7200, Kyoto, Japan)	Not manual	2	1	Probably Lo[illegible] of bias
WangJ 2018	China	No	Yes	140/90	140/90	Other	Standard mercury-based sphygmomanometer	Manual	≥3	1	Probably Lo[illegible] of bias
WangZ 2018	China	No	Yes	140/90	140/90	Other	OMRON HBP-1300 professional portable blood pressure monitor	Not manual	≥3	1	Probably Lo[illegible] of bias
Du 2019	China	Yes	Yes	140/90	140/90	Other	HEM-7071, Omron Corp., Japan	Not manual	≥3	1	Probably Lo[illegible] of bias
Wang 2019	China	No	Yes	140/90	140/90	Not specified	Omron HEM-1300	Not manual	≥3	1	Probably Lo[illegible] of bias
Wei 2019	China	Yes	No	140/90	140/90	Other	Mercury sphygmomanometer	Manual	2	1	Probably Lo[illegible] of bias
Xing 2019	China	No	Yes	140/90	140/90	Other	J30; Omron, Kyoto, Japan	Not manual	≥3	1	Probably Lo[illegible] of bias
Ding 2020	China	No	Yes	140/90	140/90	JNC	OMRON Model HEM-7112, Omron Company	Not manual	≥3	1	Probably Lo[illegible] of bias
Han 2020	China	Yes	No	140/90	140/90	Not specified	Sphygmomanometer (TERUMO-Elemano)	Not manual	≥3	1	Probably Lo[illegible] of bias

First author Year publication	Country	Hypertension definition included self-reported hypertension	Hypertension definition anti-hypertension drugs	Original Definition reported	Final definition (original or converted)	Definition used Society/ Organization criteria	Device name	Manual/ Automatic measurement	No. of measurements	No. of visits	Risk of b outcon
Li 2020	China	No	Yes	140/90	140/90	Not specified	Omron HBP-1300 Professional Portable Blood Pressure Monitor (OMRON, Japan)	Not manual	≥3	1	Probably Lo of bias
Ma 2020	China	No	Yes	130/85	140/90	Other	OMRON HEM1300 (OMRON, Dalian, China)	Not manual	≥3	1	Probably Lo of bias
Su 2020	China	No	Yes	130/85	140/90	Other	OMRON Model HEM-7071, Omron Company	Not manual	≥3	1	Probably Lo of bias
Li 2021	China	No	No	SBP/DBP	140/90	Not specified	NR	NR	NR	1	Probably Hi of bias
Ma 2021	China	No	Yes	140/90	140/90	Other	Mercury sphygmomanometer	Manual	≥3	1	Probably Lo of bias
Yu 2021	China	Yes	Yes	140/90	140/90	JNC	Omron L10; Dalian, China	Not manual	2	1	Probably Lo of bias
Camacho 2016	Colombia	Yes	Yes	140/90	140/90	JNC	Sphygmomanometer (Omron HEM-757; Omron Healthcare Co. Ltd., Scarborough, Ontario, Canada)	Not manual	2	1	Probably Lo of bias
Longo-Mbenza 2008	Democratic Republic of the Congo	No	Yes	140/90	140/90	WHO	Electronically (model HEM-705 CP, OMRON, Kyoto, Japan)	Not manual	≥3	1	Probably Lo of bias
Katchunga 2019	Democratic Republic of the Congo	No	Yes	140/90	140/90	Not specified	OMRON Hem 7001E	Not manual	≥3	1	Probably Lo of bias
Masimango 2020	Democratic Republic of the Congo	No	Yes	140/90	140/90	JNC	OMRON M6 Comfort; OMRON HealthCare Co., Ltd, Kyoto, Japan	Not manual	≥3	1	Probably Lo of bias
Markovic 2011 / Bergman	Croatia	No	Yes	140/90	140/90	Other	Mercury sphygmomanometer	Manual	2	1	Probably Lo of bias

First author Year publication	Country	Hypertension definition included self-reported hypertension	Hypertension definition anti-hypertension drugs	Original Definition reported	Final definition (original or converted)	Definition used Society/ Organization criteria	Device name	Manual/ Automatic measurement	No. of measurements	No. of visits	Risk of b outcon
Felix 2020	Ecuador	No	Yes	140/90	140/90	Not specified	Omron HEM-RML31; Omron Healthcare Co. Ltd., Scarborough, Ontario, Canada	Not manual	2	1	Probably Lo of bias
Pérez-Galarza 2021	Ecuador	No	No	130/85	140/90	Other	Digital sphygmomanometer	Not manual	≥3	1	Probably Lo of bias
Orantes-Navarro 2019	El Salvador	Yes	No	140/90	140/90	Not specified	RIESTER model RI-Champion, Germany	Not manual	NR	1	Probably Lo of bias
Mufunda 2006	Eritrea	No	No	140/90	140/90	Not specified	OMRON digital sphygmomanometer (OMRON M7 Duo 773 OMRON Healthcare Europe B.V. Kruisweg 577, 2132 NA Hoofddorp)	Not manual	2	1	Probably Lo of bias
Giday 2011	Ethiopia	No	Yes	140/90	140/90	WHO	NR	NR	≥3	1	Probably Lo of bias
Muluneh AT 2012	Ethiopia	No	No	140/90	140/90	Not specified	Digital blood pressure monitor, e.g. Bosch & Sohn Medicus UNO, or OMRON M6	Not manual	≥3	1	Probably Lo of bias
Mengistu 2014	Ethiopia	No	Yes	140/90	140/90	JNC	NR	NR	2	1	Probably Lo of bias
Abebe 2015	Ethiopia	No	Yes	140/90	140/90	WHO	Mars MS-700AMI	Not manual	≥3	1	Probably Lo of bias
Gebreyes 2018	Ethiopia	No	Yes	140/90	140/90	Not specified	Boso-Medicus Uno	Not manual	≥3	1	Probably Lo of bias
Tesfaye 2019	Ethiopia	Yes	No	140/90	140/90	Not specified	Ce/ISO Approved Medical Adult Aneroid Sphygmomanometer MT01028001	Manual	≥3	≥2	Probably Lo of bias

First author Year publication	Country	Hypertension definition included self-reported hypertension	Hypertension definition anti-hypertension drugs	Original Definition reported	Final definition (original or converted)	Definition used Society/ Organization criteria	Device name	Manual/ Automatic measurement	No. of measurements	No. of visits	Risk of b outcon
VanDerSande 2000	Gambia	No	Yes	140/90	140/90	WHO	Validated oscillometric automated digital BP machine (Omron HEM-705CP, Japan20)	Not manual	2	1	Probably Lo of bias
Cham 2018	Gambia	Yes	Yes	140/90	140/90	WHO	Automated sphygmomanometer	Not manual	≥3	1	Probably Lo of bias
Agyemang 2006	Ghana	No	Yes	140/90	140/90	Not specified	Omron M5-I monitor	Not manual	2	1	Probably Lo of bias
Obirikorang 2015	Ghana	No	No	140/90	140/90	WHO	- Sphygmomanometer, and<br>- Stetescope	Manual	NR	1	Probably Lo of bias
Kodaman 2016	Ghana	No	Yes	140/90	140/90	JNC	Omron HEM-705c instrument (Omron Healthcare Corp., Bannockburn, Illinois, USA)	Not manual	2	1	Probably Lo of bias
Agyemang 2018	Ghana	No	Yes	140/90	140/90	Not specified	Microlife Watch BP home Widnau, Switzerland	Not manual	2	1	Probably Lo of bias
Sanuade 2018	Ghana	No	No	140/90	140/90	Other	LIFE SOURCE UA-767 Plus	Not manual	≥3	1	Probably Lo of bias
Balde 2007	Guinea	No	Yes	140/90	140/90	Not specified	Sphygmomanometer	Manual	2	1	Probably Lo of bias
Camara 2016	Guinea	No	Yes	140/90	140/90	WHO	Sphygmomanometers (OMRON M2 Basic, OMRON HEALTHCARE Co. Ltd, Kyoto, Japan).	Not manual	≥3	1	Probably Lo of bias

First author Year publication	Country	Hypertension definition included self-reported hypertension	Hypertension definition anti-hypertension drugs	Original Definition reported	Final definition (original or converted)	Definition used Society/ Organization criteria	Device name	Manual/ Automatic measurement	No. of measurements	No. of visits	Risk of b outcon
DeGennaroJr 2018	Haiti	No	Yes	140/90	140/90	Not specified	Omron Series 7 electronic wrist blood pressure device	Not manual	2	1	Probably Lo of bias
Singh 1997a	India	No	No	140/90	140/90	Other	Standard mercury sphygmomanometer	Manual	NR	1	Probably Lo of bias
Kusuma 2004	India	No	No	140/90	140/90	JNC	Standard mercury sphygmomanometer	Manual	≥3	1	Probably Lo of bias
Kumar 2006	India	No	Yes	140/90	140/90	JNC	NR	NR	≥3	1	Probably Lo of bias
Kusuma 2008	India	No	No	140/90	140/90	JNC	Standard mercury sphygmomanometer	Manual	NR	1	Probably Lo of bias
Gupta 2009	India	Yes	No	140/90	140/90	JNC	Standard mercury sphygmomanometer	Manual	≥3	1	Probably Lo of bias
Midha 2009	India	No	No	140/90	140/90	JNC	Standard mercury sphygmomanometer (Diamond Co.)	Manual	2	1	Probably Lo of bias
Allender 2010	India	No	No	140/90	140/90	Not specified	Omron blood pressure monitor	Not manual	2	1	Probably Lo of bias
Thankappan 2010	India	No	Yes	140/90	140/90	JNC	OMRON -4, Omron Corporation, Kyoto, Japan	Not manual	≥3	1	Probably Lo of bias
Das 2011	India	No	No	130/85	140/90	AHA	- Sphygmomanometer, and<br>- Stetescope	Not manual	2	1	Probably Lo of bias

First author Year publication	Country	Hypertension definition included self-reported hypertension	Hypertension definition anti-hypertension drugs	Original Definition reported	Final definition (original or converted)	Definition used Society/ Organization criteria	Device name	Manual/ Automatic measurement	No. of measurements	No. of visits	Risk of b[illegible] outcon[illegible]
Bharati 2012	India	No	No	140/90	140/90	JNC	Standard mercury sphygmomanometer	Manual	2	1	Probably Lo[illegible] of bias
Samuel 2012	India	Yes	Yes	140/90	140/90	JNC	OMRON 711 device	Not manual	2	1	Probably Lo[illegible] of bias
Bhagyalaxmi 2013	India	No	Yes	140/90	140/90	WHO	OMRON-HEM7111, OMRON Healthcare Co. Ltd. Uky-Ku, Kyoto, Japan	Not manual	2	1	Probably Lo[illegible] of bias
Millett 2013	India	Yes	No	140/90	140/90	Not specified	Omron M5-I automatic machine	Not manual	2	1	Probably Lo[illegible] of bias
Bhadoria 2014	India	Yes	No	140/90	140/90	WHO	Omron HEM-7080, Omron Corporation, Kyoto, Japan	Not manual	≥3	1	Probably Lo[illegible] of bias
Farag 2014	India	Yes	Yes	140/90	140/90	JNC	Standard mercury sphygmomanometer	Manual	2	1	Probably Lo[illegible] of bias
Bhansali 2015	India	No	Yes	140/90	140/90	JNC	OMRON HEM 7101 machine (Omron Corporation, Tokyo, Japan)	Not manual	2	1	Probably Lo[illegible] of bias
Norboo 2015	India	No	Yes	140/90	140/90	JNC	Device (HEM 7000; OMRON Life Science Co. Ltd, Kyoto, Japan)	Not manual	2	1	Probably Lo[illegible] of bias
Krishnan 2016	India	No	Yes	140/90	140/90	JNC	Electronic apparatus (model 1A2, Omron Corporation, Shimogyo-ku, Kyoto, Japan)	Not manual	≥3	1	Probably Lo[illegible] of bias

First author Year publication	Country	Hypertension definition included self-reported hypertension	Hypertension definition anti-hypertension drugs	Original Definition reported	Final definition (original or converted)	Definition used Society/ Organization criteria	Device name	Manual/ Automatic measurement	No. of measurements	No. of visits	Risk of b outcon
Oommen 2016a	India	No	Yes	140/90	140/90	JNC	Monitor (Omron HEM 7080, Kyoto, Japan)	Not manual	≥3	1	Probably Lo of bias
Oommen 2016b	India	No	Yes	140/90	140/90	JNC	Random zero sphygmomanometer	Manual	≥3	1	Probably Lo of bias
Bandela 2017	India	No	No	SBP/DBP	140/90	Not applicable	Sphygmomanometer	Manual	2	1	Probably Lo of bias
Kanungo 2017	India	No	No	140/90	140/90	JNC	Rossmax - AW150 Blood Pressure Monitor (Rossmax International Ltd., Berneck, Switzerland)	Not manual	2	1	Probably Lo of bias
Prabhakaran 2017	India	No	Yes	140/90	140/90	JNC	- Random 0 sphygmomanometer and - OMRON (HEM-7080) digital blood pressure	Manual & digital	2	1	Probably Lo of bias
Tripathy 2017	India	No	Yes	140/90	140/90	JNC	OMRON HEM 7120, Omron Corporation, Kyoto, Japan	Not manual	≥3	1	Probably Lo of bias
Geldsetzer 2018	India	No	No	140/90	140/90	Other	BP monitor (Rossmax AW150)	Not manual	2	1	Probably Lo of bias
Sarma 2019	India	No	Yes	140/90	140/90	JNC	Omron HEM-7120	Not manual	≥3	1	Probably Lo of bias
Kokane 2020	India	No	Yes	140/90	140/90	JNC	NR	NR	2	1	Probably Lo of bias
Mohanty 2020	India	No	No	140/90	140/90	AHA	Automated oscillometric machines	Not manual	2	1	Probably Lo of bias

First author Year publication	Country	Hypertension definition included self-reported hypertension	Hypertension definition anti-hypertension drugs	Original Definition reported	Final definition (original or converted)	Definition used Society/ Organization criteria	Device name	Manual/ Automatic measurement	No. of measurements	No. of visits	Risk of b[illegible] outcon[illegible]
Kumar 2021	India	No	Yes	140/90	140/90	Not specified	OMRON BP monitor	Not manual	2	1	Probably Lo[illegible] of bias
Mohanty 2021	India	Yes	Yes	140/90	140/90	Not specified	Omron Healthcare Vietnam Co. Ltd, Vietnam	Not manual	≥3	1	Probably Lo[illegible] of bias
Nanditha 2021	India	Yes	No	140/90	140/90	Not specified	Omron Corporation, Tokyo, Japan	Not manual	2	1	Probably Lo[illegible] of bias
Patel 2021	India	No	Yes	140/90	140/90	Not specified	OMRON BP monitor	Not manual	≥3	1	Probably Lo[illegible] of bias
Sivanantham 2021	India	No	Yes	140/90	140/90	JNC	OMRON, HEM 7120, Omron Corporation, Kyoto, JAPAN	Not manual	≥3	1	Probably Lo[illegible] of bias
Hussain 2016	Indonesia	Yes	Yes	140/90	140/90	JNC	Omron digital self-inflating sphygmomanometers	Not manual	≥3	1	Probably Lo[illegible] of bias
Maharani 2019	Indonesia	No	Yes	140/90	140/90	Not specified	OMRON HEM-7130 made in Japan	Not manual	≥3	1	Probably Lo[illegible] of bias
Sujarwoto 2020	Indonesia	No	Yes	140/90	140/90	Other	OMROM HEM-7130	Not manual	≥3	1	Probably Lo[illegible] of bias
SarrafZadegan 1997	Iran	No	Yes	140/90	140/90	Not specified	Standard mercury sphygmomanometer	Manual	≥3	1	Probably Lo[illegible] of bias
Janghorbani 2008	Iran	No	Yes	140/90	140/90	JNC	Standard mercury sphygmomanometer	Manual	≥3	1	Probably Lo[illegible] of bias
Azimi-Nezhad 2009a	Iran	Yes	Yes	140/90	140/90	JNC	Standard mercury sphygmomanometer	Manual	2	1	Probably Lo[illegible] of bias
Ebrahimi 2010	Iran	No	Yes	140/90	140/90	Not specified	Aneroid sphygmomanometer	Manual	2	1	Probably Lo[illegible] of bias

First author Year publication	Country	Hypertension definition included self-reported hypertension	Hypertension definition anti-hypertension drugs	Original Definition reported	Final definition (original or converted)	Definition used Society/ Organization criteria	Device name	Manual/ Automatic measurement	No. of measurements	No. of visits	Risk of b[illegible] outco[illegible]
Shirani 2011	Iran	No	Yes	140/90	140/90	WHO	NR	Manual	≥3	1	Probably Lo[illegible] of bias
Malekzadeh 2013	Iran	Yes	Yes	140/90	140/90	JNC	NR	NR	2	1	Probably Lo[illegible] of bias
Esteghamati 2016	Iran	No	Yes	140/90	140/90	Not specified	- Standard mercury sphygmomanometer (2005), - Calibrated Omron M7 digital sphygmomanometers (Hoofddorp, Netherlands)	Manual & digital	≥3	1	Probably Lo[illegible] of bias
Khorrami 2017	Iran	No	No	140/90	140/90	Not specified	Omron electronic sphygmomanometer	Not manual	≥3	1	Probably Lo[illegible] of bias
Rajati 2019	Iran	No	Yes	140/90	140/90	JNC	Riester duplex blood pressure	Manual	2	1	Probably Lo[illegible] of bias
Katibeh 2020	Iran	Yes	Yes	140/90	140/90	JNC	Standard mercury sphygmomanometer (nova-presameter; Riester, Jungingen, Germany)	Manual	2	≥2	Probably Lo[illegible] of bias
Ahmadi 2021	Iran	Yes	Yes	140/90	140/90	Not specified	Standard barometer (Richter Japan)	Manual	2	1	Probably Lo[illegible] of bias
Jalali 2021	Iran	No	Yes	140/90	140/90	Other	Calibrated digital sphygmomanometers	Not manual	2	1	Probably Lo[illegible] of bias
Naghipour 2021	Iran	Yes	Yes	140/90	140/90	Other	Richter auscultator mercury sphygmomanometers (MTM Munich, Germany)	Manual	2	1	Probably Lo[illegible] of bias

First author Year publication	Country	Hypertension definition included self-reported hypertension	Hypertension definition anti-hypertension drugs	Original Definition reported	Final definition (original or converted)	Definition used Society/ Organization criteria	Device name	Manual/ Automatic measurement	No. of measurements	No. of visits	Risk of b[illegible] outcon[illegible]
Rezaianzadeh 2021	Iran	No	Yes	140/90	140/90	Other	Standard calibrated sphygmomanometer (Reister Model, Germany)	Manual	2	1	Probably Lo[illegible] of bias
Allameh 2022	Iran	Yes	Yes	140/90	140/90	Other	NR.	NR	2	1	Probably Lo[illegible] of bias
Ferguson 2011	Jamaica	No	Yes	140/90	140/90	JNC	Standard mercury sphygmomanometer	Manual	≥3	1	Probably Lo[illegible] of bias
Supiyev 2016	Kazakhstan	No	Yes	140/90	140/90	Not specified	NR	NR	≥3	1	Probably Lo[illegible] of bias
Mathenge 2010	Kenya	No	Yes	140/90	140/90	JNC	Omron digital automatic monitor (model HEM907)	Not manual	≥3	1	Probably Lo[illegible] of bias
Walekhwa 2021	Kenya	No	Yes	140/90	140/90	AHA	OMRON®, Kyoto, Japan	Not manual	≥3	1	Probably Lo[illegible] of bias
Fahs 2017	Lebanon	No	No	140/90	140/90	JNC	Digital blood pressure (Omron, M3 IT) machine	Not manual	≥3	1	Probably Lo[illegible] of bias
Ratovoson 2015	Madagascar	No	Yes	140/90	140/90	JNC	Digital blood pressure monitor, OMRON M3	Not manual	2	1	Probably Lo[illegible] of bias
Msyamboza 2011	Malawi	No	Yes	140/90	140/90	Not specified	Blood pressure machines (Omron H M4-I)	Not manual	2	1	Probably Lo[illegible] of bias
Price 2018	Malawi	No	Yes	140/90	140/90	Not specified	Portable sphygmomanometers (OMRON-Healthcare-Co HEM-7211-E-Model-M6; Kyoto, Japan)	Not manual	≥3	1	Probably Lo[illegible] of bias

First author Year publication	Country	Hypertension definition included self-reported hypertension	Hypertension definition anti-hypertension drugs	Original Definition reported	Final definition (original or converted)	Definition used Society/ Organization criteria	Device name	Manual/ Automatic measurement	No. of measurements	No. of visits	Risk of b[illegible] outco[illegible]
Mohamud 2012	Malaysia	No	Yes	130/85	140/90	Other	Omron digital sphygmomanometer	Not manual	2	1	Probably Lo[illegible] of bias
Abdul-Razak 2016	Malaysia	Yes	Yes	140/90	140/90	Other	Omron HEM-757	Not manual	2	1	Probably Lo[illegible] of bias
Naing 2016	Malaysia	Yes	Yes	140/90	140/90	JNC	Blood pressure measurement device, the Omron HEM-907	Not manual	2	1	Probably Lo[illegible] of bias
Naidu 2019	Malaysia	Yes	No	140/90	140/90	Not specified	Omron Digital Automatic Blood Pressure Monitor Model HEM-907	Not manual	NR	1	Probably Lo[illegible] of bias
Isa 2021	Malaysia	No	No	140/90	140/90	Not specified	Omron HEM-757	Not manual	2	1	Probably Lo[illegible] of bias
BA 2018	Mali	No	Yes	140/90	140/90	JNC	Frangly aneroid sphygmomanometer with a medium and large cuff size	Manual	2	1	Probably Lo[illegible] of bias
Guerrero-Romero 2000	Mexico	Yes	Yes	140/90	140/90	JNC	Standard mercury sphygmomanometer	Manual	NR	1	Probably Lo[illegible] of bias
Beltran-Sanchez 2011	Mexico	No	Yes	140/90	140/90	Not specified	Electronic monitor	Not manual	NR	1	Probably Lo[illegible] of bias
Hosey 2014	Micronesia	No	No	SBP/DBP	140/90	Not applicable	OMRON M4 Digital Automatic Blood Pressure Monitor	Not manual	≥3	1	Probably Lo[illegible] of bias
Pengpid 2022	Mongolia	No	Yes	130/85	140/90	Other	OMRON Model M5 automatic blood pressure monitor	Not manual	≥3	1	Probably Lo[illegible] of bias

First author Year publication	Country	Hypertension definition included self-reported hypertension	Hypertension definition anti-hypertension drugs	Original Definition reported	Final definition (original or converted)	Definition used Society/ Organization criteria	Device name	Manual/ Automatic measurement	No. of measurements	No. of visits	Risk of b outcon
Tazi 2003	Morocco	No	Yes	140/90	140/90	Other	Vaquez-type sphygmomanometer	Manual	≥3	1	Probably Lo of bias
Ziyyat 2014	Morocco	No	No	140/90	140/90	Other	NR	NR	NR	1	Probably Hi of bias
Pengpid 2020	Morocco	No	Yes	130/85	140/90	Other	Electronic blood pressure monitor Spengler® ES 60	Not manual	2	1	Probably Lo of bias
Damasceno 2009	Mozambique	No	Yes	140/90	140/90	Not specified	Sphygmomanometer (Omron 3)	Not manual	2	1	Probably Lo of bias
Jessen 2018	Mozambique	No	Yes	140/90	140/90	Not specified	Sphygmomanometer (Bosch & Sohn Medicus UNO, Germany)	Not manual	≥3	1	Probably Lo of bias
Mika 2020	Mozambique	No	No	140/90	140/90	Other	Orbis Pharma BP-1305	Not manual	≥3	1	Probably Lo of bias
Quasem 2001	Multicountry	No	Yes	140/90	140/90	Other	Mercury column sphygmomanometer (Diamond Co., Indus-trial Electronics and Allied Products, Electronics Cooperative Estate, Pune, Maharashtra, India)	Manual	2	1	Probably Lo of bias
Prince 2012	Multicountry	Yes	No	140/90	140/90	WHO	NR	NR	2	1	Probably Hi of bias
Basu 2013	Multicountry	Yes	Yes	140/90	140/90	Not specified	Medistar Wrist Blood Pressure Model S	Not manual	≥3	1	Probably Lo of bias

First author Year publication	Country	Hypertension definition included self-reported hypertension	Hypertension definition anti-hypertension drugs	Original Definition reported	Final definition (original or converted)	Definition used Society/ Organization criteria	Device name	Manual/ Automatic measurement	No. of measurements	No. of visits	Risk of b[illegible] outco[illegible]
Chow 2013	Multicountry	No	Yes	140/90	140/90	Not specified	Omron digital blood pressure measuring device (Omron HEM-757)	Not manual	2	1	Probably Lo[illegible] of bias
Harhay 2013	Multicountry	No	Yes	140/90	140/90	JNC	Digital oscillometric blood pressure measuring device	Not manual	≥3	1	Probably Lo[illegible] of bias
Kavishe 2015	Multicountry	No	Yes	140/90	140/90	JNC	Omron digital automatic blood pressure monitor model M6 (Omron Health Care Manufacturing Vietnam Co., Ltd, Binh Duong Province, Vietnam)	Not manual	≥3	1	Probably Lo[illegible] of bias
Gupta 2017	Multicountry	Yes	Yes	140/90	140/90	Not specified	BP Omron HEM-757 instruments (Omron Healthcare, Lake Forest, IL)	Not manual	≥3	1	Probably Lo[illegible] of bias
Bjertness 2016	Myanmar	No	Yes	140/90	140/90	WHO	Sphygmomanometer "OMRON"	Not manual	≥3	1	Probably Lo[illegible] of bias
Htet 2017	Myanmar	No	Yes	140/90	140/90	WHO	OMRON M4-1 (Japan) in 2004 and OMRON M6 (Japan) in 2014	Not manual	≥3	1	Probably Lo[illegible] of bias
Craig 2018	Namibia	No	Yes	140/90	140/90	WHO	Life Source UA-767 Plus	Not manual	≥3	1	Probably Lo[illegible] of bias
Aryal 2015	Nepal	No	Yes	140/90	140/90	Not specified	(OMRON digital device, OMRON, Netherlands	Not manual	≥3	1	Probably Lo[illegible] of bias

First author Year publication	Country	Hypertension definition included self-reported hypertension	Hypertension definition anti-hypertension drugs	Original Definition reported	Final definition (original or converted)	Definition used Society/ Organization criteria	Device name	Manual/ Automatic measurement	No. of measurements	No. of visits	Risk of b[illegible] outcon[illegible]
Mehata 2018	Nepal	No	Yes	140/90	140/90	WHO	UA-767F/FAC (A&D Medical, Tokyo, Japan) blood pressure monitors	Not manual	≥3	1	Probably Lo[illegible] of bias
Laux 2012	Nicaragua	Yes	No	140/90	140/90	Not specified	Calibrated M7 sphygmomanometer (Omron, Kyoto, Japan)	Not manual	NR	1	Probably Lo[illegible] of bias
Ezenwaka 1997	Nigeria	No	No	SBP/DBP	140/90	Not applicable	Standard mercury gauge sphygmomanometer (cuff size 23*22.5cm, Accoson, England)	Manual	≥3	1	Probably Lo[illegible] of bias
Okosun	Nigeria	No	Yes	140/90	140/90	JNC	Standard mercury sphygmomanometer	Manual	≥3	1	Probably Lo[illegible] of bias
Isezuo SA 2011	Nigeria	No	Yes	140/90	140/90	JNC	Omron SME-1 Omron Healthcare Ltd, Kyoto, Japan	Not manual	≥3	1	Probably Lo[illegible] of bias
Abegunde 2013	Nigeria	No	Yes	140/90	140/90	JNC	Accoson mercury sphygmomanometer	Manual	2	1	Probably Lo[illegible] of bias
Adediran 2013	Nigeria	No	Yes	140/90	140/90	WHO	Arcusson mercury sphygmomanometer	Manual	≥3	1	Probably Lo[illegible] of bias
Ejim 2013	Nigeria	No	Yes	140/90	140/90	WHO	Standard mercury sphygmomanometer	Manual	2	1	Probably Lo[illegible] of bias

First author Year publication	Country	Hypertension definition included self-reported hypertension	Hypertension definition anti-hypertension drugs	Original Definition reported	Final definition (original or converted)	Definition used Society/ Organization criteria	Device name	Manual/ Automatic measurement	No. of measurements	No. of visits	Risk of b outcon
Murthy 2013	Nigeria	No	No	140/90	140/90	WHO	Omron wrist instrument (UB322, Omron Healthcare Ltd, Milton Keynes, England)	Not manual	≥3	1	Probably Lo of bias
Okpechi 2013	Nigeria	No	Yes	140/90	140/90	Other	Omron Digital Blood Pressure machine (Omron M2 automatic BP monitor - Tokyo Japan)	Not manual	≥3	1	Probably Lo of bias
Oguoma 2015	Nigeria	No	No	130/85	140/90	Other	Digital blood pressure machine (Omron®, Australia)	Not manual	≥3	1	Probably Lo of bias
Odili 2020	Nigeria	No	Yes	140/90	140/90	Other	Mercury sphygmomanometer	Manual	≥3	1	Probably Lo of bias
Umuerri 2020	Nigeria	Yes	No	140/90	140/90	JNC	Omron® BP-785 Intellisense	Not manual	≥3	1	Probably Lo of bias
Jafar 2003	Pakistan	No	Yes	140/90	140/90	Other	Standard mercury sphygmomanometer	Manual	2	1	Probably Lo of bias
Tareen 2011	Pakistan	Yes	No	140/90	140/90	Not specified	Standard mercury sphygmomanometer	Manual	NR	1	Probably Hi of bias
Basit 2020	Pakistan	Yes	Yes	140/90	140/90	Not specified	Mercury sphygmomanometer	Manual	NR	1	Probably Lo of bias
McDonaldPosso 2014	Panama	No	Yes	140/90	140/90	JNC	Calibrated automatic sphygmomanometers made by American Diagnostic Corporation model 6013	Not manual	≥3	1	Probably Lo of bias

First author Year publication	Country	Hypertension definition included self-reported hypertension	Hypertension definition anti-hypertension drugs	Original Definition reported	Final definition (original or converted)	Definition used Society/ Organization criteria	Device name	Manual/ Automatic measurement	No. of measurements	No. of visits	Risk of b[illegible] outcon[illegible]
Miranda 2011	Peru	Yes	Yes	140/90	140/90	Other	NR	NR	≥3	1	Probably Hi[illegible] of bias
Bernabe-Ortiz 2017b	Peru	Yes	Yes	140/90	140/90	JNC	OMRON HEM-780 (OMRON, Tokyo, Japan)	Not manual	≥3	1	Probably Lo[illegible] of bias
Chambergo-Michilot 2021	Peru	Yes	Yes	140/90	140/90	JNC	OMRON Automatic Upper Arm Digital Blood Pressure Monitor (HEM-7113 model)	Not manual	2	1	Probably Lo[illegible] of bias
Dorobantu 2010	Romania	Yes	No	140/90	140/90	Other	Oscillometric press meters OMRONM5-I	Not manual	≥3	≥2	Probably Lo[illegible] of bias
Dorobantu 2012	Romania	No	Yes	140/90	140/90	Other	Oscillometric blood pressure (BP) measuring device model A&D UA 95 Plus	Not manual	≥3	≥2	Probably Lo[illegible] of bias
Artyukhov 2017	Russia	Yes	Yes	140/90	140/90	Not specified	Omron automated BP monitor	Not manual	2	1	Probably Lo[illegible] of bias
Balanova 2019	Russia	No	Yes	140/90	140/90	Not specified	Omron automatic tonometer	Not manual	2	1	Probably Lo[illegible] of bias
Nahimana 2017	Rwanda	No	Yes	140/90	140/90	WHO	Blood pressure machine (OMRON® digital device)	Not manual	≥3	1	Probably Lo[illegible] of bias
Seck 2014b	Senegal	Yes	Yes	140/90	140/90	JNC	Sphygmomanometer	Not manual	2	1	Probably Lo[illegible] of bias
Lovic 2013	Serbia	No	No	140/90	140/90	Other	Omron M6 Comfort automatic blood pressure monitoring devices (Omron, Kyoto, Japan)	Not manual	≥3	1	Probably Lo[illegible] of bias

First author Year publication	Country	Hypertension definition included self-reported hypertension	Hypertension definition anti-hypertension drugs	Original Definition reported	Final definition (original or converted)	Definition used Society/ Organization criteria	Device name	Manual/ Automatic measurement	No. of measurements	No. of visits	Risk of b[illegible] outcon[illegible]
Marinkovic 2014	Serbia	Yes	Yes	140/90	140/90	JNC	NR	NR	≥3	1	Probably Lo[illegible] of bias
Odland 2020	Sierra Leone	No	No	140/90	140/90	Not specified	Omron M6 AC LED	Not manual	≥3	1	Probably Lo[illegible] of bias
Geraedts 2021	Sierra Leone	No	Yes	140/90	140/90	AHA	OMRON M6 comfort machines (OMRON Healthcare, the Netherlands)	Not manual	≥3	1	Probably Lo[illegible] of bias
VanRooyen 2000	South Africa	No	No	140/90	140/90	WHO	Table-model sphygmomanometer (Model ALPK2, TycosÒ USA)	Manual	2	1	Probably Lo[illegible] of bias
vanZyl 2012	South Africa	No	No	140/90	140/90	JNC	DS-175, auto inflate electronic blood pressure monitor	Not manual	NR	1	Probably Hi[illegible] of bias
Kandala 2013	South Africa	Yes	Yes	140/90	140/90	JNC	Omron M1 electronic BP manometer (Omron Life Science, Tokyo, Japan)	Not manual	≥3	1	Probably Lo[illegible] of bias
Dolman 2014	South Africa	No	No	SBP/DBP	140/90	Not applicable	OMRON HEM- 757 apparatus	Not manual	2	1	Probably Lo[illegible] of bias
Egbujie 2016	South Africa	Yes	Yes	140/90	140/90	Not specified	NR	NR	2	1	Probably Lo[illegible] of bias
Kandala 2021	South Africa	Yes	Yes	140/90	140/90	Other	Omron digital blood pressure monitors	Not manual	≥3	1	Probably Lo[illegible] of bias
Katulanda 2014	Sri Lanka	Yes	Yes	140/90	140/90	Not specified	Omron IA2 digital BP monitors (Omron Healthcare, Alexandra Technopark, Singapore)	Not manual	2	1	Probably Lo[illegible] of bias

First author Year publication	Country	Hypertension definition included self-reported hypertension	Hypertension definition anti-hypertension drugs	Original Definition reported	Final definition (original or converted)	Definition used Society/ Organization criteria	Device name	Manual/ Automatic measurement	No. of measurements	No. of visits	Risk of b outcon
Krishnadath 2016	Suriname	No	Yes	140/90	140/90	AHA	Omron HEM-780 blood-pressure monitor	Not manual	≥3	1	Probably Lo of bias
Edwards 2000	Tanzania	Yes	Yes	140/90	140/90	WHO	Accoson mercury sphygmomanometer	Manual	2	1	Probably Lo of bias
Stanifer 2016	Tanzania	No	Yes	Single measurement of > 160/100 mmHg, a 2 time average measurement of > 140/90 mmHg.	Mixed	Not specified	Omron HEM-712 sphygmomanometer (Omron Healthcare, Inc.; Bannockburn, IL)	Not manual	2	1	Probably Lo of bias
Mosha 2017	Tanzania	No	No	140/90	140/90	JNC	Aneroid sphygmomanometer	Manual	≥3	1	Probably Lo of bias
Suriyawongpaisal 2003	Thailand	No	Yes	140/90	140/90	WHO	NR	NR	≥3	1	Probably Lo of bias
Aekplakorn 2012	Thailand	No	Yes	140/90	140/90	JNC	Microlife model A100 (Microlife AG, Widnau, Switzerland)	Not manual	≥3	1	Probably Lo of bias
Bouguerra 2006	Tunisia	No	No	130/85	140/90	ATP	Oscillometric blood pressure recorder	Not manual	1	1	Probably Hi of bias
Hammami 2011	Tunisia	No	Yes	140/90	140/90	JNC	Standard mercury sphygmomanometer	Manual	2	1	Probably Lo of bias

First author Year publication	Country	Hypertension definition included self-reported hypertension	Hypertension definition anti-hypertension drugs	Original Definition reported	Final definition (original or converted)	Definition used Society/ Organization criteria	Device name	Manual/ Automatic measurement	No. of measurements	No. of visits	Risk of b[illegible] outcon[illegible]
Aounallah-Skhiri 2012	Tunisia	No	No	15-17 years ≥ 95 percentile and 18-19 years ≥140/90 18 - 19: ≥ 90 percentile	Mixed	Other	- Stethoscope and<br>- Calibrated sphygmomanometers (Vaquez Laubry type, Spengler, France)	Manual	2	1	Probably Lo[illegible] of bias
BenRomdhane 2012	Tunisia	No	Yes	140/90	140/90	JNC	Standard mercury sphygmomanometer	Manual	2	1	Probably Lo[illegible] of bias
Sonmez 1999	Turkey	No	No	SBP/DBP	140/90	Not applicable	Aneroid sphygmomanometer	Manual	NR	1	Probably Lo[illegible] of bias
Altun 2005	Turkey	Yes	Yes	140/90	140/90	Not specified	Random zero mercury sphygmomanometers	Manual	≥3	1	Probably Lo[illegible] of bias
Metintas 2009	Turkey	Yes	Yes	140/90	140/90	WHO	NR	NR	NR	1	Probably Hi[illegible] of bias
Sengul 2016	Turkey	Yes	Yes	140/90	140/90	Not specified	Automatic oscillometric BP measuring device (Omron M3 Intelligence, HEM-7051-E; Tokyo, Japan)	Not manual	≥3	1	Probably Lo[illegible] of bias
Dastan 2017	Turkey	No	Yes	140/90	140/90	JNC	- Stethoscope, and<br>- Sphygmomanometer	Manual	1	1	Probably Lo[illegible] of bias

First author Year publication	Country	Hypertension definition included self-reported hypertension	Hypertension definition anti-hypertension drugs	Original Definition reported	Final definition (original or converted)	Definition used Society/ Organization criteria	Device name	Manual/ Automatic measurement	No. of measurements	No. of visits	Risk of b[illegible] outcon[illegible]
Oğuz 2018	Turkey	No	Yes	140/90	140/90	Not specified	Omron Deluxe Comfit Cuff (Omron Healthcare, Inc., Lake Forest, IL, USA)	Not manual	2	1	Probably Lo[illegible] of bias
Musinguzi 2013	Uganda	Yes	Yes	140/90	140/90	Not specified	Digital blood pressure monitor, model LD7	Not manual	≥3	1	Probably Lo[illegible] of bias
Guwatudde 2015	Uganda	No	Yes	140/90	140/90	Other	Digital blood pressure machine (Boso Medicus Uno1)	Not manual	≥3	1	Probably Lo[illegible] of bias
Nakibuuka 2015	Uganda	No	Yes	140/90	140/90	Other	Omron automated sphygmomanometer model HEM 907	Not manual	≥3	1	Probably Lo[illegible] of bias
Nieto-Martínez 2018	Venezuela	No	Yes	140/90	140/90	JNC	Calibrated aneroid sphygmomanometer or mercury sphygmomanometer	Manual	2	1	Probably Lo[illegible] of bias
Nguyen 2012	Vietnam	No	Yes	140/90	140/90	JNC	Digital sphygmomanometer (OMRON Healthcare Inc.H, Bannockburn, Illinois)	Not manual	2	1	Probably Lo[illegible] of bias
Do 2015	Vietnam	No	Yes	140/90	140/90	JNC	Mercury sphygmomanometer	Manual	2	1	Probably Lo[illegible] of bias
Jensen 2018	Vietnam	No	No	140/90	140/90	Not specified	Omron HEM 907 digital automatic blood pressure monitor	Not manual	≥3	1	Probably Lo[illegible] of bias

First author Year publication	Country	Hypertension definition included self-reported hypertension	Hypertension definition anti-hypertension drugs	Original Definition reported	Final definition (original or converted)	Definition used Society/ Organization criteria	Device name	Manual/ Automatic measurement	No. of measurements	No. of visits	Risk of b[illegible] outcon[illegible]
Hoang 2019	Vietnam	No	Yes	140/90	140/90	WHO	BOSO device	Not manual	≥3	1	Probably Lo[illegible] of bias
Abdul-Rahim 2001	West Bank and Gaza	No	No	140/90	140/90	WHO	NR	NR	NR	1	Probably Lo[illegible] of bias
Modesti 2013a	Yemen	No	Yes	140/90	140/90	Other	Sphygmomanometer (HEM 705 IT; Omron Matsusaka Co, Ltd, Matsusaka, Japan)	Not manual	≥3	1	Probably Lo[illegible] of bias

Sample size and demographic characteristics of the 299 surveys arranged by region and year starting collection

Region	First author year	Country	Period	Classification at data collection	Total sample size	Proportion rural and urban	Proportion of females	Mean age (whole/rural/urban)
East Asia & Pacific	Attard 2015	China	1991-1991	LIC	5,315	50.5% / 49.5%	52.5%	38.2 / 37.8 / 38.6
East Asia & Pacific	Tao 1995	China	1991-1991	LIC	950,356	52.5% / 47.5%	52.7%	40.7 / 39.3 / 42.3
East Asia & Pacific	WangZ 2004	China	1992-1994	LIC	18,746	52.6% / 47.4%	52.8%	46.7 / 46.1 / 47.5
East Asia & Pacific	Li J 2017	China	1993-1993	LIC	6,644	64.5% / 35.5%	--[1]	--[2] / --[2] / --[2]
East Asia & Pacific	Li J 2017	China	1997-1997	LMIC	7,347	63.4% / 36.6%	--[1]	--[2] / --[2] / --[2]
East Asia & Pacific	WangZ 2004	China	1998-1998	LIC	13,504	53.8% / 46.2%	52.0%	46.8 / 46.5 / 47.2
East Asia & Pacific	Li J 2017	China	2000-2000	LMIC	7,704	59.9% / 40.1%	--[1]	--[2] / --[2] / --[2]
East Asia & Pacific	Ma 2015	China	2000-2000	LMIC	2,832	45.8% / 54.2%	51.3%	72 / 71.9 / 71.7
East Asia & Pacific	Reynolds 2003	China	2000-2001	LMIC	15,540	49.2% / 50.8%	51.6%	50 / 50.2 / 49.8
East Asia & Pacific	Suriyawongpaisal 2003	Thailand	2000-2000	LMIC	5,305	41.0% / 59.0%	60.5%	53.5 / 53.5 / 53.5
East Asia & Pacific	Nguyen 2012	Vietnam	2001-2001	LIC	2,386	45.8% / 54.2%	60.6%	45 / -- / --
East Asia & Pacific	Hosey 2014	Micronesia	2002-2002	LMIC	1,638	70.8% / 29.2%	60.8%	39.7 / -- / --
East Asia & Pacific	Nguyen 2012	Vietnam	2002-2002	LIC	2,594	76.9% / 23.1%	60.6%	45 / -- / --
East Asia & Pacific	Wu 2008	China	2002-2002	LMIC	141,892	66.4% / 33.6%	--	-- / -- / --
East Asia & Pacific	Zuo 2009	China	2002-2002	LMIC	3,914	80.5% / 19.5%	55.3%	53.5 / -- / --
East Asia & Pacific	Htet 2017	Myanmar	2003-2004	LIC	4,448	--[3]	55.2%	49.2 / -- / --
East Asia & Pacific	Nguyen 2012	Vietnam	2003-2003	LIC	4,342	57.9% / 42.1%	60.0%	45.4 / -- / --
East Asia & Pacific	Prince 2012	China	2003-2006	LMIC	2,162	46.3% / 53.7%	56.3%	73.2 / 72.4 / 73.9
East Asia & Pacific	Aekplakorn 2012	Thailand	2004-2004	LMIC	41,483	44.9% / 55.1%	52.4%	42.5 / -- / --
East Asia & Pacific	Lao 2013	China	2004-2004	LMIC	7,640	61.8% / 38.2%	57.9%	43.4 / 43.4 / 43.3
East Asia & Pacific	Lewington 2016	China	2004-2009	LMIC	500,223	55.9% / 44.1%	59.0%	52 / -- / --
East Asia & Pacific	Li J 2017	China	2004-2004	LMIC	7,952	61.8% / 38.2%	--[1]	--[2] / --[2] / --[2]

Region	First author year	Country	Period	Classification at data collection	Total sample size	Proportion rural and urban	Proportion of females	Mean age (whole/rural/urban)
East Asia & Pacific	Ma 2015	China	2004-2004	LMIC	1,828	53.4% / 46.6%	51.8%	74.2 / 74.4 / 74
East Asia & Pacific	Nguyen 2012	Vietnam	2004-2004	LIC	3,316	26.8% / 73.2%	60.0%	46.8 / 45.7 / 46.8
East Asia & Pacific	Xu 2013	China	2004-2004	LMIC	7,633	61.8% / 38.2%	57.9%	43.8 / 43.9 / 43.6
East Asia & Pacific	Do 2015	Vietnam	2005-2005	LIC	17,199	76.4% / 23.6%	50.7%	44.12 / -- / --
East Asia & Pacific	Li W 2016	China	2005-2009	LMIC	45,108	50.4% / 49.6%	58.9%	51.4 / 50.2 / 52.7
East Asia & Pacific	Liu X 2017a	China	2005-2014	LMIC	2,428	34.2% / 65.8%	50.7%	52.2 / -- / --
East Asia & Pacific	Li J 2017	China	2006-2006	LMIC	7,425	67.4% / 32.6%	-- [1]	-- [2] / -- [2] / -- [2]
East Asia & Pacific	Naing 2016	Malaysia	2006-2006	UMIC	33,976	41.0% / 59.0%	55.2%	-- / -- / --
East Asia & Pacific	Nguyen 2012	Vietnam	2006-2006	LIC	2,160	89.0% / 11.0%	61.0%	46.4 / -- / --
East Asia & Pacific	Xu 2008	China	2006-2006	LMIC	3,222	46.2% / 53.8%	56.5%	60.4 / 56.9 / 63.4
East Asia & Pacific	Zhang 2008	China	2006-2006	LMIC	13,925	26.0% / 74.0%	45.9%	40 / 39.4 / 40.3
East Asia & Pacific	Zhao 2011	China	2006-2006	LMIC	3,357	53.5% / 46.5%	59.2%	50.23 / 49.1 / 51.5
East Asia & Pacific	Abdul-Razak 2016	Malaysia	2007-2011	UMIC	11,267	48.0% / 52.0%	56.3%	53.02 / -- / --
East Asia & Pacific	Basu 2013	China	2007-2010	LMIC	15,004	51.0% / 49.0%	48.0%	45 / -- / --
East Asia & Pacific	Fu 2010	China	2007-2007	LMIC	10,620	49.6% / 50.4%	55.8%	43.1 / -- / --
East Asia & Pacific	Gao 2013	China	2007-2008	LMIC	46,239	70.0% / 30.0%	60.2%	-- / -- / --
East Asia & Pacific	Hussain 2016	Indonesia	2007-2008	LMIC	9,755	50.0% / 50.0%	53.6%	54.6 / -- / --
East Asia & Pacific	Lao 2013	China	2007-2007	LMIC	6,451	61.9% / 38.1%	52.6%	44.9 / 45.7 / 43.8
East Asia & Pacific	Ma 2015	China	2007-2007	LMIC	2,277	61.0% / 39.0%	53.8%	71.5 / 70.5 / 72.9
East Asia & Pacific	Nguyen 2012	Vietnam	2007-2007	LIC	2,115	86.6% / 13.4%	61.0%	46.4 / 46 / 45
East Asia & Pacific	Wu 2016	China	2007-2011	LMIC	23,010	58.4% / 41.6%	53.1%	43 / -- / --
East Asia & Pacific	Xu 2013	China	2007-2007	LMIC	6,447	61.8% / 38.2%	52.6%	45.3 / 46.1 / 43.9
East Asia & Pacific	Aekplakorn 2012	Thailand	2008-2009	LMIC	20,426	45.8% / 54.2%	52.4%	45.4 / -- / --
East Asia & Pacific	Cai 2012	China	2008-2008	LMIC	5,551	53.6% / 46.4%	58.4%	41.5 / -- / --
East Asia & Pacific	Mohamud 2012	Malaysia	2008-2008	UMIC	4,341	49.0% / 51.0%	64.9%	47.8 / 48.3 / 47.4

Region	First author year	Country	Period	Classification at data collection	Total sample size	Proportion rural and urban	Proportion of females	Mean age (whole/rural/urban)
East Asia & Pacific	Nguyen 2012	Vietnam	2008-2009	LIC	4,292	45.3% / 54.7%	62.5%	48.2 / 48.4 / 49.9
East Asia & Pacific	Attard 2015	China	2009-2009	LMIC	5,475	50.3% / 49.7%	52.1%	47.5 / 46.8 / 48.2
East Asia & Pacific	Bjertness 2016	Myanmar	2009-2009	LIC	7,319	70.4% / 29.6%	60.9%	40.4 / -- / --
East Asia & Pacific	Jensen 2018	Vietnam	2009-2009	LMIC	2,333	67.4% / 32.6%	53.6%	37 / -- / --
East Asia & Pacific	WangJ 2014	China	2009-2010	LMIC	50,171	77.4% / 22.6%	49.8%	42.4 / -- / --
East Asia & Pacific	Pengpid 2022	Mongolia	2009-2009	LMIC	1,033	48.6% / 51.4%	60.4%	39.6 / -- / --
East Asia & Pacific	Bi 2015	China	2010-2010	UMIC	96,121	60.6% / 39.4%	54.3%	46.7 / -- / --
East Asia & Pacific	Lao 2013	China	2010-2010	UMIC	8,577	60.1% / 39.9%	51.4%	45.2 / 45.5 / 44.8
East Asia & Pacific	WangH 2013	China	2010-2010	UMIC	17,437	60.4% / 39.6%	53.2%	49.3 / 46 / 45
East Asia & Pacific	Zheng 2012	China	2010-2010	UMIC	1,003	49.4% / 50.6%	59.9%	51.2 / -- / --
East Asia & Pacific	Bi 2014	China	2011-2011	UMIC	15,350	68.7% / 31.3%	49.9%	40.7 / -- / --
East Asia & Pacific	Li J 2017	China	2011-2011	UMIC	12,338	49.3% / 50.7%	-- [1]	48.3 / 48 / 48.7
East Asia & Pacific	Naing 2016	Malaysia	2011-2011	UMIC	18,098	41.2% / 58.8%	48.0%	-- / -- / --
East Asia & Pacific	Ding 2020	China	2011-2012	UMIC	17,302	59.5% / 40.5%	51.3%	59.67 / 59.86 / 59.49
East Asia & Pacific	Fan 2014	China	2012-2012	UMIC	18,772	59.6% / 40.4%	55.1%	38.2 / -- / --
East Asia & Pacific	Huang 2017	China	2012-2013	UMIC	2,676	41.9% / 58.1%	52.7%	42.8 / -- / --
East Asia & Pacific	Liu X 2017b	China	2012-2013	UMIC	14,420	51.4% / 48.6%	50.2%	46.95 / 46.79 / 47.12
East Asia & Pacific	Wagner 2018	Cambodia	2012-2012	LIC	13,422	74.6% / 25.4%	73.4%	50.5 / -- / --
East Asia & Pacific	WangZ 2018	China	2012-2015	UMIC	451,755	51.3% / 48.7%	52.2%	43.4 / 43 / 44
East Asia & Pacific	Zhang 2016	China	2012-2012	UMIC	1,196,422	37.6% / 62.4%	53.4%	59.2 / -- / --
East Asia & Pacific	Htet 2017	Myanmar	2013-2014	LIC	1,486	-- [3]	49.9%	47.6 / -- / --
East Asia & Pacific	Hu 2016	China	2013-2013	UMIC	8,193	49.3% / 50.7%	50.2%	41 / -- / --
East Asia & Pacific	Hu 2017	China	2013-2014	UMIC	15,296	49.0% / 51.0%	59.0%	53.4 / -- / --
East Asia & Pacific	Huang 2016	China	2013-2014	UMIC	3,230	32.7% / 67.3%	53.5%	43.78 / -- / --
East Asia & Pacific	Li Q 2017	China	2013-2013	UMIC	207,323	44.5% / 55.5%	54.2%	57.72 / -- / --

Region	First author year	Country	Period	Classification at data collection	Total sample size	Proportion rural and urban	Proportion of females	Mean age (whole/rural/urban)
East Asia & Pacific	Li Y 2017	China	2013-2014	UMIC	174,621	53.8% / 46.2%	57.2%	51.6 / 51.6 / 51.6
East Asia & Pacific	Wei 2015	China	2013-2013	UMIC	3,778	42.4% / 57.6%	52.7%	46.1 / -- / --
East Asia & Pacific	Yang 2016	China	2013-2013	UMIC	19,254	49.2% / 50.8%	50.2%	45.4 / -- / --
East Asia & Pacific	Isa 2021	Malaysia	2013-2015	UMIC	3,453	53.6% / 46.4%	57.9%	50.91 / -- / --
East Asia & Pacific	Pengpid 2022	Mongolia	2013-2013	LMIC	1,178	48.6% / 51.4%	55.0%	41.4 / -- / --
East Asia & Pacific	Lu 2017	China	2014-2017	UMIC	1,738,886	61.2% / 38.8%	59.5%	55.6 / -- / --
East Asia & Pacific	WangJ 2018	China	2014-2015	UMIC	14,956	51.1% / 48.9%	53.6%	41.55 / 40.96 / 42.55
East Asia & Pacific	Han 2020	China	2014-2015	UMIC	9,225	-- [3]	60.1%	54.8 / -- / --
East Asia & Pacific	Li 2020	China	2014-2015	UMIC	14,596	61.6% / 38.4%	54.9%	-- / -- / --
East Asia & Pacific	Sujarwoto 2020	Indonesia	2014-2015	LMIC	30,451	41.3% / 58.7%	54.4%	44.98 / 46.02 / 43.91
East Asia & Pacific	Wang 2019	China	2014-2016	UMIC	47,040	71.9% / 28.1%	48.5%	44 / 43.2 / 45.9
East Asia & Pacific	Hoang 2019	Vietnam	2015-2015	LMIC	3,080	55.2% / 44.8%	57.1%	44 / -- / --
East Asia & Pacific	Ma 2021	China	2015-2015	UMIC	8,907	58.5% / 41.5%	51.3%	50.94 / -- / --
East Asia & Pacific	Naidu 2019	Malaysia	2015-2015	UMIC	19,936	-- [3]	--	-- / -- / --
East Asia & Pacific	Wei 2019	China	2015-2017	UMIC	5,132	35.2% / 64.8%	61.3%	53.04 / 54.17 / 52.42
East Asia & Pacific	ZhangFL 2017	China	2016-2016	UMIC	4,052	49.0% / 51.0%	60.0%	54.85 / -- / --
East Asia & Pacific	Du 2019	China	2016-2017	UMIC	7,512	60.1% / 39.9%	50.1%	40.66 / -- / --
East Asia & Pacific	Maharani 2019	Indonesia	2016-2016	LMIC	17,865	42.9% / 57.1%	56.5%	55 / 55.1 / 54.8
East Asia & Pacific	Ma 2020	China	2017-2017	UMIC	12,957	34.9% / 65.1%	53.5%	44.78 / -- / --
East Asia & Pacific	Xing 2019	China	2017-2019	UMIC	18,796	71.1% / 28.9%	61.0%	60.4 / 60.2 / 60.9
East Asia & Pacific	Li 2021	China	2018-2019	UMIC	8,211	43.0% / 57.0%	58.9%	64.39 / 64.05 / 64.64
East Asia & Pacific	Yu 2021	China	2018-2019	UMIC	2,426	80.2% / 19.8%	61.5%	50.68 / 51.91 / 45.66
East Asia & Pacific	Pengpid 2022	Mongolia	2019-2019	LMIC	4,110	35.0% / 65.0%	52.8%	43.3 / -- / --
East Asia & Pacific	Su 2020	China	2019-2019	UMIC	9,745,640	70.5% / 29.5%	47.8%	44.46 / -- / --
Europe & Central Asia	Sonmez 1999	Turkey	1995-1995	LMIC	1,466	33.8% / 66.2%	44.5%	47.6 / -- / --

Region	First author year	Country	Period	Classification at data collection	Total sample size	Proportion rural and urban	Proportion of females	Mean age (whole/rural/urban)
Europe & Central Asia	Altun 2005	Turkey	2003-2003	LMIC	4,910	35.0% / 65.0%	59.5%	40.5 / -- / --
Europe & Central Asia	Dorobantu 2010	Romania	2005-2005	UMIC	2,017	42.3% / 57.7%	58.0%	48.3 / -- / --
Europe & Central Asia	Harhay 2013	Armenia	2005-2005	LMIC	7,382	36.2% / 63.8%	83.7%	31.3 / -- / --
Europe & Central Asia	Harhay 2013	Azerbaijan	2006-2006	LMIC	10,637	43.5% / 56.5%	79.0%	31.7 / -- / --
Europe & Central Asia	Metintas 2009	Turkey	2006-2007	UMIC	3,000	44.0% / 56.0%	58.9%	56.3 / 57.3 / 55.4
Europe & Central Asia	Basu 2013	Russia	2007-2010	UMIC	4,355	24.0% / 76.0%	57.0%	54 / -- / --
Europe & Central Asia	Harhay 2013	Ukraine	2007-2007	LMIC	7,454	32.3% / 67.7%	68.3%	32.3 / -- / --
Europe & Central Asia	Harhay 2013	Albania	2008-2009	LMIC	6,417	53.8% / 46.2%	55.8%	31.9 / -- / --
Europe & Central Asia	Markovic 2011	Croatia	2008-2008	UMIC	2,378	26.5% / 73.5%	61.9%	-- / -- / --
Europe & Central Asia	Oğuz 2018	Turkey	2010-2010	UMIC	4,056	35.1% / 64.9%	60.7%	50 / -- / --
Europe & Central Asia	Dastan 2017	Turkey	2011-2011	UMIC	15,047	29.8% / 70.2%	53.0%	44 / 46.6 / 42.9
Europe & Central Asia	Dorobantu 2012	Romania	2011-2012	UMIC	1,975	39.5% / 60.5%	52.6%	56.7 / -- / --
Europe & Central Asia	Marinkovic 2014	Serbia	2011-2011	UMIC	1,669	34.9% / 65.1%	49.2%	-- / -- / --
Europe & Central Asia	Lovic 2013	Serbia	2012-2012	UMIC	3,878	23.8% / 76.2%	53.3%	48.89 / 54.22 / 47.22
Europe & Central Asia	Sengul 2016	Turkey	2012-2012	UMIC	5,437	25.7% / 74.3%	50.3%	42.51 / -- / --
Europe & Central Asia	Supiyev 2016	Kazakhstan	2012-2015	UMIC	953	49.8% / 50.2%	55.8%	60.7 / 60.2 / 61.2
Europe & Central Asia	Artyukhov 2017	Russia	2014-2014	UMIC	1,541	26.9% / 73.1%	60.5%	45.2 / -- / --
Europe & Central Asia	Abba 2022	Albania	2017-2018	UMIC	20,846	54.2% / 45.8%	71.3%	38.5 / -- / --
Europe & Central Asia	Balanova 2019	Russia	2017-2017	UMIC	6,714	-- [3]	55.3%	-- / -- / --
Latin America & Caribbean	Guerrero-Romero 2000	Mexico	2000-2000	UMIC	1,790	45.5% / 54.5%	70.8%	48.9 / 43.2 / 51.1
Latin America & Caribbean	Beltran-Sanchez 2011	Mexico	2002-2002	UMIC	14,280	42.3% / 57.7%	56.9%	41.6 / 43.7 / 40.9
Latin America & Caribbean	Prince 2012	Peru	2003-2006	LMIC	1,933	28.6% / 71.4%	61.2%	74.8 / 74.2 / 75
Latin America & Caribbean	Prince 2012	Mexico	2003-2006	UMIC	2,003	49.9% / 50.1%	63.3%	74.3 / 74.1 / 74.5
Latin America & Caribbean	Camacho 2016	Colombia	2005-2009	LMIC	7,448	49.7% / 50.3%	64.2%	50.8 / -- / --
Latin America & Caribbean	deSouza 2020	Brazil	2006-2006	UMIC	1,644	47.9% / 52.1%	57.8%	38.4 / 37.6 / 39.1

Region	First author year	Country	Period	Classification at data collection	Total sample size	Proportion rural and urban	Proportion of females	Mean age (whole/rural/urban)
Latin America & Caribbean	Nieto-Martínez 2018	Venezuela	2006-2010	UMIC	751	18.6% / 81.4%	69.5%	43.9 / 46 / 43.5
Latin America & Caribbean	Basu 2013	Mexico	2007-2010	UMIC	2,733	22.0% / 78.0%	52.0%	39 / -- / --
Latin America & Caribbean	Ferguson 2011	Jamaica	2007-2008	UMIC	2,848	36.0% / 64.0%	68.9%	37.6 / 38.7 / 37
Latin America & Caribbean	Laux 2012	Nicaragua	2007-2009	LMIC	855	86.0% / 14.0%	56.5%	34.6 / -- / --
Latin America & Caribbean	Miranda 2011	Peru	2007-2008	LMIC	989	20.3% / 79.7%	52.8%	48 / 48.3 / 47.9
Latin America & Caribbean	Lamelas 2019	Argentina	2010-2011	UMIC	11,140	34.9% / 65.1%	61.1%	51.78 / -- / --
Latin America & Caribbean	McDonaldPosso 2014	Panama	2010-2011	UMIC	3,406	52.6% / 47.4%	69.6%	46 / -- / --
Latin America & Caribbean	Bernabe-Ortiz 2017b	Peru	2012-2013	UMIC	2,656	38.8% / 61.2%	51.2%	55.1 / 55 / 55.1
Latin America & Caribbean	Pérez-Galarza 2021	Ecuador	2012-2012	UMIC	10,318	36.4% / 63.6%	64.3%	33.6 / -- / --
Latin America & Caribbean	Almeida 2015	Brazil	2013-2013	UMIC	1,410	34.7% / 65.3%	56.1%	50.55 / 50.52 / 50.58
Latin America & Caribbean	dePaula 2015	Brazil	2013-2013	UMIC	435	21.4% / 78.6%	61.1%	72 / 71 / 72.3
Latin America & Caribbean	Krishnadath 2016	Suriname	2013-2013	UMIC	5,536	29.8% / 70.2%	51.3%	35 / -- / --
Latin America & Caribbean	Malta 2018	Brazil	2013-2013	UMIC	59,402	13.7% / 86.2%	56.4%	-- / -- / --
Latin America & Caribbean	DeGennaroJr 2018	Haiti	2015-2016	LIC	2,131	33.1% / 66.9%	61.1%	40.8 / 42.4 / 39.9
Latin America & Caribbean	deSouza 2020	Brazil	2015-2016	UMIC	1,201	27.5% / 72.5%	63.2%	41.9 / 41.5 / 42.1
Latin America & Caribbean	Orantes-Navarro 2019	El Salvador	2015-2015	LMIC	4,817	47.0% / 53.0%	64.6%	44.9 / -- / --
Latin America & Caribbean	Santiago 2019	Brazil	2015-2015	UMIC	416	42.1% / 57.9%	64.9%	35 / -- / --
Latin America & Caribbean	Chambergo-Michilot 2021	Peru	2018-2018	UMIC	33,336	34.7% / 65.3%	56.6%	38.7 / -- / --
Latin America & Caribbean	Felix 2020	Ecuador	2018-2018	UMIC	2,020	41.4% / 58.6%	72.2%	51.4 / -- / --
Middle East & North Africa	SarrafZadegan 1997	Iran	1993-1994	LMIC	6,532	9.4% / 90.6%	58.9%	-- / -- / --
Middle East & North Africa	Abdul-Rahim 2001	West Bank and Gaza	1996-1998	LMIC	992	50.4% / 49.6%	59.8%	44 / 43.8 / 44.1
Middle East & North Africa	Bouguerra 2006	Tunisia	1996-1997	LMIC	3,857	41.8% / 58.2%	67.1%	44.9 / 44.7 / 45.1
Middle East & North Africa	Shirani 2011	Iran	2000-2000	LMIC	12,014	28.5% / 71.5%	49.0%	39.3 / 39.5 / 39.2
Middle East & North Africa	Tazi 2003	Morocco	2000-2000	LMIC	1,802	49.6% / 50.4%	58.1%	44.2 / -- / --

Region	First author year	Country	Period	Classification at data collection	Total sample size	Proportion rural and urban	Proportion of females	Mean age (whole/rural/urban)
Middle East & North Africa	Azimi-Nezhad 2009a	Iran	2004-2004	LMIC	4,928	39.0% / 61.0%	49.6%	40.2 / -- / --
Middle East & North Africa	BenRomdhane 2012	Tunisia	2004-2005	LMIC	8,007	42.1% / 57.9%	57.3%	49.6 / -- / --
Middle East & North Africa	Janghorbani 2008	Iran	2004-2005	LMIC	69,722	35.3% / 64.7%	49.7%	44.1 / -- / --
Middle East & North Africa	Malekzadeh 2013	Iran	2004-2008	LMIC	50,045	80.0% / 20.0%	57.6%	51.8 / -- / --
Middle East & North Africa	Aounallah-Skhiri 2012	Tunisia	2005-2005	LMIC	2,870	46.6% / 53.4%	54.9%	16.9 / -- / --
Middle East & North Africa	Esteghamati 2016	Iran	2005-2005	LMIC	68,850	--[3]	--	43.6 / -- / --
Middle East & North Africa	Ebrahimi 2010	Iran	2006-2006	LMIC	29,971	38.0% / 62.0%	50.1%	38.8 / -- / --
Middle East & North Africa	Esteghamati 2016	Iran	2007-2007	LMIC	4,184	--[3]	--	-- / -- / --
Middle East & North Africa	Hammami 2011	Tunisia	2008-2009	LMIC	598	13.7% / 86.3%	66.2%	72.3 / -- / --
Middle East & North Africa	Modesti 2013a	Yemen	2008-2008	LIC	10,242	50.1% / 49.9%	50.6%	40.3 / 40.1 / 40.5
Middle East & North Africa	Ziyyat 2014	Morocco	2008-2008	LMIC	1,628	24.8% / 75.2%	63.4%	54.2 / -- / --
Middle East & North Africa	Katibeh 2020	Iran	2010-2011	UMIC	2,098	10.8% / 89.2%	52.6%	54.1 / -- / --
Middle East & North Africa	Khorrami 2017	Iran	2011-2011	UMIC	5,998	33.8% / 66.2%	59.6%	43 / 42.7 / 43.14
Middle East & North Africa	Naghipour 2021	Iran	2014-2017	UMIC	10,520	56.2% / 43.8%	53.5%	51.5 / -- / --
Middle East & North Africa	Rajati 2019	Iran	2014-2017	UMIC	10,040	40.7% / 59.3%	52.5%	48.2 / -- / --
Middle East & North Africa	Rezaianzadeh 2021	Iran	2014-2017	UMIC	10,663	58.6% / 41.4%	55.7%	51.9 / -- / --
Middle East & North Africa	Fahs 2017	Lebanon	2015-2015	UMIC	1,000	66.7% / 33.3%	49.9%	54.84 / 55.05 / 54.41
Middle East & North Africa	Ahmadi 2021	Iran	2016-2019	UMIC	10,075	30.2% / 69.8%	52.8%	49.78 / 50.31 / 49.55
Middle East & North Africa	Pengpid 2020	Morocco	2017-2017	LMIC	4,555	39.6% / 60.4%	65.0%	40 / -- / --
Middle East & North Africa	Allameh 2022	Iran	2019-2020	UMIC	4,112	25.7% / 74.3%	50.0%	42.4 / -- / --
Middle East & North Africa	Jalali 2021	Iran	2019-2020	UMIC	220,241	50.0% / 50.0%	61.1%	49.82 / -- / --
South Asia	Jafar 2003	Pakistan	1990-1994	LIC	8,276	61.4% / 38.6%	52.5%	36.2 / -- / --
South Asia	Oommen 2016b	India	1991-1994	LIC	7,342	63.9% / 36.1%	56.3%	43.1 / 43.2 / 42.9
South Asia	Prabhakaran 2017	India	1991-1994	LIC	5,510	44.8% / 55.2%	54.3%	47.4 / 47.2 / 47.5
South Asia	Gupta 2009	India	1992-1995	LIC	4,712	58.6% / 41.4%	36.5%	36.3 / 36 / 36.8

Region	First author year	Country	Period	Classification at data collection	Total sample size	Proportion rural and urban	Proportion of females	Mean age (whole/rural/urban)
South Asia	Kumar 2006	India	1995-2000	LIC	2,200	54.0% / 46.0%	53.5%	49.4 / 49.5 / 49.3
South Asia	Kusuma 2004	India	1995-1996	LIC	1,316	67.5% / 32.5%	50.9%	42.1 / 41.1 / 43.1
South Asia	Sayeed 2002	Bangladesh	1995-1996	LIC	1,760	40.8% / 59.2%	37.7%	41.9 / -- / --
South Asia	Singh 1997a	India	1995-1995	LIC	3,575	49.5% / 50.5%	49.7%	40.4 / 40.2 / 40.6
South Asia	Samuel 2012	India	1998-2002	LIC	2,218	55.0% / 45.0%	47.7%	28.3 / 28 / 28.3
South Asia	Quasem 2001	India	1999-2000	LIC	723	33.2% / 66.8%	51.0%	69.7 / 70.6 / 69.3
South Asia	Quasem 2001	Bangladesh	1999-2000	LIC	480	50.0% / 50.0%	49.8%	70 / 71.3 / 68.7
South Asia	Allender 2010	India	2003-2004	LIC	3,205	19.9% / 80.1%	51.6%	39.2 / 38.9 / 39.4
South Asia	Gupta 2017	India	2003-2009	LIC	28,747	53.1% / 46.9%	56.1%	48.6 / -- / --
South Asia	Gupta 2017	Bangladesh	2003-2009	LIC	2,934	53.0% / 47.0%	54.6%	45.96 / -- / --
South Asia	Gupta 2017	Pakistan	2003-2009	LIC	1,742	43.7% / 56.3%	52.6%	47.58 / -- / --
South Asia	Midha 2009	India	2003-2004	LIC	800	50.0% / 50.0%	55.6%	34.2 / -- / --
South Asia	Prince 2012	India	2003-2006	LIC	2,004	49.9% / 50.1%	55.7%	71.9 / 72.6 / 71.3
South Asia	Hussain 2005	Bangladesh	2004-2004	LIC	6,312	75.4% / 24.6%	56.1%	35 / 37.5 / 33.5
South Asia	Farag 2014	India	2005-2007	LIC	5,928	48.8% / 51.2%	45.0%	45.3 / -- / --
South Asia	Katulanda 2014	Sri Lanka	2005-2006	LMIC	4,485	78.7% / 21.3%	60.5%	46.1 / -- / --
South Asia	Millett 2013	India	2005-2007	LIC	3,902	35.0% / 65.0%	12.8%	42.2 / 40.3 / 43.2
South Asia	Thankappan 2010	India	2005-2006	LIC	7,449	33.7% / 66.3%	51.1%	36.5 / 39.6 / 34.9
South Asia	Kusuma 2008	India	2006-2006	LIC	803	59.9% / 40.1%	51.7%	41.4 / 38.2 / 44.6
South Asia	Tareen 2011	Pakistan	2006-2009	LIC	2,495	56.8% / 43.2%	49.1%	50.3 / 51 / 49
South Asia	Nanditha 2021	India	2006-2006	LIC	4,776	54.1% / 45.9%	51.2%	38.1 / 38 / 38.2
South Asia	Basu 2013	India	2007-2010	LMIC	12,198	74.0% / 26.0%	50.0%	40 / -- / --
South Asia	Norboo 2015	India	2007-2011	LMIC	2,800	64.2% / 35.8%	55.7%	53.8 / 54.9 / 51.9
South Asia	Bhagyalaxmi 2013	India	2008-2008	LMIC	3,489	48.3% / 51.7%	48.8%	37.4 / 37.5 / 37.8
South Asia	Bhansali 2015	India	2008-2010	LMIC	13,800	70.6% / 29.4%	49.7%	40 / 40.1 / 40

Region	First author year	Country	Period	Classification at data collection	Total sample size	Proportion rural and urban	Proportion of females	Mean age (whole/rural/urban)
South Asia	Das 2011	India	2008-2010	LMIC	448	50.0% / 50.0%	42.6%	49.9 / 48.2 / 51.7
South Asia	Bharati 2012	India	2009-2009	LMIC	856	47.9% / 52.1%	62.6%	46.8 / -- / --
South Asia	Rahman 2017	Bangladesh	2009-2010	LIC	9,275	50.1% / 49.9%	53.5%	42.6 / 43.6 / 41.7
South Asia	Oommen 2016a	India	2010-2012	LMIC	5,275	62.9% / 37.1%	57.4%	45.6 / 45.9 / 45.1
South Asia	Prabhakaran 2017	India	2010-2012	LMIC	3,940	48.6% / 51.4%	51.6%	46.8 / 46.8 / 46.9
South Asia	Biswas 2016	Bangladesh	2011-2011	LIC	8,835	66.1% / 33.9%	48.8%	51.3 / -- / --
South Asia	Krishnan 2016	India	2011-2011	LMIC	5,153	56.5% / 43.5%	60.0%	51.1 / 50.2 / 52.2
South Asia	Geldsetzer 2018	India	2012-2014	LMIC	1,320,510	67.5% / 32.5%	53.1%	41.1 / 40.6 / 41.3
South Asia	Aryal 2015	Nepal	2013-2013	LIC	4,143	81.2% / 18.8%	67.8%	41.3 / -- / --
South Asia	Kanungo 2017	India	2013-2014	LMIC	18,028	60.0% / 40.0%	63.5%	38.5 / -- / --
South Asia	Zaman 2015	Bangladesh	2013-2013	LIC	4,073	71.5% / 28.5%	55.5%	42.9 / -- / --
South Asia	Bandela 2017	India	2014-2014	LMIC	1,032	66.7% / 33.3%	43.3%	36.5 / 36.4 / 36.9
South Asia	Bhadoria 2014	India	2014-2014	LMIC	911	66.1% / 33.9%	46.0%	50.4 / 50.1 / 50.9
South Asia	Tripathy 2017	India	2014-2015	LMIC	5,055	60.6% / 39.4%	53.1%	40.2 / -- / --
South Asia	Islam 2018	Bangladesh	2015-2015	LMIC	1,843	61.2% / 38.8%	51.6%	40.5 / 41.4 / 39.1
South Asia	Khanam 2021	Bangladesh	2015-2016	LMIC	12,863	72.6% / 27.4%	56.8%	-- / -- / --
South Asia	Kumar 2021	India	2015-2016	LMIC	811,808	70.4% / 29.6%	86.2%	30.5 / -- / --
South Asia	Mehata 2018	Nepal	2016-2016	LIC	13,598	38.2% / 61.8%	58.9%	36.9 / -- / --
South Asia	Basit 2020	Pakistan	2016-2017	LMIC	9,594	63.7% / 36.3%	56.3%	43.9 / -- / --
South Asia	Nanditha 2021	India	2016-2016	LMIC	6,318	39.1% / 60.9%	53.7%	41.2 / 40.4 / 41.7
South Asia	Sarma 2019	India	2016-2017	LMIC	12,012	50.7% / 49.3%	62.7%	42.52 / -- / --
South Asia	Mohanty 2020	India	2017-2017	LMIC	70,031	48.4% / 51.6%	51.9%	41.3 / 41.1 / 41.6
South Asia	Mohanty 2021	India	2017-2018	LMIC	64,427	65.4% / 34.6%	57.9%	57 / -- / --
South Asia	Riaz 2020	Bangladesh	2017-2018	LMIC	8,185	51.1% / 48.9%	53.5%	39.1 / 39.2 / 38.9
South Asia	Hanif 2021	Bangladesh	2018-2019	LMIC	4,813	71.9% / 28.0%	48.4%	65.8 / -- / --

Region	First author year	Country	Period	Classification at data collection	Total sample size	Proportion rural and urban	Proportion of females	Mean age (whole/rural/urban)
South Asia	Hasan 2021	Bangladesh	2018-2018	LMIC	4,856	51.8% / 48.2%	50.1%	46.2 / 47.6 / 44.7
South Asia	Kokane 2020	India	2018-2019	LMIC	4,985	--[3]	--	40.4 / -- / --
South Asia	Paul 2021	Bangladesh	2018-2018	LMIC	529	43.9% / 56.1%	43.7%	60 / -- / --
South Asia	Patel 2021	India	2019-2019	LMIC	38,694	69.4% / 30.6%	86.2%	-- / -- / --
South Asia	Sivanantham 2021	India	2019-2020	LMIC	2,415	30.4% / 69.6%	55.0%	44.3 / -- / --
Sub-Saharan Africa	Cooper 1997	Cameroon	1991-1994	LMIC	2,828	51.9% / 48.1%	52.0%	42.7 / 44.6 / 40.7
Sub-Saharan Africa	Okosun	Nigeria	1991-1995	LIC	1,934	54.2% / 45.8%	54.7%	40.7 / 43.5 / 37.5
Sub-Saharan Africa	Fezeu 2010	Cameroon	1994-1994	LIC	1,762	40.7% / 59.3%	57.0%	41 / 46 / 37.6
Sub-Saharan Africa	Ezenwaka 1997	Nigeria	1995-1995	LIC	500	52.0% / 48.0%	41.0%	60.8 / 62.7 / 60.1
Sub-Saharan Africa	Edwards 2000	Tanzania	1996-1997	LIC	1,689	54.9% / 45.1%	56.9%	36.92 / 42.1 / 30.6
Sub-Saharan Africa	VanDerSande 2000	Gambia	1996-1997	LIC	5,389	59.8% / 40.2%	58.7%	35.4 / 37.7 / 32.8
Sub-Saharan Africa	VanRooyen 2000	South Africa	1996-1997	UMIC	1,783	42.6% / 57.4%	57.4%	36.1 / -- / --
Sub-Saharan Africa	Kandala 2013	South Africa	1998-1998	LMIC	13,596	44.0% / 56.0%	58.2%	38.5 / -- / --
Sub-Saharan Africa	Sobngwi 2002	Cameroon	1998-1998	LIC	2,465	52.0% / 48.0%	57.5%	37.1 / 46.8 / 26.6
Sub-Saharan Africa	Longo-Mbenza 2008	Democratic Republic of the Congo	2001-2001	LIC	1,952	--[3]	60.7%	-- / -- / --
Sub-Saharan Africa	Kodaman 2016	Ghana	2002-2008	LIC	3,317	31.7% / 68.3%	56.6%	43 / 44.3 / 42.4
Sub-Saharan Africa	Balde 2007	Guinea	2003-2003	LIC	1,537	42.4% / 57.6%	52.5%	49.4 / 45.6 / 52.2
Sub-Saharan Africa	Fezeu 2010	Cameroon	2003-2003	LIC	1,398	29.7% / 70.3%	56.4%	40.9 / 47.3 / 38.3
Sub-Saharan Africa	Agyemang 2006	Ghana	2004-2004	LIC	1,431	40.4% / 59.6%	55.0%	35.9 / 39 / 34.4
Sub-Saharan Africa	Mufunda 2006	Eritrea	2004-2004	LIC	2,352	74.4% / 25.6%	50.3%	55 / -- / --
Sub-Saharan Africa	Damasceno 2009	Mozambique	2005-2005	LIC	3,081	49.9% / 50.1%	58.4%	40.8 / -- / --
Sub-Saharan Africa	Dolman 2014	South Africa	2005-2005	UMIC	1,710	52.7% / 47.3%	62.5%	48 / 46.7 / 48.7
Sub-Saharan Africa	Murthy 2013	Nigeria	2005-2007	LIC	13,504	77.6% / 22.4%	54.1%	55.9 / -- / --
Sub-Saharan Africa	Delisle 2012	Benin	2006-2006	LIC	541	31.4% / 68.6%	49.9%	38.19 / 36.3 / 38.06

Region	First author year	Country	Period	Classification at data collection	Total sample size	Proportion rural and urban	Proportion of females	Mean age (whole/rural/urban)
Sub-Saharan Africa	Basu 2013	Ghana	2007-2010	LIC	5,563	54.0% / 46.0%	50.0%	43 / -- / --
Sub-Saharan Africa	Basu 2013	South Africa	2007-2010	UMIC	4,223	31.0% / 69.0%	53.0%	42 / -- / --
Sub-Saharan Africa	Ejim 2013	Nigeria	2007-2008	LIC	543	56.7% / 43.3%	68.7%	56.3 / 59.9 / 51.4
Sub-Saharan Africa	Mathenge 2010	Kenya	2007-2008	LIC	4,376	67.3% / 32.7%	52.0%	63.5 / 65.4 / 61.6
Sub-Saharan Africa	vanZyl 2012	South Africa	2007-2009	UMIC	976	57.7% / 42.3%	73.0%	45 / 46.8 / 42.5
Sub-Saharan Africa	Giday 2011	Ethiopia	2008-2008	LIC	979	50.5% / 49.5%	47.0%	32.9 / -- / --
Sub-Saharan Africa	Houehanou 2015	Benin	2008-2008	LIC	6,762	66.4% / 33.6%	49.5%	43 / -- / --
Sub-Saharan Africa	Muluneh AT 2012	Ethiopia	2008-2009	LIC	3,223	83.2% / 16.8%	52.2%	41.1 / -- / --
Sub-Saharan Africa	Camara 2016	Guinea	2009-2009	LIC	2,491	32.0% / 68.0%	54.2%	34.2 / 33.3 / 29.4
Sub-Saharan Africa	Egbujie 2016	South Africa	2009-2009	UMIC	1,311	56.6% / 43.4%	77.2%	49.83 / 50.11 / 49.33
Sub-Saharan Africa	Isezuo SA 2011	Nigeria	2009-2010	LMIC	782	50.3% / 49.7%	47.7%	38.9 / -- / --
Sub-Saharan Africa	Lissock 2011	Cameroon	2009-2009	LMIC	452	26.5% / 73.5%	63.5%	48.8 / 50.1 / 48.3
Sub-Saharan Africa	Msyamboza 2011	Malawi	2009-2009	LIC	3,910	89.6% / 10.4%	69.7%	39.7 / -- / --
Sub-Saharan Africa	Abegunde 2013	Nigeria	2010-2011	LMIC	600	49.5% / 50.5%	64.2%	71.5 / 70.8 / 72.2
Sub-Saharan Africa	Adediran 2013	Nigeria	2010-2010	LMIC	667	49.8% / 50.2%	49.6%	43.1 / 43.31 / 43.01
Sub-Saharan Africa	Cham 2018	Gambia	2010-2010	LIC	3,219	44.9% / 55.1%	54.5%	38 / -- / --
Sub-Saharan Africa	Mengistu 2014	Ethiopia	2010-2011	LIC	1,183	41.1% / 58.9%	62.6%	34 / 33.7 / 34.3
Sub-Saharan Africa	Okpechi 2013	Nigeria	2011-2012	LMIC	2,983	53.2% / 46.8%	52.1%	41.7 / 43.9 / 39.3
Sub-Saharan Africa	Abebe 2015	Ethiopia	2012-2012	LIC	2,141	51.0% / 49.0%	54.2%	49.2 / 48.7 / 49.7
Sub-Saharan Africa	Agyemang 2018	Ghana	2012-2015	LMIC	2,492	41.9% / 58.1%	67.1%	45.8 / 46.5 / 45.2
Sub-Saharan Africa	Kavishe 2015	Tanzania	2012-2013	LIC	1,095	52.6% / 47.4%	53.9%	34 / 35.7 / 32.1
Sub-Saharan Africa	Kavishe 2015	Uganda	2012-2013	LIC	916	47.2% / 52.8%	59.1%	34.5 / 36.8 / 32.5
Sub-Saharan Africa	Mosha 2017	Tanzania	2012-2013	LIC	9,678	63.0% / 37.0%	64.9%	34.6 / -- / --
Sub-Saharan Africa	Musinguzi 2013	Uganda	2012-2012	LIC	4,563	66.7% / 33.3%	64.4%	34.9 / -- / --
Sub-Saharan Africa	Nahimana 2017	Rwanda	2012-2013	LIC	6,524	85.5% / 14.5%	62.6%	35.3 / -- / --

Region	First author year	Country	Period	Classification at data collection	Total sample size	Proportion rural and urban	Proportion of females	Mean age (whole/rural/urban)
Sub-Saharan Africa	Nakibuuka 2015	Uganda	2012-2013	LIC	5,193	26.9% / 73.1%	69.5%	34.5 / 40.1 / 32.7
Sub-Saharan Africa	Seck 2014b	Senegal	2012-2012	LMIC	1,036	44.2% / 55.8%	60.0%	48 / 43.5 / 51.6
Sub-Saharan Africa	Kandala 2021	South Africa	2012-2012	UMIC	6,867	40.0% / 60.0%	65.6%	37.7 / -- / --
Sub-Saharan Africa	Katchunga 2019	Democratic Republic of the Congo	2012-2012	LIC	5,580	73.5% / 26.5%	56.9%	35.7 / -- / --
Sub-Saharan Africa	BA 2018	Mali	2013-2013	LIC	2,102	26.6% / 73.4%	60.2%	47.78 / 47 / 48.03
Sub-Saharan Africa	Obirikorang 2015	Ghana	2013-2013	LMIC	672	53.6% / 46.4%	53.6%	46 / 46.1 / 45.9
Sub-Saharan Africa	Price 2018	Malawi	2013-2016	LIC	28,891	48.0% / 52.0%	61.7%	35.8 / 38.5 / 33
Sub-Saharan Africa	Ratovoson 2015	Madagascar	2013-2014	LIC	7,631	47.5% / 52.5%	55.0%	36 / 35.8 / 36.1
Sub-Saharan Africa	Soubeiga 2017	Burkina Faso	2013-2013	LIC	4,629	77.8% / 22.2%	51.8%	39.6 / 40.1 / 39.4
Sub-Saharan Africa	Craig 2018	Namibia	2013-2013	UMIC	3,068	53.5% / 46.5%	58.5%	46.8 / -- / --
Sub-Saharan Africa	Guwatudde 2015	Uganda	2014-2014	LIC	3,906	72.9% / 27.1%	59.8%	35.3 / -- / --
Sub-Saharan Africa	Jessen 2018	Mozambique	2014-2015	LIC	2,965	52.5% / 47.5%	61.2%	35.3 / -- / --
Sub-Saharan Africa	Kaze 2015	Cameroon	2014-2014	LMIC	439	72.9% / 27.1%	57.9%	47 / 51 / 36.5
Sub-Saharan Africa	Lemogoum 2018	Cameroon	2014-2015	LMIC	889	48.1% / 51.9%	41.5%	39 / 39 / 38
Sub-Saharan Africa	Oguoma 2015	Nigeria	2014-2014	LMIC	422	77.3% / 22.7%	64.7%	40 / 38.5 / 45.1
Sub-Saharan Africa	Stanifer 2016	Tanzania	2014-2014	LIC	481	23.1% / 76.9%	74.4%	46.9 / 48.1 / 46.1
Sub-Saharan Africa	Sanuade 2018	Ghana	2014-2014	LMIC	13,247	51.5% / 48.5%	70.9%	29.6 / -- / --
Sub-Saharan Africa	Gebreyes 2018	Ethiopia	2015-2015	LIC	9,788	88.5% / 11.5%	59.4%	-- / -- / --
Sub-Saharan Africa	Tesfaye 2019	Ethiopia	2015-2015	LIC	1,405	50.8% / 49.2%	56.7%	36.99 / -- / --
Sub-Saharan Africa	Umuerri 2020	Nigeria	2015-2015	LMIC	852	44.2% / 55.8%	55.9%	42.64 / 45.12 / 39.6
Sub-Saharan Africa	Walekhwa 2021	Kenya	2015-2015	LMIC	4,352	51.3% / 48.7%	58.8%	37.82 / -- / --
Sub-Saharan Africa	Colette 2020	Benin	2016-2016	LIC	540	75.2% / 24.8%	46.9%	40 / -- / --
Sub-Saharan Africa	Kandala 2021	South Africa	2016-2016	UMIC	8,230	47.5% / 52.5%	60.6%	39.5 / -- / --
Sub-Saharan Africa	Katchunga 2019	Democratic Republic of the Congo	2016-2016	LIC	5,286	62.2% / 37.8%	57.6%	36.4 / -- / --

Region	First author year	Country	Period	Classification at data collection	Total sample size	Proportion rural and urban	Proportion of females	Mean age (whole/rural/urban)
Sub-Saharan Africa	Masimango 2020	Democratic Republic of the Congo	2016-2017	LIC	1,317	55.4% / 44.6%	60.9%	41.1 / 44.6 / 36.7
Sub-Saharan Africa	Mika 2020	Mozambique	2016-2017	LIC	4,101	72.2% / 27.4%	59.9%	36.7 / -- / --
Sub-Saharan Africa	Odili 2020	Nigeria	2017-2018	LMIC	4,192	51.9% / 48.1%	56.7%	46.7 / -- / --
Sub-Saharan Africa	Odland 2020	Sierra Leone	2018-2018	LIC	2,071	62.9% / 37.1%	49.0%	51 / -- / --
Sub-Saharan Africa	Geraedts 2021	Sierra Leone	2019-2020	LIC	1,956	67.1% / 32.9%	55.6%	39 / -- / --
LIC	Chow 2013	LIC	2003-2009	LIC	31,685	52.7% / 47.3%	56.5%	48.55 / -- / --
LMIC	Chow 2013	LMIC	2003-2009	LMIC	58,476	50.2% / 49.8%	58.8%	50.68 / -- / --
UMIC	Chow 2013	UMIC	2003-2009	UMIC	36,463	46.8% / 53.2%	60.1%	51.19 / -- / --
All LMIC	Chow 2013	All LMIC	2003-2009	All LMIC	126,624	49.9% / 50.1%	58.6%	50.3 / -- / --

LIC: low-income country, LMIC: lower-middle-income country, UMIC: upper-middle-income country. Data not available represented as --.
[1] – The overall proportion of females across all waves was 53%.
[2] – The mean ages across all waves were 47.59 years for whole, 46.57 years for rural and 49.11 years for urban.
[3] – For these studies, we have data for urban and rural prevalence and its uncertainty (95% CI, se, etc), but we were not able to get urban and rural crude numbers.

Blood pressure characteristics of the 299 surveys arranged by region and year starting collection

Region	First author year	Country	Total sample size	Crude prevalence of hypertension rural/urban	Weighted prevalence of hypertension rural/urban	Hypertension prevalence weighted by	SBP/DBP rural	SBP/DBP urban	SBP/DBP prevalence weighted by
East Asia & Pacific	Attard 2015	China	5,315	8.1% / 18.5%	--	No	109 / 71.9	113.8 / 75	No
East Asia & Pacific	Tao 1995	China	950,356	11.1% / 16.3%	10.2% / 12.8%	Age standardized	-- / --	-- / --	--
East Asia & Pacific	WangZ 2004	China	18,746	--	22.1% / 23.1%	Age standardized	-- / --	-- / --	--
East Asia & Pacific	Li J 2017	China	6,644	12.4% / 20.1%	--	No	-- / --	-- / --	--
East Asia & Pacific	Li J 2017	China	7,347	17.2% / 24.6%	--	No	-- / --	-- / --	--
East Asia & Pacific	WangZ 2004	China	13,504	--	22.8% / 25.4%	Age standardized	-- / --	-- / --	--
East Asia & Pacific	Li J 2017	China	7,704	18.4% / 25.3%	--	No	-- / --	-- / --	--
East Asia & Pacific	Ma 2015	China	2,832	70.6% / 68.0%	--	No	-- / --	-- / --	--
East Asia & Pacific	Reynolds 2003	China	15,540	--	28.1% / 29.0%	Both	125.9 / 79.2	124.6 / 79	Both
East Asia & Pacific	Suriyawongpaisal 2003	Thailand	5,305	--	18.0% / 26.0%	Sampling weights	119 / 75	122 / 78	Sampling weig
East Asia & Pacific	Nguyen 2012	Vietnam	2,386	--	10.5% / 18.0%	Age standardized	118.13 / 73.57	118.57 / 75.95	Age standardi
East Asia & Pacific	Hosey 2014	Micronesia	1,638	--	19.2% / 20.1%	Both	124 / 75.3	124.7 / 75.3	Both
East Asia & Pacific	Nguyen 2012	Vietnam	2,594	--	8.7% / 15.8%	Age standardized	116.32 / 72.75	118.88 / 74.19	Age standardi
East Asia & Pacific	Wu 2008	China	141,892	--	17.3% / 21.1%	Age standardized	-- / --	-- / --	--
East Asia & Pacific	Zuo 2009	China	3,914	29.9% / 25.1%	--	No	-- / --	-- / --	--
East Asia & Pacific	Htet 2017	Myanmar	4,448	--	24.3% / 27.6%	Age standardized	123.7 / 75.4	122.2 / 76.6	Age standardi
East Asia & Pacific	Nguyen 2012	Vietnam	4,342	--	16.2% / 18.4%	Age standardized	119.2 / 76.54	118.98 / 75.36	Age standardi
East Asia & Pacific	Prince 2012	China	2,162	56.9% / 63.5%	--	No	137.6 / 86.8	136.7 / 76.3	No
East Asia & Pacific	Aekplakorn 2012	Thailand	41,483	35.7% / 40.1%	21.2% / 24.5%	Both	118.1 / 76.1	119.1 / 76.7	Both
East Asia & Pacific	Lao 2013	China	7,640	--	11.8% / 13.7%	Both	121 / 76	121 / 76	Both
East Asia & Pacific	Lewington 2016	China	500,223	33.4% / 31.3%	34.3% / 30.3%	Age standardized	-- / --	-- / --	--

Region	First author year	Country	Total sample size	Crude prevalence of hypertension rural/urban	Weighted prevalence of hypertension rural/urban	Hypertension prevalence weighted by	SBP/DBP rural	SBP/DBP urban	SBP/DBP prevalence weighted by
East Asia & Pacific	Li J 2017	China	7,952	21.2% / 28.4%	--	No	-- / --	-- / --	--
East Asia & Pacific	Ma 2015	China	1,828	62.7% / 60.9%	--	No	-- / --	-- / --	--
East Asia & Pacific	Nguyen 2012	Vietnam	3,316	--	10.0% / 22.6%	Age standardized	115.8 / 73.58	120.92 / 77.9	Age standardi
East Asia & Pacific	Xu 2013	China	7,633	--	12.0% / 12.0%	Age standardized	-- / --	-- / --	--
East Asia & Pacific	Do 2015	Vietnam	17,199	--	20.5% / 20.3%	Sampling weights	-- / --	-- / --	--
East Asia & Pacific	Li W 2016	China	45,108	--	41.9% / 38.4%	Age standardized	134.6 / 83.2	130.3 / 80.8	No
East Asia & Pacific	Liu X 2017a	China	2,428	17.1% / 26.7%	--	No	-- / --	-- / --	--
East Asia & Pacific	Li J 2017	China	7,425	21.9% / 24.9%	--	No	-- / --	-- / --	--
East Asia & Pacific	Naing 2016	Malaysia	33,976	36.9% / 29.3%	--	No	-- / --	-- / --	--
East Asia & Pacific	Nguyen 2012	Vietnam	2,160	--	23.2% / 22.6%	Age standardized	127.04 / 78.1	126.94 / 75.98	Age standardi
East Asia & Pacific	Xu 2008	China	3,222	49.9% / 43.7%	--	No	-- / --	-- / --	--
East Asia & Pacific	Zhang 2008	China	13,925	--	34.5% / 28.3%	Both	-- / --	-- / --	--
East Asia & Pacific	Zhao 2011	China	3,357	43.5% / 36.6%	--	No	135.1 / 85.6	129.9 / 84.6	Age standardi
East Asia & Pacific	Abdul-Razak 2016	Malaysia	11,267	51.2% / 44.9%	--	No	-- / --	-- / --	No
East Asia & Pacific	Basu 2013	China	15,004	44.3% / 33.0%	--	No	116.6 / --	118.4 / --	No
East Asia & Pacific	Fu 2010	China	10,620	29.6% / 31.3%	--	No	-- / --	-- / --	--
East Asia & Pacific	Gao 2013	China	46,239	--	25.2% / 28.1%	Both	-- / --	-- / --	--
East Asia & Pacific	Hussain 2016	Indonesia	9,755	--	46.4% / 49.8%	Age standardized	-- / --	-- / --	--
East Asia & Pacific	Lao 2013	China	6,451	--	17.0% / 13.7%	Both	127 / 79	125 / 78	Both
East Asia & Pacific	Ma 2015	China	2,277	54.4% / 58.6%	--	No	-- / --	-- / --	--
East Asia & Pacific	Nguyen 2012	Vietnam	2,115	--	24.3% / 35.0%	Age standardized	124.5 / 78.14	127.45 / 80.01	Age standardi
East Asia & Pacific	Wu 2016	China	23,010	--	27.1% / 20.3%	Age standardized	-- / --	-- / --	--
East Asia & Pacific	Xu 2013	China	6,447	--	18.9% / 10.6%	Age standardized	-- / --	-- / --	--
East Asia & Pacific	Aekplakorn 2012	Thailand	20,426	29.5% / 35.9%	19.0% / 26.7%	Both	-- / --	-- / --	--

Region	First author year	Country	Total sample size	Crude prevalence of hypertension rural/urban	Weighted prevalence of hypertension rural/urban	Hypertension prevalence weighted by	SBP/DBP rural	SBP/DBP urban	SBP/DBP prevalence weighted by
East Asia & Pacific	Cai 2012	China	5,551	34.9% / 35.6%	--	No	-- / --	-- / --	--
East Asia & Pacific	Mohamud 2012	Malaysia	4,341	30.7% / 27.2%	--	No	-- / --	-- / --	--
East Asia & Pacific	Nguyen 2012	Vietnam	4,292	--	18.7% / 30.4%	Age standardized	124.1 / 76.3	125.4 / 78.6	Age standardi
East Asia & Pacific	Attard 2015	China	5,475	25.0% / 27.1%	--	No	120.3 / 79.6	120.2 / 80	No
East Asia & Pacific	Bjertness 2016	Myanmar	7,319	27.8% / 34.0%	--	No	-- / --	-- / --	--
East Asia & Pacific	Jensen 2018	Vietnam	2,333	--	10.0% / 12.0%	Both	118 / --	119 / --	Both
East Asia & Pacific	WangJ 2014	China	50,171	--	29.0% / 32.0%	Both	-- / --	-- / --	--
East Asia & Pacific	Pengpid 2022	Mongolia	1,033	--	30.5% / 29.9%	Sampling weights	-- / --	-- / --	--
East Asia & Pacific	Bi 2015	China	96,121	--	34.5% / 35.6%	Both	-- / --	-- / --	--
East Asia & Pacific	Lao 2013	China	8,577	--	11.7% / 15.8%	Both	124 / 79	124 / 79	Both
East Asia & Pacific	WangH 2013	China	17,437	27.0% / 34.5%	--	No	-- / --	-- / --	--
East Asia & Pacific	Zheng 2012	China	1,003	44.2% / 56.1%	--	No	133.5 / 86.9	141.5 / 93.16	No
East Asia & Pacific	Bi 2014	China	15,350	--	24.6% / 20.8%	Both	122.1 / 79.3	118.6 / 77.9	Both
East Asia & Pacific	Li J 2017	China	12,338	28.3% / 29.4%	--	No	-- / --	-- / --	--
East Asia & Pacific	Naing 2016	Malaysia	18,098	37.4% / 30.9%	--	No	-- / --	-- / --	--
East Asia & Pacific	Ding 2020	China	17,302	36.1% / 40.1%	--	No	-- / --	-- / --	--
East Asia & Pacific	Fan 2014	China	18,772	24.9% / 24.9%	--	No	-- / --	-- / --	--
East Asia & Pacific	Huang 2017	China	2,676	14.8% / 13.2%	--	No	-- / --	-- / --	--
East Asia & Pacific	Liu X 2017b	China	14,420	26.0% / 21.6%	--	No	130.51 / 75.77	124.86 / 74.11	No
East Asia & Pacific	Wagner 2018	Cambodia	13,422	27.9% / 34.7%	--	No	-- / --	-- / --	--
East Asia & Pacific	WangZ 2018	China	451,755	28.8% / 26.9%	23.1% / 23.4%	Both	126.4 / 76	125.6 / 76	Both
East Asia & Pacific	Zhang 2016	China	1,196,422	22.6% / 23.1%	--	No	-- / --	-- / --	--
East Asia & Pacific	Htet 2017	Myanmar	1,486	--	34.2% / 34.5%	Age standardized	127.4 / 80	127.9 / 81.2	Age standardi
East Asia & Pacific	Hu 2016	China	8,193	22.2% / 20.7%	--	No	-- / --	-- / --	--

Region	First author year	Country	Total sample size	Crude prevalence of hypertension rural/urban	Weighted prevalence of hypertension rural/urban	Hypertension prevalence weighted by	SBP/DBP rural	SBP/DBP urban	SBP/DBP prevalence weighted by
East Asia & Pacific	Hu 2017	China	15,296	24.0% / 33.7%	--	No	-- / --	-- / --	--
East Asia & Pacific	Huang 2016	China	3,230	41.0% / 48.0%	--	No	-- / --	-- / --	--
East Asia & Pacific	Li Q 2017	China	207,323	36.1% / 32.2%	--	No	131.25 / --	128.94 / --	Age standardi
East Asia & Pacific	Li Y 2017	China	174,621	--	31.6% / 32.3%	Both	-- / --	-- / --	--
East Asia & Pacific	Wei 2015	China	3,778	36.3% / 44.5%	--	No	-- / --	-- / --	--
East Asia & Pacific	Yang 2016	China	19,254	25.2% / 24.1%	--	No	-- / --	-- / --	--
East Asia & Pacific	Isa 2021	Malaysia	3,453	28.6% / 17.2%	--	No	-- / --	-- / --	--
East Asia & Pacific	Lu 2017	China	1,738,886	46.1% / 42.5%	--	No	-- / --	-- / --	--
East Asia & Pacific	Pengpid 2022	Mongolia	1,178	--	31.3% / 25.6%	Sampling weights	-- / --	-- / --	--
East Asia & Pacific	WangJ 2018	China	14,956	--	25.9% / 22.7%	Both	130.55 / 77.32	126.18 / 75.96	Both
East Asia & Pacific	Han 2020	China	9,225	--	58.7% / 43.3%	Age standardized	-- / --	-- / --	--
East Asia & Pacific	Li 2020	China	14,596	--	25.9% / 22.7%	Both	-- / --	-- / --	--
East Asia & Pacific	Sujarwoto 2020	Indonesia	30,451	--	29.2% / 31.2%	Both	133 / 80.03	132.02 / 80.52	No
East Asia & Pacific	Wang 2019	China	47,040	--	25.8% / 28.2%	Age standardized	120.7 / 76.4	124.8 / 76.5	No
East Asia & Pacific	Hoang 2019	Vietnam	3,080	21.3% / 22.5%	19.3% / 18.4%	Sampling weights	-- / --	-- / --	--
East Asia & Pacific	Ma 2021	China	8,907	--	27.4% / 22.9%	Age standardized	124.2 / 80.4	122.3 / 78.6	Age standardi
East Asia & Pacific	Naidu 2019	Malaysia	19,936	--	33.5% / 29.3%	Sampling weights	-- / --	-- / --	--
East Asia & Pacific	Wei 2019	China	5,132	37.7% / 32.0%	26.7% / 24.1%	Age standardized	128.01 / 79.71	122.76 / 78.21	No
East Asia & Pacific	ZhangFL 2017	China	4,052	58.3% / 57.0%	56.7% / 59.2%	Both	-- / --	-- / --	--
East Asia & Pacific	Du 2019	China	7,512	36.6% / 33.4%	31.6% / 27.4%	Sampling weights	129.55 / 79.65	123.5 / 78.79	Sampling wei
East Asia & Pacific	Maharani 2019	Indonesia	17,865	51.7% / 55.4%	--	No	139.6 / 87.5	140.7 / 88.9	No
East Asia & Pacific	Ma 2020	China	12,957	--	20.9% / 19.7%	Age standardized	-- / --	-- / --	--
East Asia & Pacific	Xing 2019	China	18,796	59.2% / 50.9%	52.5% / 43.5%	Age standardized	145.1 / 86.2	137.6 / 83.4	No
East Asia & Pacific	Li 2021	China	8,211	43.5% / 36.4%	--	No	138.43 / 76.33	134.27 / 75.46	No

Region	First author year	Country	Total sample size	Crude prevalence of hypertension rural/urban	Weighted prevalence of hypertension rural/urban	Hypertension prevalence weighted by	SBP/DBP rural	SBP/DBP urban	SBP/DBP prevalence weighted by
East Asia & Pacific	Yu 2021	China	2,426	46.9% / 36.0%	--	No	131.34 / 84.45	124.24 / 82.11	No
East Asia & Pacific	Pengpid 2022	Mongolia	4,110	--	17.7% / 14.9%	Sampling weights	-- / --	-- / --	--
East Asia & Pacific	Su 2020	China	9,745,640	8.5% / 9.0%	--	No	118.95 / 72.51	120.02 / 73.58	No
Europe & Central Asia	Sonmez 1999	Turkey	1,466	35.4% / 30.4%	--	No	132.6 / 77.1	128.7 / 78.9	No
Europe & Central Asia	Altun 2005	Turkey	4,910	--	32.9% / 31.1%	Both	-- / --	-- / --	--
Europe & Central Asia	Dorobantu 2010	Romania	2,017	49.5% / 41.6%	--	No	-- / --	-- / --	--
Europe & Central Asia	Harhay 2013	Armenia	7,382	--	24.3% / 21.7%	Both	-- / --	-- / --	--
Europe & Central Asia	Harhay 2013	Azerbaijan	10,637	--	18.5% / 14.8%	Both	-- / --	-- / --	--
Europe & Central Asia	Metintas 2009	Turkey	3,000	--	57.5% / 50.5%	Age standardized	-- / --	-- / --	--
Europe & Central Asia	Basu 2013	Russia	4,355	56.7% / 50.7%	--	No	-- / --	-- / --	--
Europe & Central Asia	Harhay 2013	Ukraine	7,454	--	28.7% / 25.9%	Both	-- / --	-- / --	--
Europe & Central Asia	Harhay 2013	Albania	6,417	--	26.7% / 19.5%	Both	-- / --	-- / --	--
Europe & Central Asia	Markovic 2011 / Bergman	Croatia	2,378	74.5% / 62.7%	--	No	134.6 / 81.9	130.2 / 80	No
Europe & Central Asia	Oğuz 2018	Turkey	4,056	46.6% / 38.1%	--	No	-- / --	-- / --	--
Europe & Central Asia	Dastan 2017	Turkey	15,047	29.8% / 26.0%	28.4% / 23.9%	Age standardized	-- / --	-- / --	--

Region	First author year	Country	Total sample size	Crude prevalence of hypertension rural/urban	Weighted prevalence of hypertension rural/urban	Hypertension prevalence weighted by	SBP/DBP rural	SBP/DBP urban	SBP/DBP prevalence weighted by
Europe & Central Asia	Dorobantu 2012	Romania	1,975	41.4% / 39.8%	--	No	-- / --	-- / --	--
Europe & Central Asia	Marinkovic 2014	Serbia	1,669	57.9% / 49.9%	--	No	-- / --	-- / --	--
Europe & Central Asia	Lovic 2013	Serbia	3,878	55.1% / 38.8%	--	No	139.27 / 83.49	127.84 / 78.95	No
Europe & Central Asia	Sengul 2016	Turkey	5,437	32.5% / 29.6%	--	No	-- / --	-- / --	--
Europe & Central Asia	Supiyev 2016	Kazakhstan	953	73.6% / 70.6%	--	No	138.4 / 91.8	135 / 87.8	No
Europe & Central Asia	Artyukhov 2017	Russia	1,541	63.4% / 44.2%	--	No	-- / --	-- / --	--
Europe & Central Asia	Abba 2022	Albania	20,846	--	31.6% / 27.8%	Sampling weights	-- / --	-- / --	--
Europe & Central Asia	Balanova 2019	Russia	6,714	--	42.9% / 43.7%	Age standardized	-- / --	-- / --	--
Latin America & Caribbean	Guerrero-Romero 2000	Mexico	1,790	6.9% / 26.5%	--	No	108 / 73.7	120.4 / 77.6	No
Latin America & Caribbean	Beltran-Sanchez 2011	Mexico	14,280	--	37.7% / 37.5%	Both	-- / --	-- / --	--
Latin America & Caribbean	Prince 2012	Peru	1,933	42.6% / 52.6%	--	No	118.6 / 71.7	123 / 71.3	No
Latin America & Caribbean	Prince 2012	Mexico	2,003	56.6% / 69.2%	--	No	129.6 / 76.7	132.7 / 77.7	No
Latin America & Caribbean	Camacho 2016	Colombia	7,448	35.9% / 39.0%	--	No	-- / --	-- / --	--
Latin America & Caribbean	deSouza 2020	Brazil	1,644	35.7% / 30.7%	--	No	-- / --	-- / --	--

Region	First author year	Country	Total sample size	Crude prevalence of hypertension rural/urban	Weighted prevalence of hypertension rural/urban	Hypertension prevalence weighted by	SBP/DBP rural	SBP/DBP urban	SBP/DBP prevalence weighted by
Latin America & Caribbean	Nieto-Martínez 2018	Venezuela	751	25.4% / 31.0%	--	No	118.6 / 76.9	119.8 / 74.8	No
Latin America & Caribbean	Basu 2013	Mexico	2,733	31.3% / 31.0%	--	No	-- / --	-- / --	--
Latin America & Caribbean	Ferguson 2011	Jamaica	2,848	--	28.6% / 23.4%	Sampling weights	125.4 / 78	121.9 / 77.2	Sampling weig
Latin America & Caribbean	Laux 2012	Nicaragua	855	23.5% / 22.5%	--	No	-- / --	-- / --	--
Latin America & Caribbean	Miranda 2011	Peru	989	11.9% / 17.3%	10.7% / 18.9%	Age standardized	120.9 / 74.2	122 / 72.5	No
Latin America & Caribbean	Lamelas 2019	Argentina	11,140	49.6% / 49.5%	--	No	-- / --	-- / --	--
Latin America & Caribbean	McDonaldPosso 2014	Panama	3,406	27.6% / 31.7%	--	No	-- / --	-- / --	--
Latin America & Caribbean	Bernabe-Ortiz 2017b	Peru	2,656	26.9% / 17.9%	--	No	-- / --	-- / --	--
Latin America & Caribbean	Pérez-Galarza 2021	Ecuador	10,318	--	7.4% / 9.7%	Age standardized	-- / --	-- / --	--
Latin America & Caribbean	Almeida 2015	Brazil	1,410	48.9% / 55.5%	--	No	134.09 / 78.79	128.63 / 84.08	No
Latin America & Caribbean	dePaula 2015	Brazil	435	80.7% / 60.2%	--	No	-- / --	-- / --	--
Latin America & Caribbean	Krishnadath 2016	Suriname	5,536	--	27.6% / 25.4%	Both	-- / --	-- / --	--
Latin America & Caribbean	Malta 2018	Brazil	59,402	--	32.1% / 33.1%	Sampling weights	-- / --	-- / --	--
Latin America & Caribbean	DeGennaroJr 2018	Haiti	2,131	14.1% / 17.1%	--	No	-- / --	-- / --	--

Region	First author year	Country	Total sample size	Crude prevalence of hypertension rural/urban	Weighted prevalence of hypertension rural/urban	Hypertension prevalence weighted by	SBP/DBP rural	SBP/DBP urban	SBP/DBP prevalence weighted by
Latin America & Caribbean	deSouza 2020	Brazil	1,201	32.4% / 33.9%	--	No	-- / --	-- / --	--
Latin America & Caribbean	Orantes-Navarro 2019	El Salvador	4,817	--	32.7% / 40.1%	Sampling weights	-- / --	-- / --	--
Latin America & Caribbean	Santiago 2019	Brazil	416	28.0% / 27.0%	--	No	-- / --	-- / --	--
Latin America & Caribbean	Chambergo-Michilot 2021	Peru	33,336	--	17.3% / 20.4%	Sampling weights	-- / --	-- / --	--
Latin America & Caribbean	Felix 2020	Ecuador	2,020	24.0% / 29.0%	--	No	123.25 / 77.57	121.68 / 79.72	No
Middle East & North Africa	SarrafZadegan 1997	Iran	6,532	10.0% / 27.0%	--	No	109.1 / 67.8	134.4 / 84.9	No
Middle East & North Africa	Abdul-Rahim 2001	West Bank and Gaza	992	25.4% / 21.5%	--	No	124.8 / 76.3	122.6 / 78.4	Age standardi
Middle East & North Africa	Bouguerra 2006	Tunisia	3,857	25.7% / 29.2%	--	No	120.5 / 70.7	120.6 / 71.3	Age standardi
Middle East & North Africa	Shirani 2011	Iran	12,014	19.8% / 19.6%	--	No	-- / --	-- / --	--
Middle East & North Africa	Tazi 2003	Morocco	1,802	41.9% / 37.3%	34.3% / 32.6%	Age standardized	130 / 75.8	129.4 / 76.1	Age standardi
Middle East & North Africa	Azimi-Nezhad 2009a	Iran	4,928	26.5% / 28.8%	--	No	-- / --	-- / --	No
Middle East & North Africa	BenRomdhane 2012	Tunisia	8,007	28.3% / 32.3%	--	No	-- / --	-- / --	--
Middle East & North Africa	Janghorbani 2008	Iran	69,722	22.6% / 23.8%	--	No	-- / --	-- / --	--
Middle East & North Africa	Malekzadeh 2013	Iran	50,045	42.9% / 41.6%	--	No	-- / --	-- / --	--

Region	First author year	Country	Total sample size	Crude prevalence of hypertension rural/urban	Weighted prevalence of hypertension rural/urban	Hypertension prevalence weighted by	SBP/DBP rural	SBP/DBP urban	SBP/DBP prevalence weighted by
Middle East & North Africa	Aounallah-Skhiri 2012	Tunisia	2,870	--	6.0% / 4.1%	Sampling weights	-- / --	-- / --	--
Middle East & North Africa	Esteghamati 2016	Iran	68,850	24.7% / 26.0%	--	No	120.9 / 76.8	121.3 / 77.7	No
Middle East & North Africa	Ebrahimi 2010	Iran	29,971	--	16.3% / 17.9%	Sampling weights	-- / --	-- / --	--
Middle East & North Africa	Esteghamati 2016	Iran	4,184	20.5% / 26.5%	--	No	121.6 / 79.1	122.3 / 80.6	No
Middle East & North Africa	Hammami 2011	Tunisia	598	50.0% / 52.3%	--	No	-- / --	-- / --	--
Middle East & North Africa	Modesti 2013a	Yemen	10,242	13.2% / 12.4%	7.8% / 7.5%	Age standardized	118.8 / 74.5	119 / 74.9	Age standardi
Middle East & North Africa	Ziyyat 2014	Morocco	1,628	39.9% / 29.0%	--	No	-- / --	-- / --	--
Middle East & North Africa	Katibeh 2020	Iran	2,098	67.0% / 54.5%	--	No	-- / --	-- / --	--
Middle East & North Africa	Khorrami 2017	Iran	5,998	16.5% / 13.8%	--	No	-- / --	-- / --	--
Middle East & North Africa	Naghipour 2021	Iran	10,520	39.9% / 47.4%	--	No	-- / --	-- / --	--
Middle East & North Africa	Rajati 2019	Iran	10,040	17.0% / 14.9%	--	No	-- / --	-- / --	--
Middle East & North Africa	Rezaianzadeh 2021	Iran	10,663	27.6% / 27.8%	--	No	-- / --	-- / --	--
Middle East & North Africa	Fahs 2017	Lebanon	1,000	62.6% / 59.8%	--	No	131.87 / 81.06	130.87 / 80.44	No
Middle East & North Africa	Ahmadi 2021	Iran	10,075	13.9% / 18.5%	--	No	111.7 / 73.8	117 / 76.3	No

Region	First author year	Country	Total sample size	Crude prevalence of hypertension rural/urban	Weighted prevalence of hypertension rural/urban	Hypertension prevalence weighted by	SBP/DBP rural	SBP/DBP urban	SBP/DBP prevalence weighted by
Middle East & North Africa	Pengpid 2020	Morocco	4,555	--	27.9% / 23.4%	Both	-- / --	-- / --	--
Middle East & North Africa	Allameh 2022	Iran	4,112	16.1% / 18.7%	15.6% / 18.4%	Both	-- / --	-- / --	--
Middle East & North Africa	Jalali 2021	Iran	220,241	16.8% / 21.5%	--	No	113.8 / 70.74	115.68 / 72.46	No
South Asia	Jafar 2003	Pakistan	8,276	18.1% / 22.7%	--	No	-- / --	-- / --	--
South Asia	Oommen 2016b	India	7,342	7.6% / 18.2%	--	No	110.17 / 71.33	117.85 / 76.09	No
South Asia	Prabhakaran 2017	India	5,510	--	11.2% / 23.0%	Age standardized	114.9 / 73.1	121.2 / 74.3	Age standardi
South Asia	Gupta 2009	India	4,712	19.1% / 27.9%	--	No	124.8 / 80.3	123.8 / 80.1	No
South Asia	Kumar 2006	India	2,200	8.8% / 44.2%	--	No	-- / --	-- / --	--
South Asia	Kusuma 2004	India	1,316	19.5% / 22.7%	--	No	-- / --	-- / --	--
South Asia	Sayeed 2002	Bangladesh	1,760	17.7% / 11.3%	--	No	-- / --	-- / --	--
South Asia	Singh 1997a	India	3,575	22.8% / 24.0%	--	No	-- / --	-- / --	--
South Asia	Samuel 2012	India	2,218	1.8% / 3.3%	--	No	106.1 / 71.6	108.2 / 73.9	No
South Asia	Quasem 2001	India	723	55.0% / 70.2%	--	No	138 / 83	147 / 86	No
South Asia	Quasem 2001	Bangladesh	480	53.0% / 75.0%	--	No	-- / --	-- / --	--
South Asia	Allender 2010	India	3,205	19.1% / 26.6%	--	No	125.4 / 75.99	128.8 / 78.65	No
South Asia	Gupta 2017	India	28,747	22.8% / 39.0%	--	No	-- / --	-- / --	--
South Asia	Gupta 2017	Bangladesh	2,934	43.0% / 36.0%	--	No	-- / --	-- / --	--
South Asia	Gupta 2017	Pakistan	1,742	22.0% / 38.0%	--	No	-- / --	-- / --	--
South Asia	Midha 2009	India	800	14.5% / 32.8%	--	No	120.5 / 77.8	128.4 / 82.6	No
South Asia	Prince 2012	India	2,004	45.6% / 68.6%	--	No	124.4 / 82.2	134.3 / 89.8	No
South Asia	Hussain 2005	Bangladesh	6,312	7.9% / 1.3%	--	No	119.7 / 77.2	106.1 / 70.2	No
South Asia	Farag 2014	India	5,928	37.2% / 49.5%	--	No	-- / --	-- / --	--

Region	First author year	Country	Total sample size	Crude prevalence of hypertension rural/urban	Weighted prevalence of hypertension rural/urban	Hypertension prevalence weighted by	SBP/DBP rural	SBP/DBP urban	SBP/DBP prevalence weighted by
South Asia	Katulanda 2014	Sri Lanka	4,485	--	22.9% / 26.5%	Age standardized	127.6 / 76.6	127 / 75	No
South Asia	Millett 2013	India	3,902	13.5% / 25.6%	--	No	-- / --	-- / --	--
South Asia	Thankappan 2010	India	7,449	32.5% / 32.8%	--	No	127.6 / 78.8	127.1 / 78.7	No
South Asia	Kusuma 2008	India	803	26.6% / 37.0%	--	No	125.5 / 81.2	129.8 / 84.2	No
South Asia	Tareen 2011	Pakistan	2,495	16.0% / 35.0%	--	No	-- / --	-- / --	--
South Asia	Nanditha 2021	India	4,776	--	14.7% / 21.4%	Age standardized	-- / --	-- / --	--
South Asia	Basu 2013	India	12,198	22.1% / 27.1%	--	No	-- / --	-- / --	--
South Asia	Norboo 2015	India	2,800	33.5% / 43.4%	--	No	-- / --	-- / --	--
South Asia	Bhagyalaxmi 2013	India	3,489	15.4% / 29.1%	--	No	-- / --	-- / --	--
South Asia	Bhansali 2015	India	13,800	--	23.0% / 29.7%	Age standardized	126.5 / 77.3	129.4 / 79.8	No
South Asia	Das 2011	India	448	33.2% / 48.9%	--	No	129.75 / 80.42	136.78 / 84.8	No
South Asia	Bharati 2012	India	856	26.3% / 28.7%	--	No	-- / --	-- / --	--
South Asia	Rahman 2017	Bangladesh	9,275	17.9% / 22.2%	--	No	-- / --	-- / --	--
South Asia	Oommen 2016a	India	5,275	17.2% / 28.5%	--	No	115.82 / 76.2	119.15 / 77.91	No
South Asia	Prabhakaran 2017	India	3,940	--	28.9% / 42.2%	Age standardized	123.1 / 82.3	129.8 / 83.9	Age standardi
South Asia	Biswas 2016	Bangladesh	8,835	10.8% / 13.1%	--	No	-- / --	-- / --	--
South Asia	Krishnan 2016	India	5,153	--	24.5% / 34.4%	Age standardized	-- / --	-- / --	--
South Asia	Geldsetzer 2018	India	1,320,510	25.1% / 29.5%	--	No	-- / --	-- / --	--
South Asia	Aryal 2015	Nepal	4,143	--	24.9% / 29.1%	Sampling weights	-- / --	-- / --	--
South Asia	Kanungo 2017	India	18,028	23.9% / 29.2%	--	No	-- / --	-- / --	--
South Asia	Zaman 2015	Bangladesh	4,073	19.0% / 27.5%	--	No	-- / --	-- / --	--
South Asia	Bandela 2017	India	1,032	20.7% / 19.5%	--	No	122.97 / 82.22	122.31 / 81.58	No
South Asia	Bhadoria 2014	India	911	14.8% / 21.4%	--	No	118.04 / 72.92	121.63 / 74.43	No
South Asia	Tripathy 2017	India	5,055	40.0% / 40.4%	--	No	-- / --	-- / --	--

Region	First author year	Country	Total sample size	Crude prevalence of hypertension rural/urban	Weighted prevalence of hypertension rural/urban	Hypertension prevalence weighted by	SBP/DBP rural	SBP/DBP urban	SBP/DBP prevalence weighted by
South Asia	Islam 2018	Bangladesh	1,843	14.6% / 23.2%	--	No	115 / 74.9	117.9 / 77.9	No
South Asia	Khanam 2021	Bangladesh	12,863	27.0% / 28.6%	--	No	-- / --	-- / --	--
South Asia	Kumar 2021	India	811,808	--	11.7% / 13.1%	Sampling weights	-- / --	-- / --	--
South Asia	Mehata 2018	Nepal	13,598	16.3% / 18.9%	16.3% / 18.9%	Sampling weights	-- / --	-- / --	--
South Asia	Basit 2020	Pakistan	9,594	52.4% / 52.9%	46.8% / 44.3%	Age standardized	-- / --	-- / --	--
South Asia	Nanditha 2021	India	6,318	--	14.0% / 26.3%	Age standardized	-- / --	-- / --	--
South Asia	Sarma 2019	India	12,012	--	29.8% / 33.1%	Sampling weights	126.6 / 80.7	126.6 / 80.9	Sampling weig
South Asia	Mohanty 2020	India	70,031	--	14.8% / 14.8%	Sampling weights	128.4 / 83.6	129.3 / 84.7	No
South Asia	Mohanty 2021	India	64,427	--	37.8% / 51.8%	Sampling weights	-- / --	-- / --	--
South Asia	Riaz 2020	Bangladesh	8,185	--	19.8% / 25.2%	Sampling weights	119.7 / 78.4	122.6 / 80.6	Sampling weig
South Asia	Hanif 2021	Bangladesh	4,813	49.3% / 56.4%	49.0% / 47.7%	Sampling weights	-- / --	-- / --	--
South Asia	Hasan 2021	Bangladesh	4,856	30.6% / 36.9%	--	No	-- / --	-- / --	--
South Asia	Kokane 2020	India	4,985	--	10.7% / 25.6%	Sampling weights	-- / --	-- / --	--
South Asia	Paul 2021	Bangladesh	529	43.5% / 62.0%	--	No	-- / --	-- / --	--
South Asia	Patel 2021	India	38,694	--	20.1% / 21.0%	Sampling weights	-- / --	-- / --	--
South Asia	Sivanantham 2021	India	2,415	--	28.2% / 36.0%	Sampling weights	122 / 80	125 / 82	No
Sub-Saharan Africa	Cooper 1997	Cameroon	2,828	--	15.4% / 19.1%	Age standardized	119.65 / 73.46	120.78 / 75.47	No
Sub-Saharan Africa	Okosun	Nigeria	1,934	12.6% / 16.1%	--	No	122 / 69.4	113.9 / 71.6	No
Sub-Saharan Africa	Fezeu 2010	Cameroon	1,762	--	14.2% / 22.0%	Age standardized	117.48 / 72.07	117.22 / 77.37	No
Sub-Saharan Africa	Ezenwaka 1997	Nigeria	500	29.5% / 71.2%	--	No	127.2 / 76.8	148.6 / 88.1	No
Sub-Saharan Africa	Edwards 2000	Tanzania	1,689	32.0% / 30.1%	--	No	128 / 81.6	127.1 / 81.4	No
Sub-Saharan Africa	VanDerSande 2000	Gambia	5,389	17.8% / 20.3%	--	No	119.9 / 69.7	123.4 / 73.3	No
Sub-Saharan Africa	VanRooyen 2000	South Africa	1,783	21.2% / 26.7%	--	No	-- / --	-- / --	--
Sub-Saharan Africa	Kandala 2013	South Africa	13,596	28.7% / 31.8%	--	No	-- / --	-- / --	--

Region	First author year	Country	Total sample size	Crude prevalence of hypertension rural/urban	Weighted prevalence of hypertension rural/urban	Hypertension prevalence weighted by	SBP/DBP rural	SBP/DBP urban	SBP/DBP prevalence weighted by
Sub-Saharan Africa	Sobngwi 2002	Cameroon	2,465	--	13.7% / 25.2%	Age standardized	118.04 / 76.63	121.66 / 78.22	No
Sub-Saharan Africa	Longo-Mbenza 2008	Democratic Republic of the Congo	1,952	--	17.0% / 12.8%	Sampling weights	-- / --	-- / --	--
Sub-Saharan Africa	Kodaman 2016	Ghana	3,317	--	20.6% / 32.9%	Age standardized	125.4 / 73.6	127.3 / 77.8	No
Sub-Saharan Africa	Balde 2007	Guinea	1,537	14.9% / 43.6%	--	No	118.9 / 74.1	132.8 / 82.4	No
Sub-Saharan Africa	Fezeu 2010	Cameroon	1,398	--	38.6% / 38.6%	Age standardized	136.27 / 83.97	124.49 / 78.86	No
Sub-Saharan Africa	Agyemang 2006	Ghana	1,431	27.0% / 31.0%	--	No	127.49 / 75.38	131.8 / 79.34	No
Sub-Saharan Africa	Mufunda 2006	Eritrea	2,352	14.5% / 16.5%	--	No	-- / --	-- / --	--
Sub-Saharan Africa	Damasceno 2009	Mozambique	3,081	--	30.0% / 41.0%	Sampling weights	-- / --	-- / --	--
Sub-Saharan Africa	Dolman 2014	South Africa	1,710	34.8% / 52.2%	--	No	128.7 / 86	137.4 / 88.9	No
Sub-Saharan Africa	Murthy 2013	Nigeria	13,504	--	43.0% / 51.6%	Sampling weights	137.2 / 83.1	141.6 / 86.6	Sampling wei
Sub-Saharan Africa	Delisle 2012	Benin	541	20.5% / 19.7%	--	No	123.9 / 78.8	124.4 / 76.2	No
Sub-Saharan Africa	Basu 2013	Ghana	5,563	37.3% / 45.0%	--	No	-- / --	-- / --	--
Sub-Saharan Africa	Basu 2013	South Africa	4,223	54.7% / 47.7%	--	No	-- / --	-- / --	--
Sub-Saharan Africa	Ejim 2013	Nigeria	543	45.1% / 51.1%	--	No	135.8 / 80.4	131.7 / 85	No
Sub-Saharan Africa	Mathenge 2010	Kenya	4,376	47.0% / 57.0%	--	No	140 / 82.1	143 / 82	No
Sub-Saharan Africa	vanZyl 2012	South Africa	976	67.9% / 56.9%	--	No	-- / --	-- / --	--
Sub-Saharan Africa	Giday 2011	Ethiopia	979	9.7% / 10.1%	--	No	-- / --	-- / --	--
Sub-Saharan Africa	Houehanou 2015	Benin	6,762	--	27.5% / 29.9%	Sampling weights	128.1 / 78.3	127.6 / 79.4	No
Sub-Saharan Africa	Muluneh AT 2012	Ethiopia	3,223	7.7% / 17.4%	--	No	-- / --	-- / --	--
Sub-Saharan Africa	Camara 2016	Guinea	2,491	--	36.3% / 26.0%	Both	-- / --	-- / --	--
Sub-Saharan Africa	Egbujie 2016	South Africa	1,311	71.8% / 74.0%	--	No	-- / --	-- / --	--
Sub-Saharan Africa	Isezuo SA 2011	Nigeria	782	24.7% / 24.9%	--	No	-- / --	-- / --	--

Region	First author year	Country	Total sample size	Crude prevalence of hypertension rural/urban	Weighted prevalence of hypertension rural/urban	Hypertension prevalence weighted by	SBP/DBP rural	SBP/DBP urban	SBP/DBP prevalence weighted by
Sub-Saharan Africa	Lissock 2011	Cameroon	452	12.0% / 33.7%	--	No	135.4 / 84.4	135.3 / 84.3	No
Sub-Saharan Africa	Msyamboza 2011	Malawi	3,910	32.0% / 27.9%	--	No	-- / --	-- / --	--
Sub-Saharan Africa	Abegunde 2013	Nigeria	600	34.7% / 38.3%	--	No	-- / --	-- / --	--
Sub-Saharan Africa	Adediran 2013	Nigeria	667	4.8% / 17.3%	--	No	113.33 / 73.22	122.46 / 76.58	No
Sub-Saharan Africa	Cham 2018	Gambia	3,219	--	34.8% / 25.0%	Sampling weights	-- / --	-- / --	--
Sub-Saharan Africa	Mengistu 2014	Ethiopia	1,183	15.2% / 20.1%	--	No	113.9 / 76.8	119.4 / 76.7	No
Sub-Saharan Africa	Okpechi 2013	Nigeria	2,983	32.0% / 30.7%	--	No	134.79 / 77.95	133.7 / 77.45	No
Sub-Saharan Africa	Abebe 2015	Ethiopia	2,141	25.3% / 30.7%	--	No	129.5 / --	129.4 / --	No
Sub-Saharan Africa	Agyemang 2018	Ghana	2,492	--	25.9% / 32.0%	Age standardized	123.8 / 77.1	126.5 / 79.4	No
Sub-Saharan Africa	Kavishe 2015	Tanzania	1,095	18.6% / 16.4%	17.6% / 16.7%	Sampling weights	-- / --	-- / --	--
Sub-Saharan Africa	Kavishe 2015	Uganda	916	25.7% / 19.6%	26.3% / 20.5%	Sampling weights	-- / --	-- / --	--
Sub-Saharan Africa	Mosha 2017	Tanzania	9,678	6.8% / 10.0%	--	No	-- / --	-- / --	--
Sub-Saharan Africa	Musinguzi 2013	Uganda	4,563	21.0% / 23.6%	--	No	-- / --	-- / --	--
Sub-Saharan Africa	Nahimana 2017	Rwanda	6,524	--	15.0% / 15.7%	Sampling weights	-- / --	-- / --	--
Sub-Saharan Africa	Nakibuuka 2015	Uganda	5,193	27.1% / 22.4%	--	No	-- / --	-- / --	--
Sub-Saharan Africa	Seck 2014b	Senegal	1,036	33.8% / 43.3%	--	No	130.5 / 84.3	131.33 / 87.9	No
Sub-Saharan Africa	Kandala 2021	South Africa	6,867	43.0% / 45.1%	--	No	-- / --	-- / --	--
Sub-Saharan Africa	Katchunga 2019	Democratic Republic of the Congo	5,580	--	16.0% / 23.5%	Age standardized	115.7 / 73.9	119.7 / 77.4	No
Sub-Saharan Africa	BA 2018	Mali	2,102	21.1% / 24.7%	--	No	150.94 / 94.1	151.23 / 94.62	No
Sub-Saharan Africa	Obirikorang 2015	Ghana	672	36.7% / 32.7%	--	No	133.5 / 79.5	133 / 80	No
Sub-Saharan Africa	Price 2018	Malawi	28,891	13.6% / 14.7%	--	No	121.1 / 72.8	123.6 / 73.9	No
Sub-Saharan Africa	Ratovoson 2015	Madagascar	7,631	27.0% / 29.7%	--	No	-- / --	-- / --	--

Region	First author year	Country	Total sample size	Crude prevalence of hypertension rural/urban	Weighted prevalence of hypertension rural/urban	Hypertension prevalence weighted by	SBP/DBP rural	SBP/DBP urban	SBP/DBP prevalence weighted by
Sub-Saharan Africa	Soubeiga 2017	Burkina Faso	4,629	15.4% / 23.8%	17.0% / 27.4%	Both	-- / --	-- / --	--
Sub-Saharan Africa	Craig 2018	Namibia	3,068	39.9% / 50.0%	--	No	-- / --	-- / --	--
Sub-Saharan Africa	Guwatudde 2015	Uganda	3,906	25.8% / 28.2%	--	No	-- / --	-- / --	--
Sub-Saharan Africa	Jessen 2018	Mozambique	2,965	--	31.1% / 31.5%	Sampling weights	-- / --	-- / --	--
Sub-Saharan Africa	Kaze 2015	Cameroon	439	28.8% / 16.8%	28.8% / 16.8%	Sampling weights	121.2 / 78.1	115 / 77.4	No
Sub-Saharan Africa	Lemogoum 2018	Cameroon	889	33.9% / 42.7%	34.0% / 41.2%	Age standardized	132 / 78	136 / 81	No
Sub-Saharan Africa	Oguoma 2015	Nigeria	422	14.8% / 32.3%	--	No	120 / 72.1	133 / 80	No
Sub-Saharan Africa	Stanifer 2016	Tanzania	481	33.3% / 30.3%	33.2% / 19.4%	Age standardized	132.8 / 78.5	124 / 76.1	Age standardi
Sub-Saharan Africa	Sanuade 2018	Ghana	13,247	--	9.6% / 16.6%	Sampling weights	-- / --	-- / --	--
Sub-Saharan Africa	Gebreyes 2018	Ethiopia	9,788	--	14.9% / 19.7%	Sampling weights	-- / --	-- / --	--
Sub-Saharan Africa	Tesfaye 2019	Ethiopia	1,405	13.0% / 10.0%	--	No	-- / --	-- / --	--
Sub-Saharan Africa	Umuerri 2020	Nigeria	852	21.8% / 35.4%	--	No	123.53 / 72.25	126.19 / 78.37	No
Sub-Saharan Africa	Walekhwa 2021	Kenya	4,352	20.4% / 23.2%	--	No	-- / --	-- / --	--
Sub-Saharan Africa	Colette 2020	Benin	540	27.6% / 37.0%	--	No	-- / --	-- / --	--
Sub-Saharan Africa	Kandala 2021	South Africa	8,230	45.4% / 51.6%	--	No	-- / --	-- / --	--
Sub-Saharan Africa	Katchunga 2019	Democratic Republic of the Congo	5,286	--	15.1% / 25.4%	Age standardized	117.2 / 78.1	122 / 75	No
Sub-Saharan Africa	Masimango 2020	Democratic Republic of the Congo	1,317	18.4% / 22.5%	--	No	-- / --	-- / --	--
Sub-Saharan Africa	Mika 2020	Mozambique	4,101	10.6% / 17.5%	--	No	-- / --	-- / --	--
Sub-Saharan Africa	Odili 2020	Nigeria	4,192	32.4% / 33.7%	37.5% / 39.2%	Age standardized	-- / --	-- / --	--
Sub-Saharan Africa	Odland 2020	Sierra Leone	2,071	--	46.0% / 55.8%	Age standardized	-- / --	-- / --	--
Sub-Saharan Africa	Geraedts 2021	Sierra Leone	1,956	--	22.3% / 20.2%	Sampling weights	-- / --	-- / --	--

Region	First author year	Country	Total sample size	Crude prevalence of hypertension rural/urban	Weighted prevalence of hypertension rural/urban	Hypertension prevalence weighted by	SBP/DBP rural	SBP/DBP urban	SBP/DBP prevalence weighted by
LIC	Chow 2013	LIC	31,685	26.3% / 38.6%	31.5% / 44.4%	Age standardized	-- / --	-- / --	--
LMIC	Chow 2013	LMIC	58,476	39.7% / 39.9%	38.7% / 34.9%	Age standardized	-- / --	-- / --	--
UMIC	Chow 2013	UMIC	36,463	50.9% / 48.6%	46.9% / 45.2%	Age standardized	-- / --	-- / --	--
All LMIC	Chow 2013	All LMIC	126,624	39.2% / 42.3%	--	No	-- / --	-- / --	--

LIC: low-income country, LMIC: lower-middle-income country, UMIC: upper-middle-income country. Data not available represented as --.

REFERENCES

1 Abba MS, Nduka CU, Anjorin S, Uthman OA. Household Air Pollution and High Blood Pressure: A Secondary Analysis of the 2016 Albania Demographic Health and Survey Dataset. *Int J Environ Res Public Health* 2022; **19**. DOI:10.3390/ijerph19052611.

2 Lamelas P, Diaz R, Orlandini A, *et al.* Prevalence, awareness, treatment and control of hypertension in rural and urban communities in Latin American countries. *J Hypertens* 2019; **37**: 1813–21.

3 Sayeed M.A., Banu A., Haq J.A., Khanam P.A., Mahtab H., Azad Khan A.K. Prevalence of hypertension in Bangladesh: Effect of socioeconomic risk factor on difference between rural and urban community. *Bangladesh Medical Research Council Bulletin* 2002; **28**: 7–18.

4 Hussain A., Rahim M.A., Khant A.K.A., Ali S.M.K., Vaaler S. Type 2 diabetes in rural and urban population: Diverse prevalence and associated risk factors in Bangladesh. *Diabetic Medicine* 2005; **22**: 931–6.

5 Zaman M.M., Bhuiyan M.R., Karim M.N., *et al.* Clustering of non-communicable diseases risk factors in Bangladeshi adults: An analysis of STEPS survey 2013. *BMC public health* 2015; **15**: 659.

6 Biswas T., Islam Md.S., Linton N., Rawal L.B. Socio-Economic Inequality of Chronic Non-Communicable Diseases in Bangladesh. *PLoS ONE* 2016; **11**. DOI:10.1371/journal.pone.0167140.

7 Rahman M., Zaman M.M., Islam J.Y., *et al.* Prevalence, treatment patterns, and risk factors of hypertension and pre-hypertension among Bangladeshi adults. *Journal of Human Hypertension* 2017; : 1–15.

8 Islam JY, Zaman MM, Haq SA, Ahmed S, Al- Quadir Z. Epidemiology of hypertension among Bangladeshi adults using the 2017 ACC/AHA Hypertension Clinical Practice Guidelines and Joint National Committee 7 Guidelines. *J Hum Hypertens* 2018; **32**: 668–80.

9 Riaz BK, Islam MZ, Islam ANMS, *et al.* Risk factors for non-communicable diseases in Bangladesh: Findings of the population-based cross-sectional national survey 2018. *BMJ Open* 2020; **10**. DOI:10.1136/bmjopen-2020-041334.

10 Hanif AAM, Shamim AA, Hossain MM, *et al.* Gender-specific prevalence and associated factors of hypertension among elderly Bangladeshi people: Findings from a nationally representative cross-sectional survey. *BMJ Open* 2021; **11**. DOI:10.1136/bmjopen-2020-038326.

11 Hasan M, Khan MSA, Sutradhar I, *et al.* Prevalence and associated factors of hypertension in selected urban and rural areas of Dhaka, Bangladesh: Findings from SHASTO baseline survey. *BMJ Open* 2021; **11**. DOI:10.1136/bmjopen-2020-038975.

12 Khanam M, Hasan E, Sarker AR. Prevalence and Factors of Hypertension Among Bangladeshi Adults. *High Blood Press Cardiovasc Prev* 2021; **28**: 393–403.

13 Paul GK, Rahman MM, Hamiduzzaman M, *et al.* Hypertension and its physio-psychosocial risks factors in elderly people: A cross-sectional study in north-eastern region of Bangladesh. *J Geriatr Cardiol* 2021; **18**: 75–82.

14 Delisle H, Ntandou-Bouzitou G, Agueh V, Sodjinou R, Fayomi B. Urbanisation, nutrition transition and cardiometabolic risk: the Benin study. *The British journal of nutrition* 2012; **107**: 1534–44.

15 Houehanou Y.C.N., Lacroix P., Mizehoun G.C., Preux P.-M., Marin B., Houinato D.S. Magnitude of cardiovascular risk factors in rural and urban areas in Benin: Findings from a nationwide steps survey. *PLoS ONE* 2015; **10**. DOI:10.1371/journal.pone.0126441.

16 Colette A, Charles SJ, Rosemonde K, *et al.* The prevalence of high blood pressure and its relationship with sociodemographic, anthropometric and lifestyles indicators: A population-based study in aplahoue health District, Benin. *Univers J Public Health* 2020; **8**: 65–72.

17 Almeida RC, Dias DJL, Deguchi KTP, Spesia CH, Coelho OR. Prevalence and treatment of hypertension in urban and riverside areas in Porto Velho, the Brazilian Amazon. *Postgraduate Medicine* 2015; **127**: 66–72.

18 de Paula J.A., Moreira O.C., da Silva C.D., Silva D.S., dos Santos Amorim P.R. Metabolic syndrome prevalence in elderly of urban and rural communities participants in the HIPERDIA in the city of Coimbra/MG, Brazil. *Investigacion y educacion en enfermeria* 2015; **33**: 325–33.

19 Malta DC, Gonçalves RPF, Machado ÍE, Freitas MIF, Azeredo C, Szwarcwald CL. Prevalence of arterial hypertension according to different diagnostic criteria, National Health Survey. *Rev Bras Epidemiol* 2018; **21**. DOI:10.1590/1980-549720180021.supl.1.

20 Santiago ERC, Diniz ADS, Oliveira JS, Leal VS, de Andrade MIS, de Lira PIC. Prevalence of systemic arterial hypertension and associated factors among adults from the semi-arid region of Pernambuco, Brazil. *Arq Bras Cardiol* 2019; **113**: 687–95.

21 de Souza NP, Pessoa Cesse EÂ, de Souza WV, *et al.* Temporal variation in prevalence, awareness and control of hypertension in urban and rural areas in Northeast Brazil between 2006 and 2016. *Cad Saude Publica* 2020; **36**. DOI:10.1590/0102-311X00027819.

22 Soubeiga J.K., Millogo T., Bicaba B.W., Doulougou B., Kouanda S. Prevalence and factors associated with hypertension in Burkina Faso: a countrywide cross-sectional study. *BMC public health* 2017; **17**: 64.

23 Wagner J., Naranjo D., Khun T., *et al.* Diabetes and cardiometabolic risk factors in Cambodia: Results from two screening studies. *Journal of Diabetes* 2018; **10**: 148–57.

24 Cooper R, Rotimi C, Ataman S, *et al.* The prevalence of hypertension in seven populations of West African origin. *American Journal of Public Health* 1997; **87**: 160–8.

25 Sobngwi E., Mbanya J.-C.N., Unwin N.C., *et al.* Physical activity and its relationship with obesity, hypertension and diabetes in urban and rural Cameroon. *International Journal of Obesity* 2002; **26**: 1009–16.

26 Fezeu L., Kengne A.P., Balkau B., Awah P.K., Mbanya J.C. Ten-year change in blood pressure levels and prevalence of hypertension in urban and rural Cameroon. *Journal of Epidemiology and Community Health* 2010; **64**: 360–5.

27 Lissock C.N.A.A., Sobngwi E., Ngassam E., Ngoa L.S.E. Rural and urban differences in metabolic profiles in a Cameroonian population. *Pan African Medical Journal* 2011; **10**. http://www.embase.com/search/results?subaction=viewrecord&from=export&id=L365002892.

28 Kaze F.F., Meto D.T., Halle M.-P., Ngogang J., Kengne A.-P. Prevalence and determinants of chronic kidney disease in rural and urban Cameroonians: A cross-sectional study. *BMC Nephrology* 2015; **16**. DOI:10.1186/s12882-015-0111-8.

29 Lemogoum D., Van De Borne P., Lele C.E.B., *et al.* Prevalence, awareness, treatment, and control of hypertension among rural and urban dwellers of the Far North Region of Cameroon. *Journal of Hypertension* 2018; **36**: 159–68.

30 Tao S., Wu X., Duan X., *et al.* Hypertension prevalence and status of awareness, treatment and control in China. *Chinese Medical Journal* 1995; **108**: 483–9.

31 Reynolds K., Gu D., Muntner P., *et al.* Geographic variations in the prevalence, awareness, treatment and control of hypertension in China. *Journal of Hypertension* 2003; **21**: 1273–81.

32 Wang ZW, Wu YF, Zhao LC, Li Y, Yang J, Zhou BF. Trends in prevalence, awareness, treatment and control of hypertension in the middle-aged population of China, 1992-1998. *Hypertension Research* 2004; **27**: 703–9.

33 Wu Y., Huxley R., Li L., *et al.* Prevalence, awareness, treatment, and control of hypertension in China data from the China National Nutrition and Health Survey 2002. *Circulation* 2008; **118**: 2679–86.

34 Xu L., Wang S., Wang Y.X., Wang Y.S., Jonas J.B. Prevalence of arterial hypertension in the adult population in rural and urban China: The Beijing eye study. *American Journal of Hypertension* 2008; **21**: 1117–23.

35 Zhang L., Zhang P., Wang F., *et al.* Prevalence and Factors Associated With CKD: A Population Study From Beijing. *American Journal of Kidney Diseases* 2008; **51**: 373–84.

36 Zuo H., Shi Z., Hu X., Wu M., Guo Z., Hussain A. Prevalence of metabolic syndrome and factors associated with its components in Chinese adults. *Metabolism: Clinical and Experimental* 2009; **58**: 1102–8.

37 Fu S, Li W, Zhao Y, *et al.* Prevalence of hypertension and risk factors in Heilongjiang province in 2007. *Chinese Medical Journal* 2010; **123**: 752–5.

38 Zhao J., Pang Z.C., Zhang L., *et al.* Prevalence of metabolic syndrome in rural and urban Chinese population in Qingdao. *Journal of Endocrinological Investigation* 2011; **34**: 444–8.

39 Cai L., Liu A., Zhang L., Li S., Wang P. Prevalence, awareness, treatment, and control of hypertension among adults in Beijing, China. *Clinical and Experimental Hypertension* 2012; **34**: 45–52.

40 Zheng X, Yao D-K, Zhuo-Ma C-R, *et al.* Prevalence, self-awareness, treatment, and control of hypertension in lhasa, tibet. *Clinical and Experimental Hypertension* 2012; **34**: 328–33.

41 Gao Y., Chen G., Tian H., *et al.* Prevalence of Hypertension in China: A Cross-Sectional Study. *PLoS ONE* 2013; **8**. DOI:10.1371/journal.pone.0065938.

42 Lao XQ, Xu YJ, Wong MC, *et al.* Hypertension prevalence, awareness, treatment, control and associated factors in a developing southern Chinese population: analysis of serial cross-sectional health survey data 2002-2010. *American journal of hypertension* 2013; **26**: 1335–45.

43 Wang H., Zhang X., Zhang J., *et al.* Factors Associated with Prevalence, Awareness, Treatment and Control of Hypertension among Adults in Southern China: A Community-Based, Cross-Sectional Survey. *PLoS ONE* 2013; **8**. DOI:10.1371/journal.pone.0062469.

44 Xu B., Xu Z., Xu X., Cai Q., Xu Y. Prevalence, awareness, treatment, and control of hypertension among residents in Guangdong Province, China, 2004 to 2007. *Circulation: Cardiovascular Quality and Outcomes* 2013; **6**: 217–22.

45 Bi Z, Liang X, Xu A, *et al.* Hypertension prevalence, awareness, treatment, and control and sodium intake in shandong province, China: Baseline results from shandong-ministry of health action on salt reduction and hypertension (SMASH), 2011. *Preventing Chronic Disease* 2014; **11**. DOI:10.5888/pcd11.130423.

46 Fan L, Feng S-X, Han B, *et al.* Prevalence, awareness, treatment and control of hypertension in henan province, China. *Australian Journal of Rural Health* 2014; **22**: 264–9.

47 Wang J, Zhang L, Wang F, Liu L, Wang H. Prevalence, awareness, treatment, and control of hypertension in China: results from a national survey. *Am J Hypertens* 2014; **27**: 1355–61.

48 Attard S.M., Herring A.H., Zhang B., Du S., Popkin B.M., Gordon-Larsen P. Associations between age, cohort, and urbanization with SBP and DBP in China: A population-based study across 18 years. *Journal of Hypertension* 2015; **33**: 948–56.

49 Bi Y, Jiang Y, He J, *et al.* Status of cardiovascular health in Chinese adults. *Journal of the American College of Cardiology* 2015; **65**: 1013–25.

50 Ma L., Zhao X., Tang Z., *et al.* Epidemiological characteristics of hypertension in the elderly in Beijing, China. *PLoS ONE* 2015; **10**. DOI:10.1371/journal.pone.0135480.

51 Wei Q., Sun J., Huang J., *et al.* Prevalence of hypertension and associated risk factors in Dehui City of Jilin Province in China. *Journal of Human Hypertension* 2015; **29**: 64–8.

52 Hu M., Wan Y., Yu L., *et al.* Prevalence, awareness, treatment, and control of hypertension and associated risk factors among adults in xi'an, China a cross-sectional study. *Medicine (United States)* 2016; **95**. DOI:10.1097/MD.0000000000004709.

53 Huang X., Zhou Z., Liu J., *et al.* Prevalence, awareness, treatment, and control of hypertension among China's Sichuan Tibetan population: A cross-sectional study. *Clinical and Experimental Hypertension* 2016; **38**: 457–63.

54 Lewington S, Lacey B, Clarke R, *et al.* The burden of hypertension and associated risk for cardiovascular mortality in China. *JAMA Internal Medicine* 2016; **176**: 524–32.

55 Li W., Gu H., Teo K.K., *et al.* Hypertension prevalence, awareness, treatment, and control in 115 rural and urban communities involving 47 000 people from China. *Journal of Hypertension* 2016; **34**: 39–46.

56 Wu J., Cheng X., Qiu L., *et al.* Prevalence and clustering of major cardiovascular risk factors in China: A recent cross-sectional survey. *Medicine (United States)* 2016; **95**. DOI:10.1097/MD.0000000000002712.

57 Yang L., Yan J., Tang X., Xu X., Yu W., Wu H. Prevalence, Awareness, Treatment, Control and Risk Factors Associated with Hypertension among Adults in Southern China, 2013. *PLoS ONE* 2016; **11**. DOI:10.1371/journal.pone.0146181.

58 Zhang Y., Shi Z., Liang H., *et al.* Prevalence of hypertension in Chinese population aged over 40 and subgroup of survival stroke patients. *Biomedical Research (India)* 2016; **27**: 917–22.

59 Hu L., Huang X., You C., *et al.* Prevalence and risk factors of prehypertension and hypertension in Southern China. *PLoS ONE* 2017; **12**. DOI:10.1371/journal.pone.0170238.

60 Huang XB, Chen F, Dai W, *et al.* Prevalence and risk factors associated with hypertension in the Chinese Qiang population. *Clinical and experimental hypertension (New York, NY : 1993)* 2017; : 1–7.

61 Li J, Shi L, Li S, Xu L, Qin W, Wang H. Urban-rural disparities in hypertension prevalence, detection, and medication use among Chinese Adults from 1993 to 2011. *Int J Equity Health* 2017; **16**: 50.

62 Li Q, Wu H, Yue W, *et al.* Prevalence of Stroke and Vascular Risk Factors in China: A Nationwide Community-based Study. *Scientific Reports* 2017; **7**. DOI:10.1038/s41598-017-06691-1.

63 Li Y., Yang L., Wang L., *et al.* Burden of hypertension in China: A nationally representative survey of 174,621 adults. *International Journal of Cardiology* 2017; **227**: 516–23.

64 Liu X., Liu C., Schenck H., Yi X., Wang H., Shi X. The risk factors of 9-year follow-up on hypertension in middle-aged people in Tujia-Nationality settlement of China. *Journal of Human Hypertension* 2017; **31**: 838–42.

65 Liu X., Gu W., Li Z., Lei H., Li G., Huang W. Hypertension prevalence, awareness, treatment, control, and associated factors in Southwest China: An update. *Journal of Hypertension* 2017; **35**: 637–44.

66 Lu J., Lu Y., Wang X., *et al.* Prevalence, awareness, treatment, and control of hypertension in China: data from 1·7 million adults in a population-based screening study (China PEACE Million Persons Project). *The Lancet* 2017; **390**: 2549–58.

67 Zhang F-L, Guo Z-N, Xing Y-Q, Wu Y-H, Liu H-Y, Yang Y. Hypertension prevalence, awareness, treatment, and control in northeast China: a population-based cross-sectional survey. *Journal of Human Hypertension* 2017; **32**: 54–65.

68 Wang J., Sun W., Wells G.A., *et al.* Differences in prevalence of hypertension and associated risk factors in urban and rural residents of the Northeastern region of the people's republic of China: A cross-sectional study. *PLoS ONE* 2018; **13**. DOI:10.1371/journal.pone.0195340.

69 Wang Z, Chen Z, Zhang L, *et al.* Status of Hypertension in China: Results From the China Hypertension Survey, 2012–2015. *Circulation* 2018; **137**: 2344–56.

70 Du X, Fang L, Xu J, *et al.* Prevalence, awareness, treatment and control of hypertension and sodium intake in Zhejiang Province, China: A cross-sectional survey in 2017. *PLoS ONE* 2019; **14**. DOI:10.1371/journal.pone.0226756.

71 Wang L, Li N, Heizhati M, *et al.* Prevalence, Awareness, Treatment, and Control and Related Factors of Hypertension in Multiethnic Agriculture, Stock-Raising, and Urban Xinjiang, Northwest China: A Cross-Sectional Screening for 47000 Adults. *Int J Hypertens* 2019; **2019**. DOI:10.1155/2019/3576853.

72 Wei C, Ye S, Ru Y, *et al.* Cohort profile: The Lanxi Cohort study on obesity and obesity-related non-communicable diseases in China. *BMJ Open* 2019; **9**. DOI:10.1136/bmjopen-2018-025257.

73 Xing L, Jing L, Tian Y, *et al.* Urban-rural disparities in status of hypertension in Northeast China: A population-based study, 2017-2019. *Clin Epidemiol* 2019; **11**: 801–20.

74 Ding L, Liang Y, Tan ECK, *et al.* Smoking, heavy drinking, physical inactivity, and obesity among middle-aged and older adults in China: Cross-sectional findings from the baseline survey of CHARLS 2011-2012. *BMC Public Health* 2020; **20**. DOI:10.1186/s12889-020-08625-5.

75 Han B, Wang N, Chen Y, Li Q, Zhu C, Lu Y. Prevalence of hyperuricaemia in an Eastern Chinese population: A cross-sectional study. *BMJ Open* 2020; **10**. DOI:10.1136/bmjopen-2019-035614.

76 Li T, Song X, Wu J, *et al.* Awareness of hypertension and related factors in northeastern China: a cross-sectional study. *J Hum Hypertens* 2020; **34**: 43–50.

77 Ma A, Fang K, Dong J, Dong Z. Prevalence and Related Factors of Metabolic Syndrome in Beijing, China (Year 2017). *Obes Facts* 2020; **13**: 538–47.

78 Su Y, Lu Y, Li W, *et al.* Prevalence and correlation of metabolic syndrome: A cross-sectional study of nearly 10 million multi-ethnic Chinese adults. *Diabetes Metab Syndr Obes Targets Ther* 2020; **13**: 4869–83.

79 Li Y, Hu Q, Li X, *et al.* Intraocular pressure of adults in a coastal province in southern China: the Fujian cross-sectional eye study. *Ann Palliat Med* 2021; **10**: 12390–402.

80 Ma S, Yang L, Zhao M, Magnussen CG, Xi B. Trends in hypertension prevalence, awareness, treatment and control rates among Chinese adults, 1991-2015. *J Hypertens* 2021; **39**: 740–8.

81 Yu P, Ning Y, Gao Y, *et al.* Hypertension among Mongolian adults in China: A cross-sectional study of prevalence, awareness, treatment, control, and related factors: Hypertension among Mongolian adults in China. *J Clin Hypertens* 2021; **23**: 1786–801.

82 Camacho P.A., Gomez-Arbelaez D., Molina D.I., *et al.* Social disparities explain differences in hypertension prevalence, detection and control in Colombia. *Journal of Hypertension* 2016; **34**: 2344–52.

83 Longo-Mbenza B., Ngoma D.V., Nahimana D., *et al.* Screen detection and the WHO STEPwise approach to the prevalence and risk factors of arterial hypertension in Kinshasa. *European Journal of Cardiovascular Prevention and Rehabilitation* 2008; **15**: 503–8.

84 Katchunga PB, Mirindi P, Baleke A, Ntaburhe T, Twagirumukiza M, M'buyamba-Kabangu J-R. The trend in blood pressure and hypertension prevalence in the general population of South Kivu between 2012 and 2016: Results from two representative cross-sectional surveys—The Bukavu observational study. *PLoS ONE* 2019; **14**. DOI:10.1371/journal.pone.0219377.

85 Masimango MI, Sumaili EK, Wallemacq P, *et al.* Prevalence and Risk Factors of CKD in South Kivu, Democratic Republic of Congo: A Large-Scale Population Study. *Kidney Intl Rep* 2020; **5**: 1251–60.

86 Marković B.B., Vrdoljak D., Kranjčević K., *et al.* Continental-Mediterranean and rural-urban differences in cardiovascular risk factors in Croatian population. *Croatian Medical Journal* 2011; **52**: 566–75.

87 Felix C, Baldeon ME, Zertuche F, *et al.* Low levels of awareness, treatment, and control of hypertension in Andean communities of Ecuador. *J Clin Hypertens* 2020; **22**: 1530–7.

88 Pérez-Galarza J, Baldeón L, Franco OH, *et al.* Prevalence of overweight and metabolic syndrome, and associated sociodemographic factors among adult Ecuadorian populations: the ENSANUT-ECU study. *J Endocrinol Invest* 2021; **44**: 63–74.

89 Orantes-Navarro CM, Almaguer-López MM, Alonso-Galbán P, *et al.* The chronic kidney disease epidemic in El Salvador: A cross-sectional study. *MEDICC Rev* 2019; **21**: 29–37.

90 Mufunda J., Mebrahtu G., Usman A., *et al.* The prevalence of hypertension and its relationship with obesity: Results from a national blood pressure survey in Eritrea. *Journal of Human Hypertension* 2006; **20**: 59–65.

91 Giday A., Tadesse B. Prevalence and determinants of hypertension in rural and urban areas of southern Ethiopia. *Ethiopian medical journal* 2011; **49**: 139–47.

92 Muluneh AT, Haileamlak A, Tessema F, *et al.* Population based survey of chronic non-communicable diseases at gilgel gibe field research center, southwest ethiopia. *Ethiop J Health Sci* 2012; **22**: 7–18.

93 Mengistu M.D. Pattern of blood pressure distribution and prevalence of hypertension and prehypertension among adults in Northern Ethiopia: Disclosing the hidden burden. *BMC Cardiovascular Disorders* 2014; **14**. DOI:10.1186/1471-2261-14-33.

94 Abebe S.M., Berhane Y., Worku A., Getachew A. Prevalence and associated factors of hypertension: A crossectional community based study in Northwest Ethiopia. *PLoS ONE* 2015; **10**. DOI:10.1371/journal.pone.0125210.

95 Gebreyes YF, Goshu DY, Geletew TK, *et al.* Prevalence of high bloodpressure, hyperglycemia, dyslipidemia, metabolic syndrome and their determinants in Ethiopia: Evidences from the National NCDs STEPS Survey, 2015. *PLoS ONE* 2018; **13**. DOI:10.1371/journal.pone.0194819.

96 Tesfaye TD, Temesgen WA, Kasa AS, Yismaw YS. Prevalence and associated factors of hypertension in Amhara regional state city and its' surrounding rural districts: A community-based cross-sectional study. *Afr Health Sci* 2019; **19**: 2580–90.

97 van der Sande M, Milligan P, Nyan O, *et al.* Blood pressure patterns and cardiovascular risk factors in rural and urban Gambian communities. *J Hum Hypertens* 2000; **14**: 489–96.

98 Cham B, Scholes S, Ng Fat L, Badjie O, Mindell JS. Burden of hypertension in The Gambia: evidence from a national World Health Organization (WHO) STEP survey. *International journal of epidemiology* 2018. DOI:10.1093/ije/dyx279.

99 Agyemang C. Rural and urban differences in blood pressure and hypertension in Ghana, West Africa. *Public Health* 2006; **120**: 525–33.

100 Obirikorang C., Osakunor D.N.M., Anto E.O., Amponsah S.O., Adarkwa O.K. Obesity and cardio-metabolic risk factors in an urban and rural population in the Ashanti region-Ghana: A comparative cross-sectional study. *PLoS ONE* 2015; **10**. DOI:10.1371/journal.pone.0129494.

101 Kodaman N., Aldrich M.C., Sobota R., *et al.* Cardiovascular disease risk factors in Ghana during the rural-to-urban transition: A cross-sectional study. *PLoS ONE* 2016; **11**. DOI:10.1371/journal.pone.0162753.

102 Agyemang C., Nyaaba G., Beune E., *et al.* Variations in hypertension awareness, treatment, and control among Ghanaian migrants living in Amsterdam, Berlin, London, and nonmigrant Ghanaians living in rural and urban Ghana-the RODAM study. *Journal of Hypertension* 2018; **36**: 169–77.

103 Sanuade OA, Boatemaa S, Kushitor MK. Hypertension prevalence, awareness, treatment and control in Ghanaian population: Evidence from the Ghana demographic and health survey. *PLOS ONE* 2018; **13**. DOI:10.1371/journal.pone.0205985.

104 Baldé N.-M., Diallo I., Baldé M.-D., *et al.* Diabetes and impaired fasting glucose in rural and urban populations in Futa Jallon (Guinea): prevalence and associated risk factors. *Diabetes and Metabolism* 2007; **33**: 114–20.

105 Camara A, Baldé NM, Diakité M, *et al.* High prevalence, low awareness, treatment and control rates of hypertension in Guinea: Results from a population-based STEPS survey. *Journal of Human Hypertension* 2016; **30**: 237–44.

106 DeGennaro Jr V, Malcolm S, Crompton L, *et al.* Community-based diagnosis of non-communicable diseases and their risk factors in rural and urban Haiti: a cross-sectional prevalence study. *BMJ open* 2018; **8**: e020317–e020317.

107 Singh R.B., Sharma J.P., Rastogi V., *et al.* Prevalence of coronary artery disease and coronary risk factors in rural and urban populations of north India. *European Heart Journal* 1997; **18**: 1728–35.

108 Kusuma Y.S., Babu B.V., Naidu J.M. Prevalence of hypertension in some cross-cultural populations of Visakhapatnam district, South India. *Ethnicity and Disease* 2004; **14**: 250–9.

109 Kumar R., Singh M.C., Ahlawat S.K., *et al.* Urbanization and coronary heart disease: A study of urban-rural differences in northern India. *Indian Heart Journal* 2006; **58**: 126–30.

110 Kusuma Y.S., Das P.K. Hypertension in Orissa, India: a cross-sectional study among some tribal, rural and urban populations. *Public Health* 2008; **122**: 1120–3.

111 Gupta R, Gupta VP. Hypertension epidemiology in India: Lessons from Jaipur heart watch. *Current Science* 2009; **97**: 349–55.

112 Midha T., Idris M.Z., Saran R.K., Srivastav A.K., Singh S.K. Prevalence and determinants of hypertension in the urban and rural population of a north Indian district. *East African journal of public health* 2009; **6**: 268–73.

113 Allender S., Lacey B., Webster P., *et al.* Level of urbanization and noncommunicable disease risk factors in Tamil Nadu, India. *Bulletin of the World Health Organization* 2010; **88**: 297–304.

114 Thankappan KR, Shah B, Mathur P, *et al.* Risk factor profile for chronic non-communicable diseases: Results of a community-based study in Kerala, India. *Indian Journal of Medical Research* 2010; **131**: 53–63.

115 Das M., Pal S., Ghosh A. Prevalence of cardiovascular disease risk factors by habitat: A study on adult Asian Indians in West Bengal, India. *Anthropologischer Anzeiger* 2011; **68**: 253–64.

116 Bharati D.R., Nandi P., Yamuna T.V., *et al.* Prevalence and covariates of undiagnosed hypertension in the adult population of Puducherry, South India. *Nepal Journal of Epidemiology* 2012; **2**: 191–9.

117 Samuel P., Antonisamy B., Raghupathy P., Richard J., Fall C.H.D. Socio-economic status and cardiovascular risk factors in rural and urban areas of Vellore, Tamilnadu, South India. *International Journal of Epidemiology* 2012; **41**: 1315–27.

118 Bhagyalaxmi A., Atul T., Shikha J. Prevalence of risk factors of non-communicable diseases in a district of Gujarat, India. *Journal of Health, Population and Nutrition* 2013; **31**: 78–85.

119 Millett C., Agrawal S., Sullivan R., *et al.* Associations between Active Travel to Work and Overweight, Hypertension, and Diabetes in India: A Cross-Sectional Study. *PLoS Medicine* 2013; **10**. DOI:10.1371/journal.pmed.1001459.

120 Bhadoria AS, Kasar PK, Toppo NA, Bhadoria P, Pradhan S, Kabirpanthi V. Prevalence of hypertension and associated cardiovascular risk factors in Central India. *Journal of family & community medicine* 2014; **21**: 29–38.

121 Farag YMK, Mittal BV, Keithi-Reddy SR, *et al.* Burden and predictors of hypertension in India: results of SEEK (Screening and Early Evaluation of Kidney Disease) study. *Bmc Nephrology* 2014; **15**. DOI:10.1186/1471-2369-15-42.

122 Bhansali A., Dhandania V.K., Deepa M., *et al.* Prevalence of and risk factors for hypertension in urban and rural India: The ICMR-INDIAB study. *Journal of Human Hypertension* 2015; **29**: 204–9.

123 Norboo T., Stobdan T., Tsering N., *et al.* Prevalence of hypertension at high altitude: Cross-sectional survey in Ladakh, Northern India 2007-2011. *BMJ Open* 2015; **5**. DOI:10.1136/bmjopen-2014-007026.

124 Krishnan M.N., Zachariah G., Venugopal K., *et al.* Prevalence of coronary artery disease and its risk factors in Kerala, South India: A community-based cross-sectional study. *BMC Cardiovascular Disorders* 2016; **16**. DOI:10.1186/s12872-016-0189-3.

125 Oommen A.M., Abraham V.J., George K., Jose V.J. Prevalence of risk factors for non-communicable diseases in rural & urban Tamil Nadu. *Indian Journal of Medical Research* 2016; **144**: 460–71.

126 Oommen A.M., Abraham V.J., George K., Jose V.J. Rising trend of cardiovascular risk factors between 1991-1994 and 2010-2012: A repeat cross sectional survey in urban and rural Vellore. *Indian Heart Journal* 2016; **68**: 263–9.

127 Bandela P.V., Dongre N.N., Ambekar J.G., Prasad K.D., Devaranavadagi B.B. Study of metabolic syndrome and its components among Kurnool district population of Andhra Pradesh with different ethnic backgrounds. *Journal of Cardiovascular Disease Research* 2017; **8**: 83–8.

128 Kanungo S., Mahapatra T., Bhowmik K., *et al.* Patterns and predictors of undiagnosed and uncontrolled hypertension: Observations from a poor-resource setting. *Journal of Human Hypertension* 2017; **31**: 56–65.

129 Prabhakaran D., Roy A., Praveen P.A., *et al.* 20-Year Trend of CVD Risk Factors: Urban and Rural National Capital Region of India. *Global Heart* 2017; **12**: 209–17.

130 Tripathy JP, Thakur JS, Jeet G, Chawla S, Jain S. Alarmingly high prevalence of hypertension and pre-hypertension in North India-results from a large cross-sectional STEPS survey. *Plos One* 2017; **12**. DOI:10.1371/journal.pone.0188619.

131 Geldsetzer P., Manne-Goehler J., Theilmann M., *et al.* Diabetes and hypertension in India a nationally representative study of 1.3 million adults. *JAMA Internal Medicine* 2018; **178**: 363–72.

132 Sarma PS, Sadanandan R, Thulaseedharan JV, *et al.* Prevalence of risk factors of non-communicable diseases in Kerala, India: Results of a cross-sectional study. *BMJ Open* 2019; **9**. DOI:10.1136/bmjopen-2018-027880.

133 Kokane AM, Joshi R, Kotnis A, *et al.* Determinants of behavioural and biological risk factors for cardiovascular diseases from state level STEPS survey (2017–19) in Madhya Pradesh. *PeerJ* 2020; **8**. DOI:10.7717/peerj.10476.

134 Mohanty S, Nagarathna R, Metri K, *et al.* Trends of Hypertension and Neurological Diseases in India: A Nationwide Survey Reporting the Distribution Across Geographical Areas. *Ann Neurosci* 2020; **27**: 162–8.

135 Kumar K, Misra S. Sex differences in prevalence and risk factors of hypertension in India: Evidence from the National Family Health Survey-4. *PLoS ONE* 2021; **16**. DOI:10.1371/journal.pone.0247956.

136 Mohanty SK, Pedgaonkar SP, Upadhyay AK, *et al.* Awareness, treatment, and control of hypertension in adults aged 45 years and over and their spouses in India: A nationally representative cross-sectional study. *PLoS Med* 2021; **18**. DOI:10.1371/journal.pmed.1003740.

137 Nanditha A, Susairaj P, Raghavan A, *et al.* Secular trends in cardiovascular risk factors among urban and rural populations in Tamil Nadu, India – An ancillary analysis of the STRiDE-I study. *Diabetes Res Clin Pract* 2021; **178**. DOI:10.1016/j.diabres.2021.108930.

138 Patel S, Patel P. Trend of hypertension in gujarat–understanding the nfhs-4 and nfhs-5 data. *Natl J Community Med* 2021; **12**: 8–10.

139 Sivanantham P, Sahoo J, Lakshminarayanan S, Bobby Z, Kar SS. Profile of risk factors for Non-Communicable Diseases (NCDs) in a highly urbanized district of India: Findings from Puducherry district-wide STEPS Survey, 2019–20. *PLoS ONE* 2021; **16**. DOI:10.1371/journal.pone.0245254.

140 Hussain MA, Al Mamun A, Reid C, Huxley RR. Prevalence, awareness, treatment and control of hypertension in Indonesian adults aged ≥40 years: Findings from the Indonesia Family Life Survey (IFLS). *PLoS ONE* 2016; **11**. DOI:10.1371/journal.pone.0160922.

141 Maharani A, Sujarwoto, Praveen D, Oceandy D, Tampubolon G, Patel A. Cardiovascular disease risk factor prevalence and estimated 10-year cardiovascular risk scores in Indonesia: The SMARThealth Extend study. *PLoS ONE* 2019; **14**. DOI:10.1371/journal.pone.0215219.

142 Sujarwoto S, Maharani A. Participation in community-based health care interventions (CBHIs) and its association with hypertension awareness, control and treatment in Indonesia. *PLoS ONE* 2020; **15**. DOI:10.1371/journal.pone.0244333.

143 SarrafZadegan N., AminiNik S. Blood pressure pattern in urban and rural areas in Isfahan, Iran. *Journal of Human Hypertension* 1997; **11**: 425–8.

144 Janghorbani M., Amini M., Gouya M.M., Delavari A., Alikhani S., Mahdavi A. Nationwide survey of prevalence and risk factors of prehypertension and hypertension in Iranian adults. *Journal of Hypertension* 2008; **26**: 419–26.

145 Azimi-Nezhad M., Ghayour-Mobarhan M., Safarian M., *et al.* Anthropometric indices of obesity and the prediction of cardiovascular risk factors in an Iranian population. *TheScientificWorldJournal* 2009; **9**: 424–30.

146 Ebrahimi M., Mansournia M.A., Haghdoost A.A., *et al.* Social disparities in prevalence, treatment and control of hypertension in Iran: Second National Surveillance of Risk Factors of Noncommunicable Diseases, 2006. *Journal of Hypertension* 2010; **28**: 1620–9.

147 Shirani S, Gharipour M, Khosravi A, *et al.* Gender differences in the prevalence of hypertension in a representative sample of iranian population: The Isfahan healthy heart program. *Acta Biomedica* 2011; **82**: 223–9.

148 Malekzadeh M.M., Etemadi A., Kamangar F., *et al.* Prevalence, awareness and risk factors of hypertension in a large cohort of Iranian adult population. *Journal of Hypertension* 2013; **31**: 1364–71.

149 Esteghamati A., Etemad K., Koohpayehzadeh J., *et al.* Awareness, treatment and control of pre-hypertension, and hypertension among adults in Iran. *Archives of Iranian Medicine* 2016; **19**: 456–64.

150 Khorrami Z, Etemad K, Yarahmadi S, *et al.* Urbanization and noncommunicable disease (NCD) risk factors: WHO STEPwise Iranian NCD risk factors surveillance in 2011. *Eastern Mediterranean Health Journal* 2017; **23**: 469–79.

151 Rajati F, Hamzeh B, Pasdar Y, *et al.* Prevalence, awareness, treatment, and control of hypertension and their determinants: Results from the first cohort of non-communicable diseases in a Kurdish settlement. *Sci Rep* 2019; **9**. DOI:10.1038/s41598-019-48232-y.

152 Katibeh M, Moghaddam AS, Yaseri M, Neupane D, Kallestrup P, Ahmadieh H. Hypertension and associated factors in the Islamic Republic of Iran: A population-based study. *East Mediterr Health J* 2020; **26**: 304–14.

153 Ahmadi A, Shirani M, Khaledifar A, *et al.* Non-communicable diseases in the southwest of Iran: profile and baseline data from the Shahrekord PERSIAN Cohort Study. *BMC Public Health* 2021; **21**: 2275.

154 Jalali SF, Javanian M, Ghadimi R, Bijani A, Mouodi S. Blood Pressure Screening Campaign in the Adult Population. *HEALTH SCOPE* 2021; **10**. DOI:10.5812/jhealthscope.110707.

155 Naghipour M, Joukar F, Salari A, Asgharnezhad M, Hassanipour S, Mansour-Ghanaei F. Epidemiologic profile of hypertension in Northern Iranian population: The PERSIAN guilan cohort study (PGCS). *Ann of Global Health* 2021; **87**: 1–13.

156 Rezaianzadeh A, Jafari F, Sadeghi SE, Rahimikazerooni S. The prevalence and predictors of pre-hypertension and hypertension in Kherameh cohort study: a population based study on 10,663 persons in south of Iran. *J Hum Hypertens* 2021; **35**: 257–64.

157 Allameh M, Ghanei Gheshlagh R, Rahmani K. Prevalence and Associated Risk Factors of Hypertension for the Middle-Aged Population (30–59 Years) in Iran: A National Cross-Sectional Study. *High Blood Press Cardiovasc Prev* 2022; **29**: 75–80.

158 Ferguson TS, Francis DK, Tulloch-Reid MK, Younger NOM, McFarlane SR, Wilks RJ. An update on the burden of cardiovascular disease risk factors in Jamaica findings from the Jamaica health and lifestyle survey 2007-2008. *West Indian Medical Journal* 2011; **60**: 422–8.

159 Supiyev A., Kossumov A., Kassenova A., *et al.* Diabetes prevalence, awareness and treatment and their correlates in older persons in urban and rural population in the Astana region, Kazakhstan. *Diabetes Research and Clinical Practice* 2016; **112**: 6–12.

160 Mathenge W., Foster A., Kuper H. Urbanization, ethnicity and cardiovascular risk in a population in transition in Nakuru, Kenya: a population-based survey. *BMC public health* 2010; **10**: 569.

161 Walekhwa SN, Kisa A. Tobacco Use and Risk Factors for Hypertensive Individuals in Kenya. *Healthcare* 2021; **9**: 591.

162 Fahs I, Khalife Z, Malaeb D, Iskandarani M, Salameh P. The Prevalence and Awareness of Cardiovascular Diseases Risk Factors among the Lebanese Population: A Prospective Study Comparing Urban to Rural Populations. *Cardiology Research and Practice* 2017. DOI:10.1155/2017/3530902.

163 Ratovoson R., Rasetarinera O.R., Andrianantenaina I., Rogier C., Piola P., Pacaud P. Hypertension, a neglected disease in rural and urban areas in Moramanga, Madagascar. *PLoS ONE* 2015; **10**. DOI:10.1371/journal.pone.0137408.

164 Msyamboza K.P., Ngwira B., Dzowela T., *et al.* The burden of selected chronic non-communicable diseases and their risk factors in malawi: Nationwide steps survey. *PLoS ONE* 2011; **6**. DOI:10.1371/journal.pone.0020316.

165 Price A.J., Crampin A.C., Amberbir A., *et al.* Prevalence of obesity, hypertension, and diabetes, and cascade of care in sub-Saharan Africa: a cross-sectional, population-based study in rural and urban Malawi. *The Lancet Diabetes and Endocrinology* 2018; **6**: 208–22.

166 Mohamud W.N.W., Ismail A.A.S., Khir A.S.M., *et al.* Prevalence of metabolic syndrome and its risk factors in adult Malaysians: Results of a nationwide survey. *Diabetes Research and Clinical Practice* 2012; **96**: 91–7.

167 Abdul-Razak S., Daher A.M., Ramli A.S., *et al.* Prevalence, awareness, treatment, control and socio demographic determinants of hypertension in Malaysian adults. *BMC public health* 2016; **16**: 351.

168 Naing C, Yeoh PN, Wai VN, Win NN, Kuan LP, Aung K. Hypertension in Malaysia: An analysis of trends from the national surveys 1996 to 2011. *Medicine (United States)* 2016; **95**. DOI:10.1097/MD.0000000000002417.

169 Naidu BM, Yusoff MFM, Abdullah S, *et al.* Factors associated with the severity of hypertension among Malaysian adults. *PLoS ONE* 2019; **14**. DOI:10.1371/journal.pone.0207472.

170 Isa ZM, Ibrahim N, Ismail NH, Jaafar MH, Tamil AM, Yusof KH. Dietary sodium intake and its association with hypertension: A cross-sectional study in selangor, malaysia. *J Pak Med Assoc* 2021; **71**: S68–73.

171 Bâ H.O., Camara Y., Menta I., *et al.* Hypertension and Associated Factors in Rural and Urban Areas Mali: Data from the STEP 2013 Survey. *International Journal of Hypertension* 2018; **2018**. DOI:10.1155/2018/6959165.

172 Guerrero-Romero F., Rodríguez-Morán M., Sandoval-Herrrera F., Alvarado-Ruiz R. Prevalence of hypertension in indigenous inhabitants of traditional communities from the north of Mexico. *Journal of Human Hypertension* 2000; **14**: 555–9.

173 Beltrán-Sánchez H., Crimmins E.M., Teruel G.M., Thomas D. Links between childhood and adult social circumstances and obesity and hypertension in the Mexican population. *Journal of aging and health* 2011; **23**: 1141–65.

174 Hosey GM, Samo M, Gregg EW, Barker L, Padden D, Bibb SG. Association of Socioeconomic Position and Demographic Characteristics with Cardiovascular Disease Risk Factors and Healthcare Access among Adults Living in Pohnpei, Federated States of Micronesia. *International journal of chronic diseases* 2014; **2014**: 595678–595678.

175 Pengpid S, Peltzer K. National trends in metabolic syndrome among adults in Mongolia from three cross-sectional surveys in 2009, 2013 and 2019. *Diabetes Metab Syndr Clin Res Rev* 2022; **16**. DOI:10.1016/j.dsx.2021.102375.

176 Tazi M.A., Abir-Khalil S., Chaouki N., *et al.* Prevalence of the main cardiovascular risk factors in Morocco: Results of a National Survey, 2000. *Journal of Hypertension* 2003; **21**: 897–903.

177 Ziyyat A, Ramdani N, Bouanani NEH, *et al.* Epidemiology of hypertension and its relationship with type 2 diabetes and obesity in eastern Morocco. *SpringerPlus* 2014; **3**. DOI:10.1186/2193-1801-3-644.

178 Pengpid S, Peltzer K. Prevalence and correlates of the metabolic syndrome in a cross-sectional community-based sample of 18–100 year-olds in Morocco: Results of the first national STEPS survey in 2017. *Diabetes Metab Syndr Clin Res Rev* 2020; **14**: 1487–93.

179 Damasceno A., Azevedo A., Silva-Matos C., Prista A., Diogo D., Lunet N. Hypertension prevalence, awareness, treatment, and control in mozambique: Urban/rural gap during epidemiological transition. *Hypertension* 2009; **54**: 77–83.

180 Jessen N, Damasceno A, Silva-Matos C, *et al.* Hypertension in Mozambique: trends between 2005 and 2015. *Journal of Hypertension* 2018; **36**: 779–84.

181 Mika M, Kenneth S, Orvalho A, *et al.* The prevalence of hypertension and its distribution by sociodemographic factors in Central Mozambique: a cross sectional study. *BMC Public Health* 2020; **20**. DOI:10.1186/s12889-020-09947-0.

182 Quasem I., Shetye M.S., Alex S.C., *et al.* Prevalence, awareness, treatment and control of hypertension among the elderly in Bangladesh and India: A multicentre study. *Bulletin of the World Health Organization* 2001; **79**: 490–500.

183 Prince M.J., Ebrahim S., Acosta D., *et al.* Hypertension prevalence, awareness, treatment and control among older people in Latin America, India and China: A 10/66 cross-sectional population-based survey. *Journal of Hypertension* 2012; **30**: 177–87.

184 Basu S., Millett C. Social epidemiology of hypertension in middle-income countries: Determinants of prevalence, diagnosis, treatment, and control in the WHO SAGE study. *Hypertension* 2013; **62**: 18–26.

185 Chow C.K., Teo K.K., Rangarajan S., *et al.* Prevalence, awareness, treatment, and control of hypertension in rural and urban communities in high-, middle-, and low-income countries. *JAMA - Journal of the American Medical Association* 2013; **310**: 959–68.

186 Harhay M.O., Harhay J.S., Nair M.M. Education, household wealth and blood pressure in Albania, Armenia, Azerbaijan and Ukraine: Findings from the Demographic Health Surveys, 2005-2009. *European Journal of Internal Medicine* 2013; **24**: 117–26.

187 Kavishe B., Biraro S., Baisley K., *et al.* High prevalence of hypertension and of risk factors for non-communicable diseases (NCDs): A population based cross-sectional survey of NCDS and HIV infection in Northwestern Tanzania and Southern Uganda. *BMC Medicine* 2015; **13**. DOI:10.1186/s12916-015-0357-9.

188 Gupta R., Kaur M., Islam S., *et al.* Association of household wealth index, educational status, and social capital with hypertension awareness, treatment, and control in South Asia. *American Journal of Hypertension* 2017; **30**: 373–81.

189 Bjertness M.B., Htet A.S., Meyer H.E., *et al.* Prevalence and determinants of hypertension in Myanmar - a nationwide cross-sectional study. *BMC public health* 2016; **16**: 590.

190 Htet A.S., Bjertness M.B., Oo W.M., *et al.* Changes in prevalence, awareness, treatment and control of hypertension from 2004 to 2014 among 25-74-year-old citizens in the Yangon Region, Myanmar. *BMC public health* 2017; **17**: 847.

191 Craig LS, Gage AJ, Thomas AM. Prevalence and predictors of hypertension in Namibia: A national-level cross-sectional study. *PLoS ONE* 2018; **13**. DOI:10.1371/journal.pone.0204344.

192 Aryal K.K., Mehata S., Neupane S., *et al.* The burden and determinants of non communicable diseases risk factors in Nepal: Findings from a nationwide STEPS survey. *PLoS ONE* 2015; **10**. DOI:10.1371/journal.pone.0134834.

193 Mehata S, Shrestha N, Mehta R, *et al.* Prevalence, awareness, treatment and control of hypertension in Nepal: data from nationally representative population-based cross-sectional study. *Journal of hypertension* 2018. DOI:10.1097/HJH.0000000000001745.

194 Laux T.S., Bert P.J., González M., Unruh M., Aragon A., Lacourt C.T. Prevalence of hypertension and associated risk factors in six Nicaraguan communities. *Ethnicity and Disease* 2012; **22**: 129–35.

195 Ezenwaka C.E., Akanji A.O., Akanji B.O., Unwin N.C., Adejuwon C.A. The prevalence of insulin resistance and other cardiovascular disease risk factors in healthy elderly southwestern Nigerians. *Atherosclerosis* 1997; **128**: 201–11.

196 Okosun IS, Forrester TE, Rotimi CN, Osotimehin BO, Muna WF, Cooper RS. Abdominal adiposity in six populations of West African descent: prevalence and population attributable fraction of hypertension. *Obes Res* 1999; **7**: 453–62.

197 Isezuo SA, Sabir AA, Ohwovorilole AE, Fasanmade OA. Prevalence, associated factors and relationship between prehypertension and hypertension: a study of two ethnic African populations in Northern Nigeria. *J Hum Hypertens* 2011; **25**: 224–30.

198 Abegunde K., Owoaje E. Health problems and associated risk factors in selected urban and rural elderly population groups of South-West Nigeria. *Annals of African Medicine* 2013; **12**: 90–7.

199 Adediran O.S., Adebayo P.B., Akintunde A.A. Anthropometric differences among natives of Abuja living in urban and rural communities: correlations with other cardiovascular risk factors. *BMC research notes* 2013; **6**: 123.

200 Ejim E.C., Onwubere B.J., Okafor C.I., *et al.* Cardiovascular risk factors in middle-aged and elderly residents in South-East Nigeria: the influence of urbanization. *Nigerian journal of medicine : journal of the National Association of Resident Doctors of Nigeria* 2013; **22**: 286–91.

201 Murthy G.V.S., Fox S., Sivasubramaniam S., *et al.* Prevalence and risk factors for hypertension and association with ethnicity in Nigeria: Results from a national survey. *Cardiovascular Journal of Africa* 2013; **24**: 344–50.

202 Okpechi I.G., Chukwuonye I.I., Tiffin N., *et al.* Blood Pressure Gradients and Cardiovascular Risk Factors in Urban and Rural Populations in Abia State South Eastern Nigeria Using the WHO STEPwise Approach. *PLoS ONE* 2013; **8**. DOI:10.1371/journal.pone.0073403.

203 Oguoma V.M., Nwose E.U., Skinner T.C., Digban K.A., Onyia I.C., Richards R.S. Prevalence of cardiovascular disease risk factors among a Nigerian adult population: relationship with income level and accessibility to CVD risks screening. *BMC public health* 2015; **15**: 397.

204 Odili AN, Chori BS, Danladi B, *et al.* Prevalence, awareness, treatment and control of hypertension in Nigeria: Data from a nationwide survey 2017. *Glo Heart* 2020; **15**. DOI:10.5334/GH.848.

205 Umuerri EM, Aiwuyo HO. Prevalence and correlates of prehypertension and hypertension among adults in Delta State, Nigeria: A cross-sectional community-based study. *Ghana Med J* 2020; **54**: 48–57.

206 Jafar T.H., Levey A.S., Jafary F.H., *et al.* Ethnic subgroup differences in hypertension in Pakistan. *Journal of Hypertension* 2003; **21**: 905–12.

207 Tareen M.F., Shafique K., Mirza S.S., Arain Z.I., Ahmad I., Vart P. Location of residence or social class, which is the stronger determinant associated with cardiovascular risk factors among Pakistani population? A cross sectional study. *Rural and remote health* 2011; **11**: 1700.

208 Basit A, Tanveer S, Fawwad A, Naeem N, NDSP Members. Prevalence and contributing risk factors for hypertension in urban and rural areas of Pakistan; a study from second National Diabetes Survey of Pakistan (NDSP) 2016–2017. *Clin Exp Hypertens* 2020; **42**: 218–24.

209 Mc Donald Posso AJ, Motta Borrel JA, Fontes F, Cruz Gonzalez CE, Pachón Burgos AA, Ortega AC. High blood pressure in Panama: Prevalence, sociodemographic and biologic profile, treatment, and control (STROBE). *Medicine (United States)* 2014; **93**. DOI:10.1097/MD.0000000000000101.

210 Miranda J.J., Gilman R.H., Smeeth L. Differences in cardiovascular risk factors in rural, urban and rural-to-urban migrants in Peru. *Heart* 2011; **97**: 787–96.

211 Bernabé-Ortiz A., Carrillo-Larco R.M., Gilman R.H., *et al.* Impact of urbanisation and altitude on the incidence of, and risk factors for, hypertension. *Heart* 2017; **103**: 827–33.

212 Chambergo-Michilot D, Rebatta-Acuña A, Delgado-Flores CJ, Toro-Huamanchumo CJ. Socioeconomic determinants of hypertension and prehypertension in Peru: Evidence from the peruvian demographic and health survey. *PLoS ONE* 2021; **16**. DOI:10.1371/journal.pone.0245730.

213 Dorobantu M., Darabont R.O., Badila E., Ghiorghe S. Prevalence, awareness, treatment, and control of hypertension in romania: Results of the SEPHAR study. *International Journal of Hypertension* 2010; **2010**. DOI:10.4061/2010/970694.

214 Dorobanţu M, Darabont R, Ghiorghe S, *et al.* Profile of the Romanian hypertensive patient data from SEPHAR II study. *Romanian journal of internal medicine = Revue roumaine de médecine interne* 2012; **50**: 285–96.

215 Artyukhov I.P., Grinshtein Y.I., Petrova M.M., Shabalin V.V., Ruf R.R. Prevalence of arterial hypertension in the Krasnoyarsk Krai (Siberia, Russia). *BMC Cardiovascular Disorders* 2017; **17**. DOI:10.1186/s12872-017-0559-5.

216 Balanova YA, Shalnova SA, Imaeva AE, *et al.* Prevalence, awareness, treatment and control of hypertension in Russian Federation (data of observational ESSE- RF-2 study). *Ration Pharmacother Cardiol* 2019; **15**: 450–66.

217 Nahimana M.-R., Nyandwi A., Muhimpundu M.A., *et al.* A population-based national estimate of the prevalence and risk factors associated with hypertension in Rwanda: implications for prevention and control. *BMC public health* 2017; **18**: 2.

218 Seck S.M., Doupa D., Guéye L., Ba I. Chronic kidney disease epidemiology in Northern Senegal: A cross-sectional study. *Iranian Journal of Kidney Diseases* 2014; **8**: 286–91.

219 Lovic D., Stojanov V., Jakovljević B., *et al.* Prevalence of arterial hypertension in Serbia: PAHIS study. *Journal of Hypertension* 2013; **31**: 2151–7.

220 Marinković M., Ilić N., Djokić D., *et al.* Prevalence of hypertension in adults in the Šumadija District, Serbia - A cross-sectional study. *Vojnosanitetski Pregled* 2014; **71**: 245–50.

221 Odland ML, Bockarie T, Wurie H, *et al.* Prevalence and access to care for cardiovascular risk factors in older people in Sierra Leone: a cross-sectional survey. *BMJ Open* 2020; **10**: e038520.

222 Geraedts TJM, Boateng D, Lindenbergh KC, *et al.* Evaluating the cascade of care for hypertension in Sierra Leone. *Trop Med Int Health* 2021; **26**: 1470–80.

223 Van Rooyen J.M., Kruger H.S., Huisman H.W., *et al.* An epidemiological study of hypertension and its determinants in a population in transition: The THUSA study. *Journal of Human Hypertension* 2000; **14**: 779–87.

224 van Zyl S, van der Merwe LJ, Walsh CM, Groenewald AJ, van Rooyen FC. Risk-factor profiles for chronic diseases of lifestyle and metabolic syndrome in an urban and rural setting in South Africa. *African Journal of Primary Health Care and Family Medicine* 2012; **4**. DOI:10.4102/phcfm.v4i1.346.

225 Kandala N-B, Tigbe W, Manda SO, Stranges S. Geographic variation of hypertension in sub-Saharan Africa: A case study of South Africa. *American Journal of Hypertension* 2013; **26**: 382–91.

226 Dolman RC, Wentzel-Viljoen E, Jerling JC, Feskens EJM, Kruger A, Pieters M. The use of predefined diet quality scores in the context of CVD risk during urbanization in the South African Prospective Urban and Rural Epidemiological (PURE) study. *Public Health Nutrition* 2014; **17**: 1706–16.

227 Egbujie B.A., Igumbor E.U., Puoane T. A cross-sectional study of socioeconomic status and cardiovascular disease risk among participants in the prospective Urban Rural Epidemiological (PURE) study. *South African Medical Journal* 2016; **106**: 900–6.

228 Kandala N-B, Nnanatu CC, Dukhi N, Sewpaul R, Davids A, Reddy SP. Mapping the burden of hypertension in south africa: A comparative analysis of the national 2012 sanhanes and the 2016 demographic and health survey. *Int J Environ Res Public Health* 2021; **18**. DOI:10.3390/ijerph18105445.

229 Katulanda P., Ranasinghe P., Jayawardena R., Constantine G.R., Rezvi Sheriff M.H., Matthews D.R. The prevalence, predictors and associations of hypertension in Sri Lanka: A cross-sectional population based national survey. *Clinical and Experimental Hypertension* 2014; **36**: 484–91.

230 Krishnadath ISK, Jaddoe VWV, Nahar-van Venrooij LM, Toelsie JR. Ethnic differences in prevalence and risk factors for hypertension in the Suriname Health Study: A cross sectional population study. *Population Health Metrics* 2016; **14**. DOI:10.1186/s12963-016-0102-4.

231 Edwards R., Unwin N., Mugusi F., *et al.* Hypertension prevalence and care in an urban and rural area of Tanzania. *Journal of Hypertension* 2000; **18**: 145–52.

232 Stanifer J.W., Egger J.R., Turner E.L., Thielman N., Patel U.D. Neighborhood clustering of non-communicable diseases: results from a community-based study in Northern Tanzania. *BMC public health* 2016; **16**: 226.

233 Mosha NR, Mahande M, Juma A, *et al.* Prevalence, awareness and factors associated with hypertension in North West Tanzania. *Global Health Action* 2017; **10**. DOI:10.1080/16549716.2017.1321279.

234 Suriyawongpaisal P. Cardiovascular risk factor levels in urban and rural Thailand - The International Collaborative Study of Cardiovascular Disease in Asia (InterASIA). *European Journal of Cardiovascular Prevention and Rehabilitation* 2003; **10**: 249–57.

235 Aekplakorn W, Sangthong R, Kessomboon P, *et al.* Changes in prevalence, awareness, treatment and control of hypertension in Thai population, 2004-2009: Thai National Health Examination Survey III-IV. *J Hypertens* 2012; **30**: 1734–42.

236 Bouguerra R., Ben Salem L., Alberti H., *et al.* Prevalence of metabolic abnormalities in the Tunisian adults: A population based study. *Diabetes and Metabolism* 2006; **32**: 215–21.

237 Hammami S., Mehri S., Hajem S., *et al.* Awareness, treatment and control of hypertension among the elderly living in their home in Tunisia. *BMC Cardiovascular Disorders* 2011; **11**. DOI:10.1186/1471-2261-11-65.

238 Aounallah-Skhiri H., El Ati J., Traissac P., *et al.* Blood pressure and associated factors in a North African adolescent population. a national cross-sectional study in Tunisia. *BMC public health* 2012; **12**: 98.

239 Ben Romdhane H., Ben Ali S., Skhiri H., *et al.* Hypertension among Tunisian adults: Results of the TAHINA project. *Hypertension Research* 2012; **35**: 341–7.

240 Sönmez HM, Başak O, Camci C, *et al.* The epidemiology of elevated blood pressure as an estimate for hypertension in Aydin, Turkey. *Journal of Human Hypertension* 1999; **13**: 399–404.

241 Altun B., Arici M., Nergizoğlu G., *et al.* Prevalence, awareness, treatment and control of hypertension in Turkey (the PatenT study) in 2003. *Journal of Hypertension* 2005; **23**: 1817–23.

242 Metintas S., Arikan I., Kalyoncu C. Awareness of hypertension and other cardiovascular risk factors in rural and urban areas in Turkey. *Transactions of the Royal Society of Tropical Medicine and Hygiene* 2009; **103**: 812–8.

243 Sengul S., Akpolat T., Erdem Y., *et al.* Changes in hypertension prevalence, awareness, treatment, and control rates in Turkey from 2003 to 2012. *Journal of Hypertension* 2016; **34**: 1208–17.

244 Daştan İ., Erem A., Çetinkaya V. Urban and rural differences in hypertension risk factors in turkey. *Anatolian Journal of Cardiology* 2017; **18**: 39–47.

245 Oğuz A, Telci Çaklılı Ö, Tümerdem Çalık B. The Prospective Urban Rural Epidemiology (PURE) study: PURE Turkey. *Turk Kardiyol Dern Ars* 2018; **46**: 613–23.

246 Musinguzi G, Nuwaha F. Prevalence, Awareness and Control of Hypertension in Uganda. *Plos One* 2013; **8**. DOI:10.1371/journal.pone.0062236.

247 Guwatudde D., Mutungi G., Wesonga R., *et al.* The epidemiology of hypertension in Uganda: Findings from the national non-communicable diseases risk factor survey. *PLoS ONE* 2015; **10**. DOI:10.1371/journal.pone.0138991.

248 Nakibuuka J., Sajatovic M., Nankabirwa J., *et al.* Stroke-risk factors differ between rural and urban communities: Population survey in central uganda. *Neuroepidemiology* 2015; **44**: 156–65.

249 Nieto-Martínez R, González-Rivas JP, Ugel E. Prevalence of cardiometabolic risk factors in three populations from Venezuela: the VEMSOLS STUDY 2006-2010. *Medicas UIS* 2018; **31**: 15–22.

250 Nguyen Q.N., Pham S.T., Nguyen V.L., *et al.* Time trends in blood pressure, body mass index and smoking in the Vietnamese population: A meta-analysis from multiple cross-sectional surveys. *PLoS ONE* 2012; **7**. DOI:10.1371/journal.pone.0042825.

251 Do H.T.P., Geleijnse J.M., Le M.B., Kok F.J., Feskens E.J.M. National prevalence and associated risk factors of hypertension and prehypertension among vietnamese adults. *American Journal of Hypertension* 2015; **28**: 89–97.

252 Jensen P.N., Bao T.Q., Huong T.T.T., *et al.* The association of estimated salt intake with blood pressure in a Viet Nam national survey. *PLoS ONE* 2018; **13**. DOI:10.1371/journal.pone.0191437.

253 Hoang VM, Tran QB, Vu THL, *et al.* Patterns of Raised Blood Pressure in Vietnam: Findings from the WHO STEPS Survey 2015. *Int J Hypertens* 2019; **2019**. DOI:10.1155/2019/1219783.

254 Abdul-Rahim H.F., Husseini A., Bjertness E., Giacaman R., Gordon N.H., Jervell J. The metabolic syndrome in the West Bank population: An urban-rural comparison. *Diabetes Care* 2001; **24**: 275–9.

255 Modesti P.A., Bamoshmoosh M., Rapi S., *et al.* Relationship between hypertension, diabetes and proteinuria in rural and urban households in Yemen. *Journal of Human Hypertension* 2013; **27**: 572–9.
