# Supplementary material for: Urban-rural differences in hypertension prevalence in low-income and middle-income countries, 1990–2020: A systematic review and meta-analysis
Source: PLoS Med. 2022 Aug 25;19(8):e1004079. doi: 10.1371/journal.pmed.1004079 (PMC9410549; doi:10.1371/journal.pmed.1004079)
Supplement: S3 Data — (PDF) [file pmed.1004079.s005.pdf]

S3 Data

Urban-rural differences in hypertension prevalence in low-income and middle-income countries, 1990-2020: a systematic review and meta-analysis

Table of Contents

Pooled prevalence of hypertension between urban and rural areas from 66 countries, by sex and time periods 2

Pooled prevalence of hypertension between urban and rural areas from 66 countries, by income status and region, 1990-2020 3

Pooled prevalence of hypertension between urban and rural areas from 66 countries, by income status, region, and time period 4

Sensitivity analysis for pooled prevalence of hypertension comparing random effects model versus generalized mixed model. 5

Pooled prevalence of hypertension between urban and rural areas from 66 countries, by sex and time periods

Model	Period / Moderator	Area	Sex	n	Prevalence of Hypertension (95% CI)	P-value for the moderator	I^2	tau^2	P-value for heterogeneity	R^2
**Overall**										
	Period 1990-2020*	Rural	All	291	27.9% (26.3, 29.6)		99.97%	0.01984	<0.001	
	Period 1990-2020*	Urban	All	291	30.5% (28.9, 32.0)		99.95%	0.01815	<0.001	
	Period 1990-2020*	Rural	Males	121	26.8% (24.4, 29.2)		99.85%	0.01708	<0.001	
	Period 1990-2020*	Urban	Males	121	30.6% (28.4, 32.8)		99.71%	0.01391	<0.001	
	Period 1990-2020*	Rural	Females	122	25.7% (23.1, 28.4)		99.94%	0.02165	<0.001	
	Period 1990-2020*	Urban	Females	122	28.8% (26.2, 31.5)		99.90%	0.02115	<0.001	
**By period**										
	Period 1990-2004*	Rural	All	291	23.8% (20.5, 27.1)	0.005	99.97%	0.01935	<0.001	2.47%
	Period 2005-2020*	Rural	All		29.3% (27.4, 31.1)					
	Period 1990-2004*	Urban	All	291	29.8% (26.6, 33.0)	0.612	99.94%	0.01820	<0.001	0.00%
	Period 2005-2020*	Urban	All		30.7% (28.9, 32.5)					
	Period 1990-2004*	Rural	Males	121	21.4% (17.5, 25.3)	0.001	99.73%	0.01563	<0.001	8.48%
	Period 2005-2020*	Rural	Males		29.7% (26.8, 32.5)					
	Period 1990-2004*	Urban	Males	121	28.1% (24.5, 31.8)	0.099	99.58%	0.01372	<0.001	1.38%
	Period 2005-2020*	Urban	Males		31.9% (29.2, 34.6)					
	Period 1990-2004*	Rural	Females	122	21.3% (16.8, 25.7)	0.016	99.91%	0.02076	<0.001	4.11%
	Period 2005-2020*	Rural	Females		28.1% (24.8, 31.3)					
	Period 1990-2004*	Urban	Females	122	26.7% (22.3, 31.2)	0.258	99.85%	0.02111	<0.001	0.20%
	Period 2005-2020*	Urban	Females		29.9% (26.7, 33.2)					

* For the overall estimates, we used the original PURE study containing data from 14 LMICs countries (n=126,624 participants). For the stratified analysis by income and region, we used data from 7 studies that reported data at country level for 9 countries from the original PURE study (n=104,196 participants, 9 surveys).

Pooled prevalence of hypertension between urban and rural areas from 66 countries, by income status and region, 1990-2020

Moderator	Period	Area	Sex	n	Prevalence of Hypertension (95% CI)	P-value for the moderator	I^2	tau^2	P-value for heterogeneity	R^2
LIC	1990-2020	Rural	All		21.9% (19.3, 24.6)					
LMIC	1990-2020	Rural	All	299	27.4% (24.9, 29.8)	<0.001	99.95%	0.01720	<0.001	15.57%
UMIC	1990-2020	Rural	All		36.3% (33.6, 39.1)					
East Asia & Pacific	1990-2020	Rural	All		28.9% (26.2, 31.7)					
Sub-Saharan Africa	1990-2020	Rural	All		26.3% (23.2, 29.5)					
South Asia	1990-2020	Rural	All		23.8% (20.3, 27.3)					
Middle East & North Africa	1990-2020	Rural	All	299	26.9% (21.6, 32.2)	<0.001	99.96%	0.01846	<0.001	9.39%
Europe & Central Asia	1990-2020	Rural	All		43.8% (37.8, 49.8)					
Latin America & Caribbean	1990-2020	Rural	All		31.2% (25.8, 36.6)					
LIC	1990-2020	Urban	All		27.7% (25.0, 30.4)					
LMIC	1990-2020	Urban	All	299	30.3% (27.8, 32.7)	0.001	99.92%	0.01787	<0.001	4.07%
UMIC	1990-2020	Urban	All		35.1% (32.3, 37.9)					
East Asia & Pacific	1990-2020	Urban	All		29.5% (26.7, 32.2)					
Sub-Saharan Africa	1990-2020	Urban	All		30.7% (27.5, 33.8)					
South Asia	1990-2020	Urban	All		31.5% (28.0, 35.0)					
Middle East & North Africa	1990-2020	Urban	All	299	27.6% (22.3, 32.9)	0.136	99.94%	0.01841	<0.001	1.18%
Europe & Central Asia	1990-2020	Urban	All		37.7% (31.7, 43.7)					
Latin America & Caribbean	1990-2020	Urban	All		33.3% (27.9, 38.7)					

Pooled prevalence of hypertension between urban and rural areas from 66 countries, by income status, region, and time period

Moderator	Period	Area	Sex	n	Prevalence of Hypertension (95% CI)	P-value for the moderator	I^2	tau^2	P-value for heterogeneity	R^2
LIC	1990-2004	Rural	All	299	20.0% (15.9, 24.2)	<0.001	99.95%	0.01723	<0.001	15.45%
LMIC	1990-2004	Rural	All		28.6% (23.7, 33.4)					
UMIC	1990-2004	Rural	All		30.5% (17.5, 43.6)					
LIC	2005-2020	Rural	All		23.3% (19.8, 26.8)					
LMIC	2005-2020	Rural	All		27.0% (24.1, 29.8)					
UMIC	2005-2020	Rural	All		36.6% (33.8, 39.4)					
East Asia & Pacific	1990-2004	Rural	All	299	23.3% (18.0, 28.7)	<0.001	99.95%	0.01816	<0.001	10.86%
Sub-Saharan Africa	1990-2004	Rural	All		21.1% (14.2, 28.0)					
South Asia	1990-2004	Rural	All		22.6% (16.3, 28.9)					
Middle East & North	1990-2004	Rural	All		26.2% (17.3, 35.1)					
Europe & Central Asia	1990-2004	Rural	All		34.1% (15.2, 53.1)					
Latin America &	1990-2004	Rural	All		35.9% (22.5, 49.2)					
East Asia & Pacific	2005-2020	Rural	All		30.9% (27.7, 34.1)					
Sub-Saharan Africa	2005-2020	Rural	All		27.7% (24.2, 31.2)					
South Asia	2005-2020	Rural	All		24.3% (20.2, 28.5)					
Middle East & North	2005-2020	Rural	All		27.3% (20.8, 33.8)					
Europe & Central Asia	2005-2020	Rural	All		44.9% (38.6, 51.2)					
Latin America &	2005-2020	Rural	All		30.3% (24.4, 36.2)					
LIC	1990-2004	Urban	All	299	28.6% (24.4, 32.8)	0.011	99.91%	0.01800	<0.001	3.37%
LMIC	1990-2004	Urban	All		30.8% (25.9, 35.7)					
UMIC	1990-2004	Urban	All		40.0% (26.7, 53.2)					
LIC	2005-2020	Urban	All		27.0% (23.5, 30.6)					
LMIC	2005-2020	Urban	All		30.1% (27.2, 33.0)					
UMIC	2005-2020	Urban	All		34.8% (32.0, 37.7)					
East Asia & Pacific	1990-2004	Urban	All	299	27.0% (21.6, 32.3)	0.189	99.91%	0.01837	<0.001	1.37%
Sub-Saharan Africa	1990-2004	Urban	All		29.1% (22.1, 36.0)					
South Asia	1990-2004	Urban	All		32.3% (26.0, 38.7)					
Middle East & North	1990-2004	Urban	All		28.5% (19.6, 37.4)					
Europe & Central Asia	1990-2004	Urban	All		30.8% (11.8, 49.7)					
Latin America &	1990-2004	Urban	All		46.4% (33.0, 59.8)					
East Asia & Pacific	2005-2020	Urban	All		30.3% (27.2, 33.5)					
Sub-Saharan Africa	2005-2020	Urban	All		31.1% (27.5, 34.6)					
South Asia	2005-2020	Urban	All		31.2% (27.0, 35.4)					
Middle East & North	2005-2020	Urban	All		27.1% (20.6, 33.6)					
Europe & Central Asia	2005-2020	Urban	All		38.5% (32.2, 44.8)					
Latin America &	2005-2020	Urban	All		30.8% (24.9, 36.7)					

Sensitivity analysis for pooled prevalence of hypertension comparing random effects model versus generalized mixed model.

Model	Period / Moderator	Area	Sex	Prevalence of Hypertension (95% CI)	
**Overall**				Random effects model	Generalized mixed model
	Period 1990-2020*	Rural	All	27.9% (26.3, 29.6)	26.1% (24.4, 27.8)
	Period 1990-2020*	Urban	All	30.5% (28.9, 32.0)	29.2% (27.5, 30.9)
	Period 1990-2020	Rural	Males	26.8% (24.4, 29.2)	26.4% (23.9, 29.2)
	Period 1990-2020	Urban	Males	30.6% (28.4, 32.8)	30.7% (28.3, 33.2)
	Period 1990-2020	Rural	Females	25.7% (23.1, 28.4)	25.2% (22.4, 28.2)
	Period 1990-2020	Urban	Females	28.8% (26.2, 31.5)	28.4% (25.7, 31.3)
**By period**					
	Period 1990-2004*	Rural	All	23.8% (20.5, 27.1)	21.5% (18.7, 24.6)
	Period 2005-2020*	Rural	All	29.3% (27.4, 31.1)	27.7% (25.7, 29.7)
	Period 1990-2004*	Urban	All	29.8% (26.6, 33.0)	28.1% (24.9, 31.6)
	Period 2005-2020*	Urban	All	30.7% (28.9, 32.5)	29.5% (27.6, 31.5)
	Period 1990-2004	Rural	Males	21.4% (17.5, 25.3)	20.9% (17.4, 24.8)
	Period 2005-2020	Rural	Males	29.7% (26.8, 32.5)	29.7% (26.5, 33.2)
	Period 1990-2004	Urban	Males	28.1% (24.5, 31.8)	28.7% (24.9, 32.8)
	Period 2005-2020	Urban	Males	31.9% (29.2, 34.6)	31.8% (28.8, 34.9)
	Period 1990-2004	Rural	Females	21.3% (16.8, 25.7)	20.6% (16.7, 25.0)
	Period 2005-2020	Rural	Females	28.1% (24.8, 31.3)	27.9% (24.3, 31.7)
	Period 1990-2004	Urban	Females	26.7% (22.3, 31.2)	26.7% (22.4, 31.6)
	Period 2005-2020	Urban	Females	29.9% (26.7, 33.2)	29.3% (25.9, 33.0)

* For the overall estimates, we used the original PURE study containing data from 14 LMICs countries (n=126,624 participants). For the stratified analysis by income and region, we used data from 7 studies that reported data at country level for 9 countries from the original PURE study (n=104,196 participants, 9 surveys)
