# Supplementary material for: Urban-rural differences in hypertension prevalence in low-income and middle-income countries, 1990–2020: A systematic review and meta-analysis
Source: PLoS Med. 2022 Aug 25;19(8):e1004079. doi: 10.1371/journal.pmed.1004079 (PMC9410549; doi:10.1371/journal.pmed.1004079)
Supplement: S4 Data — (PDF) [file pmed.1004079.s006.pdf]

S4 Data

Urban-rural differences in hypertension prevalence in low-income and middle-income countries, 1990-2020: a systematic review and meta-analysis

Table of Contents

Difference in hypertension prevalence between urban and rural areas from the 299 surveys included from 66 low-income and middle-income countries, by sex, income status and region 2

Difference in hypertension prevalence between urban and rural areas from the 299 surveys included from 66 low-income and middle-income countries, by sex, income status and region

Period	Category	n	Urban - Rural prevalence difference (95% CI)	P-value for the moderator	I^2	tau^2	P-value for heterogeneity	R^2
**Sex**								
Period 1990-2020	Males	121	3.90% (2.40, 5.40)		98.79%	0.00577	<0.001	
Period 1990-2020	Females	122	2.98% (1.65, 4.31)		99.22%	0.00477	<0.001	
**Sex by period**								
Period 1990-2004	Males	121	6.65% (4.21, 9.09)	0.006	98.01%	0.00539	<0.001	6.46%
Period 2005-2020	Males		2.40% (0.59, 4.21)					
Period 1990-2004	Females	122	5.35% (3.16, 7.55)	0.009	98.79%	0.00448	<0.001	6.12%
Period 2005-2020	Females		1.70% (0.09, 3.31)					
**Income status by period**								
1990-2004	LIC	40	8.16% (5.97, 10.35)	<0.001	99.40%	0.00441	<0.001	18.16%
1990-2004	LMIC	39	2.27% (-0.27, 4.81)					
1990-2004	UMIC	4	9.26% (2.37, 16.15)					
2005-2020	LIC	56	3.87% (2.01, 5.74)					
2005-2020	LMIC	85	2.90% (1.39, 4.41)					
2005-2020	UMIC	85	-1.72% (-3.21, -0.22)					
**Region by period**								
1990-2004	East Asia & Pacific	25	3.66% (1.07, 6.25)	<0.001	99.33%	0.00394	<0.001	26.76%
1990-2004	Sub-Saharan Africa	15	7.55% (4.10, 11.00)					
1990-2004	South Asia	18	9.33% (6.22, 12.45)					
1990-2004	Middle East & North Africa	9	2.41% (-1.91, 6.73)					
1990-2004	Europe & Central Asia	2	-3.31% (-12.72, 6.09)					
1990-2004	Latin America & Caribbean	4	10.35% (3.75, 16.95)					
2005-2020	East Asia & Pacific	71	3.66% (1.07, 6.25)					
2005-2020	Sub-Saharan Africa	58	7.55% (4.10, 11.00)					
2005-2020	South Asia	41	9.33% (6.22, 12.45)					
2005-2020	Middle East & North Africa	17	2.41% (-1.91, 6.73)					
2005-2020	Europe & Central Asia	18	-3.31% (-12.72, 6.09)					
2005-2020	Latin America & Caribbean	21	10.35% (3.75, 16.95)					

* For the overall estimates, we used the original PURE study containing data from 14 LMICs countries (n=126,624 participants). For the stratified analysis by income and region, we used data from 7 studies that reported data at country level for 9 countries from the original PURE study (n=104,196 participants, 9 surveys).
