# Supplementary material for: Urban-rural differences in hypertension prevalence in low-income and middle-income countries, 1990–2020: A systematic review and meta-analysis
Source: PLoS Med. 2022 Aug 25;19(8):e1004079. doi: 10.1371/journal.pmed.1004079 (PMC9410549; doi:10.1371/journal.pmed.1004079)
Supplement: S5 Data — (PDF) [file pmed.1004079.s007.pdf]

S5 Data

Urban-rural differences in hypertension prevalence in low-income and middle-income countries, 1990-2020: a systematic review and meta-analysis

Table of Contents

Systolic and diastolic average blood pressure distribution 2

Urban and rural difference in systolic and diastolic blood pressure 3

Systolic and diastolic average blood pressure distribution

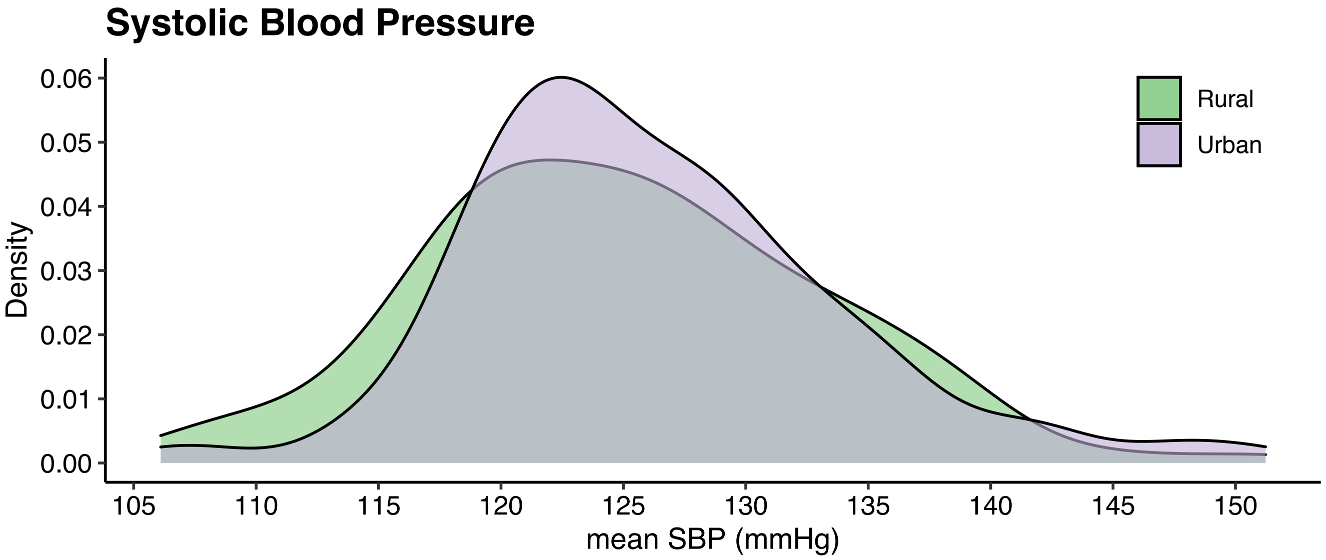

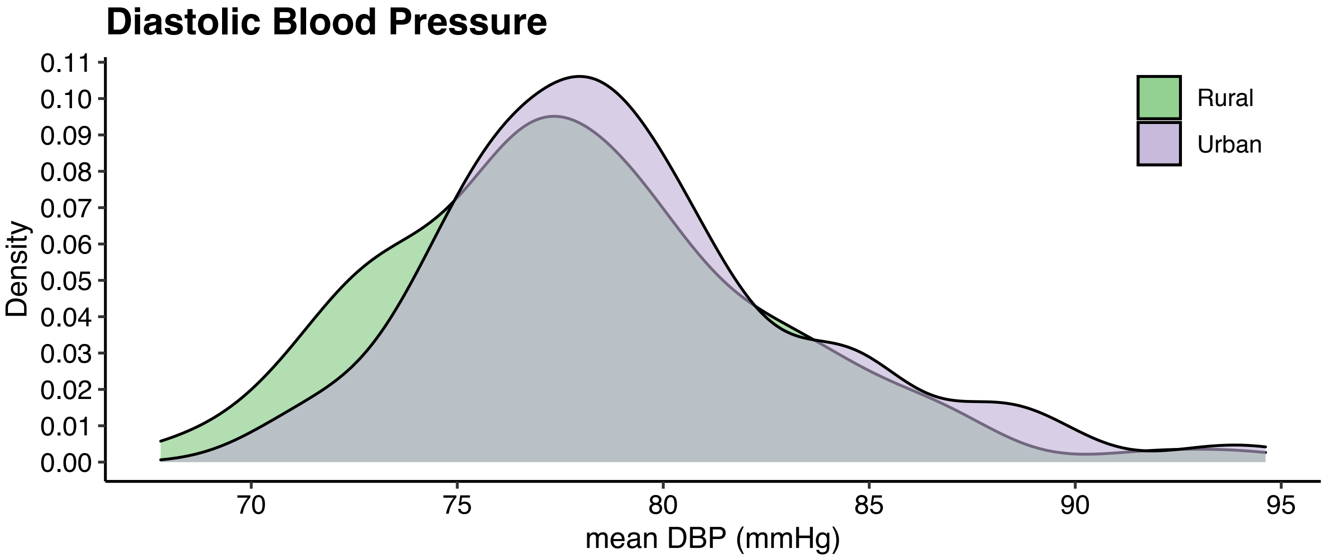

DBP – diastolic blood pressure; SBP – systolic blood pressure

Urban and rural difference in systolic and diastolic blood pressure

	All 1990-2020	Period 1990-2004	Period 2005-2020	All 1990-2020	Period 1990-2004	Period 2005-2020
	Difference U-R SBP (mmHg)	Difference U-R SBP (mmHg)	Difference U-R SBP (mmHg)	Difference U-R DBP (mmHg)	Difference U-R DBP (mmHg)	Difference U-R DBP (mmHg)
**Overall (n = 105)**	0.99 (-0.03, 2.02)	2.34 (0.60, 4.09)	0.30 (-0.95, 1.55)	1.11 (0.51, 1.70)	1.89 (0.88, 2.90)	0.71 (-0.01, 1.43)
**By income status (n = 105)**						
LIC (n=41)	2.08 (0.46, 3.70)			1.84 (0.90, 2.78)		
LMIC (n=39)	1.44 (-0.21, 3.10)			1.11 (0.15, 2.07)		
UMIC (n=25)	-1.35 (-3.36, 0.66)			-0.03 (-1.20, 1.14)		
**By region (n = 105)**						
East Asia & Pacific (n=32)	-0.82 (-2.58, 0.95)			-0.09 (-1.12, 0.93)		
Sub-Saharan Africa (n=33)	2.31 (0.53, 4.09)			2.24 (1.21, 3.28)		
South Asia (n=21)	2.93 (0.80, 5.06)			1.71 (0.48, 2.94)		
Middle East & North Africa (n=8)	0.70 (-2.71, 4.12)			1.14 (-0.83, 3.12)		
Europe & Central Asia (n=4)	-5.87 (-10.80, -0.93)			-2.25 (-5.12, 0.62)		
Latin America & Caribbean (n=6)	1.57 (-2.12, 5.27)			1.37 (-0.76, 3.51)		
