# Supplementary material for: Urban-rural differences in hypertension prevalence in low-income and middle-income countries, 1990–2020: A systematic review and meta-analysis
Source: PLoS Med. 2022 Aug 25;19(8):e1004079. doi: 10.1371/journal.pmed.1004079 (PMC9410549; doi:10.1371/journal.pmed.1004079)
Supplement: S6 Data — (PDF) [file pmed.1004079.s008.pdf]

# S6 Data

**Urban-rural differences in hypertension prevalence in low-income and middle-income countries, 1990-2020: a systematic review and meta-analysis**

## Table of Contents

**Urban-rural difference in hypertension prevalence in 299 surveys, stratified by time, income and region, comparing the main analysis with a random-effects meta-analysis with a three-level multivariate random-effects model ........ 2**

**Study level moderators of urban – rural difference in hypertension prevalence (univariate) ........ 3**

**Meta-regression models and heterogeneity change according to moderator........ 4**

**Urban-rural difference in hypertension prevalence from 1990 to 2020 across six global regions........ 5**

**Urban-rural difference in hypertension prevalence in 299 surveys, stratified by time, income and region, comparing the main analysis (random-effects meta-analysis) with sensitivity analysis (three-level multivariate random-effects model)**

| Moderator | Period | Random effects (Main analysis) N=299 | Three-level meta-analysis (Sensitivity analysis) N=299 |
|---|---|---|---|
| **Overall** | 1990-2020 | 2.49% (1.61, 3.37) | 1.82% (0.47, 3.16) |
| | 1990-2004 | 5.87% (4.15, 7.58) | 4.92% (3.02, 6.81) |
| | 2005-2020 | 1.38% (0.40, 2.36) | 1.03% (-0.31, 2.36) |
| **By income status** | | | |
| LIC | 1990-2020 | 5.67% (4.22, 7.13) | 4.61% (2.92, 6.31) |
| LMIC | 1990-2020 | 2.74% (1.41, 4.07) | 1.30% (-0.34, 2.94) |
| UMIC | 1990-2020 | -1.22% (-2.73, 0.28) | -1.05% (-2.90, 0.81) |
| **By income status and time** | | | |
| LIC | 1990-2004 | 8.16% (5.97, 10.35) | 6.34% (4.00, 8.68) |
| LMIC | 1990-2004 | 2.27% (-0.27, 4.81) | 2.34% (-0.33, 5.01) |
| UMIC | 1990-2004 | 9.26% (2.37, 16.15) | 8.47% (1.26, 15.68) |
| LIC | 2005-2020 | 3.87% (2.01, 5.74) | 3.41% (1.43, 5.39) |
| LMIC | 2005-2020 | 2.90% (1.39, 4.41) | 1.28% (-0.48, 3.04) |
| UMIC | 2005-2020 | -1.72% (-3.21, -0.22) | -1.26% (-3.09, 0.57) |
| **By region** | | | |
| East Asia & Pacific | 1990-2020 | 0.50% (-0.87, 1.86) | 1.08% (-1.31, 3.48) |
| Sub-Saharan Africa | 1990-2020 | 4.24% (2.62, 5.86) | 4.04% (2.20, 5.89) |
| South Asia | 1990-2020 | 7.50% (5.73, 9.26) | 6.62% (3.68, 9.56) |
| Middle East & North Africa | 1990-2020 | 0.72% (-1.93, 3.36) | -0.03% (-3.47, 3.42) |
| Europe & Central Asia | 1990-2020 | -6.04% (-9.06, -3.01) | -6.07% (-9.31, -2.84) |
| Latin America & Caribbean | 1990-2020 | 2.20% (-0.57, 4.97) | 2.24% (-0.72, 5.20) |
| **By region and time** | | | |
| East Asia & Pacific | 1990-2004 | 3.66% (1.07, 6.25) | 3.94% (0.86, 7.02) |
| Sub-Saharan Africa | 1990-2004 | 7.55% (4.10, 11.00) | 7.52% (4.02, 11.02) |
| South Asia | 1990-2004 | 9.33% (6.22, 12.45) | 8.50% (4.81, 12.18) |
| Middle East & North Africa | 1990-2004 | 2.41% (-1.91, 6.73) | 1.80% (-2.79, 6.38) |
| Europe & Central Asia | 1990-2004 | -3.31% (-12.72, 6.09) | -3.46% (-12.94, 6.02) |
| Latin America & Caribbean | 1990-2004 | 10.35% (3.75, 16.95) | 10.38% (3.56, 17.21) |
| East Asia & Pacific | 2005-2020 | -0.62% (-2.16, 0.92) | 0.01% (-2.33, 2.36) |
| Sub-Saharan Africa | 2005-2020 | 3.36% (1.60, 5.13) | 3.19% (1.28, 5.11) |
| South Asia | 2005-2020 | 6.68% (4.63, 8.74) | 5.92% (3.03, 8.82) |
| Middle East & North Africa | 2005-2020 | -0.20% (-3.39, 3.00) | -0.91% (-4.65, 2.83) |
| Europe & Central Asia | 2005-2020 | -6.32% (-9.42, -3.23) | -6.29% (-9.49, -3.10) |
| Latin America & Caribbean | 2005-2020 | 0.58% (-2.37, 3.53) | 0.75% (-2.30, 3.81) |

**Study level moderators of urban – rural difference in hypertension prevalence (univariate)**

| Variable | Level | N of studies | Urban – Rural difference (95% CI ) | P-value for the moderator | $I^2$ | $tau^2$ | P-value for heterogeneity | $R^2$ |
|---|---|---|---|---|---|---|---|---|
| Rural and Urban definition | Used national definition: no | 100 | 2.50% (0.96, 4.03) | 0.995 | 99.68% | 0.00540 | <0.001 | 0.00% |
| | Used national definition: yes | 199 | 2.49% (1.42, 3.56) | | | | | |
| | Used metric: no | 223 | 2.16% (1.14, 3.17) | 0.200 | 99.70% | 0.00538 | <0.001 | 0.10% |
| | Used metric: yes | 76 | 3.48% (1.73, 5.23) | | | | | |
| | Used score: no | 295 | 2.47% (1.58, 3.35) | 0.632 | 99.71% | 0.00540 | <0.001 | 0.00% |
| | Used score: yes | 4 | 4.30% (-3.17, 11.78) | | | | | |
| | Used groups: no | 275 | 2.20% (1.29, 3.11) | 0.026 | 99.70% | 0.00531 | <0.001 | 1.33% |
| | Used groups: yes | 24 | 5.85% (2.78, 8.93) | | | | | |
| Hypertension definition | Included self-reported diagnosis: no | 219 | 2.59% (1.56, 3.62) | 0.715 | 99.71% | 0.00540 | <0.001 | 0.00% |
| | Included self-reported diagnosis: yes | 80 | 2.22% (0.53, 3.92) | | | | | |
| | Included taking anti-hypertensive drug: no | 76 | 3.31% (1.55, 5.06) | 0.291 | 99.67% | 0.00538 | <0.001 | 0.03% |
| | Included taking anti-hypertensive drug: yes | 223 | 2.22% (1.20, 3.23) | | | | | |
| | Converted by equation: no | 278 | 2.55% (1.64, 3.46) | 0.636 | 99.48% | 0.00539 | <0.001 | 0.00% |
| | Converted by equation: yes | 21 | 1.71% (-1.67, 5.08) | | | | | |
| | Adjusted by age/sampling weights: no | 178 | 3.20% (2.06, 4.34) | 0.057 | 99.62% | 0.00533 | <0.001 | 0.90% |
| | Adjusted by age/sampling weights: yes | 121 | 1.47% (0.11, 2.84) | | | | | |
| | Extraction based on number/percentage | 122 | 2.13% (0.75, 3.51) | 0.506 | 99.58% | 0.00540 | <0.001 | 0.00% |
| | Extraction based only on percentage | 177 | 2.74% (1.60, 3.88) | | | | | |
| Number of blood pressure readings | Other (NR or 1 reading) | 68 | 0.73% (-1.10, 2.56) | 0.032 | 99.53% | 0.00532 | <0.001 | 1.24% |
| | ≥2 readings | 231 | 3.01% (2.02, 4.00) | | | | | |
| Method of blood pressure measurement | NR | 30 | 3.73% (0.97, 6.50) | 0.111 | 99.60% | 0.00534 | <0.001 | 0.88% |
| | Other (automatic, semi-automatic) | 137 | 1.48% (0.19, 2.78) | | | | | |
| | Manual | 132 | 3.25% (1.94, 4.57) | | | | | |
| Sampling bias | No | 252 | 2.14% (1.19, 3.09) | 0.061 | 99.71% | 0.00533 | <0.001 | 0.95% |
| | Yes | 47 | 4.48% (2.22, 6.73) | | | | | |
| Detection bias: urban/rural | No | 243 | 2.89% (1.92, 3.86) | 0.058 | 99.68% | 0.00533 | <0.001 | 0.91% |
| | Yes | 56 | 0.70% (-1.35, 2.75) | | | | | |
| Detection bias: blood pressure | No | 285 | 2.45% (1.55, 3.35) | 0.649 | 99.68% | 0.00540 | <0.001 | 0.00% |
| | Yes | 14 | 3.41% (-0.66, 7.48) | | | | | |
| Any bias | No | 210 | 2.37% (1.32, 3.41) | 0.666 | 99.68% | 0.00540 | <0.001 | 0.00% |
| | Yes | 89 | 2.79% (1.16, 4.43) | | | | | |

## Meta-regression models and heterogeneity change according to moderator

| | Moderator | Number of surveys | $I^2$ | $tau^2$ | P-value for heterogeneity | $R^2$ |
|---|---|---|---|---|---|---|
| **Overall** | | | | | | |
| Model 1 | None | 299 | 99.71% | 0.00538 | <0.001 | - |
| **Study features** | | | | | | |
| Model 2 | Use of groups to define urban and rural areas + number of blood pressure readings + sampling bias + detection bias (urban/rural) + prevalence adjusted by age/sex/sampling weights | 299 | 99.53% | 0.00500 | <0.001 | 7.20% |
| **Country status** | | | | | | |
| Model 3 | Model 2 + Region | 299 | 99.25% | 0.00398 | <0.001 | 26.09[illegible] |
| Model 4 | Model 2 + Income classification | 299 | 99.25% | 0.00436 | <0.001 | 19.07[illegible] |
| | **Linear terms** | | | | | |
| Model 5 | Model 2 + Year of starting data collection (linear term) | 299 | 99.42% | 0.00485 | <0.001 | 9.84% |
| Model 6 | Model 2 + HDI (linear term) | 299 | 99.35% | 0.00430 | <0.001 | 20.21[illegible] |
| Model 7 | Model 2 + Proportion of urban population (linear term) | 299 | 99.38% | 0.00449 | <0.001 | 16.52[illegible] |
| Model 8 | Model 2 + Infant mortality rate (linear term) | 299 | 99.31% | 0.00414 | <0.001 | 23.14[illegible] |
| Model 9 | Model 2 + GNI per capita (linear term) | 299 | 99.31% | 0.00426 | <0.001 | 20.85[illegible] |
| | **Restricted Cubic Splines (degrees of freedom)** | | | | | |
| Model 10 | Model 2 + HDI (rcs, 3) | 299 | 99.19% | 0.00413 | <0.001 | 23.21[illegible] |
| Model 11 | Model 2 + Proportion of urban population (rcs, 4) | 299 | 99.22% | 0.00424 | <0.001 | 21.16[illegible] |
| Model 12 | Model 2 + Infant mortality rate (rcs, 4) | 299 | 99.12% | 0.00398 | <0.001 | 26.08[illegible] |
| **Combined** | | | | | | |
| Model 13 | Model 2 + Region + Income classification | 299 | 99.03% | 0.00385 | <0.001 | 28.39[illegible] |
| Model 14 | Model 2 + Region + Income classification + Year of starting data collection (linear term) | 299 | 98.87% | 0.00381 | <0.001 | 29.21[illegible] |
| Model 15 | Model 2 + Income classification + Year of starting data collection + interaction term for income and year | 299 | 98.88% | 0.00430 | <0.001 | 20.20[illegible] |
| Model 16 | Model 2 + region + Year of starting data collection + interaction term for region and year | 299 | 98.95% | 0.00389 | <0.001 | 27.83[illegible] |

rcs = restricted cubic spline

## Urban-rural difference in hypertension prevalence from 1990 to 2020 across six global regions

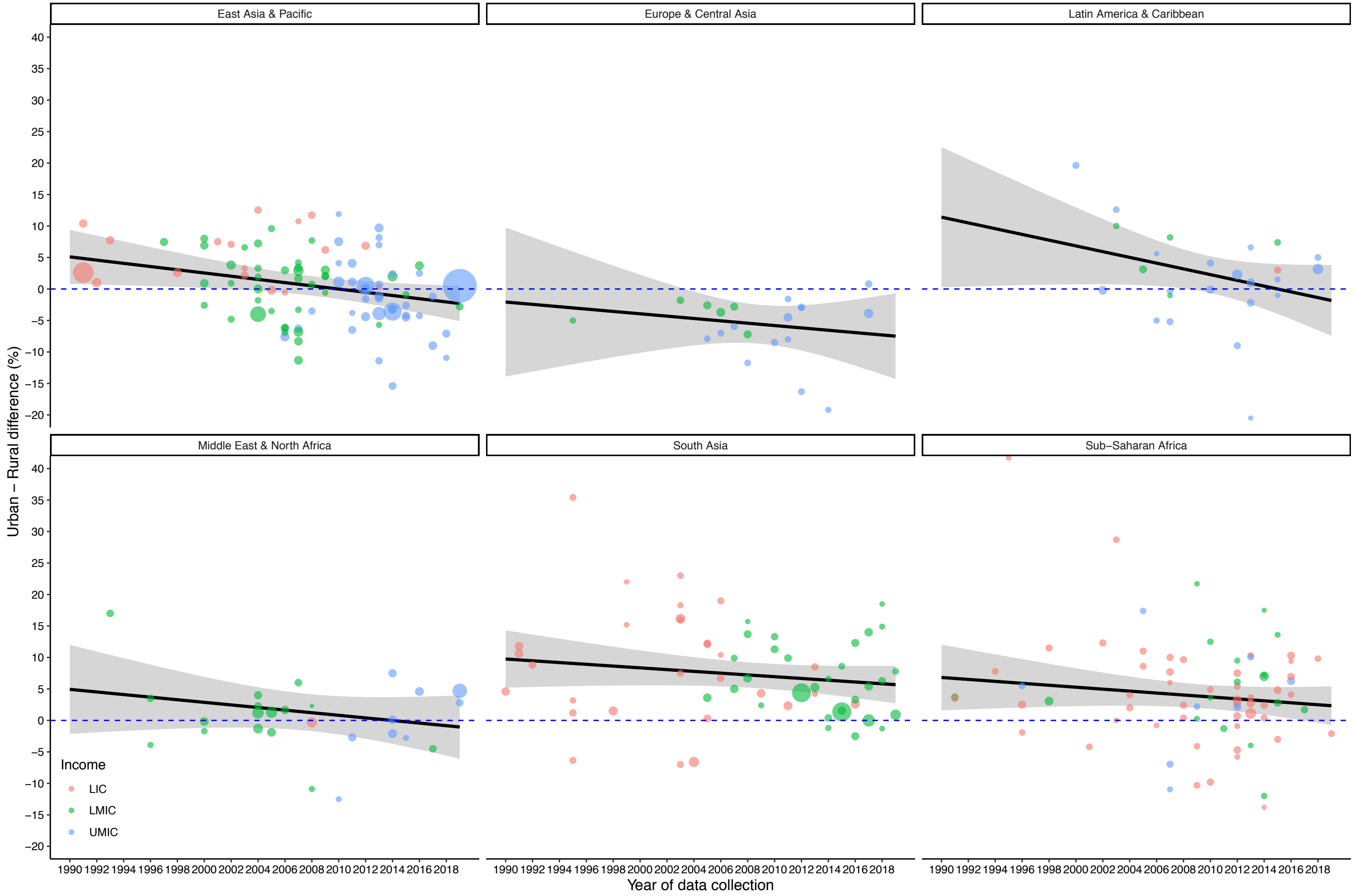


Predicted urban-rural differences from meta-regression model with region, year, and region and year interaction, and five study level features: use of groups to define urban and rural areas, number of blood pressure readings, sampling bias,detection bias (urban/rural) and prevalence adjusted by age/sex/sampling weights. The plot shows the prediction (marginal mean from model-16) of this model varying year from 1990 to 2019 and setting the five study level features to the least biased category (no use of groups, ≥2 readings, probably low risk of sampling bias, probably low risk of detection bias (urban/rural), and adjusted prevalence). Shaded areas represent 95% confidence interval and circle sizes proportional to inverse of variance. LIC: low income; LMIC: lower-middle income; UMIC: upper-middle income countr
