# Supplementary material for: Urban-rural differences in hypertension prevalence in low-income and middle-income countries, 1990–2020: A systematic review and meta-analysis
Source: PLoS Med. 2022 Aug 25;19(8):e1004079. doi: 10.1371/journal.pmed.1004079 (PMC9410549; doi:10.1371/journal.pmed.1004079)
Supplement: Attachment — Submitted filename: reply_editors_reviewers_r1.pdf [file pmed.1004079.s009.pdf]

Requests from the editors:

1) Please revise your title according to PLOS Medicine's style. Your title must be nondeclarative and not a question. It should begin with main concept if possible. Please place the study design only (for example, "A systematic review and meta-analysis") in the subtitle (i.e., after a colon).

ANSWER: We revised the title as suggested:

Urban-rural differences in hypertension prevalence in low-income and middle-income countries, 1990-2020: a systematic review and meta-analysis

2) Please include line numbers in your next draft

ASNWER: We added lined numbers.

3) Abstract:
a) Please ensure that all numbers presented in the abstract are present and identical to numbers presented in the main manuscript text.

ANSWER: We revised the abstract.

b) Please provide both 95% CIs and p values where appropriate.

ANSWER: We revised the abstract. We would like to highlight we used p-values only to evaluate heterogeneity and moderators effect on the meta-regression.

4) The Data Availability Statement (DAS) requires revision. For each data source used in your study:
a) If the data are freely or publicly available, note this and state the location of the data: within the paper, in Supporting Information files, or in a public repository (include the DOI or accession number).
b) If the data are owned by a third party but freely available upon request, please note this and state the owner of the data set and contact information for data requests (web or email address). Note that a study author cannot be the contact person for the data.
c) If the data are not freely available, please describe briefly the ethical, legal, or contractual restriction that prevents you from sharing it. Please also include an appropriate contact (web or email address) for inquiries (again, this cannot be a study author).

ANSWER: We revised our statement. As this is a systematic review, all data are public and available within the paper. We will make the database available in a public repository.

5) Author Summary – Please reduce the length of bullet points to no more than 4 lines per point.

ANSWER: We reduced to no more than 4 lines per point.

6) Thank you for providing the PRISMA checklist. When completing the checklist, please use section and paragraph numbers, rather than page numbers.
a) Please add the following statement, or similar, to the Methods: "This study is reported as per the Preferred Reporting Items for Systematic Reviews and Meta-Analyses (PRISMA) guideline (S1 Checklist)."

ANSWER: We added this statement (lines 157-159, clean version) and updated the checklist with section/paragraph numbers.

7) In line with PLOS Medicine's guidelines, please update your search to the present time.

ANSWER: We updated our search to 10/03/2022, and more than 7.5 thousand titles/abstracts were retrieved. After the full process of screening, we could include 65 new articles (corresponding to 67 new surveys).

8) In figures 2, 3, 4, please show the axis beginning at zero for both graphs. If this is not possible, please show a break in the axis.

ANSWER: We revised **Figures 2**, **3** and **4**, starting the Y-axis at zero. Thanks.

9) Please define the abbreviations in Tables and Figures e.g., LIC, LMIC, UMIC

ANSWER: We now add abbreviations definitions in the footnotes.

10) Please confirm that the appropriate usage rights apply to the use of this map. Please see our guidelines for map images: https://journals.plos.org/plosmedicine/s/figures#loc-maps

ANSWER: We used the public shapefile from World Bank, complying to the Plos guidelines.

https://datacatalog.worldbank.org/search/dataset/0038272

"This dataset is classified as Public under the Access to Information Classification Policy. Users inside and outside the Bank can access this dataset.
License: Creative Commons Attribution 4.0" – S1 Data

11) References:
a) Please ensure that journal name abbreviations consistently match those found in the National Center for Biotechnology Information (NCBI) databases. https://journals.plos.org/plosmedicine/s/submission-guidelines#loc-references.

ANSWER: We revised the Journal names.

12) Please remove the 'Data sharing" and "Declaration of interests" from the end of the main text. In the event of publication, this information will be published as metadata based on your responses to the submission form.

ANSWER: We removed them.

Comments from the reviewers:

Reviewer #1: I confine my remarks to statistical aspects of this paper. The general approach is fine, but I have some issues to reolve before I can recommend publication.

ANSWER: Thank you for your comment and review.

The lack of line numbers made the review process harder.

ANSWER: We apologize for not adding line numbers. We have added them now.

The general approach is OK, but I have some issues to resolve before I can recommend publication.

On p. 2, you give the mean age with a +- figure, but you don't say what this is (SD? Standard error? Something else?) The other numbers are given with 95% CI.

ANSWER: The reviewer is correct. That was for SD, we added this information (line 61, line 288, clean version).

p. 5 When you say things like "leading cause" please also give rates.

ANSWER: We revised the introduction and added the actual number in the sentence about global deaths and revised the sentence about causes of death in LMICs (line 117; 120-121, clean version).

p. 9 I don't fully understand the first sentence in data analysis about dividing it two. Why was this done? Maybe I am missing something.

ASNWER: We apologize for not being clear. We conducted the meta-analysis and meta-regression for the entire period (1990-2020, year defined by the year when the data collection started), and for two defined periods (1990-2004 and 2005-2020, based on the year when the data collection started) using the time-period as a moderator. The cutpoint between the two periods was based on the median year between 1990 and 2020. One of our hypotheses was that the urban-rural difference varied over calendar time, therefore we presented the pooled results in these two time-periods. We revised this statement in the manuscript (lines 236-239, clean version).

Peter Flom

Reviewer #2: The authors provide a review report documenting the urban-rural difference in hypertension in low- and middle-income countries. The strength of this paper is that this is the first systematic review focused on urban-rural differences in the prevalence of hypertension in LMICs. The results highlighted the importance of hypertension prevention and control even in rural areas. There is significant heterogeneity in the methodology of the included studies. There is no discussion about risk factors, awareness, and control of hypertension, which could make this review more beneficial and valuable. My comments are as follows:

ANSWER: Thank you for your comment and review.

Major comments:

1) Time trend analysis using cross-sectional surveys in different populations would have several limitations. Calculating the rate of change using two different populations conducted in different areas may be misleading. Meta-regression may not be the best method for this type of data.

ANSWER: Thank you for highlighting this point. We selected only population-based surveys, with a clear urban-rural contrast and that measured simultaneously urban and rural areas aiming to increase their comparability. However, we agree that the time-trend analysis has this limitation and further discussed it in the revised discussion (lines 491-495, clean version).

2) Different studies are using different definitions of rural and urban areas. Would it be possible to do a sensitivity analysis by including studies that used the consistent definition?

ANSWER: We evaluated the influence of urban-rural definitions in the urban-rural prevalence difference using four main groups (**S6 Data**): use of national definition (census); used of any metric, use of a derived composed score and use of certain groups to define rural areas (e.g., farmers). The estimates for those using census, metrics and scales were consistent, the only definition that explains part of the heterogeneity was use of certain groups ($p=0.026$).

We also evaluated the urban-rural definitions in terms of risk of bias: 56 (19%) surveys were classified as probably high risk of bias in the domain of urban-rural definition. This risk of bias influenced the urban-rural prevalence difference (Low risk: 2.14%, 95% CI, 1.19 to 3.09; High risk: 0.70%, 95% CI, -1.35 to 2.75, **S6 Data**, in univariate analysis). In the revised version the estimates from the models (**Figure 2**, **3** and **4**) accounted for use of groups, risk of bias in sampling and urban-rural definition, number of blood pressure readings and whether the prevalence was adjusted by age-sex/sampling weights. Predictions were made based on a model including only: no use of groups; low risk of bias in the sampling and urban-rural definition; ≥2 blood pressure readings; and adjusted prevalence.

We now highlight these results in the revised version (lines 356-363, clean version and S6 Data).

3) It would be great if authors would also have data on risk factors. Why authors did not extract them? A reasonable explanation for rural-urban variation is incomplete.

ANSWER: We discussed this point in depth during the planning phase and protocol definition. We decided not to include risk factors or risk factor adjusted multivariable estimates (e.g, OR) for several reasons. The primary reason was that we aimed to describe the phenomena of urban-rural differences from a public health decision making perspective (e.g. health service planning, resource allocation), rather than focusing on aetiology or specific mechanisms underlying the phenomena, which might be very heterogenous and context dependent. Second, very few studies reported adjusted estimates, and even fewer reported comparable risk factors beyond age and sex. We would not have been able to maintain the global coverage of LMICs, an important focus of the study, with the available risk factor adjusted data. Third, due to the large number of studies, detailed extraction of risk factors other than age and sex and their heterogeneity would have been very challenging from a practical point of view.

Minor comments:

5) Presenting the prevalence of hypertension in terms of range would be easier for many readers.

ANSWER: Thanks for this suggestion. We re-wrote the paragraph of hypertension prevalence in terms of range (lines 298-306, clean version).

6) The authors presented results for HDI and IMR but the detailed methodology is missing. Which year HDI and IMR were used? Did the author account for data collection year or paper published year?

ANSWER: We apologize for not being clear. We matched each indicator (HDI, IMR, Urban Population, GNI) information by year of starting data collection for each survey. We clarified this in the methods of revised version (lines 232-233, line 238, clean version).

7) What do you mean by representing 87% of LMICs population by 2018?

ANSWER: We analysed surveys from 66 LMICs (updated search) based on samples targeting the general population. Assuming the surveys are generalizable to the national population, the estimates apply to a total population in these 66 LMICs (2018) representing 88% (updated search) of the estimated population of all LMICs in 2018, as per the World Bank information. However, we deleted this statement so as to avoid confusion.

8) As the authors had discarded non-English papers it would be interesting to know how much data was missed.

ANSWER: After the title/abstract screening phase, we discarded 43 (43/1,309, 3.3%) potential full-texts because of language (**Figure 1**). We do not expect that inclusion of non-

English papers would have changed our results. We add this as a limitation (lines 484-487, clean version).

9) What was the response rate for each study? Did the studies use a random sampling technique for sample selection? It would also be interesting to know whether data were collected at home or hospital setting or elsewhere.

ANSWER: We included only population-based surveys, and so the sampling strategy was carefully evaluated. All studies used a valid technique for sampling (random sampling, multistage sampling, self-weighted sampling), the WHO Steps recommendation, or they captured the population serving a local area (like studies evaluating rural villages). We excluded studies that recruited specific populations, such as attending hospitals, convenience sample, etc (85 studies - Specific population, **Figure 1**). We added this information in the methods (lines 171-172, clean version)

Regarding response rates, we did not systematically extract these data, but we did evaluate them, where reported by the authors, in our evaluation of risk of bias in sampling (e-Methods: Risk of Bias assessment, **S1 Protocol**). Additionally, 121 (40%) surveys were standardized by age-sex and/or adjusted by the sampling weights.

10) Self-reported data on the diagnosis of hypertension is interesting in the context of LMICs. Would it be possible for authors to run a sensitivity analysis by excluding those who self-reported the diagnosis of hypertension?

ANSWER: We conducted this analysis (**S6 Data**) and the urban-rural difference did not differ between surveys including self-reported diagnosis (n=80) in the definition and those that did not include self-reported diagnosis (n=219) (included: 2.22%, 95% CI, 0.53 to 3.92; not-included: 2.59%, 1.56 to 3.62, p-value: 0.715). To avoid any bias between urban and rural areas regarding self-reported diagnosis / awareness, we did not included studies that reported only self-reported diagnosis, so surveys must have SBP/DBP measured AND self-reported diagnosis or antihypertensive medications, as per our inclusion criteria. We included these results in lines 363-366, clean version.

Reviewer #3: This is an interesting review highlighting urban-rural variation in hypertension prevalence in low- and middle-income countries (LMICs). Especially given the ongoing double and or triple burden of diseases in LMICs and the rapid urbanisation taking place in these settings.

The introduction, data analysis, results, and discussion sections were well written and I noted the comparisons with previous regional reviews.

ANSWER: Thank you for your comment and review.

However, I will strongly recommend that the authors update their search as they conducted their search from 01/01/1990 to 01/05/2018, their search is almost four years old and although publications in the last two years might have been dominated by the COVID-19 pandemic, I suggest that the search be updated to at least 31/12/2021.

ANSWER: We updated our search to 10/03/2022, and more than 7.5 thousand titles/abstracts were retrieved. After the full process of screening, we could include 65 new articles (corresponding to 67 new surveys).

Otherwise, this study has the potential to influence public health decision making and resource allocation towards interventions targeting prevention and management of hypertension in LMICs.

ANSWER: Thank you.

Reviewer #4: This is a well-written manuscript. I have a few general comments:

ANSWER: Thank you for your comment and review.

1. The author may need to include non-English articles as a lot of low-and middle-income countries (China, Iran, Vietnam) are non-English speaking countries.

ANSWER: We agree with the reviewer this is a limitation of our review. After the title/abstract screening phase, we discarded 43 (43/1,309, 3.3%) potential full-texts because of language (**Figure 1**). In the revised version acknowledged that in the discussion (lines 484-487, clean version).

2. In the method section, I would suggest justifying the reason for including the country-level socio-economic variables in the regression model.

ANSWER: Thank you for the opportunity to improve our manuscript. We add the reasons in the methods (lines 222-285, clean version).

3. Figure 3 indicates the large heterogeneity in HDI and other indicators among countries our different income groups. Does the control of the region, income status (LIC, LMIC, UMIC,) and country-level socio-economic are controlled in the regression lead to overfitting?

ANSWER: That is an important point. We agree with the reviewer and for this reason all models in **Figure 2**, **3**, and **4** included only one variable describing the country. The models were further adjusted by study characteristics (risk of bias in sampling and urban-rural definition, use of groups to define urban-rural, whether the prevalence was adjusted by age/sex/sampling weights and number of blood pressure measurements). The figures have country income status differently coloured just for illustration and it is not adjusted for it. We clarified these points (lines 265-266, clean version, Figures legend).

4. Given the heterogeneity in Urban-rural differences in hypertension across countries and regions, the author adjust the fixed effect of countries and/or regions? have the author considered the three-level meta-regression?

ANSWER: Thank you for this suggestion. We conducted a sensitivity analysis fitting a three-level meta-regression considering the country and study as multi-level. The results are broadly consistent with the main analysis. We included it in Supplementary material in the revised version (**S6 Data**) (lines 247-250; 346-349; clean version, S6 Data)

Moderator	Period	Random effects<br>(Main analysis)<br>N=299	Three-level meta-analysis<br>(Sensitivity analysis)<br>N=299
**Overall**	1990-2020	2.49% (1.61, 3.37)	1.82% (0.47, 3.16)
	1990-2004	5.87% (4.15, 7.58)	4.92% (3.02, 6.81)
	2005-2020	1.38% (0.40, 2.36)	1.03% (-0.31, 2.36)
**By income status**			
LIC	1990-2020	5.67% (4.22, 7.13)	4.61% (2.92, 6.31)
LMIC	1990-2020	2.74% (1.41, 4.07)	1.30% (-0.34, 2.94)
UMIC	1990-2020	-1.22% (-2.73, 0.28)	-1.05% (-2.90, 0.81)
**By income status and time**			
LIC	1990-2004	8.16% (5.97, 10.35)	6.34% (4.00, 8.68)
LMIC	1990-2004	2.27% (-0.27, 4.81)	2.34% (-0.33, 5.01)
UMIC	1990-2004	9.26% (2.37, 16.15)	8.47% (1.26, 15.68)
LIC	2005-2020	3.87% (2.01, 5.74)	3.41% (1.43, 5.39)
LMIC	2005-2020	2.90% (1.39, 4.41)	1.28% (-0.48, 3.04)
UMIC	2005-2020	-1.72% (-3.21, -0.22)	-1.26% (-3.09, 0.57)
**By region**			
East Asia & Pacific	1990-2020	0.50% (-0.87, 1.86)	1.08% (-1.31, 3.48)
Sub-Saharan Africa	1990-2020	4.24% (2.62, 5.86)	4.04% (2.20, 5.89)
South Asia	1990-2020	7.50% (5.73, 9.26)	6.62% (3.68, 9.56)
Middle East & North Africa	1990-2020	0.72% (-1.93, 3.36)	-0.03% (-3.47, 3.42)
Europe & Central Asia	1990-2020	-6.04% (-9.06, -3.01)	-6.07% (-9.31, -2.84)
Latin America & Caribbean	1990-2020	2.20% (-0.57, 4.97)	2.24% (-0.72, 5.20)
**By region and time**			
East Asia & Pacific	1990-2004	3.66% (1.07, 6.25)	3.94% (0.86, 7.02)
Sub-Saharan Africa	1990-2004	7.55% (4.10, 11.00)	7.52% (4.02, 11.02)
South Asia	1990-2004	9.33% (6.22, 12.45)	8.50% (4.81, 12.18)
Middle East & North Africa	1990-2004	2.41% (-1.91, 6.73)	1.80% (-2.79, 6.38)
Europe & Central Asia	1990-2004	-3.31% (-12.72, 6.09)	-3.46% (-12.94, 6.02)
Latin America & Caribbean	1990-2004	10.35% (3.75, 16.95)	10.38% (3.56, 17.21)
East Asia & Pacific	2005-2020	-0.62% (-2.16, 0.92)	0.01% (-2.33, 2.36)
Sub-Saharan Africa	2005-2020	3.36% (1.60, 5.13)	3.19% (1.28, 5.11)
South Asia	2005-2020	6.68% (4.63, 8.74)	5.92% (3.03, 8.82)
Middle East & North Africa	2005-2020	-0.20% (-3.39, 3.00)	-0.91% (-4.65, 2.83)
Europe & Central Asia	2005-2020	-6.32% (-9.42, -3.23)	-6.29% (-9.49, -3.10)
Latin America & Caribbean	2005-2020	0.58% (-2.37, 3.53)	0.75% (-2.30, 3.81)
