# Supplementary material for: Urban-rural differences in hypertension prevalence in low-income and middle-income countries, 1990–2020: A systematic review and meta-analysis
Source: PLoS Med. 2022 Aug 25;19(8):e1004079. doi: 10.1371/journal.pmed.1004079 (PMC9410549; doi:10.1371/journal.pmed.1004079)
Supplement: Attachment — Submitted filename: reply_editors_reviewers_r2.docx [file pmed.1004079.s010.docx]

**Requests from the editors:**

**Requests from Editors:**

**1)	Abstract: Methods and findings – please add inclusion and exclusion criteria for studies**

**ANSWER:** We add inclusion and exclusion criteria in the abstract.

**2)	Abstract: please move the following statement "This systematic review and meta-analysis has been registered with PROSPERO (CRD42018091671) from the "Conclusions" to the "Methods and findings" section.**

**ANSWER:** We move the sentence to “Methods and findings”.
